# What are the challenges that social prescribers face when supporting people within dementia and how can these be addressed? A qualitative study

**DOI:** 10.1371/journal.pone.0317749

**Published:** 2025-01-17

**Authors:** Claudio Di Lorito, Marie Poole, Greta Rait, Kritika Samsi, Louise McComiskie, Louise Robinson, Jane Wilcock

**Affiliations:** 1 Research Department of Primary Care & Population Health, Centre for Ageing Population Studies, University College London, London, United Kingdom; 2 Population Health Sciences Institute, Faculty of Medical Sciences, Newcastle University, Newcastle, United Kingdom; 3 NIHR Health and Social Care Workforce Research Unit, The Policy Institute, King’s College London, London, United Kingdom; 4 Age UK Barnet, London, United Kingdom; University of the Witwatersrand Johannesburg, SOUTH AFRICA

## Abstract

**Introduction:**

Evidence suggests that social prescribing might have a positive impact on identity, control, creativity and quality of life in people with dementia. While evidence on the benefits of social prescribing is accumulating, there is a sparsity of research on the experiences of social prescribers. This study aims to identify the challenges that social prescribers face when supporting people with dementia and their families and strategies to address these.

**Materials and methods:**

A qualitative study involving 24 social prescribers from all regions in England. Semi-structured interviews investigated challenges and strategies that social prescribers experience in their own practice. Data were analysed through thematic analysis. Results on “Challenges” and the respective “Strategies” are presented in a chronological order that reflects the different stages of contact with and support for the client with dementia, from referral to discharge.

**Results and discussion:**

This study identified unique barriers that social prescribers face when working with people with dementia, particularly around communication, motivation, engagement and overdependency. It identified person and system-level strategies that can be used to address these challenges. These include expanding opportunities for dementia training, offering in-person support, including social prescribing in annual dementia reviews, and increasing integration of services within Integrated Care Systems and collaborations between health care service providers and with the third sector. Improving delivery and effectiveness of services is crucial to ensure that social prescribing fulfils its ethos of personalised care approach for all, including people with dementia, as envisioned in the NHS long term plan.

## Introduction

General practice is described as the bedrock of the National Health Service (NHS) in England, but growing demand, an ageing population, and more people with complex or multiple long-term conditions have increased General Practitioners’ (GPs) workload [1]. To meet these challenges and provide universal personalised care, care is being delivered by multi-disciplinary teams including social prescribers [2, 3].

The concept and practice of social prescribing was set out in the United Kingdom through the National health System (NHS) Long Term Plan [4]. The plan was published by NHS England in 2019, and established priorities in healthcare service provision for the following ten years [4]. Social prescribing has since then also gained momentum internationally [5]. While social prescribing around the world presents with different terminologies and practices, the aims and structural components are shared across the globe [6].

Social prescribing attends to all needs that are non-medical, e.g., social, and psychological [7]. It is based on the growing recognition that non-medical factors (i.e., social determinants) such as education, income, and housing have a direct impact on health behaviours and outcomes [8, 9]. In social prescribing, a client (or patient) is referred to a social prescribing team or worker [7]. There are different terminologies adopted to designate social prescribers including link workers, health and wellbeing coaches, care coordinators, community connectors, advisors, support brokers, navigators, or health workers [7]. The core role and remit, however, is similar [7].

Upon referral, the social prescriber develops in collaboration with their client a personalised support plan based on the question ‘*what matters to the person*?’ This tailored approach allows signposting the client to resources in the community that respond to their own individual non-medical needs [3]. Some examples of resources that clients are linked with include exercise classes, art groups, gardening clubs, peer support, social care teams, benefits and financial support, and voluntary organisations [5].

Social prescribing is an all-age, whole population approach, and evidence on its benefits is mounting. A systematic review on the impact of social prescribing on service users from the general population found improvements in health and wellbeing, health-related behaviours, self-concepts, feelings, social contacts and day-to-day functioning [10]. Research also found a reduction in appointment rates and health service usage, with one study reporting a short-term reduction (i.e., after four months) of healthcare usage as a result of referral and use of social prescribing schemes [11]. The mechanism for reduced healthcare usage was proposed in a realist review, suggesting that link workers represent a vehicle for accruing clients’ confidence, motivation, connections, knowledge and skills, thereby reducing their reliance on GPs [12]. The benefits of social prescribing can be especially important for communities who experience long term / progressive conditions [13], resulting in reduced opportunities to engage in social activities, isolation and loneliness, poor mental, emotional wellbeing, and quality of life [14].

These include people with dementia and their families. Evidence on social prescribing as an effective synthesis able to address social determinants of health while potentially reducing healthcare demand and costs in people with dementia is growing [15]. Research suggests that personalised social and community-based support might have a positive impact on identity, control, creativity, and quality of life for people with dementia [16, 17]. A Randomised Controlled Trial showed statistically significant improvements in depression and quality of life in people with dementia as a result of attending a social support group [18]. Another study found that referral to weekly arts from the onset of symptoms empowered individuals and family carers to maintain fulfilling active life in the community and thereby relieved emotional strain and isolation [19]. A systematic literature review on the effects of community-based arts and health interventions found that arts-based activities had a positive impact on cognitive processes, in particular on attention, stimulation of memories, enhanced communication and engagement with creative activities [20]. A study evaluating the impact of a Dementia-Friendly Exercise Class on People with Dementia found Positive effect on loneliness, mood and cognition [21].

Current evidence and service intentions, therefore, suggest that social prescribers may have potential to address a range of non-medical care needs, and support integrated care, personalisation and planning across primary, social and community care systems. Approaching complex challenges in this way would meet the key challenges of tackling inequalities in outcomes, experience, and access, supporting people to stay well and independent, helping those with preventable conditions, supporting those with long-term progressive conditions and in optimising collective resources [22].

While evidence on the benefits of social prescribing is accumulating, we were unable to identify research on dementia social prescribing from the individual perspective of social prescribers. There are unique barriers and challenges in working with clients with dementia, and evidence on what these are, and strategies that can be used to address these, is currently missing in order to inform good practice in this area. This paper aims to address this evidence gap in dementia social prescribing and help inform good clinical practice by answering the following questions:

1. What are the challenges and barriers that social prescribers face when supporting people with dementia and their families?
2. What are possible strategies through which these challenges can be addressed?

## Methods

This was a qualitative study comprising semi-structured individual interviews. It obtained ethics approval from the UCL Research Ethics Committee (ID: 25445/001). Findings are reported according to the COnsolidated criteria for REporting Qualitative research [COREQ] criteria [23].

### Sample and recruitment

The inclusion criteria were:

- Self-identifying as a social prescriber (e.g., social prescribing link worker, health and wellbeing coach, care coordinator, community connector, community advisor, community support broker, community navigator or community health worker).
- Experience of social prescribing with people with dementia and their families.
- Working in statutory (primary and secondary healthcare services, local councils), private (homecare services) or voluntary sectors (charities, grassroot organisations).
- Any locality within England.

We recruited through:

- Existing professional and private networks of the study team.
- Development of new network/connections with relevant researchers/professionals/groups.
- Identification of Primary Care Networks (PCNs), voluntary sector organisations (charities), private sector (homecare providers) and local councils that offer social prescribing services nationally through Google searches.
- Registration to professional networks (e.g., The National Academy of Social Prescribing, NASP) and advertised the study in their outputs (newsletters, blogs).

Recruitment was initially undertaken using snowball sampling [24], by asking identified professionals to circulate information about the study within their own networks. This was to pragmatically address the challenges of involving a very busy workforce in research. However, as data collection progressed, we adapted our sampling and employed a purposive sampling strategy [24] to make the study sample as diverse as possible. We scoped participants’ professional role, location, and demographics (gender, age group, ethnicity) to identify areas of underrepresentation, and when recruiting new participants, we prioritised demographic and professional characteristics that were under-represented at the time. We pursued diversification guided by the principle that social prescribers experience different barriers, challenges and benefits based on geographical areas where they operate (e.g., urban vs rural/coastal, larger vs smaller catchment areas), professional role and organisation they work for (e.g., working for the statutory vs third sector), and demographic characteristics (e.g., white male vs black woman) which may affect clients’ responses.

Recruitment started on 14 June 2023 and continued until data sufficiency was reached [25] on 14 November 2023. Given the lack of guidance around data saturation for sample sizes in qualitative research (e.g. when it is reached), in line with Guest, Bunce and Johnson [26], we adopted the concept of ‘conceptual density’ (i.e. gathering data until a *sufficient depth* of understanding of the domains under investigation is reached) [27].

Initial contact with potential participants was made by email. The email contained an information sheet outlining the study, what taking part would involve and the voluntary nature of participation, a consent form and a demographic form. The potential participant was advised to contact the research team if interested in taking part and return the signed consent form. The team would then make sure the person met the inclusion criteria and if so, agreed a time/date for the interview.

### Data collection

Individual semi-structured interview through Microsoft Teams were undertaken by CDL and recorded. The interviews were based on a topic guide informed by a scoping review [28] of existing literature and co-designed with the research team and the social prescriber collaborator (Table 1). The scoping review was undertaken on Google scholar by inputting the terms “social prescribing” and “dementia”. Results were screened and a list of emerging themes was created through inductive thematic analysis [29] by CDL. The emerging themes were presented to the study team and the PPIE co-applicants, as well as our social prescriber study collaborator (LMC) to sense check them and confirm their relevance/appropriateness. The discussion resulted in four final themes: Information and access to social prescribing in participant’s service; type of clients’ needs, and support offered; Confidence and skills; Resources and support. In the same session, questions were co-developed for each of the theme.

**Table 1:** Topic guide.

**Notes for interviewer**	Thank the person for taking part. Introduce yourself and give a brief reminder of the study. Ask if the person has any questions. Take/review completed consent. Seek confirmation of permission to audio record the interview. Remind participant they can take a break or stop at any time
**Topic 1: Information and access to social prescribing in participant’s service**	1. How do clients hear and learn about social prescribing? 2. How does/do the person(s) you support get started with social prescribing? 3. Who initiates the process of getting support from you (social prescribing link worker)? 4. Who arranges/organises it for the person? 5. Do you think that the process of gathering information and accessing social prescribing is working? What can be done to improve this process?
**Topic 2: Type of clients’ needs and support offered**	6. Can you tell me about the person(s) with dementia who you support and what social needs they have? 7. Based on these social needs, what type of social activities do they do, if any? 8. Which of these social activities are facilitated / were suggested by you (social prescribing link worker)? 9. How are these facilitated, i.e., how do you (social prescribing link worker) support the person with their social activities?
**Topic 3: Confidence and skills**	10. How do you think that your clients (both with dementia and caregivers) find the support that you offer? 11. Do you feel that you confidently understand the social needs of your client with dementia and their carer(s)? How do you try to understand their needs? 12. Are the activities offered tailored to the person’s needs and those of carers? How do you personalise the support you provide to your clients’ needs? 13. What are some of the skills and knowledge required to better support clients with dementia and their caregivers to fulfil their social needs? 14. How do you think your confidence and skills can be boosted to better support your clients?
**Topic 4: Resources and support**	15. What kind of resources and support are there in place to support you in your role? 16. What can be better done to make you a more confident social prescribing link worker in terms of, e.g., guidance, mentoring, training, support, logistics? 17. What information do you think it is important to include in a new resource supporting social prescribing link workers to better support clients with dementia and caregivers? 18. What format should this resource have? (e.g., phone app available offline; website; resource pack)
**Final remarks**	Is there anything else you think is important to tell us about information/support for social prescribing link workers to better support people with dementia and their families/friends that we haven’t already talked about?
**End**	Thank you for taking part. Can we come back to you when we are doing discussion groups?

The topic guide was used to prompt an open-ended guided discussion focusing on challenges and strategies of participants in their own practice, but was used flexibly, so that if and when new relevant themes and topics emerged during the interview, they were explored further.

### Data analysis

Audio files of participants’ interviews were securely transferred to a university-approved transcription agency, which transcribed and fully anonymised them. Transcriptions’ anonymity was double checked by the research team, who assigned participants pseudo anonymised codes (Table 2). The transcripts were analysed through inductive thematic analysis [28], setting two themes based on the guiding study research questions:

1. Challenges and barriers (for social prescribers).
2. Strategies (used to address challenges and barriers).

**Table 2:** Participants’ characteristics.

ID	Ethnicity	Location	Occupation
SP01	Asian or Asian-British	Yorkshire and the Humber	SPLW
SP02	White British	Northeast	Care Coordinator
SP02	White (any other)	East Midlands	SPLW
SP03	White British	East Midlands	SPLW—Dementia Project Lead
SP04	Black, Black British, Caribbean or African	London	SPLW
SP05	White British	West Midlands	Senior Public Health Officer
SP06	White British	Northeast	Senior Health—Well Being Dementia Lead
SP07	White British	Yorkshire and the Humber	SPLW
SP08	White (any other)	West Midlands	Head of Service, Wellbeing & Care
SP09	Black, Black British, Caribbean or African	London	SPLW
SP10	White British	Yorkshire and the Humber	Dementia Coordinator
SP11	Black, Black British, Caribbean or African	London	SPLW
SP12	White British	Northeast	SPLW
SP13	White British	Northeast	Community Wellbeing Manager
SP14	Asian or Asian-British	Northwest	Consultant Psychiatrist
SP15	White (any other)	East of England	SPLW
SP16	White British	West Midlands	Health and Wellbeing Coach
SP17	White British	East of England	SPLW
SP18	White British	East of England	SPLW
SP19	White British	Yorkshire and the Humber	Senior Project Lead—Dementia Coordinator
SP20	White British	London	SPLW
SP21	White (any other)	East of England	Arts and Health Officer
SP22	White British	London	SPLW
SP23	White British	Southwest	SPLW
SP24	White British	Southeast	SPLW

CDL inductively coded data within one of these two themes. The coding frame was presented by CDL in a data clinic meeting to the core research team (JW, MP) to reach consensus on its accuracy (i.e., that every challenge/barrier and strategy presented by participants in the interviews had been identified and coded into theme 1 or 2).

Because of the large amount of data within each theme, the core research team decided to re-arrange the data around challenges/barriers and strategies and present it based on “stages of support”, i.e., from pre-contact with clients through to discharge. This was done within two separate follow-up group meetings on Zoom. The first was held with three social prescribers who had not taken part in the individual interviews, and who were recruited via the research team’s professional network. In this meeting, the data was presented to the social prescribers, who identified seven different stages of support that the challenges/barriers and strategies fit into:

1. Referral and preparatory groundwork.
2. First approach.
3. Nurturing motivation and rapport.
4. Identifying clients’ needs.
5. Identifying resources and signposting to community-based assets.
6. Continued engagement of clients.
7. Termination, discharge and follow up.

The group also identified an overarching theme:

8. Ongoing professional development.

All data on challenges/barriers and strategies were then coded into one of these eight themes by CDL. A second meeting was held with three Patient and Public Involvement and Engagement (PPIE) members with lived experience of dementia. In the meeting, the coding frame and analysis was presented to the group, discussed and refined based on participants’ feedback. The group agreed with the framework, but felt that some of the themes’ names should be renamed by using jargon-free language, to enhance clarity and understanding, as follows:

1. Pre-contact groundwork and preparation.
2. First contact.
3. Developing motivation and rapport.
4. Identifying needs.
5. Identifying and signposting to resources.
6. Engaging the client in social prescribing.
7. Discharge and continuity of care.
8. Ongoing professional development.

Data are presented below by theme, as revised by the PPIE members.

## Results

Twenty-four social prescribers were approached, and all agreed to take part in the study (participants’ characteristics in Table 2). Participants were interviewed over six months between June and November 2023. The interviews generated 951 minutes of data. The average length of interviews was 38 minutes (range: 17–49).

Results on “Challenges” and the respective “Strategies” (for the full list see Table 3) were organised in a chronological order that reflects the different stages of contact with and support for the client with dementia, from referral to discharge. Interview quotations below represent only part of the large amount of data generated in the study, which is fully reported in the S1 File.

**Table 3:** Challenges and respective strategies by stage of support.

Stage in the support process	Challenges	Strategies
**Pre-contact/referral**	You lack confidence/experience in dementia.	Consider expertise within your team to get guidance. In some cases, social prescribers have found it helpful to pass the referral to a more experienced colleagues in their team
		Be pro-active, not reactive. Do some preliminary research (online and in person), finding the charities or the statutory services that your client can be signposted to. In most instances your locality will have a web-based directory where you can find what is going on. Sometimes, GPs, PCNs, or charities have directories that they have collated, or the information might be available if you speak with others in the team
		See the section on ongoing professional development at the end of this table
	You are passed on a referral with little to no information on the client’s situation	If you work in PCNs, get access to the GP records. You can see what was said during the consultation and what prompted the referral. However, check with the client that the need identified in the original referral is still unaddressed or if anything has changed, as they might need something else
		If you work in a charity, you will be relying on information relayed to you by the client, which may be inaccurate. Consider involving caregivers and families in the conversations
	Your referral is placed in a long waiting list, due to the amount of referrals you receive	Prioritise referrals in a matter of urgency. Urgency could be risk of homelessness, no food, no gas and electric. High priority could be struggling financially (because of lack of attendance in place). Moderate could be an interest in social groups or activities
	You are unsure who you will speak to when contacting the client	You may want to explore whether legal arrangements are in place, such as lasting power of attorney or who gave consent in the referral process. This will clarify who you will be having discussions with and most importantly who will have a final say in any course of action
	You are unsure about what is going on in the community to support your client	Social prescribers often report feeling “Jack of all trades, masters of none”. You may want to consider signing up to newsletter and mailing lists of different relevant organisations such as the National Academy for Social Prescribing: https://socialprescribingacademy.org.uk/. Getting in touch at intervals with organisations, to see if there is anything new that you have missed, is also helpful
	You want to make sure that the client knows you are going to contact/ring them	You may want to consider posting a letter via regular mail. Not only are older generations more familiar with regular mail than e-mail, but also it is best for people with dementia to have a physical readily available reminder about the appointment
**First contact**	You realise that the client has communication issues (e.g., will not be able to relay accurate information)	Consider going back to the GP and asking them to make a referral for the caregiver, so you can support the couple. Remember that dementia is a systemic issue that does not affect the person only, but also (and sometimes even more) the caregiver and family. Whilst operating in a person-centred approach is key to social prescribing, it is equally important not to overlook the carer and how their health and wellbeing also affects the person with dementia
		Suggest a face-to-face appointment, either at the surgery (saves time and resources), at the client’s home (if the person cannot travel) or at a community place. Especially in cases of mid to late-stage dementia, it is advisable to have the carer and family present
		Be aware that there may language barriers with non-native English speakers. The client may have reached a point in their life where they spoke great English, but because of the dementia they are now reversing back to their native language. You may want to consider using a translator (if available), although some find it more effective to involve a family member because they know the person better
	You realise that the client may have issues around capacity	It might be advisable to close the referral, let the GP know and push forward for a mental capacity assessment. This will allow for appropriate support arrangements to be in place, e.g., appointing a mental capacity advocate on the client’s behalf
	You realise that your client may have dementia, but a diagnosis is not in place	Social prescribers have reported instances where they started working with a client without a formal diagnosis of dementia, and then realised that actually they do not retain the information from previous consultations, or they may seem a little bit confused. At the same time, it is very hard, especially when people reach a certain age, to know what normal forgetfulness or normal changes in behaviour is and what is actually a dementia concern. It is advised to refer the client for a memory assessment, to initiate the process of getting a diagnosis, because that is the starting point to make them eligible for further support. Remember, no diagnosis equals no adequate support in place
	You are unsure whether to use the word “dementia” or not	Be aware that not every person with dementia wants to hear or use the term “dementia”. In fact, in some language, the word “dementia” does not exist. Sometimes, you may read information in the client’s referral (or notes) or have a feeling or doubt about whether it is appropriate to use the word dementia. It may be helpful to involve the caregiver or family of the person to learn about if and how dementia needs to be approached and discussed, if at all. Sometimes, to offer support, you do not need to use the word at all.
	The client or the caregiver (family) refuses your support when you first contact them	Consider offering a six-month courtesy call. Be aware that your client (and their carers) may have been referred to you upon receiving a diagnosis and be still trying to absorb all the information and work out their grief: https://www.alzheimers.org.uk/get-support/help-dementia-care/understanding-denial-lack-of-insight
		Consider offering to send an information pack via email or the post, with relevant information and contacts, so the person can get back to you in their own time. The info pack needs to have information accessible for both the person with dementia and the carer, and always be up to date.
		Be aware that people with dementia experience “rejection to care” (https://www.scie.org.uk/dementia/living-with-dementia/difficult-situations/refusing-help.asp). Also, their cognition fluctuates depending on days and times of the day. You may find it helpful to offer to call back on another day, potentially trying at a different time or to talk to a carer or family member. Sometimes, it might be helpful to use terminology a little bit differently to kind of fit the person’s needs, to kind of ease them in. For example, a client may say that all they need is a cleaner. So instead of presenting yourself as a social prescriber, try to say you are there for the purposes of cleaning (https://www.alzheimers.org.uk/blog/lying-to-someone-with-dementia). Ultimately, an initial “no” may not be definitive, so do not close the referral at first rejection, but try a couple of times more
		You may want to consider that the original referral came at a point of crisis, and by the time you make contact with the client, the crisis may have resolved. Sometimes people just need to know there is someone if they need it
		Try to describe your role rather than saying the name of the role. A lot of people who hear the word social prescriber may confuse it with social worker or someone who prescribes medications
		Ultimately, remember that you cannot impose support on anyone, so it is the client’s decision whether they want to engage.
	The client does not remember they have been referred to you or having consented to the referral	You may want to go back to the person who originally referred the client (e.g., GP) to double check.
**Building trust and rapport**	You are anxious that you may not be able to develop rapport with your client	Try to establish an equal relationship, conveying the message that you are a partner in care and that you and your client are a team. Show them you are human, that you do not know everything about anything (e.g. saying “I don’t know what that is, but I’ll go and find it out with you and we will do some research together”.
		You may want to be aware that your first call(s) need to be longer with a client with dementia. So be flexible and dedicate a good set of time to the call. Be open to have a bit of like a general chit chat first, to talk about different topics, and that you are actively listening to get the person engaged
		Be patient and compassionate. Sometimes you might be able to recognise that your client is not in that space with you, that their mind is somewhere else. Accept it, instead of trying to bring them back to reality, because that helps them feel safe with you.
		Be adaptable and sensitive. If you ask a question and the client is not receptive at that time, leave it, then ask it another time. Bringing up a certain sensitive topic can create frustration on the person, so being able to recognise and be sensitive towards that is key
		Show an interest in the person. So, for example, if they’ve got a dog, ask about the dog and what is its name, what kind of dog is it. The next time you call, be mindful to ask, how’s e.g., Sherry doing
		Be reliable. For example, phone people when you say you’re going to phone, because people can be really disheartened if you do not
		Many social prescribers find it more conducive to building rapport when they hold very honest, practical conversations with clients. This helps clients have a clearer understanding of what is “on the table”, what they should expect and what options are available to them
**Identifying needs**	You want to identify your client’s needs correctly	Sometimes, the reason for the referral might be different than the actual need of the client. It is helpful to hear from the client’s side whether they agree with what the referral has brought to your attention.
		Sometimes, information relayed by the client may be unreliable. You may consider a visit in their home. Social prescribers report that seeing the person in their own environment reveals much more that any remote conversation with them. Some clues indicating unaddressed needs may be their appearance (clothes, keeping unkempt), food (quantity and quality available), the way they move is important, their environment (e.g., is there any hoarding?) There are strategies to check these things without being intrusive, for example, by asking for a cup of tea and checking the quality of milk or glancing inside the fridge when opened
		To check for the consistency of answers, ask a question at the beginning of the conversation and then ask again towards the end and see if they had any recall that they had already been asked and if they gave the same answer
	You find it difficult to identify what the need of your client is, what they really want	Some people may struggle to identify their need and priority. You may commonly hear your client say “I don’t know what I need. No one’s asked me this before. I have no clue where to start”. It might be helpful to invite them to tell their background story, what has been happening for the past six months. Something important will come up. Some social prescribers find a technique called motivational interviewing helpful: https://motivationalinterviewing.org/understanding-motivational-interviewing
		When the person is telling you their story, you will notice certain themes that they have brought up. It is helpful to summarise and ask the client whether they agree that these issues and needs are recurring and might be important to address
	Your client comes up with many needs and they/you do not know which to prioritise	You may want to go through a prioritisation process. Ask the person what the most important thing to them is right now, so that you can finetune their needs. You will find that usually by addressing a core need, a domino effect will be set in motion and other needs will automatically be addressed too. If there is time and capacity, once the priority need is addressed, you may want to consider others.
**Identifying/signposting to resources**	There are not relevant/appropriate support services/resources for your client locally	Be aware of what is going on in surrounding areas, so you can refer the client to resources and people in a different locality
	Your client has a specific interest/need and you are unsure if an activity/resource/organisation can help/is effective	Be aware that information posted online or on leaflets might not be up to date. Go in person to speak to the relevant people. If it is an activity or group, attend one session, as it might give you indication if it responds to your client’s needs. You may find something exciting and new yourself. Establishing links with people in the community will facilitate future referrals too.
		Look out and search for the evidence base. For example, if your client is interested in physical exercise classes, look for the literature that has been published in that area, so you can identify what has been proven to be effective. This will also reassure you that you are suggesting activities based on evidence of effectiveness. Remember to look for evidence on scientific reliable sources. For example, on https://scholar.google.com/, you can input the main words such as “exercise” and “dementia” to identify published literature in this area
	You have identified support, but the client has been placed on a long waiting list	You may want to offer bridging that gap in time, to keep the momentum going, so that you do not risk losing the client along the way. Try asking them if there is anything that you can help with whilst they are waiting. There might be some prep and preliminary work that you can help with (e.g., filling in forms)
	You have signposted the client to a service, but they have failed to get back to the client	You may want to contact the service on behalf of the client to gently chase it up. It may be also helpful, before you refer a client to an organisation, to check their own waiting lists. If these are too long, you might want to look for alternatives before signposting your client
**Engaging the client in social prescribing**	Your client is resistant to engage in the activity you offered	Some people say “I don’t want to do that”, as opposed to “I am afraid I can’t do it very well”. You may want to consider exploring barriers to engagement, for example, through motivational interviewing: https://motivationalinterviewing.org/understanding-motivational-interviewing. You may find that the client is experiencing barriers that do not enable engagement in the activity. Common barriers include mobility and transport, finance, and bereavement. If you know the barriers, you can work to address them. In some instances, e.g., if the client lacks confidence to make that first step in joining an activity, If you have capacity, you may want to consider accompanying the person during their first session
		It is common experience to have clients who have an attitude of “I’ve been there, tried that. I’ve done that like I’ve done that before”. If that is the case, you may want to explore the issue to build that motivation. Questions like “how long ago did you try? What’s different now? Would you be against trying that again?” can help you explore their preparedness. Suggestions like “Let’s have a little look at amending that, adapting it, adapting it slightly” can win their cooperation. Alternatives like “you can’t do XY you said, but could you do a B&C. What does that look like? Let’s have a look and try” can challenge their resistance.
		There have been instances where the client with dementia refused to do e.g., an activity, but their carer and family was adamant that they should do it. Consider always the voice of the client, as social prescribing is person-centred, unless the caregiver or family have power of attorney. In any case, avoid confrontation, and try to be as inclusive as you can of everyone in the decision-making process
		Have you considered whether the service and activity is culture-relevant to the client? In particular, if the client is from a different background, mainstream dementia services might not respond to their needs. For example, a person with dementia from an ethnic minority might revert back to a time where mixing with other cultures was not appropriate or common, so they might be resistant to engage in a dementia café where most attendees are not from their community. Consider demographic factors and gender roles as well. Social prescribers report that men from older generations may be hesitant to ask for help. Some people do not consider themselves to be carers. Ideally, you may want to address culture by asking your client about religion, ethnicity and gender identity. Just be aware that these may be sensitive topics. It is also important not to assume that all people from a specific community will want to attend culture-specific activities and groups, and prefer mixing with others
		Some people with dementia do not want to be involved in dementia groups and activities. May report not wanting to see what they will be like down the line. In line with the ethos of person-centredness, it is good practice to be open and prepared to signpost your client to non-dementia-specific resources as well
	The client has forgotten about previous calls/sessions or needs reminders about decisions you previously agreed upon	Starting and ending consultations with recap summaries might be helpful. You may it helpful to ask the client to write some things down and to place the paper in a specific place or sending emails to caregivers or relevant family members. Include a list of agreed actions. This will act as a reminder but also prompt action planning. To safeguard you, it may be also helpful to check with them ongoingly that you have their consent and to record in writing whether the client has declined anything
	You struggle to keep your client motivated to engage with you	Clearly state that the client has control in the present and make them feel empowered. You may want to explain that it is important to be proactive in the present, to look at what the options are, so that the person is in charge of making decisions. On the contrary, control is taken away from the person if things are left unaddressed and something has to be put in place as an emergency. Some strategies that work to make clients empowered are asking them how they would like you to communicate with them, giving them options (e.g., three links to activities), asking them to check which one they would like to engage with versus referring them directly
		It may help to take baby steps with your client. Lower your expectations and understand that the first goal that you might want to agree with them is that they will answer the phone when you make appointments. When you have that initial goal achieved, you can slowly build on.
		Try to give your client a vision with questions like “What would that give them? What would their life look like if they had done that? What would their family and friends see? What would they say about them? What would they observe if everything was different and how they wanted it to be? Or how would it benefit their relationship?
	You struggle to speak to the person as the carer is talking over them	Many social prescribers report that carers are often present in consultations and sometimes they take over. To make sure that the focus is on the person with dementia, set clear ground rules at the beginning, saying you will direct questions to the person and inviting the care to only fill in the gaps when needed.
	The carer is gatekeeping and resisting to let the client engage in activities	You may get the carer onboard by explaining that anything that you are doing is in the best interests of both the person and them. For example, that they are also going to benefit from the person with dementia being more socially active or taking part in community events. They might have extra respite time or social opportunities with other carers
	You are asked questions about areas that are out of your remit	Social prescribers have reported being frequently asked questions e.g., in the medical area. This might be due to a link with primary care and wearing an NHS badge. Other than explaining roles clearly, a quick strategy to escape these questions is to pretend to not know the answers
	You struggle emotionally when hearing stories from clients/caregivers	You may want to create your own space for reflection. Social prescribers tend to have packed schedules, to go through the hustle and bustle with no time to actually reflect on, e.g., what is actually going on in the client’s headspace, how that makes me feel, what support and infrastructure I need to be able to support the client better whilst safeguarding my own wellbeing as a worker
		Learn about resources for social prescribers’ emotional wellbeing. Some examples include mentoring schemes, supervision, team meetings, informal catch ups with colleagues, WhatsApp groups, peer support groups, drop-in sessions. These are all viable venues where to present cases to reflect on practice and to find support on those type of cases that might be difficult. It is important to have a system in place to support you emotionally, as social prescribers have often reported getting upset, in particular with difficult cases and when they work in isolation from home
**Discharge and continuity of care**	You want to check how your previous client is doing	Set up a catch-up appointment or call. If possible, ask to be included in the client’s annual dementia review (https://www.alzheimers.org.uk/get-support/help-with-dementia-care/gp-annual-review-person-dementia)
	At the time of discharge, you notice that the client has become over reliant on your support (e.g., they come up with a “new” need)	You may consider not closing the referral and offering the client to get in contact with the surgery or organisation again, if they need support in the future
		Set boundaries and be aware that leaving your phone number or email may send the wrong message. Instead, have people go through the switchboard team, if possible. They might help sometimes, saying you are not available, so clients learn you are not there for them always
		To avoid dependency, it may help to rotate, so that the same clients are not seen by the same social prescriber on subsequent referrals.
		Try to be really honest and upfront yet tactful, clarifying that you are not there for long term support, that you are not a mental health professional, counsellors, the befriending service. Your job is to help them to engage with the services that are specialised. Remember that although it might be difficult to say, you would be doing a disservice by keeping the client on long term.
		You may use motivational interviewing (https://motivationalinterviewing.org/understanding-motivational-interviewing) to help the client arrive at the conclusion that they do not need your support any longer
	You are anxious because you are discharging a client who lives independently and does not receive much support from family	You may want to create a diary with a plan for continuing support. Try to identify an organisation in the community that offer to visit people in their homes, spend some time with them, or take them out. Include contacts from this organisation in the client’s plan. Share with the organisation the plan that you have created with the client, client’s information and everything that you have done (with their consent). Let the organisation take over. Agree with the organisation that if the client needs further social prescribing, they could get in touch with you directly and you could just step back in. This strategy should reduce your and your client’s anxiety about discharge
	You are anxious that the client becomes overdependent on your support	Experience suggests that normally there will be an initial introductory phase and then there will be a spike of activity. And then it will inevitably plateau and eventually subside, as clients will start to adjust to the situation, or their circumstances will change
**Ongoing professional development**	You want to keep up to date with dementia	If you have not received basic dementia training, it may be essential to sign up for a course. Dementias present in such a wide range of symptoms and behaviours that it is essential for social prescribers to have awareness of the different types of dementia and the way they might affects clients and families, as well as your own provision of support. Some elements of trainings that social prescribers have found helpful are what dementia looks like, how it is potentially going to progress depending on type, what to do if a person with dementia goes missing (https://www.scotland.police.uk/what-s-happening/missing-persons/the-herbert-protocol/), and strategies to work around memory impairment (e.g., have memory books, have things photographed, write key things or reminders on post it notes, try not to move things around in the home)
		Try to take refresher courses at least once a year to keep up to speed with new evidence and practice. Some social prescribers also wish to learn about the dementia pathway, i.e., what is the pathway from the client seeing a GP? What happens after a diagnosis? What are the primary, secondary care and statutory services available and how can a person access them? What are the legal arrangements and the practical and finance support people can arrange? If training is not mandated or offered within your job role, you may want to look for free and for pay courses and resources online
		Consider national policies and dementia strategies have been released in your locality, as these provide key frameworks and information within which you can operate. E.g., https://www.alzheimers.org.uk/about-us/policy-and-influencing/national-policies. Note that these may be country-dependent UK and not necessarily applicable to other countries
	You want to learn and experience first-hand how to support a client with dementia	If possible, shadow someone within your team who has more experience than you
	You want to progress your career and become a specialist in dementia care	Social prescribers have reported that in the current status of services, it is difficult to embed specialism in social prescribing, as social prescribers are required to support a diverse range of populations. However, if you have a special interest in dementia and want to invest in professional development, you may want to consider courses and programmes that give you accreditation (diplomas, certificates) to boost your CV and open up new horizons

### Pre-contact groundwork and preparation

Social prescribers discussed some of the challenges, and possible solutions, of preparing to support a client with dementia. A typical issue was the lack of details and background information about clients on referrals:

*“In the referral, it tells me who’s made the referral, their contact details and usually a little bit about the situation. It is very dry, doesn’t tell me where the person is accepting of it, whether he wants to talk”.*

*SP11*

Lack of in-depth information was exemplary of poor liaison and communication within integrated care systems (ICSs), in particular between primary and secondary health services:

*“I think one thing that I do struggle when it comes to referral for a client with dementia is it often reflect how secondary care and primary care aren’t as well linked as they should. So, for example, you might find no information around the actual input that the memory clinical service has had on that client. And you could end up duplicating work”.*

*SP13*

Therefore, some further fact finding around client’s circumstances was required. For social prescribers working within in primary care, this happened through accessing clients’ medical records:

*“Before I meet them, I examine people’s medical records to get a much clearer idea of what their diagnosis is, and I know whether they’ve got consent for relatives to speak for them”.*

*SP18*

For social prescribers working in the third sector, however, this was not possible, causing a delay in information gathering until it could be relayed by the client (or family) directly. When a referral for a client with dementia was received, the social prescriber did not always feel competent and confident enough to support that client. Some social prescribers reported that their teams had designated a dementia expert social prescriber who could confidently take on the case:

*“We consider expertise within our team. We’ll look at the referral and if dementia is mentioned, we try to pass the referral to the one who’s better suited for that client”.*

*SP02*

There were also strategies in place to handle workflow when services are stretched. An example was implementation of traffic light systems based on matter of urgency:

*“We prioritise referrals in a matter of urgency. So, urgency would be a person with dementia who is homeless, has no food, no gas and electric. Then, we have high priority, which may be a person with dementia who hasn’t got attendance. We tend to put them on high priority just because how long the process takes to claim. And then we have moderate, which would be people that are interested in social groups or activities”.*

*SP09*

Once a social prescriber was assigned, the case was opened based on matter of urgency, and first contact was made.

### First contact

Social prescribers agreed that barriers to communication were common when establishing contact with a client with dementia. First contact often occurred over the phone to save resources, something which could be problematic for social prescribers:

“I think this is this is a big problem, but we are kind of discouraged from face-to-face contact for home visits because it costs time and expenses for travel”.

SP03

In the context of remote interactions, the client with dementia might find it challenging to communicate effectively. They might be confused, reluctant, or unable to interact. A common challenge was client’s resistance to accept support during that first call:

*“When I make the phone calls it’s sometimes very dry at first and it’s going nowhere. They’d say—Oh, thank you very much, but I’m alright. I don’t need anything”.*

*SP11*

There was a risk that social prescribers may not identify resistance for what it was. They might mistake the decline to accept support for a lack of current needs, and close the referral too early, leaving the clients’ needs unidentified and unaddressed:

*“It happened in the past that I called somebody, and they tell me I don’t need anything. So, I closed the referral, but then later I received a call from their sister, saying—yeah, let’s do this. I’ve got power of attorney—But those people that don’t have the sister, they may not have that second chance”.*

*SP03*

Some social prescribers described some strategies to deal with communication difficulties effectively. The first was to initiate communication in paper format:

*“We would send a letter first, so they know who we are, why we’re ringing and when we intend to ring. If they’ve got something in writing, they can pop that in the calendar. If not, we would hope that a family member might pick that letter”.*

*SP11*

Another one was checking information around mental capacity and putting mechanisms in place to support the client:

*“If I see there could be issues with mental capacity, I close the case, let the GP know and I push forward for a mental capacity assessment. This would enable us to instigate power of attorney or appoint a mental capacity advocate for the client”.*

*SP10*

If it was documented that the person was likely to have mental capacity to consent to contact but had difficulty communicating this, a good option could be to pay one or more home visit(s):

*“I had a client once who was not engaging on the phone. So, I went and knocked on the door and nothing. So, I went a second, third time and eventually we got the person sat on that day. And she was totally fine talking”.*

*SP01*

Another effective tool to support understanding was using clear and accessible language when explaining the role and potential of social prescribing:

*“Describing your role rather than saying the name of the role can be helpful because for a lot of people when you say social prescribing the first thought is social worker. So, they get a little bit worried”.*

*SP13*

Trying to establish communication with a family member could also prove very helpful, but it was important to keep in mind that ultimately social prescribing was person-centred, and clients needed to want to cooperate:

*“Unless someone has got power of attorney and the person is deemed not to have any capacity, I have to listen to what the client is telling me”.*

*SP18*

When all strategies proved ineffective, the social prescribers reported it was important to acknowledge that the client might simply not be ready to accept support:

*“Then I just close the referral. But I let them know that they can always come back to us if they change their mind or that we can schedule a six-month callback as well if they want it. Sometimes people just want to know there’s that someone if they need it”.*

*SP12*

### Developing motivation and rapport

Social prescribers reported that motivation and trust were key to building rapport with clients. It was important to acknowledge some strategies to promote these. Social prescribers felt that motivational interviewing could sometimes help to empower clients with dementia in the decision-making process:

*“It’s about exploring in a motivational way: how long ago did you try? What’s different now? Would you be against trying that again? Let’s have a little look at amending what you tried in the past. Let’s have a look and try”.*

*SP15*

It could also help to instil a sense of agency and control to further motivate clients:

*“I often find myself saying—we can either sit and do nothing, or we can start looking at the options. And that puts the person in much more control”.*

*SP11*

The social prescribers reported that ultimately it was about trying to clients a vision of the changes that engaging in the process could bring in their lives:

*“What would that give them? What would their life look like? What would the difference be for their families”?*

*SP15*

Sometimes, involving family members in these discussions could help, particularly if the social prescribers were able to convey the message that they could also benefit from the person they cared for making decisions on goals and processes:

*“Remember who the client is. Try to direct only some questions to those who can fill in the gap. Explain to families that they’re also going to benefit from the person deciding what it is that they want out of it”.*

*SP20*

### Identifying needs

When identifying clients’ needs, the social prescribers reported that notes on the original referral may reflect GPs’ views, and there may be discrepancies with those of clients. Needs may also have changed over time. The social prescribers suggested it was helpful to double check with clients the reasons for referral and current needs:

*“Even when I read the notes on the GP, I like to let the client lead the conversation because what they said during that consultation with the doctor might change. They might need something else”.*

*SP17*

It was key to acknowledge that when identifying needs culture sensitivity was key on the part of the social prescriber. Because of language barriers, cultural factors and background, clients might be reluctant to open up, or unable to identify and voice their needs:

*“Some people do not see themselves as carers in certain cultures. They’d say—this is what you do for your family. Or in a lot of languages there isn’t a word for dementia, so it’s not necessarily understood what it is”.*

*SP11*

It was therefore important for social prescribers to think about how to effectively communicate when supporting these clients, helping them to start conversations around and voice their needs. For example, to facilitate communication with these clients, interpreters may help, as well as involving more language-proficient family members. Another common barrier that social prescribers experienced in identifying clients’ needs was sensitivity of certain topics. A social prescribers reported finding difficulties to initiate conversations around sensitive areas:

*“Sometimes I struggle to bring up the conversation myself, so most times I expect the client to kind of bring it up because you don’t want to cause that kind of conflict from the beginning.”*

*SP13*

It was important to challenge personal assumptions and understand that because a client belonged to a certain ethnicity or have a gender identity, it does not mean they wanted to take part in activities designed for or specific to that group:

*“Just from experience 50% of clients are willing and happy to engage with community service that are part of their own culture. But 50% of them just say no”.*

*SP13*

### Identifying and signposting to resources

When it comes to finding support for clients with dementia, many social prescribers reported that they started with an online search to look for evidence of effectiveness on the type of support that the client needed:

*“I look online for the evidence base in the literature to have the knowledge to say for example, these are the top five exercises that are proven to be good for good brain health”.*

*SP04*

They then identified who in the community offered that activity. This could also be done online. However, not all services were advertising online, and the information provided might not be up to date, as described by a social prescriber:

*“While the website stated that the service could not offer transport, they could find ways and make budget lines work so that they could support an individual in exceptional circumstances. I only learned that by having an in-depth conversation with the people in the organisation”.*

*SP22*

By going in person, social prescribers often found out about services that were not advertised/updated on websites:

*“You won’t find out unless you go and look at places in person to check for yourself”.*

*SP05*

Another helpful strategy to learn about resources and opportunities in the community was signing up to social prescribers’ associations and events:

*“The National Academy of Social Prescribers and the National Association of Link Workers have been doing monthly newsletters with information on initiatives and activities. So, it is good to sign up. Regional conferences are also a good way to actually get all in the same room and be able to network and make those connections”.*

*SP22*

Finally, social prescribers advocated staying curious and thinking broadly and creatively. They should look out to wider provision that they might not be as familiar with, as this may open up new opportunities for the client (and themselves).

### Engaging the client in social prescribing

Social prescribers reported that they may struggle to maintain engagement of their clients with dementia throughout the support process. They often found that seeing clients in person, as opposed to holding sessions over the phone, facilitated engagement. A relaxing and informal atmosphere was key, and this could be set from the start with some small talk:

*“I start by asking questions like—How was your day? Talk to me about your day—Humour also works well. Or if they’ve got a dog, I’d ask about its name, and the next time I’ll remember the name. If you show a little bit of humanity and interest, clients open up”.*

*SP21*

Even when securing initial engagement, maintenance over time may prove problematic, because of memory problems, apathy or lack of motivation, and changes of circumstances. The sudden changes that dementia entails could be addressed by gathering ongoing consent and using summary/reminders in sessions:

*“There have been times where I have to remind clients who I am really exactly, what conversations we’ve had and revisit those things over and over. So, I start every consultation with a summary of the previous session and end it with recap notes. I ask them to write these and suggest putting them somewhere visible or where they can remember. I also check with them ongoingly that they know that they are with you and that they agree to your support in this journey”.*

*SP10*

Clients with dementia may experience “bad days” and social prescribers should be aware that strategies to engage them might not work on those days. They should therefore be flexible and open to revisit actions later on. They also reported the importance to keep in mind that to successfully engage clients with dementia, social prescribers needed to be realistic about goals they set:

*“Sometimes, it is baby steps, really. The first goal that we might agree is that they’ll answer the phone to when we make an appointment, and that really is a lot sometimes”.*

*SP15*

Acknowledging that sometimes lack of engagement may be caused by other factors that need exploring sensitively and over time was also important:

*“I had somebody who wanted to be linked with activities and exercises, but when we started talking, they wouldn’t really engage. By digging, I identified mobility issues, bereavement and financial issues that created one big barrier to engagement. And once we started addressing them, the client started to engage”.*

*SP03*

### Discharge and continuity of care

The social prescribers reported that discontinuing support with clients with dementia presented with particular challenges. For example, issues of overdependency could develop:

*“Suddenly the social prescriber comes in, they listen, they try to put things in place. They’ve just been given somebody who’s there for them. So, sometimes those people do hang on”.*

*SP03*

For clients who lived alone and who had limited support from family or friends, separation could be especially difficult. In these cases, when terminating rapport, a gradual process to prepare clients may help. It started by establishing honest conversations with clients from the inception of support, in which the social prescriber set realistic expectations and professional boundaries:

*“It’s about being really honest and upfront with the individual that we’re not here for long term support. We’re not mental health professionals, counsellors, or the befriending service. My job is to help them in to engage with the services that are specialised and other professionals in that field, so I’ll be doing them a disservice if I just keep them on long term. That’s really harsh to say to somebody who is isolated, but really important”.*

*SP15*

When the time of discharge was nearing, the social prescriber invited the client to agree a post-support plan. An example was to:

*“Find a local organisation who offers to go to people’s homes, spend some time with them, take them out. And collaborate together. Send them the resources you created for the client, the client’s information with their consent, and details of everything that you did. And then the organisation can take over”.*

*SP03*

Continuing support plans ensured that in case of future need, the client could easily be referred back to social prescribing services, avoiding that they “fell into the cracks of the system”. It was important to remind clients that they could get referred to social prescribing multiple times if they needed it. Leaving contact details and information further reassured them that support was available. Social prescribers also felt that giving out personal numbers should be avoided, as clients may feel free to call social prescribers unannounced. Strategies employed to discourage this included for example that:

*“Everybody has to come through the switchboard Team, who are instructed to say—Oh, sorry, Mrs. X, you’re not currently working with Y. Is there anything I can help you with? I can open a case up for you with one of our other team. We also tend to rotate to just avoid working with the same clients over and over”.*

*SP20*

All of these strategies around discharge were in place not only to benefit the clients, but also to ensure the safeguarding, quality of work and emotional wellbeing of social prescribers.

### Ongoing professional development

Ongoing professional development was an overarching theme applicable to all stages of support. Social prescribers received mandatory training, but not around dementia:

*“The National Association of Social prescribers is the only mandatory training at the moment that social prescribers have to do once in post. It is made up of 10 modules that you have to complete. But there’s nothing about dementia in it”.*

*SP02*

Social prescribers could get dementia training only if and when they chose to. While professional development should be ongoing and proactive, given the diversity of clients and referrals they received, social prescribers reported that they sought dementia training reactively, rather than proactively, typically when receiving a referral for a client with dementia. The problem was that not all clients with dementia had received a formal diagnosis yet, or dementia might not be mentioned in the referral. Without adequate training, social prescribers found themselves second guessing whether their client had dementia or not:

*“It’s very hard, especially when people reach a certain stage of their life to know what normal forgetfulness or normal changes in behaviour are that come with age, and what is actually a dementia concern. We’re not particularly well trained to spot that”.*

*SP05*

A dementia diagnosis was a key step to personalised care approaches, so it was important for social prescribers to consider ways to raise their dementia awareness. One suggested strategy was undertaking dementia training around:

*“The basics, about how you speak to people who’ve got dementia and how it affects people, just to take a bit of that mystery out of it because not all link workers have had experience of dementia in their career or families”.*

*SP08*

It was suggested that part of the training could focus on different stages of the condition, so that social prescribers were able to grasp the range of different experiences and challenges that different clients may present with. This would include being able to approach clients sensitively based on the degree of acceptance and preparedness they had towards “dementia support”:

*“Before my training, I didn’t know that some people don’t want to hear the word dementia. I would take on a dementia referral, call the client and I would just say—hi, I’m calling regarding the dementia of Mr X”.*

*SP17*

It was also helpful for social prescribers to know what the care pathway was once clients get a diagnosis of dementia:

*“It would be important to learn what is the pathway from a person seeing to a GP? How do they reach the conclusion of the diagnosis? And then from the diagnosis, what happens?”*

*SP02*

## Discussion

This study explored the unique challenges, and potential strategies to address these, when working as a social prescriber for people with dementia.

One of the biggest challenges identified by participants was the provision of social prescribing via telephone consultation. We were unable to identify data on the proportion of social prescribing across the country that is delivered remotely, but only a handful of social prescribers reported being able to offer face-to-face appointments. Remote appointments are instead the norm and mostly used for resource and cost saving. However, evidence from our study, and the wider literature, suggests that in dementia care face-to-face support is best to build trust and rapport, facilitate communication [30] and combat rejection of care [31]. In line with current evidence around communication technologies and their risk of generating digital exclusion of the most vulnerable [32], despite the guiding question: “*what matters to me most*” [33], when communications occur remotely, the social prescribers reported that it can cause confusion, reluctance, or inability on the part of the client to interact effectively. This runs the risk for social prescribing to become unequitable and disenfranchising for (some) clients with dementia, especially those with advanced dementia [34] or sensory impairments [35].

Because of this, social prescribers reported often find it challenging to help clients identify and prioritise needs, priorities and end goals. This is further compounded by a lack of knowledge/clarity about role and remit of social prescribing on the part of the clients, who often ask questions beyond the expertise of social prescribers e.g., in the medical area. Once clients’ needs and goals have been identified, social prescribers source community-based assets that address these. The service expectation on social prescribers to be able to identify any kind of service in the community can also be daunting, making them feel like “Jack of all trades and master of none”.

Another challenge that participants identified in providing effective support to people with dementia lies in their generalist (i.e., non-specialist) role and remit. Social prescribing addresses all non-medical needs, and it has a whole-population approach [7]. This means that a social prescriber, by service requirements, is expected to support at any time a range of clients with different needs/conditions, including those with dementia. Study participants reported however, that there is currently no mandatory specialist training in dementia because of lack of resources, as also identified in the literature [36]. Having training in dementia as optional for social prescribers means that getting training depends only on personal commitment and enthusiasm, and availability of resources. As a result, study participants reported that training in dementia and the distinct barriers that it presents, particularly around motivational issues [37], resistance to care [38] and separation anxiety [39], tends to occur only reactively, when a social prescriber is referred a client with dementia.

Another great challenge that study participants identified is that despite the ethos of "integration” in the model of integrated care systems (ICSs) [40], service provision remains quite fragmented, in line with findings from previous literature evaluating dementia care and support [41]. Issues around a lack of communication between services was reported by several participants, who were relayed little information on the type of service, support, and care that some of their clients had been previously provided. Participants also reported receiving referrals from memory clinics (part of secondary care services) with very few details about their clients’ background, needs and reasons for referral. Poor communication and integration between primary and secondary care has also been found in the literature [42], and runs the risk of replicating treatment (e.g., referring a client to a memory clinic, which they may have done already), not moving clients along the pathway, and in turn wasting NHS resources.

### Implications for practice

There are some important implications to be derived from study findings to improve current provision of social prescribing. Exploring and identifying individual needs, and co-designing a support plan, with clients with dementia is a necessary condition to make social prescribing truly person-centred. This requires a degree of flexibility in services to allow face to face consultations and in-person home visits, as participants reported that current means of communications with clients (i.e., via telephone) are not optimal. While face-to-face visits require more financial, time and staff resources, they could pay back because they might facilitate rapport, clients’ engagement, and effectiveness of support [39]. This potential should be considered with caution at present, however, as only limited evidence exists around cost benefits of social prescribing [43].

Preparing and training social prescribers in primary care whilst addressing lack of resources in the system could be addressed through encouraging specialism in dementia, i.e., designating a “dementia-specialist” in social prescribing teams. Such experts can support the implementation of learning, developing tools, and leading on delivery of care, whilst supporting culture change and the professional development, and mentoring of more generalist colleagues. Having access to and working within teams with designated experts in dementia care (e.g., Admiral nurse) has been found to reassure and empower staff to make changes and improve dementia care in other settings, including acute hospitals [44]. Our study found that that social prescribing teams already informally share their clients based on experience and expertise, so implementing this change could be a feasible step.

Effective provision of social prescribing can also be promoted through collaborating with third sector organisations. Voluntary, community, faith and social enterprise organisations contribute to ICSs unique local expertise in working with people in the communities where they live [40]. They possess the know-how and a wealth of expertise they can share with social prescribers. Many work at the intersection of dementia and other characteristics, and can contribute resources that can equip providers with the skills and knowledge to provide highly tailored care to the diverse community of people with dementia (e.g., https://lgbt.foundation/help/pride-in-practice/). The opportunity here is to develop a community of practice with multi-disciplinary expertise to support communities who traditionally “*fall within the cracks*” of healthcare, including ethnically, culturally, and sexually diverse populations [45]. Meeting the distinct needs of underserved communities of people with dementia would promote an ethos of universal equitable care [46].

In terms of continuity of care services, it is also important for Integrated Care Systems (ICSs) to invest on continuing improvement of liaison, collaboration, and communication with secondary health services [47]. Existing literature on the general older population suggests that the potential of social prescribing to support clients’ health and wellbeing can only be realised through strategic alignment of local level implementation [48]. Our study findings emphasise that this is even more crucial in social prescribing with clients with dementia, due to unique challenges such as communication barriers, clients’ motivational issues and overdependency, to the importance of continuation in support and to the expectation on social prescribers to support their clients effectively by tapping into levels of competence which are beyond their remit [49].

Some initiatives with other clients’ groups have shown great potential providing a cohesive service provision model to improve effectiveness and continuity of care within and beyond social prescribing [50]. For example, a study found that social prescribers valued having access to an occupational therapist when supporting people with Motor Neuron Disease, due to the complexity of the condition and the barriers they faced [13]. Reablement services have successfully implemented this type of service model, and the Royal College of Psychiatrists has advocated the need for inclusion of occupational therapist’s expertise to address clients’ needs that go beyond the level of competence and training of social prescribers [50]. While it must be noted that all the aforementioned evidence is specific to the UK policy, systems and practice, and as such, not necessarily generalisable to other contexts/countries, this evidence presents important learning points. Effective provision for clients with dementia can be pursued through extensive collaboration with other statutory and community partners, structured processes for documentation, seamless exchange of information, and allocated case managers coordinating input from multiple agencies [51].

### Strengths and limitations

This study was characterised by certain strengths. It presents evidence about challenges and offers practical strategies (Table 3) that can be used by a range of social prescribers supporting people with dementia in different roles, and which may be transferrable to other population groups. The evidence provided in this study may also be helpful to inform future practice. The study was able to recruit a diverse sample of social prescribers working within primary care and voluntary organisations, and in different roles in social prescribing, which captured a range of different challenges and solutions based on variation of service provision nationally, demographics of the area, availability of resources and demand and offer.

In terms of limitations, the sample only included participants who self-identified as social prescribers. Because of this, some social prescribers who are delivering social prescribing activities but may not have formally received accreditation as “social prescribers” may have refrained from contacting us. In addition, the study did not involve people with dementia and their families. It is important therefore, for future studies, to involve a wider range of professionals who deliver social prescribing, as well as other stakeholders.

## Conclusion

This study identified unique barriers that social prescribers face when working with clients with dementia, particularly around communication, motivation, engagement and overdependency. Further, it identified person and system-level strategies that can be used to address these challenges and improve delivery and effectiveness of services for this population group, including expanding opportunities for dementia training, delivering support in person, including social prescribing in annual dementia reviews, and increasing integration of social prescribing in the context of ICSs and collaborations between health care services and third sector. Further research is needed to investigate experiences from the perspective of specialist, as opposed to generic social prescribers and service users themselves, both people with dementia and their family carers. The cost effectiveness of such an intervention in dementia care also remains an evidence gap.

## Supporting information

S1 File (DOCX)
