# Supplementary material for: What are the challenges that social prescribers face when supporting people within dementia and how can these be addressed? A qualitative study
Source: PLoS One. 2025 Jan 17;20(1):e0317749. doi: 10.1371/journal.pone.0317749 (PMC11741600; doi:10.1371/journal.pone.0317749)
Supplement: S1 File — (DOCX) [file pone.0317749.s001.docx]

**SP01**

[Start of recorded material at 00:00:00]

Respondent: ... frozen but –

Interviewer: It looks – yes, your image is frozen at the moment. I can hear you.

Respondent: Yes, I can hear you –

Interviewer: OK, that’s good. It’s not a problem. Don’t worry. The important bit is the audio. So, let me just grab my questions. So yes, the first question I wanted to ask you, [unintelligible 00:00:26] is, how does social prescribing work within your specific service, as in what’s the route for clients to access the service?

Respondent: Just off that question, you were talking about social prescribing link workers, NHS?

Interviewer: Yes. Yes.

Respondent: Yes, yes, that’s fine, because since social prescribing link worker 2019 started with NHS, because I sit – so, in [anonymised], the model of social prescribing is a bit different from other cities, and the difference is that social prescribers mostly sit in the voluntary organisation, and they are jointly contracted with local primary care networks to provide this support to their patients.

So the PCN I am with, instead of going on their own and recruiting a social prescriber, they went with the model that, because for the last 20 years they were working with us, most of the surgeries, and so they came to us and said, “Look, this is what you do, and we think it’s good because you’re already working with us. Would you like to have a joint contract, where you provide us the service, and we provide you the clients, patients? Because we think it’s a good service you provide, and we don’t want this social prescriber to sit on its own somewhere. It can be part of your community team.”

And another thing which is good for us is, because we’re the biggest surgery in the primary care network, it’s co-located with us, so we’re in the same building, actually, which works really good for us. So I, as a social prescriber, [unintelligible 00:02:15] social prescribing link worker provide support and work to our primary care network, and I do receive referrals through System One, or any other system which GPs use, or email, which is NHS secure email, as I have a laptop given by the surgery, by the PCN, on which I access the System One, and I get the referrals through.

If any health professional who is on that system, and work with this boundary of where I work, which is called [anonymised], they can refer to me via our task through the system. So, it’s like internal email, so it’s the quickest way to do it. So, what I’ll do is, every day I go on system, I check the tasks today, and I see someone, they speak to person, and actually I realise, actually, this person could do with extra help. Let’s talk to them, get the consent, and then they’ll task me, say a one-liner, because health professionals are very busy, so we don’t want them to write notes.

So, they send me a line saying, as in a case of dementia saying, “Patient has onset of dementia, family is struggling, please have a chat.” That’s what they will do. Then I will take my time, ring, make sure we get all the information, and then from there, we’ll take it to the next step.

Interviewer: I see. I see. So essentially, the clients are – by the way, do you call them clients or patients? What’s your preference?

Respondent: In my work, everyday work, I call them clients.

Interviewer: OK, I’ll go with clients.

Respondent: Obviously with the surgery, when we talk to surgery or health professionals, we say patients, because basically, because I sit in a community organisation, it’s the same person. Could be client or the patient, same person.

Interviewer: Yes, yes, yes, yes. OK, so essentially, just to recap, it’s the GP that kind of refers the person to you, and you take over from them because obviously they’re already very busy, as you said. So, from that point on, it’s actually you that liaise with the person and support them throughout, so you take over from the referral point onward, right?

Respondent: Anything non-medical is taken over by me, from there. Obviously if it’s to go back and to look at some medication, or something else comes up while working with the patient and the family member, I would send the same task back. It’s like an internal messaging, so I’ll send the task back to say, “You sent this referral. While working, this happened. Can you please look into, because that’s a medical ... ?” Anything non-medical, from housing, to losing weight, to getting them into a dementia services, I will do that.

Interviewer: I see. That’s very clear. Thank you, [anonymised]. And I wanted to ask you, it seems that you’ve already responded to this question, but do you think that the process of accessing social prescribing within your service is working well, and, if there’s anything that can be done to improve this process, so accessing from the side of the patients?

Respondent: I think, at the moment, it’s working very well, because I’ve [unintelligible 00:05:45] with the referrals, and anything [unintelligible 00:05:48] more referrals.

Interviewer: Yes.

Respondent: The only thing is that, at the moment, I’m the only one fulltime link worker for the whole PCN.

Interviewer: OK. Do you feel that you’re a bit overburdened, or what’s your workload like?

Respondent: My workload at the moment is between 40 and 50 new referrals a month, plus complex cases which are ongoing. And which means I can’t do fully everything, because it’s one person, as we’ve been asking to PCN, say, “What we need is another worker,” so we can halve the burden on one worker, so other things we can do is, like, this work helps us to understand what needs of community are and how we can meet these needs through different groups, through different trainings, and whatever we need to be at work with.

But, as one person, you’re just dealing with the referrals, and signposting [unintelligible 00:06:55] that sure all the time. You don’t have extra time to actually put something in practice, which I still do, because I have [unintelligible 00:07:05] which I sit in community organisation called [anonymised], and it’s in my email, so which means I can work [anonymised] and create something. I don’t have, personally, time to do it, as in link worker contract, whilst every 10,000 patients should have one link worker, which means, at the moment, we should have three, because we have 30,000 patients. So, that’s more resource battle.

Interviewer: I see, so that’s the point for improvement. I was wondering, given how strained you are at the moment, are you basically only mostly signposting resources and opportunities to clients, or are you actually engaging with them in these activities and opportunities?

Respondent: At the moment, I can’t engage in the – like, go and engage, so most of the time, I speak to them. In certain cases where activity, if I see that without my home visit, nothing will do or nothing will move, I will do that. But that, as you can see, a phone call can take 15 minutes, a home visit will take two hours, so I have to prioritise with the time. But that’s why we are constantly asking that we need more, and trying to do as much as we can. But even with the signposting, the good thing here is that I have backup from community organisation. So, if I get someone in my community organisation, I have someone who actually leads the dementia work.

So, I speak to family, I speak to person, and then I refer them into our work, so that is my colleague, who sits next door. So, I go and chat to them, make a referral, and then they take it from there. So, I use that resource at the moment, but it could be, if I have more resource, I could do better job in that by making sure everything is ready, and then they go to next step.

Interviewer: I understand.

Respondent: Now, I just have to get them there.

Interviewer: Yes, sure, OK. Let’s think about some of the clients that you have supported, or you are supporting. What kind of social needs do these clients living with dementia usually have? And, based on these social needs, what kind of activities do you usually signpost them to?

Respondent: So the social needs, basically, it’s the loneliness comes in, and again, because it’s loneliness, and time spent with family, even family is with them, what to do, how to entertain, how to do something which they like, which they will – how to stimulate their brain a little bit so they’re happy, they’re not just sit down and look at their face, or have a cup of tea. So, for that reason, say today, we had two hours, it’s called [anonymised]. So, where the people with dementia and their carers, they come in, and they do a lot of different activities, which is from raffle, to playing different games, and music.

So, that’s what happened, and my colleague, she leads on that, and then we have volunteers who, at one time, they were carers, or for some reason, so there we have volunteers. We have few staff members, because of the area we are in, it’s multilingual, so to cater for people who speak Urdu, Punjabi, or different – Somali language, and Arabic, so we have a few staff there to assist in that. Before, they wouldn’t had people coming, but now we have more Asian, we have Arabic speaking people coming in, because they can see that we can support them.

Interviewer: Yes, OK, so these are basically, you said volunteers, who are from those specific communities, and they can speak the language so that they can help people.

Respondent: Yes, so we have volunteers, and a few staff because we can’t rely all the time on the volunteers. But we have volunteers who speak Somali. We didn’t have a volunteer who speaks Urdu, Punjabi at the moment, so we have staff who speak the language, so that she’s going in, so that’s how we balance.

Interviewer: Yes, sure. And it seems like, as you said, more people from these traditionally underserved communities are joining because you’re able to provide that service, so that’s great to hear.

Respondent: Yes, and because they can see that the needs are met, so it’s because in communities, underserved communities, it’s a word of mouth trust, so it goes out, “Yes, it’s good. So and so in there. [anonymised]” it’s addressed to the organisation itself, and then they say, “so and so is there, who speaks ... ” So, more people are coming forward.

Interviewer: Yes, it’s a positive domino effect kind of thing, isn’t it?

Respondent: Yes.

Interviewer: Yes, I get that. So, you mentioned the dementia café. Can I ask you who that dementia café is organised by? Is it yourselves? Is it something that was already going on in the community?

Respondent: No, it’s actually a project which was city wide, but on this level, on our turf, we are doing it. So we get the – as a whole, we get the money from [anonymised] City Council to do different well-being activities, as part of one of that, and I was actually thinking, because I’m a link worker who’s actually the first point of contact most of the time, but then I’ll ask my colleague, [anonymised], who I couldn’t see today, but I’ll actually, if you’re happy to talk to her, she’s the one who actually runs those activities.

Interviewer: Oh, yes.

Respondent: That’s the second part. If you want to talk to her, I can ask if she has time.

Interviewer: That would be really helpful, actually.

Respondent: Because [anonymised] has, like, 40 years of experience, I would say, on this, and she’s from a different project. But this, she is very thorough in these things. Not only these two cafés, she does – we have an allotment where she does different things for people with dementia. She’s recently working with university [anonymised] to create music, and they actually have this – it’s not a bear, it’s a sloth type, bear type thing, where it has heartbeat, so people can put them on chest, and they can feel the heartbeat, and if you rub the hands, their favourite music can be in that. And if you slip – just rub the hand, music change, all that. So, they are doing different research, and they are using different methods in those cafés. And, they are well-attended, the cafés. I can say they’re full.

Interviewer: Nice. Yes, if you could please ask your colleague if she’s willing to have a chat with me, that would be really helpful. I’d appreciate that, thank you.

Respondent: No problem.

Interviewer: Thank you so much. OK, so let me ask you, you also seem to have quite extensive experience in this area, so I wanted to ask you how would a social prescribing link worker, with maybe less experience than you, and a network in the community, how would they learn about the activities that are available out there for people living with dementia?

Respondent: Say, on the [anonymised] level, I would ask anybody who is doing this. It’s the – because as I said, [anonymised] model is a bit different than – say, we have just next door an area called [anonymised]. The [anonymised] model is that the link workers are sitting in NHS building, the hospital, totally different. In [anonymised], as I said, most of us, we sit in the community organisations, the old – and then primary care networks now became primary care networks, so this is another thing to follow. So, most of the primary care networks were with community organisations, and the link workers are sitting in those. So, that’s how it’s covered.

So, any link worker who comes in new, one of the things which the organisations who are doing this before, so what they will do is, they will send me emails saying, “We have somebody new in post. Would you like to have a word?” Just have initial chat, because they know. So, we’ll send enough other emails saying, “We are doing this, yes, but have a chat.” Other thing I will say, go online, there’s a chapel directory in which all organisations put what they do. So, you can, if you put the word, “Dementia,” it will give you the list.

Other hand, you have to be a bit proactive, send the email around, check who is – we have about 15 different community organisations, I would say, in [anonymised]. Send the email out, say, “What would you do?” Or go on their website and check. That whole website is now fully updated, you can check everything what we do, when we do, how we do.

On the other hand, for me, my activities here in this area, I actually be into everything [unintelligible 00:16:58] the time, even dementia café, I went there, so I can tell someone what actually happens there. We have full programme of a weekly programme, different walks that we think – some of the, actually, groups I started, and some of the groups, I went over the years, I kept going, to know what’s happening. So, if I signpost someone, I can say, “Look, this is how it works. This is what you need to do.” And if I can’t go, say if it’s someone new, I will make sure, so if I have a referral, the families, where I will say, “Look, we provide this, and they’re happy to go into that.” I’ll email my colleague for the dementia café, saying, “So and so would like to come.” If she has time, she’ll get in touch anyway. If not, at least she will know they’re coming.

Interviewer: It sounds as if, [anonymised], from what you’re saying, the model within [anonymised] is very much rooted in the community, which seems to be very, very helpful because you guys are within that community, and know in and out what’s going on, as opposed to maybe other models. So, it would be really interesting for me to have a chat with somebody say from [anonymised], because you say they have a very different model, whereby the people are actually within the NHS. So, I’d be curious to know whether it’s a bit more difficult for their social prescribing link workers to learn, and know what’s going on in the community. That would be a really interesting comparison.

Respondent: Yes.

Interviewer: OK, let me ask a bit about skills that are required from social prescribing link workers to work effectively with this specific population. So, do you think that, generally speaking, people working in this area, think about your colleagues, for example, do you think they are confident at understanding the social needs of people living with dementia, and also those of the carers?

Respondent: If I start with the person who’s actually leading the project, obviously, yes. And not only that, she’s actually been to so many – she actually runs the training for others to understand, so dementia-friendly training, gold, bronze and silver training, she does, because she’s been trained as a trainer.

Interviewer: I see, and the training is for other social prescribing link workers, right?

Respondent: Not only for that. So, what happens here is, she took this post first to the [unintelligible 00:19:40] and they said, “We would like to do something around dementia,” some time ago. And she put her then – we asked her if she would, because she was a good fit in that, and because of her background working with communities, especially elderly people. So, once she’d said yes, she went on full training, and then she’s, as I said before, that she’s very thorough, so if you want to look at our website and check what we do around dementia café and everything, you’ll see the pictures and everything. So, she went for this full train the training programme.

When she finished that, what she’s done then is, they said, “OK, I would like to work with all of our staff and volunteers to train them.” So, now she runs silver, gold, but in these three sessions. I had been to silver, and up to gold. It’s understanding, so what is dementia, how you do ... So, all of our staff have been there, including managers and our board members. Same thing, we offer it to any new volunteers, any people. So, the big version, half a day version, there’s just overview of the things. So, even I had, last month, I had two students, occupational therapy students which came for a placement with us for eight weeks. They went actually with her. They’d done the two sessions with – they actually ran the session with her, and then they were trained. So, anybody who comes into our circle would be actually trained around that –

Interviewer: I see. And what do you think are some of the skills and knowledge that are required to support clients with dementia? What are some of the things that you’ve been trained on, that are really helpful when it comes to this population?

Respondent: So, [unintelligible 00:21:36] when I get a referral, which says – even doesn’t say anything about dementia or memory, it all starts from when I start speaking to someone, and then I can feel that, actually, there is something around here with the memory, because the person is repeating a few things, and we just ... So, I would go into the file and check, actually, is this person being actually referred to memory clinic? Because, as I said, we’re in the same building, I could go to the GP who made referral and I say, “I’m concerned about memory here. Please can you make a referral to memory clinic to start the process?” While they’re there, I’ll speak to [anonymised], who’s my colleague, saying, “I think, according to our training, I think they need some extra help.”

So, she, not saying that she may have it, she will then say, “OK, send me the referral.” So, she will get in touch with them and see if any family members she can speak to, start putting things in place. If nothing, we’ll just say, “OK, fine. At least we gave information.” If it’s just simple things, if they just need reminding of something, if they started taking their medication differently, so it’s just a little help in here, I just might be – next door to me is the pharmacy team, so I’ll go to them and say, “I think, look at this, and that’s, I think, the medicine need to be more – I’m concerned about medication taking, can you follow it up?” So, [crosstalk] or something, so that process will start, just to safeguard the person.

But, if we speak to family and they say, “Actually it's more than that,” then we’ll take it from there as where to go, but otherwise, it will be myself talking to aa few people here, and then [unintelligible 00:23:27] actually [anonymised] could follow up. Family member will say, “Actually, I don’t know what to do.” And this happened a lot, say, “Yes, these things are happening, but I don’t know what to do anymore because I can’t get my hand on what it is.” So, [anonymised] will speak to them and say, “These are the things. If these things happening, let’s talk about it.”

Interviewer: I understand. And, when you, for example, when you support somebody living with dementia, what kind of skills do you think you should have as a link worker, in terms of communication skills, in terms of all the things that are important, to be able to deal with somebody living with dementia? I’m not sure if the question is clear, [anonymised], let me know –

Respondent: Oh, no, I think what you said, what is – a renewal every year, at least, of the training which we’d done, the gold set especially. The way it was done was actually, it was like a role play, so you can see, when the role play was happening, you can see how the things were unravelling in front of you, and so actually, OK, but this one, this could happen. So, two people are talking, and one person is pretending to have that. So, it’s that type of training needs to be, I think, every year. So, you go back, because it just goes on, because too many things happening around.

But that local training needs to happen, and the staff – and it could be open to anybody, even link workers, receptionists. It’s like a life navigation. If you’re supposed to be part of the life navigation, anybody who comes across someone should be able to have a little bit of this information, so they can say, “OK, there is something going on.” As I said, in my case, speaking on phone, if I see someone’s down there, I could tell, OK, there is something going on. Let’s follow it up, why their memory is not as good as before, they’re getting slower on the things.

It’s because we have volunteers who’ve been with us for years and years, so yesterday – not yesterday, the day before- sorry, last Friday, I met someone who’d been 20 years our volunteer, first of all because of some accident. And, when I met her other day, I thought, OK, I need to follow it up because this is the time we need to put things in place, because it will be if somebody was repeating the things and missing a few things. So, I thought, “OK, we need to start the process to make sure,” because you can – the main thing which I see when people start on this journey is that one side of their brain is really active, and they tell you everything, and then one side, they can’t, so they struggle and they get confused.

Interviewer: Yes. Yes. It seems that one key aspect for social prescribing link workers to have been a basic training, as you said, around dementia, around what are the behavioural manifestations that tells you that somebody might have dementia. So, training seems to be quite important, from your point of view.

Respondent: Yes.

Interviewer: I see. And what kind of – so think about this new resource that we are creating, OK? As part of this study, we are creating a new resource to help social prescribing link workers. I wanted to ask you, what information do you think it’s important to include in our new resource?

Respondent: I would say I recently worked with some researcher from [anonymised] and they were creating a new resource called – what was it called? [anonymised]. And that was – that is about, while you’re waiting for your hip or knee surgery, it can take up to six months to one year. What do you do then? So, this is a website where you can go, you can look at the different resources, you can look at the different exercises. You’ve got things you can do while you’re waiting.

Interviewer: Yes.

Respondent: Similar, I think, very similar, is something where you have – you think, or you just want to know more [unintelligible 00:28:27] the person themselves or their family, carer, if they go there, what they will see. I think if I would go there as a professional, what I would like to see, very general, as you said, a little presentation of what symptoms could be. Where is the time to look into different, where is the time to [unintelligible 00:28:49] yourself as a professional, or even as a family member, when to ask for help, so in both ways. And what next could, should happen, if there’s a pathway, what is the general pathway?

Interviewer: Yes.

Respondent: So, if you have – so ask your GP, ask if they could make a referral into memory clinic, and that’s how it starts, generally. So, I have seen someone the other day which was, it was written, “Something is a danger of dementia,” or something, written, for the last 13 years, but nobody done the referral. No referral been through. I couldn’t see any referral, so I checked properly. No referral was done, the person has from the last 13 years, at risk of dementia, recorded, but no referral. So, I phoned up saying, “Can you make referral to memory clinic?” Because the person’s actually falling around, all that what is happening.

So, when they are actually active, they’re ringing out and saying, “I need help,” and when you’re trying to get back, she’s not even picking up phone. I had a client once who was not engaging on the phone. So, I went and knocked on the door and nothing. So, I went a second, third time and eventually we got the person sat on that day. And she was totally fine talking.

Interviewer: Yes, to be able to capture those signs, initial signs.

Respondent: Yes.

Interviewer: And what I find really interesting about what you told me, [anonymised], about your service, is that we tend to think that usually the pathway is, the person gets diagnosed with dementia, and then they are referred to social prescribing link workers. But it seems, from what you’re saying, that sometimes in your service, it’s the opposite, and you refer people to memory clinics to get a diagnosis, is that correct?

Respondent: I can’t refer because I’m not a medical [unintelligible 00:30:58] person, but I will follow up with the doctor or nurse who can. I go back to the referral and say, “Actually, this person who’s having falls, looking at that, I don’t think their memories are that good. Has anybody looked at their memory to make a referral? No, can you please look into it?”

Interviewer: Yes, I understand that.

Respondent: And that’s how my follow up will go.

Interviewer: Yes, and that seems very, very important because it triggers that process of getting a diagnosis, that we all know is very important.

Respondent: Sometimes for family, sometimes for people, sometimes for services, that diagnosis is the problem. If it’s not there, you don’t get the help.

Interviewer: Of course, completely agree.

Respondent: So, that’s why, while we are working, what we can do, we have the memory clinic, we have memory – and that side not memory – not the clinic, but the cafés, that we can support them. But on the other hand, to get the next step, they need the memory clinic to look into it. So, we do both things. I will work on the one hand, medical model, on the other hand, social model, say go to both.

Interviewer: Yes. No, I completely agree with that.

Respondent: And it goes back to, as you said, if I wouldn’t have that experience or know-how, or the links we have with here, or the persuasion I do sometimes, but a new link worker, it’s not possible. This comes with all experience, and how you are.

Interviewer: Yes, yes.

Respondent: Sometimes you have to fight for things, even you’re right, but you have to persuade the GP, saying, “Look, need to [crosstalk].”

Interviewer: Yes. Because is it correct, [anonymised], to say that you get, from your GP, you get referral of all sorts of clients, not just clients with dementia, right?

Respondent: Anybody and everybody 18 plus. 18 plus, and so the main thing will be, because they’re so busy, and so many times, you ask – so, which type referral you want? We send all information, again and again. So, sometimes I just be very, very clear and simple, and say, “Anything non-medical,” because if it doesn’t belong to me, I’ll say no, if it’s totally wrong referral, otherwise if I can sort out, it’s a non-medical, it’s nothing to do with you, might be something with me or somebody else, I’ll just signpost. So, for you to have it easy and simple, anything non-medical, housing, benefits, dementia care, social care, even the wheelchair, I’ve got.

Interviewer: So, that’s why you get lots of work. Now I understand. Wow, OK. And how do you think that the GP decides who gets referred to you? How do they screen if these people actually have social needs that need to be addressed by you guys? Or do they just send anybody?

Respondent: As I said, the main surgery – not over the years, because they’ve been sending, if they sent something wrong, I went back and said, “Look, this is not mine. This is mine, and this is not.” So, I’ll just give you a simple example, let’s say someone, a GP is sending, or reception is sending me a referral saying, “This person is asking for a letter, support letter.” That’s [unintelligible 00:34:26] support letter, social thing, send to me.

But I went back and said, when they asked for support letter, this letter is actually medical letter they’re asking for, from a medical professional, which I am not. And, I haven’t worked with this person, so I don’t know. If I’m working with someone and they need a support letter for their reason, I can say that, but this is what they’re asking. So, differentiate a little bit. That’s what my work, job is, to keep educating them what is a referral for a link worker, the right referral. If the work is non-medical – what is medical, I can’t do.

Interviewer: Yes, it’s interesting that you have to educate GPs about this.

Respondent: Yes.

Interviewer: That’s very interesting. OK. I don’t want to take too much of your time, Wokas, so I just wanted to really ask you a closing question, and that is, do you think there’s anything else at all that you think you want to tell me about this – we’ve discussed about, because it’s important for me to know?

Respondent: I think it’s what this research, or whatever you’re making, it is too, it’s a really important thing, because families, when they start, to those elements of this starting especially, they don’t – because in the UK, if you look at the white population, most of the people living on their own, family only comes few hours a week or few hours a day. So, it’s a big change. On other hand, Asian population, or population where they family lives together, it’s the same thing, because everybody’s busy, and the same person who was independent, and doing the things, suddenly lose that. They start falling around, or they just start forgetting things.

So, it is very hard for some families to understand what happened to the person, why we have to turn back and look after them, when they were actually recently looking after us, or they were independent. So, this is where, if we have enough information, where something comes in, and we straight away say, “Look at this,” say I am talking about pain work, if somebody works with me, I’ll look to put the pain toolkit in front of them, and say, “Look at these tools. It starts from acceptance, and goes all the way down, from who’s your team, and how to pace.” So, for them to understand, the best, biggest thing is to understand. Once they will understand, they will become part of it.

Interviewer: Yes, I agree, and yes, and that’s what we’re trying to do. We’re trying to raise awareness, first off, because it’s through awareness, and through knowing, and as you said before, word of mouth as well, that people eventually learn that there are opportunities for them to combat social isolation. So, that’s what we’re trying to do, and I really appreciate your support and your insight into this. I wanted to ask you, [anonymised], if you could ask your colleague, yes, if she’s interested in having a conversation with me, and you can tell her it could take also just 30 minutes. It won’t be long. It depends on everybody’s availability. It’s not a long thing, but it would be really helpful to support the project.

Respondent: No, I will ask, and I know that [anonymised] will be very helpful, and very happy to do this, because she is that type of person who would love to do –

Interviewer: Oh, nice. Yes.

Respondent: And talk about it, because she’s very passionate. She’s very passionate.

Interviewer: That’s commendable, really commendable when you have people who are passionate about what you do.

Respondent: Because, without that, it’s just – because this, especially, care of dementia, needs a lot of passion, compassion, time.

Interviewer: It does.

Respondent: So, what we have, what a community organisation like us have, is we’re local, we give that time, time before, not only going to when you go to the café, it’s when I make a referral, I know how it works. [anonymised] will take the time, she’ll actually speak as long as she has to, explain everything, then she’ll send information in the post or email, and she’ll make sure they have every single thing what they need, before they come.

Interviewer: Yes. Yes.

Respondent: It’s all the time taken and that helps people. So, people who actually said, and I’d spoken to them after, and they said how good it is because now they have a place where they can go and enjoy with their loved one, instead of sitting and thinking, “What shall I do now?” Because, we have actually two times a week, when we go together, we enjoy, actually. So, that’s totally different from thinking, “What shall I do now?”

Interviewer: Yes, yes. No, I agree with that. OK. All right, [anonymised], so thank you a lot for your time. That was really, really helpful. I hope to give you some updates about the study in due time, so that you’re in the loop about what we’re doing, because I think it’s important to also see how you helped to shape what we’re creating. So, I’ll let you know how we progress through the study and do spread the word about the opportunity to take part. We’re always looking for colleagues of yours to interview, OK?

Respondent: Don’t worry, one part of my, actually, job is – not part of my job, which I’m made of, is that I do help research. I actually do a lot of research help in the new, so today I was in a meeting, which is starting now. This week, I’m taking a group of community members to a place where they’ll be part of this group where they will be looking at frailty and robotics. So, I work with researchers all the time, so this is my passion, that I want people to help research.

Interviewer: It’s great. It’s great, and keep doing that, because if research is shaped by people like you, who are working in the real world, it is much more meaningful and relevant.

Respondent: Cool. But yes, keep me in the loop, and even the final report, I would like to see –

Interviewer: For sure. Yes, 100 percent. I’ll keep in touch, and hopefully we’ll find some more people to interview from your side. But thank you so much.

Respondent: I will send my colleagues, you send me the consent and everything, so I’ll email her, and I’ll copy you in saying, “Whenever she has time, she can ... "

Interviewer: Oh, OK. I’ll do that as soon as we’ve finished. I’ll send you the documents via email, OK?

Respondent: I have the documents that you sent in the email.

Interviewer: Oh, yes, well actually, you can –

Respondent: So, I can just forward –

Interviewer: Yes, you can just forward those ones, yes.

Respondent: Yes, I can forward those saying, I’d just spoken to [anonymised] and she’s happy to speak.

Interviewer: Perfect, thank you so much, [anonymised], and have a lovely day, and thank you again.

Respondent: You too. Thank you.

Interviewer: Take care. Bye. Bye.

Respondent: Bye. Bye.

[End of recorded material 00:41:43]

**SP02**

[Start of recorded material at 00:00:00]

Interviewer: Do you want to give me a bit of a background about where you work, what you do, and how you operate within your service with social prescribing?

Respondent: Yeah. I've worked in the NHS for a long time. Previous to this job I was a secretary in a GP surgery, and before that, receptionist, and then I worked in mental health and stuff which led me down the path. I was in the previous job for 12 years, and then I just wanted a change. I wanted to be more present with the help for the patients, the public. So, I saw this job, and there were two roles advertised, there was link worker and care co-ordinator. There's a fine line between the two, as you're probably aware.

So, I applied for both of them. I was interviewed by three different people, and they chose me to be care co-ordinator. Now, in my experience, the only difference between care co-ordinator and link worker is there's a more hands on approach to linking the person with medical care. And making sure that the medical professionals are accessing the person and the person is accessing the medical professionals in the same way, making sure all of that is – but I think there's a lot of crossover.

The link workers do the same, really, maybe it’s not as in depth as I might go. But I work for a company, at the moment, called [anonymised]. It's a small organisation who started off in [anonymised] and then expanded into [anonymised]. At the moment, we're in, do they call it, consultation? Where they have decided to let go of the [anonymised] division, we'll call it. But the PCNs that we've been working for have found so much value in our work that they're now taking us on.

So we're not unemployed, we're being kept on and my particular team is being kept on by [anonymised]. So predominantly, I work with [anonymised], I work with three GP surgeries. One of which, it was two surgeries which is now amalgamated. So I work with [anonymised]. And for a long time, I was doing care co-ordination and link work with residents in care homes, and also the wider age group, like from 16 to 17 and transitional ages up over.

And they've just started to streamline and narrow the case load that I'll be taking. So I'll be taking frail housebound dementia, that kind of cohort. And I've started taking a case load at the moment, even though we're still in talks about how that's going to look. But I have had frail people and people with dementia on my case load previously.

Interviewer: I see. So you got that experience already.

Respondent: Yeah, so I've worked here now, it'll be two years in November, and I suppose that will probably lead you to, have you got a next question or should I just keep talking?

Interviewer: No, what I wanted to ask you is, thanks for the description of your background, that's really helpful. I wonder within the service you're currently working on, what's the pathway for people to be referred to you? I suppose they would go to the GP and the GP will refer people to you. But you'll tell me about this. Also, I wanted to ask, within the same question, do you specifically get people who have been diagnosed with dementia? Or is it not always the case? But tell me a bit more about that maybe.

Respondent: So we get referrals, you're quite right, we get referrals from the GP surgery. And it could be various different routes. Like I got one yesterday. A gentleman who has recently been diagnosed with Parkinson's dementia has been to the memory clinic, the elderly person's memory clinic. And along with all the other people that he visits, the elderly mental health appointment that he had spoke to him, and this gentleman is not keen on engaging with anybody or taking help from anybody. He's relying on his daughter.

So they have said to the GP surgery, “Can you refer to social prescribing to try and get this guy to engage with the help that’s offered to him?” So that's one. Another one is the patients could be in seeing the GP, the GP might not know where to go with them. Just literally think this person needs something, we don't know what it is. Send them to social prescribing and it can be, like I say, anybody. There's no definitive pathway for dementia.

Interviewer: Yes, I understand.

Respondent: But I guess in my role, because I'm going to be taking housebound and frail, I'll probably acquire a greater portion of people with dementia than, say, my colleagues would.

Interviewer: I understand that. So is it fair to say that actually, [anonymised], sometimes it's you, the person who might potentially suggest that somebody has got dementia and that they would need to get a diagnosis. I'm asking because I interviewed another person the other day, this was from [anonymised] and they were saying that sometimes they get referrals for people who they then suspect have dementia. So they send them back either to the GP or to a memory clinic to get a diagnosis. It's kind of the reverse, if that makes sense. Do you suspect it's the same with you?

Respondent: Yeah. So if I was having it, I'm not trained in dementia. I've done my own research and I suppose that's going to be one of your future questions, what training I've had. But if I find that I'm noticing different things within the conversations, I'll report that back to the GP surgery. And like you say, that's quite right, that might result in a referral on to find out if there's a diagnosis there.

Interviewer: Yeah, which is great because, as we all know, the GPs have very limited time with people. They might not realise, so if you're facilitating in a way that process of getting a diagnosis, which is so very important for the obvious reasons, then it sounds as if you guys have a really core role within primary care.

Respondent: I would report any of that back but I also report, because I'll go and visit the person in their home. I don't believe that you can get a good gauge of what's going on if you're not seeing it first-hand. So I'll visit the people in their homes and I'll also let the GP know if there's any struggles that way. If the person's got a couple of steps that they can't manage at the front door, do a full 360 of what's going on and how they're managing in their home.

Interviewer: OK, that's great. And so have I understood correctly that you as a care co-ordinator are the first professional the person is referred to and then you pass them on to a social prescriber. How does that really work with the social prescribers?

Respondent: So because the umbrella is social prescribing, I'm a care co-ordinator and there are link workers. Up until now, I wouldn't pass any of my cohort on. I would just do forms and I think continuity of care, building up a rapport with that person to then move them on to somebody else when I could quite easily do that for them within that role. I personally have kept them with me and just done everything and seen it through. So I've done the care co-ordination and then if they've wanted linked in with social groups and befriending and things like that, then I've done that also.

Interviewer: Interesting, OK. So in all effects, social prescribing is also done by you as a care co-ordinator?

Respondent: Yeah.

Interviewer: So this is good to know because I thought at some point, the phrase care co-ordinator makes me think that you're the first person to whom they're referred to. But that's not always the case because they could be referred to a link worker as well, right?

Respondent: Yeah.

Interviewer: Yeah, I get it.

Respondent: But within my team, if somebody was referred, well, the way it happens is the GP will send the referral through. Our [anonymised] team get together every Tuesday and Thursday. We consider expertise within our team. We'll look at the referral and if dementia is mentioned, we try to pass the referral to the one who's better suited for that client. Because within our team, although they've all got a link worker role, one of them has a background in alcohol and drug abuse. One of them has a background in housing and care homes. Another has a psychology degree. Another used to be a counsellor.

So we would say, “Who's the best person to.”

Interviewer: Sure. You've got different expertise, and based on that, you decide who's suitable for that specific person, I see. That's really actually an eye opener because it makes me think that in this project, we're always saying, and the title actually, also says that, that we're creating resources for social prescribing link workers. But actually, because of the role that you just described, probably the aim should be creating resources for social prescribing. Because otherwise, it's as if we're excluding the care co-ordinators by saying we're creating just for the social prescribing link workers, does that make sense?

Respondent: Yeah, and on that note, I am the only care co-ordinator in the whole of [anonymised]. But I only work with [anonymised] and the rest of the funding, the other PCNs have decided to use that funding for link workers as opposed to a care co-ordinator, so, I'm it.

Interviewer: You're basically a rare creature.

Respondent: I'm a lone wolf.

Interviewer: Yeah, you are. So are you saying that you suspect also within the country, there's not many care co-ordinators would probably be the case?

Respondent: Yeah, there's not as many care co-ordinators as there are social prescribers. But on that note though, the training that we're given is the same. There's one small difference, which I've not had yet, and that's the two day accredited care co-ordinator, which is set down in like [anonymised] or something like that. That's the only thing that separates a care co-ordinator from a link worker.

From my experience, everyone within the company that I was employed by initially had exactly the same training.

Interviewer: Yeah, but that little thing that you done in [anonymised]. And what was that about, if I can ask that extra bit that you had for training?

Respondent: So that just came out recently in the framework, in the NHS guidelines, the gender for change and all of that. When all of those documents came out, that's when it said that a care co-ordinator should have a two day accredited care co-ordinator training. But because it's new, some of the care co-ordinators have been doing this role for a year, two years and not had this. And now there's a waiting list for it, so some of us are still waiting to get on this.

But I think, from what I’ve heard, it’s around £800 per person to go on this two day. And then like I live in [anonymised], so I would have to get from [anonymised] to wherever it is. I think it's [anonymised] or Manchester, somewhere like that.

Interviewer: Yeah, I see, but it's something that recently came up, so maybe there's a bit of a backlog before everybody gets that. And just one final question around the kind of structure of the services. I also read about health.

Respondent: [Unintelligible 00:14:26]

Interviewer: No, another role within social prescribing, is it health and –

Respondent: Health and wellbeing coach?

Interviewer: Yes How does that fit, that role?

Respondent: So we have a health and wellbeing coach. She was a care co-ordinator like me and then they moved her over to being health and wellbeing coach. And her role had a couple of prongs to it. The first one being virtual group consultations for different cohorts. She had a very successful one on menopause. There was another one on healthy eating, another one on diabetes. And what they do there is they get like a group of people on a group consultation that have a doctor there, a pharmacist there, say a mental health worker there. And then [anonymised], the health and wellbeing coach would facilitate that, organise that, and then they would deal with each person.

And they would have maybe breakout rooms and have a one to one or if they were happy to discuss in a group setting, they would do that. But the GP was there to do any prescriptions that needed done. The mental health worker would be there to organise an appointment. It was a really, really good resource, the virtual group consultations. And then her other role that she was designated with is the first 100 days of life.

So people wanting to get pregnant and supporting them from day dot to 100 days all through vaccinations, through health and wellbeing, through everything.

Interviewer: That sounds great support.

Respondent: And other health and wellbeing coaches do different things but that’s what I know of what [anonymised] did.

Interviewer: So I guess the natural question that will lead us to the dementia area is who's, for lack of a better word, in charge of dealing with dementia clients within your service? And how do you really approach this population group?

Respondent: So first off, I should tell you that the company we work for are self-managing teams. So we don't have a line manager, all we have is the people that own the company and then a couple of HR and then there's no hierarchy at all. So as for link workers as a whole, if you're designated a person, if you're tasked, referred to a person who has dementia, it's basically the onus is on us to make sure that we're clued to source the training. To link in with the Admiral Nurses and Age UK and other dementia organisations, to link in and find out. It’s down to us, there’s no –

Interviewer: There's no set rules, basically.

Respondent: Yeah, so the GP will send the referral. They don't know who they're going to send that referral to within the team. It's us that decide who gets that person.

Interviewer: I get it. OK, so it's a bit less formal in a way because you're amongst yourselves. So you decide amongst yourselves. I remember you said before that you got particular areas of expertise within the team. So I was wondering who's got that in dementia and if not, who's got a bit more experience in that area?

Respondent: So I suppose it was me and me and [anonymised]. I did have [anonymised] as a colleague. She was a care co-ordinator along with me. And like I say, we both did a lot of work in the care homes and we would generally be given the older population. [anonymised] has since decided that that makes her too sad and she wants to work with people of all ages. So she's gone off to be a link worker and left me to be, so it would be me.

Interviewer: It would be you which is great.

Respondent: That's in the [anonymised] team though, [anonymised]. That's in the [anonymised] team. The other teams like [anonymised], they're just link workers. They haven't got hard and fast, who's better at what. They just share that out.

Interviewer: So this would mean that within those teams, when they get a referral, how does it work differently than you guys?

Respondent: Just because I've got the title, care co-ordinator.

Interviewer: OK, I see, whereas they don't have a care co-ordinator?

Respondent: No, they don’t have one.

Interviewer: I understand, thank you. So let me ask you, since you are the one who usually deals with this group. What do you think would be helpful for you to see in a resource that's freely available on the web? Say, for example, a website, what would really help you in terms of maybe information or maybe in terms of resources? It's really an open question just to get a sense of what would be helpful for you.

Respondent: For me, particularly with dementia, it would be important to learn what is the pathway from a person seeing to a GP? How do they reach the conclusion of the diagnosis? And then from the diagnosis, what happens? Does that make sense? So what does the patient go through when they go to the appointment and somebody says to them, “You have dementia,” what happens then? What resources is the patient given?

Because I only get what the patient tells me. And sometimes it's, “Yeah, I can't remember. Everything was just all over.” So finding out that, and I have done some, I've looked up my own bits of training in regard to dementia because we're not given any at all. The National Association of Social prescribers is the only mandatory training at the moment that social prescribers have to do once in post. It is made up of 10 modules that you have to complete. But there’s nothing about dementia in it. We're given this mandatory training and then we're told, “Go forth and find out what you need to find out about dementia.” And we go and source our own training.

See, I link in with the admiral nurses, so I get a bit of information from them. I also can link in with the carers centre for people that are needing carers and things like that. But it is just like a dummy's guide, like this is what happens from point A to point B. And I know that's a lot of different dementias, I've gotten my head around some of those. There's a dementia linked to alcoholism, there's a dementia linked to age, there's a dementia with downs syndrome. There's all of those different ones.

And it would be like what treatment is given to people, like what can I explain to the person that maybe has been explained to them already? What can I explain to them again? One of the reasons the social prescribing has been put into place is we take away time from the GP surgeries, we save the GP surgeries time. And if we can do that for the dementia services as well, we’re like little spiders crawling out into the community now, it's bigger and bigger and bigger. So if we can help the person and help the service, that would be –it's just forewarned is forearmed, isn't it? Being able to answer the questions of the person.

Interviewer: But in order for you to be able to do that, I completely agree with you, you need some basic information around, for example, as you said, what are the types of dementia? And what kind of symptoms and needs come with that specific diagnosis? Say somebody has got Alzheimer's, it's completely different from the needs that a person with vascular dementia might have. So there is a need for, I get that, information around dementia. But also information on, as you say, quite crucially on the pathway that people go along when they get the diagnosis.

Now, the general thing that we hear from people is that when they're given a diagnosis, they themselves are not given any further information about what's next. What do I do now? What's in place for me? What kind of benefits do I get? What kind of support do I get? So, I agree with you that when the person comes to you, most of the times they can't really tell you what they told them because most of the times they're not told much.

They're given the diagnosis, “Here you go, see you.” So, it's about making sure in the first place that the person is given something other than the diagnosis. But it's also making sure that you guys who are, I can tell increasingly the point of reference to support them, because as you say, the GP hasn't got much time. You're there to take away that time, that you're in a position to know where you can signpost people.

There is a website, this is part of our previous project at [anonymised]. We developed a website called [anonymised]. I don't know if you're familiar with it. It's freely available on the web. This website was intended to provide information and resources when you first receive a diagnosis. So within this website, we have a section for professionals where it literally explains what the pathway is and what to expect when people get a diagnosis.

So what I can do, [anonymised], is first off, I'll send you an email when we're finished with a link to the website so you can explore it. Because what we're looking to do, potentially, is to create a section there for social prescribing with this information that we're developing and which is needed. So I'll do that, so you can start maybe looking at that one and see if you find it helpful to begin with. But we're going to be integrating with more and more information that's needed from people.

Can I ask you, you mentioned mandatory training before, can I ask you what that consists of roughly?

Respondent: I thought you might ask us that. So I've got the list so bear with us now, I’ll just reload. So mandatory training, it's very, very basic. So mandatory training is things like information governance, safeguarding adults, safeguarding children, resuscitation, preventing radicalisation, NHS conflict resolution, moving and handling, motivational interviewing. Infection prevention and control, health and safety, fire safety, equality, diversity and human rights, core skills and something called Connect Five.

That is the mandatory training. And then we source our own non-mandatory training, and I've done quite a bit.

Interviewer: I see. So within that mandatory training, is there anything at all around dementia that you remember of?

Respondent: No, nothing.

Interviewer: So it's down to the person?

Respondent: Yeah. So we do things like we'll go onto Washington Mind, Alison, Personalised Care Institute, Eventbrite. And we're given those names and it's like, “Here are some places you can get some training, go and find it.” And so that's what we do. So I've done personalised care and support planning, I've done dementia awareness, learning disabilities awareness. I could read them all out to you but I would bore you to death.

Interviewer: I get the point. Do you think that based on your case load, you've got loads of experience? I was wondering if you've seen many people with cognitive impairment and dementia? And if so, do you think that mandatory training in that area would be better than just letting people decide whether they want to get it or not?

Respondent: 100%. It should be mandatory. It definitely should because regardless of my role as a care co-ordinator, the people that are being referred, it might not even just be the person that's been diagnosed with dementia, it could be their partner, it could be their son, it could be their carer. And you need to be able to help those people and understand the person that they're caring for or living with, it should, 100%.

Interviewer: OK, that's a great point in fact because dementia does not affect only the person who's got it. Sometimes actually, it affects more those who are around them. And if you don't have that background info on why a carer might be so burdened, then you don't know how to help them. So I get that point. So mandatory training would probably be the way to go which is great to hear.

Respondent: Paper resources would be good as well. Like if there was something that the hospitals or the dementia services give out to patients, if we could have access to that as well and say, “Did you get this? Oh, you've lost it. Well, there you go, there's another one.”

Interviewer: I see. Yeah. If we're talking about training, what kind of training do you think is a bit more engaging, is a bit more interesting? What kind of format do you like in training?

Respondent: I've always found shadowing a really good format for me. So, I mean, obviously, if you're putting it on a website, and it's something we need to access, I think having like a video of the person in their appointment, and then maybe shadowing and that way, being able to watch what happens. Watch videos of how it happens. We can read, you can send us documents and we can read through but we have a lot of different people on my case load and a lot of things to remember.

So being able to go to a particular website and go, “Oh yeah, I need to freshen up on this.” We're being able to watch those kinds of things and having a, like you say, a guide on this type of dementia, this type of dementia, this type of dementia and underneath those headings, the problems, the things that might come up against the things they might experience or need help with.

For the likes of benefits and stuff like that, it's not our job. So we will signpost and we've got people within the DWP who we rely on and who are really, really helpful. And we'll just signpost and say, “Can you sort this person out?” And they generally do.

Interviewer: Yeah, but it sounds that your kind of preferred format would be having some sort of practical videos, ideally, where you see the real life situation, how the process is when they get a diagnosis, and maybe kind of simulate almost a shadowing experience. Because you're looking at the video, it’s as if you're in there in a way, rather than reading something that makes you a bit more detached.

Respondent: Yeah.

Interviewer: I get that, OK. Yes, in [anonymised], we have lots of videos. So that tells me that that's probably the way to go if we are integrating with something for a social prescribing workforce.

Respondent: The link workers and myself actually, we always like to be able to have a certificate. So some sort of accredited training, I think you'd get more people going, “Oh, well, if it's accredited, I'll come and do it. If it's going to give me a qualification, then –

Interviewer: Yes, that's another good point, because it's also about professional development and looking at future development, isn't it? OK, that's a good point. So, this was great, lots of information. So I'm super happy with what we've discussed. Was there anything final that comes into your mind or are you happy with what we've discussed?

Respondent: I'd just be really interested in what you're doing because, like I say, one of the little discussions that's been going on in the background, I haven't decided fully, is whether my role would include doing things like the Rockwood Frailty Score and the Dementia Care Reviews. And then one person that's looking at my role is saying, “No, no, the doctors do them, the nurses do them. You don't need to be doing them.”

Somebody else is going, “Well, it would actually free up a lot of time if you could do these in the community.” So it's a possibility, so I think just being clued up and more information about this subject would just be so helpful for me.

Interviewer: Yeah, I agree. Because that could be a bit overwhelming if at some point you're asked to do that, but you haven't got that information on what is it? How do I go about it? Yeah.

Respondent: And I could link in with the care co-ordinators all over the country because I'm in a WhatsApp group with them all. And they're so helpful and they've offered to let us do like a Teams and see how they do things because some of them do the Dementia Care Reviews. So it would be helpful, I think more is better, rather than just saying, “Go and find out yourself.” Having a little bit of structure and saying, you are going to come, it's inevitable, you are going to come across a person with dementia or somebody who cares with a person with dementia because the numbers are so high, it's going to happen. So being armed is better.

Interviewer: Yeah, no, that's great. I was wondering, Respondent, how would I be able to access more care co-ordinators if I wanted to have a chat with them, the same as I did with you? Would you be able to potentially signpost my contact to your colleagues?

Respondent: Yeah.

Interviewer: Because that will be super helpful because as you said, you guys are not many but you are such a crucial part within social prescribing. I don't want to miss out on hearing from your side as well.

Respondent: I'll give your email address in the WhatsApp group for the care co-ordinators for the rest of the country.

Interviewer: OK, that would be great. So that maybe I can get in contact and hear from somebody else from your group. That would be really helpful. Did you say you're going to give me their email or do you want me to?

Respondent: I'll give your email to them.

Interviewer: I see, OK, good, yes, please. That would be really, really helpful, [anonymised]. I guess that's it. So, what I'll do is I’ll stop the recording first off.

[End of recorded material 00:35:50]

**SP03**

[Start of recorded material at 00:00:00]

Interviewer: A general question for you in terms of what is your role, and what you do in your practice in terms of social prescribing?

Respondent: So my role is called social prescribing link worker. Technically, we’re being trained to link our patients with the services that can be further beneficial to them. We use our techniques of motivational interviewing to explore with patients what it is that they actually want, not what other people want for them or what they think they’re expected to want.

And we identify those goals, something they would like to do, look into barriers and try to remove the barriers for them or activate them so they able to overcome these barriers.

Interviewer: This applies, I suppose, to people living with dementia, do you support any people living with dementia?

Respondent: Yes, I have quite few actually. Each of them is very different. I've got some patients that live completely independently, and that's where it gets a little bit harder. Because the progress is a little bit harder to track, especially if there is a lot of frigidness involved. Sometimes it can be really hard because I've had a couple of cases where patients memory started deteriorating over a year ago, but it wasn't picked up on.

So they haven't had a memory assessment, they haven't had a diagnosis and I start working with them and then realise that actually they don't retain the information from previous consultations or they may seem a little bit confused. So we put in place for them the memory assessment to actually see if they’re eligible for further support or how they actually could be supported further.

Interviewer: I see. So remember when we had that training day, I was just sharing with you guys, the fact that what's coming out of other interviews is that sometimes people have not been diagnosed with dementia when they are referred to you. So this is kind of confirming what you're saying, right?

Respondent: Yes. And it's happening a lot, especially when there is a language barrier. So I guess it comes from that somebody who's got limited English can actually say during those consultations what they want to say or they forget that they wanted to say something. So this can go undiagnosed for a very long time. Very often it's in combination with poor mobility as well. So when I actually speak with a patient, I feel something should have been done a year ago.

And now things start moving, they’re referred for rehabilitation, they’re referred for the memory assessment, they’re referred to adult social care, and now they started getting the support they are. But like one of my patients told me, they’re absolutely grateful for our service because they felt like nobody was interested in them until now. And that's so sad.

Interviewer: Yeah, it is sad. It is sad but at the same time, it really shows that social prescribing has really got a central role in the process of health and wellbeing for people living with dementia which is great. It's sad but it's also great that at least at some point you were able to step up into the process. You mentioned before motivational interviewing. That's something that really catches my interest. Do you think it's working with your clients living with dementia? What do you think are some of the barriers there?

Respondent: So, it's a little bit difficult to say. Because there are, there are four components of motivational interviewing, affirmations, open questions, reflective practice and summaries. And I think what happens is we use these in different portions with different clients. So for example, with people who may suffer from dementia, the open questions work for me better than actually affirmations or I use it more because it helps me to find out what the person is.

So I've got somebody who says, “I would like to go out more.” And then when we explore, “Why would you like to go out more? And what could you do?” We actually arrive at the point where they say or where we find out that actually they don't want to go out more, but other people want them to go out more. So they feel like they should be going out more.

And we actually find that that’s not what they want to do. They have other interests. So we may arrange for somebody to actually come in as a befriender for that chat so that they don't feel lonely and isolated. But it helps us a lot to explore what the person wants and maybe identify. If somebody tells me, “Oh, I didn't want to do that,” then I can actually use motivational interviewing to explore why. Because sometimes the reason may be, “Because I can't walk very well.”

So then we get a rehabilitation team in place that can support them with that. So I do find it quite helpful just in a different way and different parts.

Interviewer: So because of the characteristics of the specific population, you find that some of the components of motivational interviewing are more helpful than others. And you mentioned the open question because as you explore with the person really what they want as opposed to what other people might want from them. Out of curiosity, did you say that affirmation is a bit more difficult with this population you find?

Respondent: Not difficult, but I think I use it less. So I do use all the components but affirmations in terms of motivational interviewing, they have to be genuine. Everything has to be genuine for a patient to relate and open up because people can detect when something is said just like, “Well done,” and it's not meant. So we actually find meaningful affirmations in terms of, “You seem to be very strong about this,” or, “You have been doing so well handling your finances so far.” And we find that particular thing that they really strong at.

But it can be harder with somebody who's suffering from advanced dementia. And I don't feel like, not that it doesn't have such a big impact, but I'm not sure how to explain it. But I don't feel like so far with the patients I've been having on my case load that I used it that much. Because I found other components more beneficial.

So like reflective practice and reflecting on what's been said and open questions, I actually find them more useful for me during our interaction. So I use them more.

Interviewer: I understand. That’s very clear, thank you. Just to really a couple of final points about this motivation interviewing because I found it really interesting. And what I've seen in other contexts, people do not use it, or they do not use a specific framework to have those conversations with the clients. So I wanted to ask you, is it something that you guys are routinely trained on, motivational interviewing? It's embedded within your service.

Respondent: It is. So it's embedded within our role where we have a statement that social prescribing. Link workers use motivational interviewing to empower and activate the patient. And it really works. So I had somebody who I was about to discharge and they didn't want to be discharged because they felt like they were losing that access and connection. And somebody finally listened and now we were going to close the case.

So I actually use motivational interviewing when I would ask, “OK, so can you tell me how beneficial the motivational interviewing is for you right now? What are you getting out of that?” And they started talking, “Well, actually, nothing at the moment.” And I'm saying, “So, why do you think we should keep your case open?” And they start talking again and they arrive at a conclusion, “Actually, there is no reason to keep my case open.”

And it's them actually setting their goals to rules, it's not me telling the patients what they need to do, rather it's them arriving at that conclusion. And then it has a bigger, higher efficiency in actually reaching those targets. For example, if they said they want to go out, but it comes from within and they arrive at that conclusion, then they're more likely to continue with it even when I'm away because it was them.

It wasn't me telling them they have to do that. And it's the same with the discharge process because they arrive at that conclusion. There is not a little bit of regression that can occur at the end of the therapeutic relationship. So we’re actually avoiding that as well and increasing the efficiency of the relationship from previous encounters.

Interviewer: Yes. I guess you touched on a very important point which is actually when a person develops that inner motivation, as opposed to motivation that's instilled in them, they are more likely to continue with the behaviour, whatever the behaviour is. And it seems that that's what you're trying to do, especially towards the end of your report with the client which is great. I think in relation to this person you were just mentioning, what we found, particularly with participants in dementia and receiving interventions, is that when the intervention is about to terminate, that kind of anxiety, almost that instinctual anxiety starts to develop. Because they might have built some dependency toward the therapist or the person supporting them.

So we feel it's absolutely happening in people living with dementia. Do you find in your experience that this is the case?

Respondent: Yeah. So, I had one particular hard closure where the person was living alone in a house. They didn't have much visitors. They were borderline. So they were not completely clear cut diagnosis, but they were very forgetful. And it was really difficult to follow the plan. So we devised a diary and everything. And what helped us towards the end was I've put in place, unfortunately they no longer operate, but there was an organisation, Independent Age. And they would come to people's homes, spend some time with them, take them out.

So I think it is important to find a local organisation who offers to go to people's homes, spend some time with them, take them out. And collaborate together. Send them the resources you created for the client, the client’s information with their consent, and details of everything that you did. And then the organisation can take over

Interviewer: Yeah, it's having those feelings that you're not left alone and knowing that there is, at the backdrop, a support network that you might resource to, if you need to. So I think that really helps with the anxiety issues that this group might experience.

Respondent: Because if I may just say something, one thing. Because the people who suffer with dementia, they may be going with forgetfulness for some time, their environment gets used to it, so they start ignoring it. But they may feel like they’re left alone for some time and then Suddenly the social prescriber comes in, they listen, they try to put things in place. They've just been given somebody who's there for them. So, sometimes those people do hang on. So having that gradual transition is very, very important.

Interviewer: I completely agree with that. So you touched on motivational interviewing and how beneficial in some respects specific elements are with this population. Do you think that in the resource that we're trying to create, there should be referenced to motivational interviewing?

Respondent: Yeah, definitely.

Interviewer: And another thing is that you touched upon is the fact that you get people, people refer to you. And then at some point in the process of supporting them, you realise they might have dementia and they're sent back to official diagnosis. So I was wondering, [anonymised], how do you guys, in general, really understand that there is something going on in the person and is it just purely based on your experience? Because I guess it's a bit of a risk in services to just rely on you guys to pick up those signs and refer the person back. So I was wondering, how do you see the person has got some issues and how do the social prescribers know about dementia actually?

Respondent: So, we've got a basic concept. We've had training on dementia, I think it's level two. But I personally don't think it's quite extensive and there should be much more in it. Because I can have different clients. It happened in the past that I called somebody, and they tell me I don't need anything. So, I closed the referral, but then later I received a call from their sister, saying - yeah, let's do this. I've got power of attorney - But those people that don't have the sister, they may not have that second chance. And I'm not quite sure really how to prevent that because unless we have that conversation with some patients, we don't actually realise something is going until third consultations because then we realise that actually, they don't retain the information that we spoke about.

But it's not something we would frequently screen for. So it comes from the communication from talking. It's not like we've got a screen list or anything. And I do think that we may be actually closing the referrals for patients that could really benefit because they say they don't want to engage. But then if we called on another day, or if there was somebody with them, they might say something different.

Interviewer: Yeah, it's something that came up a lot when we did, last year, the study to build Forward with Dementia. We interviewed lots of social care workers and what they told us was really difficult, was what we call a Rejection of Care, is that person with dementia experiences because somebody who's unfamiliar, they do not know who the person is. They see this stranger, essentially, and most of the time they have this process whereby they just say, “No.”

And also, as you say, it depends on the days, because it fluctuates a lot. So you might have a day where they're a bit more engaged and open, and other days where it's really a, “No, no.” But I understand your point, and it's a good point. If you actually have that one call to really decide and offer that service, and you give that call to the person on the day when they're just rejecting everything that's thrown at them, then you just lose them along the way, because they haven't got another chance, right?

Respondent: So what we try to do is if somebody is straight away, “No, no,” we tend to ask, “Are you familiar with the service? Would you like to talk about a little bit more?” Or we just say, “OK, you don't want to, is there any reason for that?” Or we just ask, “Can we call on another day?” And we try not to end the relationship right then, rather than when somebody maybe – It even happened that somebody was frustrated from an encounter with HMRC and they just were in no mood to talk and just shut that down.

So we try actually not to act on that first call when somebody says, “No, no, no, I don't want anything.” But we try to book the time to actually talk to them, explore with them. And it's very common with elderly people and people with dementia, that they actually don't realise there are so many things they could and they only realise when we start talking about things. I had somebody who wanted to be linked with activities and exercises, but when we started talking, they wouldn't really engage. By digging, I identified mobility issues, bereavement and financial issues that created one big barrier to engagement. And once we started addressing them, the client started to engage.

Interviewer: Yeah. And these conversations that you initially have with patients, are they occurring over the phone?

Respondent: Most of the times, yes, but that's another thing because we're still going on the guidelines to limit the face to face contact. But patients can come to GP surgeries or we can meet them in the community, or we do home visits as well. But as a standard rule, the first contact is always over the phone.

Interviewer: I was asking because there might be barriers there with people living with dementia. You receive a phone call, young people are reluctant to say yes over the phone to strangers. So I wonder whether that could be an ulterior barrier.

Respondent: It could be as well. What I found complicated for me, and this is for your research, right.

Interviewer: Yeah.

Respondent: Because I'm going to share something with you because –

Interviewer: [anonymised], just let you know, you will read on the information sheet, whatever you're saying is completely confidential and your name will never appear. So we're only going to be using codes, so rest assured.

Respondent: Thank you. I think this is a big problem, not as a huge problem, but we discourage from face to face contact as for home visits because it costs time and expenses for travel. So we almost discourage from that. And it's really hard because I feel that we should have face to face contact with every patient because you're assessing the person as a whole. It's a holistic approach. So even the way they're walking, how they look, how they move is important.

And we don't get that opportunity because at the moment there is quite a bit of pressure on not doing the home visits because it costs.

Interviewer: And this is also after the initial contact, is it throughout the process?

Respondent: Yes. So when I do home visits, we don't have an alone policy in place at the moment either, so it's being developed. We don't have really proper policy on, no protective devices, nothing. So at the moment, if we go for a home visit for the first time, we've got to go in pairs. But then we go by ourselves because then again, it costs more time because another member of staff is tied down in that visit. It costs them time to get there, it costs them expenses to get there.

So, even though our company actually says, “Yes, we do everything.” I think this is this is a big problem, but we are kind of discouraged from face-to-face contact for home visits because it costs time and expenses for travel. And if we do it very often, it's on our own cost as well. So we have to pay for the travel. We have to make up the time we spend traveling.

Interviewer: Which really doesn't encourage you guys, as you were saying before, I really understand that. And particularly, I think with patients living with dementia, again, this would be quite crucial actually to develop that initial report. But also for you guys to see what the environment and the situation of the person is a bit better than over the phone. So I take your point that face to face would be ideal, actually, particularly with people living with dementia.

Respondent: And even home visits, because then we can see the environment, how they live, what it looks like, is it unkept? Is there any hoarding? We don't get that information over the phone or at the GP surgery. So I actually feel that home visits are really important.

Interviewer: And particularly when you're talking over the phone with somebody living with dementia, the information that they relay to you might not be accurate of course, because of the issues that we know. So you're relying on that information to actually build a care package, but sometimes it might not be reflective of what the real situation is.

Respondent: And we've had the cases when we referred somebody, for example, to community rehabilitation team because they told us, “This is how my house looks like.” And then I read from the report that's not the case. So yeah, it happens.

Interviewer: Yeah, maybe even more so, as you said before, with people who live independently and maybe they are either from abroad and maybe don't speak the language 100%. So these are all added barriers, do you feel that?

Respondent: I do, yeah.

Interviewer: What can be done really, if anything? Because obviously you say, the face to face visits would be ideal, but the reality is, as you suggested, that services do not encourage that because there is always a balance in costs and benefits. So I wonder, do you have any other strategies that you adopt at the moment when you really want to engage with a client effectively?

Respondent: So, I think we have a great team of really compassionate and kind people, so we do do home visits. We don't do it for every patient, but if I feel, and especially if somebody is elderly, then I would like to see them face to face. So if I can't necessarily do home visits, I invite them to the GP surgery to see them. But if I do feel somebody struggles with mobility, I'm not sure whether they need any personal car or social car referral, I will arrange my time in a way so that I can go. But it means that sometimes I do have to make up time for travelling.

But we do home visits because I think our team really cares for our patients so we try to do our best.

Interviewer: Based on the kind of needs of the patient, I suppose you have priority groups with possibly where you say, “Well, this is a case where it's necessary.”

Respondent: I think this thing would be resolved within the organisation, if they had a clear policy on how to do the home visits, what is acceptable, what is not, how is it with costs, then they wouldn't have to be worried about what we do and how we do because we would be following these policies. So it really lays with the organisation that employs the social prescribers. I don't think anybody else can do anything.

Interviewer: Let me ask you a couple of questions, [anonymised] about the level you think people are aware of social prescribing. So in your experience, do people in general, maybe particularly people living with dementia, know that it exists? And if not, because that's my impression so far, not all people know what it is, how do people get to know about it? How do people get to the point where you can support them?

Respondent: So, I don't think any of my patients suffering with dementia actually knew what it was or who I was. But I explained, “We can have a chat, we can explore what's important to you, what you would like to do, and then have a look if there are any services that could support you with that.” So that's during that initial contact. Another thing we do is we go into community. So just yesterday, we were at the Community Health Hub, which is organised by PCN. There are different things. There's access to free hygiene bank, food bank vouchers. There are some donations that people can take in the form of clothes. And there are also stalls with stop smoking services, prenatal care.

There were us, and this time there were pharmacists advising on diabetes and a food car and podiatry in general. So they do these community hubs every two, three weeks. And we try to have their presence every time so that we can talk to people. So we have our own stall. I'm clinically trained as well, so I check their blood pressure, weight, height, BMI. We can advise them on weight management programmes straight away.

We can signpost people that have maybe problems with their landlords or isolation or they need maybe boiler exchange. But people are more likely to talk in their communities than actually over the phone or at the GP surgery. So we go to different events. In a couple of weeks, there's the [anonymised] that has always a couple of performances. So we're going to be there as well.

And we go to Days of Action organised by the council, again with a stall and we talk to people. So we try to increase the knowledge of us being there and being able to help. The problem we've encountered and kind of a barrier is that not all social prescribing services work the same. And some of the patients already access social prescribing somewhere else, but they were just signposted and they didn't find that helpful.

So the social prescribing service we work for, they're really big in motivational interviewing and really exploring with the patient. It's not just giving them information where to get the service. It's about helping them to connect with the service and maintain that connection.

Interviewer: Yes, that is a big difference actually, just signposting people to a service, it's very different from actually having that integration of the person, but also that important continuation once you guys step down. I think that's quite crucial. Did you get any feedback on why it wasn't particularly helpful with other services?

Respondent: The question was why it wasn't particularly helpful.

Interviewer: Yeah. I was just curious to ask.

Respondent: The person was signposted to the company they've already tried to get in touch with and they didn't find them helpful. They couldn't get the support they wanted.

Interviewer: Was this person living with dementia that you're aware of?

Respondent: Not this one in particular, no. That was from the general population. But that's another thing because I know we're networking, I know about some services that actually have only three contacts per patient. Initial consultation, signposting and follow up and that's it. It's not our service. I have to say, we've got an amazing manager who really fights for us to have time for our patients. So we get an hour for an initial assessment, to have the time to talk about everything and the follow ups can be as long as one hour as well, as long as the patient needs it.

And we're not limited by the amount of contacts. We just keep them on our case load until we feel that there is either nothing to link them with or that they achieve the call.

Interviewer: I see. There is that flexibility in terms of the regularity of meetings, but also the length of the support.

Respondent: Yes. But our other services don't have that.

Interviewer: There is huge diversity. That's what I am seeing from service to service. And that has an impact on the benefits that actually the patient achieves. So, there are very different experiences of social prescribing from what I'm seeing, and a huge spectrum of diversity in between. And I really think it's important, the work that you guys are doing in terms of outreach, going into the community to let people know what this is about, what it can do for you, how do you access it, and what the benefits could be.

Because as I said at the beginning of my question, I don't think lots of people at the moment know what it is and what it can do, and how it can help, so it’s important.

Respondent: I kind of think that the title, social prescribing is a bit unfortunate because when I introduce myself, people think two things. Either I'm from the social care or I can give them prescriptions. And it's really hard to go through everything and explain to them, “That's not what I'm doing. I'm not clinical. Let's talk about anything that's on your mind. But it can be quite difficult because the title itself suggests something completely different.

Interviewer: That's an interesting point. How would you ideally call it?

Respondent: That's a hard question. I think navigator sounds good, something in front of that, but I know we've got community navigators and I'm aware that social prescriber was chosen so that it would be more accepted within PCN and GP surgeries. Because it suggests that we can do something for our patients. It gives us a little bit more power to do things. So I know why they chose the title, but when I call my patient and say, “I'm a social prescriber,” they don't know. And they are creating this inaccurate expectation of what I can do for them.

So I'm not quite sure, health and wellbeing navigator. I'm not quite sure how to phrase it because we cover lots of stuff. We cover clinical health inequalities, life adversities, mental health. So I'm not really sure how to call it.

Interviewer: It's really a holistic package that's got so much in it, that it's difficult to define it. I understand. And I also understand where they're coming from, in terms of using the word prescribing. It really links it back to primary care somehow. The clinical work that people are doing in prescribing stuff to patients. So I can understand also that rationale there.

So let me just ask you a final point, because I don't want to take too much of your time, I’m already exhausting you. What do you think is helpful, really helpful, if you can think of a couple of things to include on the website for you guys to be able to help people living with dementia better?

Respondent: I think, generally, what would be nice, because I was at the initial training, it would be nice to have some information about examples of the social prescribing with people who live with dementia. Because I think it was very helpful to see particular case studies. Because it just gave me more idea how I can talk to my patients or how I can carry my visits. And it wouldn't have to be case studies, but it could be something that would show steps or strategies. Strategies, that's the right word, yeah.

Interviewer: OK, different strategies that you can use with the person, maybe in such a way that it's potentially in like a table format or an illustration, something that's easy to read and manage as opposed to a long text, maybe?

Respondent: Yes, yes, like points. Like when you're working with a person living with dementia, things to consider. This is the way how you can lead your consultations. This is how you can ensure that they understand and are able to follow the plan. Kind of like a hints and tips.

Interviewer: Yes, I get that. With the current website that we've developed, [anonymised], people have told us that they also found short videos, short animations, quite helpful. Not necessarily based, as you said, on real case studies, but just you, again, explain that process and those strategies. Do you think that videos on website are good?

Respondent: That would be good when I have time. But what I tend to do is when I'm on the go, I tend to skip the videos. So, yeah, I agree, they are good. As a part of the training and everything, they're brilliant because they're short, they're to the point.

Interviewer: But I take your point that sometimes maybe it's more helpful for people on the go to maybe print something so in a written format or an illustration is sometimes easier than actually staying there and watching a video. I can see that.

Respondent: And I think, I know this is the website that's focusing on people living with dementia. But for those that have not been diagnosed yet, I think it would be nice to have a checklist as well of the things, like red flags or something that would tell me, oh, this person might be actually living with dementia. They have not been diagnosed, maybe that's something I would need to look into.

Interviewer: Yeah, some of the signs that might indicate that there is something going on there. Maybe one of those signs, which is actually quite important, you mentioned before, is if you get that initial resistance, that those no's at the initial contact, don't maybe just give up on the person. And maybe explore if there might be something underlying, that initial no.

That could be one of the signs, potentially, I suppose. And do you think [anonymised] that a website is something helpful or do you think that people like you working in social prescribing would prefer another format for this information?

Respondent: I think the website would be great. Because we’re constantly looking up the information. Everything is changing so quickly. So I am actually on my browser the majority of my time and ensuring the services are still running during the referrals. So having a centralised website focused on social prescribing for people living with Dementia would be brilliant.

Interviewer: Yeah, that's what we feel. So we'll try to pursue this strategy.

Respondent: Sorry, I talk a lot. I'm passionate about my work.

Interviewer: It's amazing.

Respondent: What I think also because some social prescribers might actually not have that experience of working with people with dementia because we’re not specialised. So we’re here for everybody and very often it happens we get referrals in cluster. So this month we may have 10 referrals for patients with a learning disability. But no referrals for patients who are living with dementia. So I think some of our colleagues actually may not be exposed to that and may not be getting that experience. So that's why the centralised website is brilliant for those people who don't have regular contact.

And then because if you don't have regular contact, if you don't practice the skills, if you don't have that process of thought of thinking, oh, this looks like they might be a little bit more forgetful, maybe we should look into it. It doesn't come that easily, so it's always good to have that centralised point of knowledge where we can refer to.

Interviewer: Yeah, I understand that. Let me see if I’ve got any more questions. I think I covered everything that I wanted to ask you. Was there anything final that you wanted to tell me about what we've discussed?

Respondent: No, I think that's all, thank you. I hope it is helpful.

Interviewer: No, this is super helpful and very, very insightful, [anonymised]. What I wanted to ask you is actually, we're actively recruiting social prescribing link workers for interviews. So if you know anybody in your service, it seems that you guys are doing massive work and great work. So I would be happy for you to pass them my email address, because we're really looking for people, to hear from them. Because we want this to be co-designed based on your needs. So it's quite important that we hear what you have to say.

So have a think, if you can think of any colleague, just refer them to me and I'll be happy to. Thank you so much. So I'll let you go. Have a lovely weekend.

Respondent: You too. Have a great weekend.

Interviewer: You too, take care.

Respondent: Bye.

Interviewer: Bye.

[End of recorded material 00:39:41]

**SP04**

[Start of recorded material at 00:00:00]

Interviewer:  The general question, if you could tell me a bit what your role is and particularly in terms of whether you support people living with dementia specifically.

Respondent: Yeah, so if you remember, I'm a little bit unusual for this because I am a social prescriber. I've been doing it three, four years, but particularly at the moment, I'm leading the project with [anonymised] for looking more at systems and processes and connecting services up. So at the moment, I'm not directly supporting people with dementia. But if you me for my normal day job, so in a year's time, if I go back to that, that's what I will be doing. I'll be taking referrals on my case load with patients, yeah.

Interviewer: It's interesting, your current role, because maybe we can explore a bit around those organisational issues and connectedness between different services. But just to start with, maybe what I can ask you is, first off, how do people get referred to social prescribing within your practice? Because obviously there's a huge diversity out there. And quite importantly, do you feel that people know about social prescribing when they refer to you guys?

Respondent: Yeah, it's been so interesting because, like I said, it's been about three years now and I would say it's just going in the right direction as in people know about it. And it's now being offered as people are starting to look at the GP practice now as not just a GP. I turn up, I just want to see my GP. So I think it's this big education that's out there about the GP isn't necessarily the best person to see you. So it's changing. I think it's an education process.

So at the moment in [anonymised], all referrals have to come through the GP or certainly through – we're a bit wishy washy on this one, through a healthcare professional of some sort. Occasionally, we've linked in with some of the community therapy teams. So some of the OTs that we know who have stumbled across people when they've done a home visit and they've come back to us and said, “Can we refer into you?” And we said, “Yes, you can.”

So it's that kind of, I don’t know community therapy, is that primary care? It is primary, isn't it? I lose track on the primary, secondary, yeah, it's primary care, isn't it? So it is, so it's those healthcare teams that will refer in. So we've not gone down the route of saying, “You can self-refer.” I think that was possibly the model that was thrown out there at the beginning when social prescribing was put on the table, that eventually, maybe five, 10 years’ time when we've got a greater workforce, we can do self-referrals.

But I think if we did self-referrals now, I don't know how we'd manage it. I don't know. I don't know how we'd manage it. So we generally say to people – so say I went to a memory cafe and I was chatting to somebody and she says, “Oh, that sounds amazing, can I be referred to you?” I would probably say, “You need to go back to the GP and ask there, ‘Can I be referred?’” which is a bit long winded. But that's the route we're taking, I guess, just to manage things a little bit.

Interviewer: Yeah, I understand that because the service is still in its developmental stages in a way. It requires that there is a system in place because the resources are not such that allow. So you mentioned a quite interesting phrase about educating people, about the existence of this model and what it could do. So how do you think people are being educated? What do you guys do to educate people?

Because I was talking to another social prescriber the other day, and she was saying that they do some outreach work as well in terms of going actually into the community to some events. Within your practice, what do you guys do to educate people?

Respondent: Yeah, lots of that. I think the whole community engagement element is huge, I think. Yeah, there are some specific events that have been put on that have been amongst the [anonymised] – all the GP practices have put on special workshops in like an afternoon to say, “Come along and meet your social prescribers, meet your clinical pharmacist, meet your first contact physios.”

In other words, all the extra R's roles, because that's what we're part of, aren't we? This additional role thing. Come and meet them. There has been some public events that have been organised from a strategic point of view. But I think as far as just us, as a team, we deliberately have got out to all the community groups. So we've gone to coffee mornings. This is why I keep in touch with this, even though this is not my day job.

So the team, I think there's 10 of them now in [anonymised], they have put together friendship and community calendars. And I think, from memory, there's at least one event per week that they would go to. So it might be a health walk on a Tuesday. And then the following week, it might be an open water swim on a Wednesday. Next week, it might be a coffee morning on a Thursday. So they've tried to put strategic place things. They primarily did it because they were building up friendship groups and to address loneliness and isolation.

But that was kind of in recognition, just to come full circle to what you were saying, kind of in recognition of we need to go out there to find people, to let them know what's available, if that makes sense. Once they've got to the GP, once they’ve got across that GP threshold, they're going to find out about us. Because the GP will tell them, hopefully, although I would just say a quick caveat here. If you looked at a general surgery and say there were five GPs, I can imagine referrals come from maybe two and the other three don't use them. It's so strange. I think, again, come back in 10 years’ time.

Interviewer: It’s interesting.

Respondent: It is so interesting. This shouldn't be recorded, should it really? The more I learn about GPs, the more I realise this tunnel vision for some of them, not all. But social prescribing is new for them and they're thinking, “Why? I'm medical, I sought this out with a load of drugs,” and they do a great job. So they need an education as well as far as to say, “There is another option. Look at your patient holistically. They don't necessarily need to do that route. Is there somebody else in your team that could offer social prescribing?”

So even within the GP surgery, we're talking about a challenge. Sorry, we digress, don't we?

Interviewer: No, this is a very important point, I think. It's part of that cultural background of a medical model that obviously by training, GPs tend to put on people to adopt when they deal with patients.

Respondent: Yeah. It's a clinical approach, isn't it?

Interviewer: It’s a clinical approach, yeah.

Respondent: So there’s me saying it, “It's all fine once they've crossed the threshold,” it isn't because of that. Like I said, there will be some surgeries that I work with and they would say hello to me over a coffee. But I'm thinking, “You've never sent anybody my way. Do you actually know what I do?”

Interviewer: Yeah, what am I doing here?

Respondent: It's all very polite, but actually I'm part of your team. That was a real issue. I haven't been back in the practices particularly for a year. And I would deliberately turn up and do meetings in the surgery to make sure I was physically there. I don't think lockdown helped establishing ourselves as part of the GP practice team. But I do think that still there's a long way to go for the GPs to see us as a team. This is a team, it's not just you. So I did deliberately make sure that some of my appointments were based in GP practices. So I was being seen visually.

Interviewer: And I suppose that this could apply in particular. potentially to patients living with dementia. I suppose because of that medical model, a GPC is a person living with dementia, they immediately think, “Oh, behavioural, psychological symptoms equals prescription of drugs,” as opposed to potentially, medication, exactly. So maybe even more so with this population, there is room for sensitising GPs or changing culture.

Respondent: Just before I pause, just make sure we cover annual dementia review with that in mind because of the work that [anonymised] and I are doing. But with that in mind, we've got a big – I know it's a bit specific to me and [anonymised] at the moment. But I think it's with this in mind that you've just said, when the GP has that contact, who is the best person with somebody with dementia? And again, what's that team approach? How can that be quite holistic? Do you want me to say a bit more about that now or not or am I dancing around?

Interviewer: No, we will forget, so why don't we touch on this now?

Respondent: Yeah, so [anonymised] and I are really keen, and this is me being not medical, but anybody with a long term condition, in theory, has an annual review. And so when I realised this in my journey with dementia patients that they are entitled to this annual dementia review, it was kind of like, well, there's your annual touch point. Because I am finding patient after patient, patient after patient are saying, “I feel abandoned, get the diagnosis, we're cast off, go and live well with this.” And they don't know how to live well.

And when I say to them, “You do know that you're entitled to this annual dementia review,” their eyes go, they just can't believe it. And so [anonymised] and I have been exploring, what does this dementia review look like to give patients that feeling of, “Yes, you've cast me off to say go and live well, but at least I know that once a year I'm going to be back in touch with somebody.” It's like getting back on that hamster wheel, isn't it? I'm okay just now, but I want to know I can get back on.

And so it was saying to GPs, “Are you the best person to do an annual dementia review?” So part of the work that we're going to be doing is exploring a more holistic approach. To say that, what can a social prescribing approach look like for a patient once a year that covers a far more broader range of issues that isn't just the clinical bit? So this is all your community connections, are you coping with your carer coping? Do you know what respite looks like? So it's very much a carer dementia patient double approach, so far more holistically.

And then just leaving a 10 minute appointment slot for the clinical bit, because I know that they'll want to see it, they'll want to speak to somebody about maybe medication or the continence nurse or whatever it is. So it's knowing that we, as social prescribers, can do a longer conversation, a more general conversation, “Have you talked about lasting powers of attorney? Have you talked about the end of life stuff?” Those longer conversations which I think GPs, they've had their training, it's 10 minutes, I need to tick.”

So that's where we're going that absolutely a social prescriber with a little bit of clinical help from another person, it could be the clinical pharmacist, it could be a GP, but having that first contact. So an annual dementia review would look like you'd see the social prescriber for half an hour, 40 minutes. They do holistic stuff, document it on system one, and then set up the final little appointment with the clinician.

Interviewer: So two points about what you just said. Very, very interesting. The first one is, of course, the prerequisite, if you want, for the annual dementia review to be a productive process is for the person to have received a diagnosis, of course, because this is a dementia annual. And this raises all issues around what I've heard from social prescribers, actually some people are referred to them without a diagnosis in place. And they sometimes are the ones who are spotting those signs and referring the person back for an official diagnosis. So that's the first thing I wanted to mention.

And the second one is, it was an important point, do you have anything to say about this first point? It'll come back. Is that your experience?

Respondent: Yeah, that's a good one. That’s interesting. I think personally, I've not had many of those, I don't know. Because I'm thinking, why would they have been referred in the first place? Well, there are numerous reasons why that person could have been referred to a social prescriber in the first place. And maybe, yeah, I don't know. That doesn't sound particularly familiar that we've had people who are just needing that nudge.

Interviewer: The only thing that surprises me about this thing that came up is that because most people are referred to social prescribers through the GP. It's just a bit weird to me that the GP actually didn't spot. I know they have very limited time to see the patient, but it's still a bit weird. Because sometimes it's quite visible. And I also understand potentially the early stage is a bit more difficult to really grasp those little cues and signs.

So maybe that is the case. Maybe it's the case of people who at very early stages of their journey, and they probably slipped that process of diagnostics before they were referred to social prescribers. But this is something that actually didn't occur to you in your experience with social prescribers?

Respondent: No, not particularly. No, I think more I'd be thinking about would be, and I made a note of this about the lifestyle elements that I think social prescribing has got a massive part to play, as we're trying to help people make change, to make life better and to improve their health and it's that lifestyle changes. And so for me, I guess where I'm going on this is, it's not about necessarily spotting that the people are heading towards a diagnosis.

But actually have those messages on much earlier on for you and I, for any of us to say, “What are we doing to prevent a potential diagnosis? So lifestyle as far as your diet and exercise. So where am I going with this one? It’s that shift between, “You might have memory issues, but that could be because you're getting a bit older.” So, I joke to them, I say, “That's me, I've got memory issues and it could be menopausal, it could be old age.” I'm not old age, I'm aging, as we all are.

But it's that kind of, what am I doing to slow that down or to avoid that risk? So it's more about lifestyle. So I'm quite keen to say to people, “Whether you've got a diagnosis or not, whilst you're potentially thinking, have I got a diagnosis? Are you saying I've got an issue? That kind of fear of sending me back to the GP.” Whilst we even think about that, can we look at what's good for good brain health? So, what are you doing lifestyle wise? Sorry.

Interviewer: So, it's a bit of difference between the preventive work, or at least the maintenance of skills that a person has, as opposed to, I can understand the expectation sometimes that comes with having an official diagnosis.

Respondent: Yeah, absolutely.

Interviewer: But my point was just in relation to the annual dementia review and how important it is. So in order to have that, I suppose you have to have a diagnosis.

Respondent: Yes, that's a good point actually, yes, you would do. Yeah, so that's the thing, isn't it? Once you've got the diagnosis, now how do you live well with it?

Interviewer: The other thing, it came back to me, was around the annual dementia review you mentioned. Ideally you'd have a bit more time with your social prescribing link workers so that you have those conversations. And what came to mind to me, there is therefore a central role for social prescribers to actually refer the person to anything really that is not necessarily medical. Because for the medical part, they'd have a conversation with the GP.

But for anything else, say, for example, a need for social care, supporting the house, getting washed, getting meals. Do you see that role for social prescribers to then refer the person to whoever they need?

Respondent: Absolutely. And I was just thinking, the resources that we use loads in [anonymised] is the dementia directory, which I've referred to lots. And I think, what is it now, a 21 page document now, that was something I set up for myself, just literally, Oh yeah, where, where do I refer to social services? What's the phone number for so and so? And who's the person that does technology care? And just literally was dumping everything all on one page.

Then it got bigger and bigger. And then, Oh, where's the memory cafes? So this resource, which I'm really, really happy to share. And I've been pushing it out everywhere saying, “If it works for us in [anonymised], then duplicate, it's only a document, it's nothing fancy.” But because it's got so big, we've then colour coded it into certain sections. So it is about having that resource to almost say to somebody, if I was doing a poor job, well it's not even poor, I think it's still pretty good, as a social prescriber, at the very least you'd be sent this directory to say, “I think this is everything in your local area,” so it could be about social care.

But I'd like to think, and it's got all the general stuff, stuff that you can do if you're sitting in your armchair in [anonymised]’s. Things that you could do if you want to go out, as a carer, there's a whole section for carers. But I'd like to think with the annual dementia review, or even without the annual dementia review, even as social prescribers, my colleagues would be doing the, “I'm going to sit with you and go through that.” Because as we know, people get fed up with being given publications, and if I'm not careful, although I think it's wonderful because I've written it, it's just another booklet. And it's like when people get the diagnosis, “Here's your Alzheimer's guide, off you go and live with it.”

What people often want is someone to walk you through that and to skip to the relevant section. So I might be chatting to you and there's no way you're even going to look at the care side of things just now because you're just not ready for that. But you're really interested in getting out in the community. “Right, let's go to section three. Look at the community stuff and let's talk through that. And how can you get there? Let's look at transport. Let's look at money, etc, etc.” So I think it's that, what have we got that we know about in our local area? In this case, it's all in our folder directory.

And then it's that chatting it through with somebody. And I think that would be what I'd want to do at that dementia review. Have those resources, but to do that, just to work through and say, “What's most important to you today? And it would be different next year when we touch base again.” Yeah, does that answer your question?

Interviewer: It does. It has. First off, it seems like a very great and helpful resource, not only for the person you're having the annual dementia review to learn what's available out there. Because it's essentially, from my understanding, also kind of a repository of all things that locally are available to people. But it also a wonderful resource for the actual social prescriber link worker who might be a bit less experienced in dementia things than you, for example. And they might really need referring to a document which really lists what they could offer to the person.

So I think it's great. I think it's important, ideally, that different services in different localities develop a document like this because obviously, there's huge variance. But I wondered, [anonymised], if you're happy to share that with me, just so that I take a look more in terms of the template, how you've organised it in terms of sections and the colouring. Because I agree with you, it needs to be accessible and not overwhelming. And it seems that you worked in this direction, so that maybe that could be a template for other services, which of course would populate everything with their own local resources.

But I think it's great stuff. And I think that's what's needed, particularly because what I'm seeing is that lots of services get people living with dementia. But they don't actually have that background necessarily in supporting that population. So they're a bit lost and they don't know really what's out there necessarily.

Respondent: Absolutely. No, that’s right. What I would say to you, so you might not be interested in the document, is all the formatting side of it, we haven't done anything with yet. So it's actually not got a good template. So I always say to patients, “At some point between now and the end of my project, I will have to make this look a little bit nicer because at the moment it's just a Word document. But [anonymised] and I have said, at the moment we know the content is really good, but it is just a Word document. And at some point we need to – well I think [anonymised]'s quite keen about getting some students from [anonymised] University involved with it or somebody to look at it and make it look a bit prettier. Because I've got a bit of colour, that's about it but it's not formatted very well at all.

Interviewer: Don't worry. Those are the final touches. I think the concept is really important and the goal of that document. Can I go back to when you were talking about how to educate people and the Outreach work that you guys do? You mentioned lots of activities going on in the community that you participate to. I was wondering if there's any of those who are dementia specific. Any Outreach work that you guys do around dementia groups and all that?

Respondent: Yeah, so I would say, um, there's something called RDAN, which is [anonymised] Dementia Action Network. So because we'd heard about DAN, the Dementia Alliance Network that had been set up for years ago, I stumbled across that one in [anonymised] and thought, what a great idea. Every three months, you're getting everybody round a table that talks about dementia and trying to connect people up against this thing. And we all know what everybody else is doing. Otherwise, if we all work in our silos, it's rubbish.

So I then set up a local one of these action networks. So that's why we've got this [anonymised] Action Network. And there's about 40 or so members now. And I'm just going to quickly grab something to remind me. I'm just looking at some of the members. So thinking about the outreach side, in that group of about 40 members, you've got your classic memory cafe leads. So they're all involved. You've got the libraries, you've got the PPGs from the surgeries. You've got people who are doing dementia friends training, carers awareness training.

I've got people from the borough council there. I've got people from the care homes team, from the country park. Anybody that I stumble across who I think has got a connection to the community and is interested in supporting people living with dementia, I say, “Do you want to be part of this network?” And then we meet four times a year. And we meet in person with cake. Because I think it's really important to forge those relationships. And at those meetings, we are always plugging what is out there, what is out there, what is out there. So I think that would class as outreach. I'm just thinking about how I understand your question correctly.

Interviewer: Yeah.

Respondent: [anonymised] and I at the moment, because of our project, we dictate the agenda. But we do find that we give time for community groups to update what they're doing. And we've got a new green memory cafe that we've set up recently. So it was about sharing that and saying, “Right, we're thinking about doing this. Has anybody got any ideas?” And just working together. And then that goes back to those groups, and then obviously, so for instance, each of those memory cafe leads would take that to their memory cafes, and the carers groups that I'm supporting. They would take that back to their groups. And I personally try and spread myself around each of those groups every now and again.

But yeah, that's time limited really. So I would say, I think it's a funny time. If I wasn't in this project position, I think my 10 colleagues, in answer to your question, they would be going to groups, community engagement groups with people living with dementia. But they’ve left it alone because they know that I'm doing it all. They kind of go, “Oh, anything with dementia, [anonymised] is doing,” which is not really true. So when I'm out of this project, I would say some of that community engagement will be with dementia groups.

Interviewer: But it seems that even at the moment, you're kind of liaising with the key organisations leads so that they can then present the opportunities to their own members. It's cascading that information from top to bottom.

Respondent: It is, yeah.

Interviewer: So another question I wanted to ask you is, once a person living with dementia is referred to social prescribing, how does it practically work? In other words, social prescribing link workers support the person, is it through the phone? Is it face to face? How long? How regularly? Can you give me a bit of pointers around that? Of course it's different, I know.

Respondent: Of course, yeah. So on average, I would say, you literally pick up the phone and say, “I've got a referral.” And you're always gauging whether that person that you're speaking to is the person living with dementia or is it the carer? Actually, let's go back a stage. So sometimes I would look at the referral and depending what the GP or referee has put in there, I have been known to go back and say to the GP, “Would it be easier if you put the referral in the carer's name?” Because you're then kind of scooping both up with one go. Because people living with dementia, as we know, they're going to forget or they're going to get confused.

So to make things much easier, it's about dealing with the couple, if that's appropriate. It's not always appropriate but it's almost easier and I think yes, being a bit selfish here because this is the thing, people say I'm a dementia expert. I'm absolutely not, it's like I'm a fraud. I'm really not. The work that I'm doing with [anonymised] is more about this other system. I've only done a bit of training and I really don't know. I'm not confident, I'm being really honest, with speaking with people who live with dementia. But it's something that I think I get nervous about.

Interviewer: So you think that that rapport building with a caregiver as well facilitates that?

Respondent: Absolutely, yes. And you'd like to be sensitive because it's actually saying to the person living with dementia, “You've still got a voice, just because you've got that diagnosis, I'm not ignoring you.” But I'm just going in with, trying to keep all options on the table, and thinking, if it was appropriate, “I'll speak to both of you.” So I’m kind of guided by the GP a little bit on that one. If they say, “Oh, there's no communication issues. You'll be absolutely fine.” You go with it, go straight to the patient with the dementia. Maybe it's an early diagnosis and they're functioning very well.

But yeah, you'd be picking up the phone, you'd be making that appointment on the phone. It would be interesting to see if this has slightly changed in the year that I've been out. But generally, what I would have been doing is I would probably offer, almost certainly offer a face to face appointment. And it would either be in the surgery, yeah, I think we've generally been encouraged to do that in the surgery, or we're certainly allowed to go and do home visits.

And I would much prefer that. I don't really like doing phone calls. I don't think it feels very good. And let me think what I would do now. I would probably say, “Look, whilst you're waiting to see me,” because I think the target is to speak to them within five days of getting a referral. And we're generally hitting those targets. It's tight, but there's generally not a waiting list.

Interviewer: Yeah, it’s quite tight.

Respondent: Yeah, it’s good. I think we're very proud of what we do in [anonymised]. But then we actually don't see them to talk through what the real issues are, for maybe two weeks. I think that's the target, two or three. So what we've tended to do, and I think this is what the team are doing, is they would send the directory to them, if it's appropriate. Email it to them or pop it in the post and say, “Have a little read through, this is a document that we use and it's very helpful. Have a read through so that when I come and meet with you, you've got an idea of which section we can go through.”

So you're giving them that preparatory stuff right at the very beginning, and then you'd be going to meet with them. And I would say, on average, some, well, one patient I had probably about eight or nine appointments with, he was a quite long term on my case load. And most others, I think would be encouraged to do two or three contacts. Interestingly, so like I said, if I was doing the day job, the team leader is a lady called [anonymised]. She's a lovely lady, very dynamic and very creative, and she would say to us, when referrals start getting really, really busy, we have to change our way of working.

She really doesn't want waiting lists, so she gets us to tweak the way that we're working. So the reason I'm saying this is on a good day when we're not at capacity, we've got time, we would take the referral and we'd really, really be thorough. We would be the one that maybe helps them look at getting a new phone set up because they can't operate the phone that they've got because it's not working.

We will be the one that makes the referral to maybe a care agency and to look at a little bit of respite, or we'd be the one that helps them set up the community transport to get to the memory cafe. But on a bad day, when we're at real capacity, we're encouraged, this is where I think it's going to be interesting for you to hear, we're encouraged very much to go straight to the specialists. And in our area, it's the Alzheimer's Society have been commissioned to provide something called – I'm going to look at it now, they’re called The Dementia Support Service. And in theory, they would do all that.

Interviewer: I see.

Respondent: They would do all that thorough, “Right, OK, have you got this in place? Have you got that in place?” dah, dah, dah. Between you and I, when I heard that service was being commissioned and it was, “Oh, everything is going to be all right now, [anonymised] because we've got this lovely commission service coming up.” So really all our job should be, as social prescribers, is to signpost. You've given me this, and all I'm going to do is I'm going to pass you on. I'm going to pass you on to the specialists and they are called The Dementia Support Service. That sounds great but my nervousness, and again, I wish this wasn't being recorded, my nervousness is, are they going to deliver? Are they in a control? This is my control bit here.

And we've said this as a team, we're good. We know our area. We really know what we know. But does The Dementia Support Service know that? And will they do home visits? Will they do face to face? Will they go above and beyond and know about the seven memory cafes that we know about? Do they know about – and it's having trust. When you signpost somebody on, you've got to trust that that service is good and it's still early days.

Interviewer: Is that a local service?

Respondent: Well, I'd be interested to see if you hear around how much that's happening. It's by the Alzheimer's Society, so obviously, that's national. But in [anonymised], they have commissioned this, I'm just looking at here, the overall term is called the Alzheimer's Society Wellbeing Service. And they've, they've got four offers. One of them is telephone support, which I think that would be a national thing. One of them is to offer CST at the 14 week CST course after someone's had a diagnosis.

They've also been commissioned to do some carers courses in four weeks. And then the final bit is this one that I've been talking about, this Dementia Support Service. And these are meant to be advisors that are in the local area who will do home visits if you need that one to one support. So that sounds good, doesn't it? That sounds good.

Interviewer: It does, it sounds quite holistic. It's important, however, as you're saying, that they really know the local resources and initiatives because that's quite key, isn't it? The person is living in their local environment.

Respondent: Absolutely. Well, this is an example, as soon as I heard that I'd been commissioned two years ago, I thought, “Wow, that's amazing.” Well, maybe three years ago, “Wow, that's amazing.” So I straight away emailed them and said, “Who have you got in the [anonymised] area? I'll share all our resources and we'll get to know them. And then we'll have a great relationship. We'll signpost backwards and forwards. We'll be there for each other.” And I was told there wasn't somebody who covered our specific area. They covered a much, much bigger area. And that's when your alarm bells sound, and I've got to know them a little bit and they seem lovely.

And it's not about personal, it's not about a personality thing, but I'm nervous about any service that has been held up as this amazing, “Oh, in [anonymised], everybody is fine because they get referred to this service.”

Interviewer: So really what you're doing, as you said before, you're only resorting to this service when you're at full capacity, just because you want to be hands on, if possible.

Respondent: Yeah, I think so. I think we try and do both. So I like to think we go above and beyond. So if we did a referral to somebody like the Dementia Support Service, what I would be doing, and I think this is good practice, and I think they the team still do this, is you wouldn't discharge that patient. You would put it in your calendar that maybe four weeks’ time, or whatever time frame is appropriate, four weeks’ time, you go back and you say, “Have they been in touch? I've taken the responsibility to signpost you to this service and refer you.” So we've done the referral.

In fact, yeah, we do the referral. That's the least we can do is do the referral and send a directory, but it's the follow up, isn't it, to say, “Have they been in touch? Have they been good?” We try and get some feedback. Have they done what you need them to do? Have they answered all your questions? In which case, the job’s good one, you don't actually need me. So that's why I'm quite curious as to almost potentially we could be redundant in the dementia world in [anonymised] or [anonymised], because this service is put in place. Is there both?

Interviewer: It depends how it works.

Respondent: Yeah, absolutely. I bet that's different for you, isn't it? That's probably different in each area of people you are speaking to. Have you got a good range of areas?

Interviewer: Yeah, I will say we're covering nationally and so far we've got good representation from, of course, [anonymised]. We've got some people from [anonymised] who have talked to us. I've got lots of contacts in [anonymised], so I've got a couple of people but yeah, that the aim. We're still at the beginning of the study, but that's the hope. I was wondering, [anonymised], if you could think of really anything that comes to your mind that would be really helpful to have on a resource, potentially on a website for social prescribing link workers in terms really of supporting them to deliver better support to patients.

And this might be around information, around links and that's what made me think of your resource that you've developed around having, again, a template on which that informs your approach to the client. Anything really that comes to your mind from your experience that could be helpful.

Respondent: I think, very topically, because [anonymised] and I, we keep half picking this little plate up and spinning it and I think we haven't got time for this. But I would say, right, I'll hold that for a moment. What I would say, and this is why I think it's quite difficult, a challenge for you to do a resource, because I think the biggest thing that is an issue for us is about knowing what happens locally which is why I think it's quite hard for you if you're putting together a national resource.

When I made some notes earlier, I thought, “What is it that would really help us as social prescribers to support patients?” It's actually knowing who is who and what is what and how they link together and give me some numbers and let me know. So I'll give you a simple example. When I know some people have got issues with continence. It's that kind of, “What do I do about that? So is there a continence team? I don't know. Am I allowed to refer social prescribing? Am I allowed to refer or do I have to go back to the GP and bother the GP?” Because it feels like you're bothering the GP to find out, have I got? Can I? Can I access that? Is that which team?

It’s that knowing who's involved with who. I don't know how you can help, because that must be very different. So it's even things like when I first started this project, I spent the first six months just knocking on people's doors going, “Right, you're the MAZ team. Tell me who you link up with. Right, you're the therapeutic intervention team. Who are you? What do you do? Do you talk to them? You're the care homes team with dementia. Do you talk to them?”

And a lot of people don't talk. So I think, unfortunately, I don't know how you answer this one. It's about knowing your local system and who's connected to who within primary care. So put that to one side, I don't know how you answer that one really.

Interviewer: But actually I think, of course on a website whose remit is national, you can't be specific and you can't be local in terms of literally putting down the context that you should be liaising with. But what you can do maybe in terms of a national website or a national resource is give out pointers as to what you just said to me, get to know people, maybe ask that person if the area you're trying to explore is that area. And make sure you're aware of the local resources and maybe in order for you to get to know the resources, do this or that. The kind of overarching strategies to be able to connect to local –

Respondent: Yeah, that's good.

Interviewer: Opportunities, as opposed to the actual links. That's the only way you can really tackle it.

Respondent: Like a checklist, isn't it?

Interviewer: Yeah.

Respondent: Yeah, I do love a good checklist actually. [anonymised] and I were saying we do like that because it is almost, you're just casting your eyes down thinking, “Yeah, who do I need to go and reach out to? And who potentially is involved in this patient's journey whilst they're living well?” So that's some of my frustrations. But one of the things I would say as a resource would be the whole thing about exercise and brain health. And again, this preventative or not even preventative stuff, oh yeah, it is actually, whether you've got a diagnosis or not, it's about what kind of exercise and possibly it's lifestyle stuff.

Diet, I guess, a little bit, but exercise is more the thing. Because [anonymised] and I have just been recently talking about tai chi. And a couple of weeks ago I spoke to somebody who does Nordic walking and we just said, “Oh,” and she knows somebody who's done some research for Parkinson's and Nordic walking. And I just said, “Oh, wouldn't that be interesting to find out? Because I bet Nordic walking with all that kind of co-ordination with hands and legs and your brain, and you're still physically out and you've got the green side of social prescribing, you're outside nature. Oh, that would be really, really good. I'd love to find out more whether we –”

So it's the kind of thing that I would love as a social prescriber to have the knowledge that when I see somebody, I look online for the evidence base in the literature to have the knowledge to say for example, these are the top five exercises that are proven to be good for good brain health. So I think that side of things would be really helpful. At the moment, I'm just kind of making it up. I've heard Nordic walking is good for Parkinson's, well OK. And I'm thinking, then [anonymised] was saying, she thinks there has been some evidence around tai chi.

What else? We were meeting only yesterday and she said something about gardening, tai chi and something else. But it's that knowledge, and then being able to find very simple packs for people to actually access. So it might be that you need to find your local tai chi group. But also, addressing some of the stuff that can be done at home.

So I know that in my directory, there is a section about if you're stuck at home. So I think I must have started this when it was lockdown and saying to people, “If you’re housebound, even without a video, you can still send off for, I think it's the physios have done a little booklet, the get up and go booklets or something that you can do.” Here's your top six exercises, so you've got a pack at home. So it's knowing what exercises will be good for people to be doing to keep brain active and physiologically active as well.

Interviewer: I think it's good to hear this and I think probably we're on the right track in the sense that what we're trying to do for the website is to gather the evidence, the scientific evidence, to show that there is a benefit in, say, physical exercise. And also as a result of presenting that evidence, of course, in an accessible format for people, then also signpost website users to links in order to find those local opportunities to do some of these activities.

Let me give you an example. What I'll do with it, I'll share my screen very quickly because I want to show you something. Do you see this?

Respondent: Yes.

Interviewer: So what we're doing is we're gathering all the different links that actually enable social prescribers, say, for example, to identify, say here, for example, gardening clubs for people living with dementia. The way you do this is hyperlinks, when you click on them, the page will open up. And hopefully, this will link you to a local search engine where the person can just input, the social prescriber can just input the postcode where the person lives in and it will show all the local opportunities.

Do you think that's something that could work? So presenting the evidence and then putting these links where the workers can literally find by postcode?

Respondent: Absolutely.

Interviewer: OK.

Respondent: Yeah, no, that's great. Yeah. And next time, don't show me the Alzheimer's Society one though.

Interviewer: No, I won’t.

Respondent: Because they don't actually have them all on there. This is what I mean. Take that bit off the recording, please.

Interviewer: Yes, don't worry. We won't transcribe that one. There was one about physical activity, actually. This is a study about the benefits of physical activity in dementia. This one's a good one. But there was also, where did I find one on –dementia friendly? No, that's about film screenings, daycare, heart and dementia. There was one about physical activity. But anyway, it's usually –

Respondent: There's a gymnastic one there, there's a gymnastic one, isn't there? I just saw on there.

Interviewer: Did you see it?

Respondent: Yeah.

Interviewer: There’s loads, but essentially I just wanted to show you what we're trying to do here. And it seems that it's in line with what you were saying to me with the idea.

Respondent: Yeah, that’s right, yeah. I think it is. I like the idea, like you said, the two things, the evidence base, which gives you the confidence that we know what we're talking about here and then, because as you know, you probably picked this up, social prescribers are, is it jack of all trades, master of none? So it's that feeling of, “Oh, I know a little bit of this, a little bit of that, a little bit of that,” but we have to then go and go to specialists.

Interviewer: Yeah, it's trying to fight that impostor’s syndrome that sometimes people have because they need to know everything about anything. So it's overwhelming. But no, I –

Respondent: But that's when the relationship is important with your patient, isn't it? And I think, I hope this is the case, that the people that who are social prescribers are quite approachable and it's very much, “I don't know what that is for you, but I'll go and find it out with you and we'll do some research together.” You actually become, you're quite human. You're standing alongside somebody, you're not imposing something like a GP might do with a white coat on. It's that kind of, we're partnering together here and helping you improve your wellbeing.

Interviewer: Yeah, it is that also equal relationship and gives the idea that the power is not in the clinical side, it's just sharing that power in terms of deciding what the care package is.

Respondent: Yeah, it's OK to say, “I don't know the answer, but I'll find out with you.” Yeah.

Interviewer: OK, [anonymised]. I don't want to take any more of your time. This was extremely helpful. No, really, it was. What I'll wrap up with is, again, if you have any colleagues, you mentioned [anonymised] before, if they're happy to talk with me, this will be really helpful. Because what we're trying to do, again, is co-design something that's helpful for you guys, as opposed to what we think should be it.

So, anyone, just give them my email address, we’re actively recruiting, so it would be really helpful.

Respondent: That's good.

Interviewer: And I guess I'll just let you go through your wonderful weekend.

Respondent: Before you go, I want to ask a quick question. So one of the things I did just note, I thought I'd ask you about this. So one of the things that, again, I'll be interested if you can even comment on this even now. But the model that might be happening in certain places, whether there are people who are – what we're trying to say? So after this project is finished, I'm quite intrigued as to what [anonymised] and I will pull together as our learning. And whether we think going forward, whether we have specific social prescribers who have a specialism or whether, and I know that that happens in little areas.

I've heard in one place in [anonymised] that they have an over 65s group and they have an under 65s group. And I don't know if you've got anything you can add to that. Because I do wonder, [anonymised] is a bit nervous about what ideas I'm coming up with. She said, “You’ll come back saying everything needs to be done by the social prescriber. They can do all these annual dementia reviews.” And I did say to her, “Look, whatever we do find, I hope there will be a workforce to go with it.”

But what could be the case is at the end of this project, [anonymised] and I say, “Actually, it would be really good to have a dementia specific social prescriber.” And some people might run a mile from that because they like the variety in the caseload. They actually like the fact that they'll get an 18 year old one day and then a 90 year old another day. But I'm just intrigued, have you found that there are different models again that some are finding specialisms or not?

Interviewer: What I can tell you from the interviews I've done so far, that the specialism is already happening somehow, although informally. So there’s services that do not have this system in place where you have, again, that dedicated team or worker to support specific populations. However, what happens is usually based on experience, people by default somehow share the word. So it's already happening, [anonymised], that's what I'm saying.

Maybe there is room to actually standardise this, because I think specialism is actually good. Because you have that knowledge, you have that experience, and I've touched a bit today in our interview. But it's something that comes up over and over, people really have basic training on dementia, for example, if any. So it's down to the personal interest and experience. And actually, you've got people who also have an interest in a specific area. I've talked to people who have an interest in dementia because they just had lived experience of a grandmother or whatever. So actually, specialism, I think, if possible, it's the way to go.

Of course, it's resource intensive because it requires training. It requires a wider workforce.

Respondent: Recruitment, yeah.

Interviewer: But it's already happening, that’s what I can tell.

Respondent: That’s helpful to know. Yeah, that doesn't surprise me. And I think we're lucky we've got a team of 10. So even within the 10, I'm watching some of these WhatsApp messages fly by when people are saying, “Oh, there's a lovely old one for you, [anonymised]. Do you want to take the old one?” Or whatever it is, and you get to know within your team who's good and who isn't.

Interviewer: Probably at the beginning it’s resource intensive. But maybe in the long run, it actually saves resources, it saves time. So it's maybe an investment that will pay back. That's what I'm saying.

Respondent: Yeah, I’m intrigued, good.

Interviewer: It's just some food for thought.

Respondent: Yeah. No, that's good. Thank you. I did wonder if that would be happening. That's great.

Interviewer: Yeah, it is.

Respondent: Well, you have a good weekend too, and I hope you get your bathroom back.

Interviewer: And I hope you get your guest out.

Respondent: Yeah, well, she's gone. She has gone which is lovely. So it just feels like you can breathe now.

Interviewer: Yeah. So enjoy your place then.

Respondent: Great to see you, [anonymised]. Thank you for today. Bye now.

Interviewer: Thank you. Bye bye.

[End of recorded material 00:51:46]

**SP05**

[Start of recorded material at 00:00:00]

Interviewer: Do you want to give me a bit of background about what you do in your service and maybe also how the service operates in terms of social prescribing?

Respondent: Yeah, of course. So I'm part of the [anonymised] social prescribing network, the [anonymised] Borough of [anonymised]. We have about 20 or so social prescribers across the borough. But I work for a PCN that is made of four practices. There are two of us working there. I've been working there for almost four years. How it works is we get our referrals from anyone, any staff member from the practice. So that's from reception all the way up to the clinicians. And they send that in and then we contact the patients and we go from there.

Interviewer: You were saying you get referrals from receptionists as well. Does it mean that the person can self-refer?

Respondent: Yeah, so the person can self-refer. We also have a self-referral portal on the website that is in development. But yeah, they can just call the reception team and just say, “I need some support from the social prescribers,” or “I have this social issue, this lifestyle issue.” And the reception will send it straight to us instead of going for a GP appointment, which wouldn't be the most appropriate for some of those situations.

Interviewer: Do you think that the system of skipping the GP appointment works in terms of saving time or do you think it would still be best to have that process in place? What's your take on this?

Respondent: So I think it works very well for people that are not coming with any source of medical issue. So it works really well to skip that. If we do find out that they, I don't know, they mention something like a shortness of breath or headaches that are not going away, then we can actually send them back for a GP appointment. But it works really well also to reduce the amount of pressure on the GPs. But they'll come our way anyway, eventually.

Interviewer: Because at the end of the day, I think one of the goals of social prescribing was indeed to relieve that pressure on GPs. So it kind of fits into the picture. I was asking this question because what came up from other interviews is that actually when it comes to dementia, because sometimes the GP, again, are not seeing the person directly, but you guys are first. Sometimes the person comes to you without a diagnosis. And actually, sometimes it's you guys spotting those signs and referring the person back for a diagnosis. Is that what happens?

Respondent: Yes, I've seen that happen as well a couple of times. Most of the time, if I can spot it or their caregiver is saying, “Actually, I have some really, really key concerns about their memory,” the GPs are more than happy to do the referral. So we, in our borough, we can't refer into the Memory Service, the memory clinic.

So if I was to contact the GP and let them know the situation, the GP would normally just do the referral straight away into the memory clinic. And then if the patient themselves or their caregiver wants to see the GP as well, more than happy for them to have an appointment. But it is more thorough for them to go to the memory clinic anyway because they will get that full health check on top of addressing the concerns they have around the memory.

Interviewer: Yeah, I see. So if this might happen, because that seems to be the case in terms of you guys spotting the signs of potential cognitive impairment or memory issues. Do you think that currently the social prescribing link workforce is prepared or aware of how to spot these signs? Maybe think of a situation where you don't have necessarily a caregiver who comes to you with already issues. Do you think people would be able to spot those signs? How prepared are they?

Respondent: So, actually, I would say no. It's very hard, especially when people reach a certain stage of their life to know what normal forgetfulness or normal changes in behaviour are that come with age, and what is actually a dementia concern. We're not particularly well trained to spot that.

Interviewer: Have you ever encountered instances where there was a bit of a resistance if you approached the subject?

Respondent: Yes, I have, one lady in particular, but hers I think was quite complex. I wasn't sure because she was also reaching menopausal age. So the brain fog comes with that as well. So I did say, “Do you want to speak to the GP about this just to see? Have any of your family members mentioned that there's been a change?” So she wasn't happy at all with the conversation, but she was happier with the idea that it could be menopause versus dementia.

I also had another lady who was experiencing forgetfulness, but hers was, she had a family history of dementia. So she was much more open minded to go to the doctor than to be referred to the memory clinic. So I think, yeah, it's a tricky situation.

Interviewer: I suppose it's very individual and it's also down to sometimes culture or your background. I come from [anonymised] and we come from a culture where dementia is a bit of a stigmatised condition in a way. So people are a bit more resistant, maybe compared to people who were born in [anonymised], for example, or come from different cultures. So it's very much down to the individual.

Is it fair to say that if you have, not necessarily you, or your colleagues, if do have that lack of confidence in understanding really what's going on, your default thing is to go back to the GP and have an appointment with them?

Respondent: Always, yeah, always which of course, then does add pressure onto them. But we're able to give a little bit more context as to why we feel this way, what the concerns are for us, what we might be missing. So yeah, we would always, always go back to the doctors.

Interviewer: That's great. So it seems from what you said to me that you did have some experience in supporting people living with dementia or suspected, if you want, cases of people with memory issues. What's your overall experience been with them?

Respondent: So do you know what I've found? People at the mild to moderate stage of dementia have actually been quite easy to support. People at the advanced stage have been probably my most complex, complex clients. At the mild to moderate stage, they are still quite compos mentis. They know what they like, they know what they'd like to do that's meaningful to them. We can get them into some activities, whether that's because we have a choir in the area as well. We have memory cafes, we have day centres, if that's what they're looking for. But once it gets to the more advanced stage, it becomes quite tricky to even work with them directly at all.

Interviewer: Obviously, the complexity of the situation, once it develops and it progresses. But I was wondering, has it anything to do at all with the rapport that you build with the person which is usually done via the telephone? Is it the case actually, I wanted to ask you, is it mostly phone support?

Respondent: So for us, I think we're one of the people that do quite a 50:50. We have a lot of people come in and we have a lot of people over the phone.

Interviewer: I was asking because lots of people have told me so far that it is greatly encouraged to stick to phone support, remote because it's less resource intensive in a way. So that's why I was asking, but it's refreshing to hear that you guys are in the 50:50 ratio. So what do you think are the complexities then in working with someone at a more advanced stage? What have you found so far?

Respondent: So I think the biggest difficulty I have found is the communication and also, even the basic things like pinning down that they understand consent, that they are consenting to this, can take a lot of repetition and a lot of different ways of going around it. Also, yeah, the communication can be a big barrier. You might be having the same conversations again. And sometimes they don't even remember me.

Interviewer: Yeah, it happens all the time.

Respondent: All the time. They won't remember me. So we're just going through the same, almost interventions, over and over again. So that's probably been the most difficult. I found that managing their social issues, so if they're needing support with finances, if they're needing support with housing, when they're in an advanced level of dementia, it's quite hard to make those changes. Again, you would need their consent and you would need someone to support them through the entire process. And that just makes it so much more complicated, yeah.

Interviewer: Yeah. I was wondering, and this is maybe a controversial one, but do you think there is room or there is benefits to be achieved from this specific population from social prescribing? I mean the more advanced one.

Respondent: The more advanced, I don't know. The reason I would say that is because working with – my experience anyway, working with people with advanced dementia, it really is a tricky line between social prescribing and almost support worker. It's not the same in that you can't just prescribe them with the social support.

You can't even signpost them to things. It is much more of a hand holding process. So, I don't know if they actually would benefit the most from it. They do benefit from the activities if we're able to identify them or able to identify the avenues of support that they need, but the process is much longer and much more complicated. So, they would benefit from more intensive support, I would say, than social prescribing.

Interviewer: Yes, things like, for example, social care, personal care, that sort of stuff.

Respondent: Yeah, which we do a lot of with people with advanced dementia. It's a lot of getting social care involved, making sure they have a social worker. Then also, bringing on top of that the importance of them being involved in their community, even if they have some mobility difficulties or some communication difficulties. There are still things that they enjoy doing that will make them feel fulfilled.

Interviewer: So is it fair to say that with these specific more advanced cases, it's more a work of referring them to other sources rather than you guys really working with them in kind of social?

Respondent: Yeah, definitely. I think we're just not the best place to support them. So we almost try and find people that will pick up the baton from what we've identified that they need. And then it's a lot more of referring and almost advocating as well for why they need that support.

Interviewer: That's really interesting. It makes me really think whether the word social prescribing is really encompassing what you guys do. Because all this work around signposting and finding resources that are not necessarily about social needs, right, could be around care. So it doesn't really give justice to all the work that you do, because social prescribing makes me think, obviously, we find a social activity or somebody.

Respondent: Yeah.

Interviewer: Yeah, right.

Respondent: Exactly, but it's so broad, which can also make it a bit more complicated. Because there's almost nothing that someone can come with you in that you can’t do or you can't find them. It's absolutely anything that is non-medical that we deal with, yeah.

Interviewer: It's like you work with a white canvas and literally they can come to you with any kind of need.

Respondent: Yeah.

Interviewer: Well, how does that. make you feel in terms of supporting specific populations? Because again, you don't have a specialty, I suppose.

Respondent: No, we don't have a specialty. I love it, to be honest, because I like to see a range of different people. I like to be able to increase my knowledge in a range of different areas. So for me, it works quite well. But I can imagine for others that almost the lack of specificity in the role would be challenging on its own.

Interviewer: Yeah. I suppose it boils down to whether you're the kind of person that likes to be challenged and likes to get out of their comfort zone, which is your case, and other people are a bit more structured in their approach. So yeah, I've heard cases where people feel a bit overwhelmed when they're not 100% confident that they can support a specific population, a specific group.

So when you approach a client, say this client has specific needs, we can talk about dementia, but regardless, and you haven't worked potentially with that population group. How do you go about learning stuff, in general?

Respondent: So, it always starts with a research basis, and I would normally do this once I've got the referral in, because I'll get a broad idea from whoever is referring what it is that they need. So I will start to search, it normally always starts online, I want to find the charities that are involved or the statutory services that might be able to support with this. And then it will normally be a phone call or I will be an email. And in some cases, when I need a lot of information, I would even ask if they would have a meeting with my entire social prescribing team because we've never heard of this service and it's so great, and I think all of us need to know.

So that's always how it starts. And depending on how the conversations go, obviously the learning can go in one direction or it can go in another, depending on what they need. Sometimes I've even been to – like if I'm looking for a social group, I have been there before just to make sure I can find out where it is, when it happens. If I can't get in touch with places, which often happens with the religious groups, like I can't get in touch with the mosque on the phone or the church, I'll just go there.

I just show my face and find out what actually is happening and I found that that's the most useful way. Yeah, so that's normally how I do it.

Interviewer: So you find a face to face approach with certain communities is the way to potentially gain a bit of trust in the process, I suppose.

Respondent: Yeah, absolutely. I think it's to build trust, to build that link as well. Because we are link workers, we're meant to be that link between primary care and the community or primary care voluntary organisations, primary care statutory services. So getting your face known, getting people to understand what it is that you're doing, and they might see you more often if you're having to bring one of your clients there.

So it's just good to build that link and to build that network almost. I think that's part of our role as well, but I know it's different for everyone across different services.

Interviewer: And I suppose, making that first step into going in the community to specific services is an investment in time because obviously you build that relationship. And then when you get new clients who might have similar needs, you can just go back to the service.

Respondent: Yeah, exactly.

Interviewer: Is it difficult for you to learn about specific services in the community, what's going on in the community and how do you do that? How do you find out really?

Respondent: Yeah, it is difficult to keep on top of things and the reason it is, is because not everything is very well promoted. So You won't find out unless you go and look at places in person to check for yourself, then you'll be like, “Oh wow, they've got this whole community fair on that no-one's heard about.” None of the community knows, none of the residents know, none of the staff know. So it can be quite difficult and really the only way to get through it is just to continue trying to build that knowledge.

Even in areas that I feel quite confident with, I still want to keep on top of the communication with people, make sure I'm on their mailing list to know what's happening and just getting in touch at intervals to see if there's anything I've missed. Is there anything new happening that I could promote to some of my clients? Is it that some of them might find it useful? That's normally how I go about it, but it is hard to keep on top of what's actually happening.

Interviewer: Yeah, it's really a lot and people I've spoken to, because we're interviewing people from all over the UK, right, and that's a huge diversity in services. But one thing that's emerging throughout is that local services have developed a kind of directories, books almost, that they share within the staff where they include information again on what's going on in the community. Is this something that you guys also have or how do you share learnings within your group?

Respondent: Yeah, so we use elemental software for our social prescribing, and on there we have a huge directory of all of the services. We also have a shared file between the social prescribers where we have all of the services, what they do or what are the demographics that they support. On top of that, like I said, all of us will find different services or different people. If we think that they're really useful, we will have a team meeting with our manager to speak about the service, what they do, how we can refer in. We do that regularly.

And then also our social prescribing service has a quarterly coffee morning with all of the community services and statutory services. So that everyone in the borough as much as possible, of course, some people miss it, can know what each other are doing and how best to support residents for whatever needs. So it's not just us, we want to include everyone.

Interviewer: Yes. I suppose it's also a way for the actual services and organisations in the community to learn about social prescribing because what I've learned is that lots of people actually do not know what it is, what it can do. Does it happen with people who are actually referred to you as well, your patients?

Respondent: All the time. All the time, they don't know what it is. They confuse us with social workers or they confuse us with case workers or they'll think if you're helping with benefits, they think you're a benefits advisor. If you're helping with housing, that you're a housing advisor. But part of our process is whenever we pick up the referral, the first thing we do is explain what social prescribing is, what we can do and what we can't do which really stops or tries to limit the confusion around our role.

Interviewer: Yeah, I suppose it's particularly challenged with, again, people who might have dementia or cognitive impairment.

Respondent: Definitely.

Interviewer: How do you, or how would you, go about explaining, trying and making sure that they do understand what's available for them?

Respondent: So in those cases, it's more about repetition. So we're likely going to have to go through the scope of my practice in every single session that we will have, which is not a problem for me. If they ask for something, I can tell them how far I can take them. So if they are asking for a social worker, “I can get you in touch with the service that can get you a social worker, and then we'll go from there.” Or if they're asking for, I don't know, housing support, “I can get you to an appointment. That's as far as I can take you.”

And most of them, everyone actually is very receptive to that because you're not just saying, “No, I can't help you. This is nothing. this is completely out of my role.” It's more that, “I can help you up to a certain point. And then this is for this person or this service or this organisation to help you with further.” Or, “I can't help you with this but I know someone who can.”

Interviewer: Yeah. I suppose it places extra time you have to dedicate to the patient. And my question is, what's the typical amount of time that you spend with a person once they are referred to you? How often do you see them? How long for do you support them? What's the general thing with that?

Respondent: So it really differs, of course, on the person's needs. I think the practices that I work for are really – the side that I work with [anonymised] is the most deprived side of the borough. So they're quite keen on social prescribing and the social area of support. They're very, very flexible. So my appointments can last anything from people that are very, very empowered and able to do it themselves. The appointment can be 15 minutes.

But I've also had appointments that go all the way up to two hours which are normally the face to face ones. Face to face appointments always take me longer. And especially the more complex they are, as you can imagine, with a cognitive impairment, that appointment is likely to be a minimum of an hour anyway. That's the time that I would allocate to that person to come in and go through the initial needs that they present with. And then going through what kind of solutions or what can we do to address them. That will take an hour on its own. And people with cognitive impairments will be face to face always, just so it's easier for them.

Interviewer: So there's that flexibility in terms of your service to be able for you to dedicate extra time given the particular characteristics of this population. It's not like they tell you it has to be done on the phone, you have this amount of time. It's pretty flexible.

Respondent: Yeah, they let us book our own diaries and we have an occupational therapist, mental health occupational therapist, in our particular group of practices. So I do go out, for people that really need it, I will go out on home visits for her, which is not very usual for social prescribing. But we can, we do have the scope to do that, yeah.

Interviewer: It's great to hear. And again, as you say, it's not very common. I've even heard stories where actually, patients being referred to social prescribing are being passed on to Alzheimer's Society who does work on their behalf, just to release a bit of pressure on the system which is raising lots of issues among the social prescribers. Because obviously, they're saying, “Alzheimer's Society is great, but it's a big national organisation. Will they know the things going on in the actual local community to be able to support people?” But that's just beyond the point.

I just wanted to say that it's great to hear that you guys have that flexibility to really support people which is great. I just wanted to ask you, because you say you're very much working with any client population that comes to you. I was wondering if over time, as the service develops within your practice, are you noticing that people are specialising into specific populations? So that when somebody is referred to you who, for example, has dementia, it is one of you specifically that tends to support that person or is that not happening?

Respondent: So there's only two of us and at the moment that's not happening. No, we don't really have specialties but I do see that developing across other social prescribing services that some people have developed a specialty. But I think in our area, the needs are so complex that we both just have to have a pretty good foundation. So we don't have that, no.

Interviewer: And a good point that you're making, I think is that sometimes it's not a patient, a client does not necessarily belong to one group. There might be an intersection of, so particularly when it comes to specific areas, like the one you work in, you might have people who are living with dementia and who are from a specific community, say they are from the Muslim community. So their needs overlap and it may be specialism doesn't really have a place when there's this complex presentation of different –

Respondent: I would definitely agree with that. We have such a huge, diverse range of ethnic backgrounds, religious backgrounds, even our language list is huge. So I think, yeah, for us in our particular area, specialising wouldn't really work because everyone is so complex.

Interviewer: And I suppose, how do you meet the challenge of providing a culture appropriate service? Because you mentioned the languages, right, so what if a person doesn't really speak English as their first language? Do you have any pointers that you can give me around maybe cultural appropriateness of your service?

Respondent: Of course. So when it comes to languages, we do have access to interpreters all the time. So if they're coming in for face to face, we'd normally have the interpreter on the phone. Although we can book them to come in, it's just much easier to just have them on the phone on loudspeaker which works quite well, particularly with people with dementia. I've noticed it as it progresses, they tend to regress into speaking one language, even though they used to speak two or three or four, they'll speak the language that's most comfortable for them.

So we have the interpreter on the phone. In terms of cultural, specific organisations, we do have a few in the community. So we have a big population of Jewish residents. We have a big population of Muslim residents. We have a big population of Hindu residents, and of course, non-denominational residents as well. So we have specific charities that we know can help people with specific needs. Also, on top of that, we have the actual religious institutions. We have the churches, the mosques, the temples and the synagogues as well that we can approach who do such amazing work and don't promote their services very well, if I'm honest.

So that's what we do to try and keep things culturally specific. And we're also aware of the cultural needs, the things that maybe a Muslim family would provide for someone with dementia will be quite different actually to maybe the things that a British family will provide. Typically, what we see is the Muslim families or just families from ethnic minorities tend to have more support in place and often will still be living with their family.

We find that the British families, the older person or the person with dementia, will tend to be living on their own or with their partner and will probably need more statutory service support. The difference is that we see a lot between the cultures.

Interviewer: So based on what you were saying, and that's what I've seen as well in my experience with dementia. Do you find that generally speaking that the people that are most referred to you as a service are from non-ethnic minority groups? Because they would have more support in place and maybe even a hesitancy to approach primary care services and so on.

Respondent: Yeah. Definitely. Yes, we do. We would see, in general, we would see more people that are British or English descent needing more complex or more layered support. Whereas their counterparts in ethnic minority groups, it will probably be the caregiver approaching, first of all. And then it will be specific things like, I don't know, “They're very bored, we need something for them to do.” And then it would be more about letting them know about the day centres and they'll pick it up and run off with that and get them in into the support they need.

Or it would be, “Oh I go out to work from this hour to this hour, is there someone that can come and support them?” And maybe they don't need any personal care support, they just need someone to sit, we'd find them a sitting service maybe to help. So the needs are completely different across the populations, yeah.

Interviewer: It sounds as if somehow they're less complex in a way, or maybe you are less burdened by the complexity of the needs. Because some of the support needs, it seems to be already in place in a way.

Respondent: Yeah. I would say that their complexity, when you actually dive into it, is very much the same. It's just the amount of support that they would have in place. Definitely our ethnic minority residents and clients will always, I've never met one that doesn't have family around them, to support them. They will always have someone or they will have their religious group, so whether it's the church that comes in, the synagogue that comes in, the mosque that comes in, to bring them food or just to provide them a befriending service.

They will have a different kind of support provided, whereas we find with the English or the non-ethnic minority communities, they might be a little bit more sparse in terms of the support. They often have their spouse, but maybe children that live far away or out of the country. So it will be probably more of a combination of social care and along with social prescribing that would be needed.

Interviewer: I wonder if you end up noticing that with the ethnic minority groups, you end up having to provide support somehow to the caregiver as well, because they take so much on, on themselves. I wonder if you've noticed any difference in terms of, well, at some point maybe the caregiver almost becomes a client in a way, a patient in a way.

Respondent: Yeah, definitely seen that. A lot of times, there's almost this sense of duty that these family members take on and they don't think they also understand how much work it can be to support someone that is obviously losing their cognitive function. And yeah, they often end up becoming clients, or while speaking to them, I can identify that they need some help, in which case we would connect them to carers organisations, caring support groups just to have a carers needs assessment to see what could be put in place to relieve the pressure from them. Because if they burn out, then there's also a lack of support for them and for the person with the dementia.

Interviewer: When you identify these needs for the caregiver, maybe, for example, refer them to care assessment, do they need to be officially referred to you guys through the GP route? Or do that informally, if that makes sense?

Respondent: Yeah, so I would normally do it informally. I wouldn't ask them to go through a whole referral. We would discuss it separately from their issues with whoever they're supporting. And then we would do that referral for them, as long as their consent is there, it's fine. Sometimes it's connecting them to therapy or counselling services as well just to deal with the mental health side of caring for someone as well as the mental health side of maybe losing someone while they're still alive because you're losing the person that you knew, yeah.

Interviewer: Yeah, it’s that bereavement process happening when the person is still with you somehow.

Respondent: Still with you, exactly.

Interviewer: The last question before I ask you the core thing that I wanted to ask you is, do you guys get any dementia training as well? Or is it again down to, as you said before, you guys doing some research around it and there's no formal training around dementia?

Respondent: Yeah, we get dementia training. We're quite lucky in this borough. We've had, I think, two separate dementia trainings. So yeah, that's just to give us an idea of how they will present, what kind of things we need to look for, what are the areas of need. And even just to know some of the services that we can think of off the top of our head that might be able to support.

Interviewer: Good. That's very great. So my final question, which is the kind of important thing I wanted to ask you is, thinking about this resource that we're going to be developing, and this is a really open question, what do you think would be really helpful to include in the resource that might support you guys? Maybe you're lucky from what I hear working in the service you're working in. So maybe think about in general social prescribers working with people living with dementia.

Respondent: I think it would be really key to include things about what to expect and how to work with someone with dementia across the different stages of dementia, how to approach someone. Because I think the first time that you meet someone with advanced dementia, if you've never worked with someone like that before, can be quite challenging if you're going in completely blind. So I would say that is quite important. I would also say maybe putting carers resources on there too. Because, because of the complexity, sometimes of patients with dementia, it's easy to overlook the carer completely and just focus on what we can do for this person. So I would say that that's also important to include on there.

Carers often forget about themselves as well. And I would say, of course, the usual thing. So again, the client themselves and the carers can be so wrapped up in their practical needs, that they forget that they used to be someone that had a routine that used to go to work or used to volunteer. So I think it's really important that they have social activities or volunteering activities that are suitable for them to do. That would be really helpful on the list.

I wouldn't write off people with dementia involved in community activities, so making sure they're still connected is really good. Because I found that there's almost a link between the dementia and mental health. So the depression and the anxiety that can come with that and the social isolation as well. So I would think that's quite important.

Interviewer: So you're advocating even potentially for the more advanced stages of dementia to still try and find something that is socially engaging and stimulating.

Respondent: Definitely.

Interviewer: So just going back to what you mentioned, it might be helpful to include something, I suppose, around communicating with people living with dementia, particularly in the initial development of report with them.

Respondent: Yeah, absolutely.

Interviewer: I think you mentioned also, or you hinted to that initial resistance that people living with dementia might have in getting that support.

Respondent: Yeah, absolutely. How to approach the conversation. You don't want people living with dementia to feel that you think that they're stupid because they have a cognitive impairment, which isn't the case. A lot of them retain the same skills. They just need a way to show it or to use it or to put it to use. So yeah, I would say that's quite important. It's a sensitive topic and it's important for people to approach it sensitively.

Interviewer: Yeah, and it's such a different population to work with because, as you said before, it requires an investment of time on your part to really get that conversation and trust going. And the other point you quite crucially mentioned, I think, is around the caregiver. I think both in terms of being aware of the underlying pressure that people supporting people living with dementia have and also potentially including them in the conversations.

Because you said before, there might be stages where it's actually crucial to get accurate information. And that might come from a caregiver, I suppose.

Respondent: Yeah, definitely had that a lot of the times where the client is saying one thing, “They're completely fine,” and then the caregiver will be like, “They leave the front door open whenever they go out.” Yeah, so it's really important to involve the caregiver and also offer the caregiver support. Because dementia doesn't get better, it will only advance so that they're aware of what is out there for them if it gets to a stage that they can't cope.

Interviewer: No, that's great to hear. In terms of how this resource is presented, would you rather go for like a website? I'm thinking about what you said before about researching on the web. Would you rather have, for example, something that's printed out? Because some people have also told me, “I'm always on the run. Sometimes it's easy for me to have things printed so I can read them.” What's your preference in terms of the format?

Respondent: I think people, if their social prescribing service does a lot of home visits, it would be crucial for them to have it on paper so they can flick through. If it's a service like mine where we do most – well, all of our visits are in the practices, I prefer to have it on the computer. But if I was doing a home visit, I would just print it off and take it with me. But I would prefer a website, but everyone is different.

Interviewer: Yeah, I suppose the website gives you that extra benefit of being able to access it wherever you are, like geographic allocation, don't carry stuff with you, and maybe include some printable buttons at the end of each of the sections that we create so that people can do that.

Respondent: Absolutely, yeah. So you can take a page with you if you're going to see someone or if I know I have someone coming in, I would print this specific page for them or their caregiver so they can have a look. That would be really helpful. I think it would be an amazing resource.

Interviewer: So, I think I'm done with my questions. So if you're happy with it, I'm just going to wrap it up. It was extremely helpful. Let me just stop the recording so that I can properly say goodbye.

[End of recorded material 00:39:47]

**SP06**

[Start of recorded material at 00:00:00]

Interviewer: And really the first question is what is your role and what do you do within your service? And also, the second question will be how the service is structured in terms of social prescribing.

Respondent: So the role of social prescribing is to signpost. That's specifically the role. And, so we are supposed to limit the sessions to a maximum of six sessions. Most contact is via phone and we get to understand the needs of the client. And then we go away and we can look at the case and also establish what is a priority. And then we can research different organisations and services that are available. And then we send, we either email or text or verbally let the person know, the client know what is available and what we recommend that we can provide as a signpost.

So we're not there to give advice but we can support to quite a minimal level, because obviously the needs of everyone is different. Every case is different but the signposting is quite structured in regards to what's available. But it's expansive, it's not exhaustive. So if someone else comes to us like today with a resource or an organisation that we could signpost to, then that would be utilized. So it's continuously expanding.

Interviewer: I see. But the main thing about the role of social prescribing, and this is at least the case within your service, is purely to signpost or mostly to signpost people to opportunities in the community.

Respondent: Yeah.

Interviewer: Would you see the role as mostly around social needs of people or is it more of a holistic kind of spectrum of needs that people might have?

Respondent: It is very holistic, yeah, the approach is completely holistic, because most of them are – well, I'm quite new to this service, to social prescribing. My background is secondary mental health care. But the staff that I've spoken with within our team, I think there's about 25 of us, a lot of us have either a psychology background or mental health or social care. So we're a diverse team so that is fundamental, I think, to the holistic approach.

So to establish the needs, someone might say, “I'm having some difficulties at work.” But without understanding the physical health needs, their current mental state, their work environment, there may be something else at the root cause of the problem. So it may not be the job, it's what's going on at home or it could be physical health is affecting their ability to work consistently, to be happy. That may be affecting the mental health and that is ultimately affecting their work life.

So, yeah, everything is always a holistic assessment to find out what's going on.

Interviewer: It makes sense. And I suppose also, an observation to support this kind of approach and argument is that people might have an intersection of different conditions or different circumstances. So take a person living with dementia, they might have comorbidity with health issues or mental health, by all means. So I see why social prescribing is, in all effects, a wider approach than the social element of it. And I often wonder with people I interview whether actually, social prescribing is the right terminology. Because you guys tend to do so much more than just the social.

Respondent: Yeah. I think, when someone asks us what we do, it's easier to say like, “Well, what don't we do?” When you're trying to explain to someone what we do, you're kind of a GP for non-medical issues.

Interviewer: Yeah, and does it raise issues around feeling overwhelmed in approaching a patient or a client with really a blank canvas, completely blank? And how do you feel about what you're required to do in all different areas?

Respondent: Yeah, I think they get through that at recruitment. So I think that the recruitment process is to get people that have worked in difficult and challenging environments, multicultural environments, within multi religious environment. They're just every different – it helps if you've got a lot of experience in different areas. And I think mental health is fundamental because you get an understanding of needs and the challenges that people are facing.

But yeah, I don't know about overwhelming. I find it very interesting because each case, until you make contact with that person, you don't know what you're going to get. So it can be difficult, but the way we work is very structured in the way that we make the initial contact. And that's viewed as like an assessment where we establish the needs, the fundamental needs of the priority. So we're not trying to deal with everything in one go. So on the first assessment, we might find out that there's five different problems, but one is a priority that they need to resolve. And you'll often find that resolving the priority will help the other four.

Because the other four could be a result of the main problem, as having difficulties in other areas of their life. So, yeah, once we signpost and to get the fundamental issue, we'll do that on the first term, like first contact, first meeting. We'll signpost to those organisations that can help address the first problem, the major problem. And then maybe the second or third engagement, we might start to look at the other concerns, maybe one or two of the other concerns might no longer be there because they've dealt with the major problem.

So it's really well structured in our approach so it's not overwhelming for the client.

Interviewer: Yeah. Also, because as you said before, it's time limited to six sessions. And I suppose that somehow also helps because people, especially with very complex needs, might ideally be looking at support, prolonged support. And actually, you get lost in actually prioritising those outcomes when it's not time bound. So that structure, I can understand that it might help. What's the approach with people living with dementia? Do you have any experience so far with this specific group? Any feedback on this very complex population?

Respondent: Yeah, you've nailed it there, like you’re saying how complex it is. Because I know from my background of mental health, I worked in dementia units and worked in a community with people with dementia, and the phases can accelerate very quickly, can't they, the different phases of dementia? The different forms of dementia, the different presentations, and it is very complex, and it changes very quickly and rapidly.

And not only that, as social prescribers, we've got to consider what support network is out there, are they already having carers? Are they receiving care? Do they have family support? Friends support? Are they engaging with anyone at the moment? So it is very complex. But I haven't had a great deal of contact. The main signposting we use is quite specific. So it will be specifically related to dementia services. So, we have a facility part of Age UK. We have a facility that we organise groups for people suffering dementia.

And we're specific to [anonymised], and then we can signpost to wider organisations like, I’m not sure if it’s Mencap or something. I would have to look at our database. But I think those are the challenges is establishing what the needs are for what stage the client is at, at that time.

Interviewer: Yes. Also because it fluctuates very much over time to the extent that one day it might be at a certain stage, the next day, it might actually seem as if it's improving. But by the third stage, it's gone down even more than the first. It's very difficult to be consistent, actually, even in the type of support that these people need. Do you think that within your service, looking forward, there’s going to be a bit of a specialism trend whereby people who have a certain background? You have it in mental health, for example, will support specific patients who are referred to the team because of that expertise? So that you kind of share.

Respondent: Yeah. I would hope so. I would hope so, absolutely, because there's a huge gap in care. So, what I've noticed, so my journey in mental health started in secondary care units, like acute adult units. And then I used to work in a crisis and home treatment team. So that's supporting people in the community, but also seeing people who are discharged from the unit to support them at the early stages of discharge going back into community.

And then I've also worked in A&E for the psych liaison department. I've worked in recovery houses. I've worked in Dementia Unit, I've worked in the Elderly Assessment Unit. And there is, there really is a gap between the two, from secondary to primary care. And I've noticed the staff here at the GP practice, there's a lack of understanding and knowledge of who does what. There’s a need or a need of understanding for what each other do and not only that, what's available. Because what the understanding of mental health needs are in a community is very different in primary care to secondary care.

So there is a huge gap of people working in the community, if they just had a bit of an additional knowledge or specialist training and specialist knowledge. Like you've suggested, for working in dementia or for working with learning difficulties, different severities of learning difficulties and so on, different needs. Then I think the strain would be eased dramatically. Because currently all the patients for all the things that I've just mentioned are coming to GPs. And GPs think, “Right, OK, you need mental health support, right, where do we go?” And there's that gap. Now we’re social prescribing, a lot are getting referred to ourselves and we can signpost to different organisations.

But still, there's a gap within that to be more specialist in. I've suggested already to the practice here, and they said, “It makes sense to have specialist mental health social prescribers.” S you get some from social background who are very understanding of housing needs, financial needs, people who are high functioning and then people that are lesser functioning who have more specific care needs. But the demand is so huge, it's like, where's it going to come from? Who’s going to fund it?

Interviewer: Yeah, of course, yeah. And also what kind of specialism are we looking at? Is it going to be specialism based on specific population groups? Say, for example, dementia, say, for example, culture, relevant approaches, or is it based on conditions? So you mentioned mental health, it could be other conditions. But what I take from you is that you advocate regardless for a more specialist approach to social prescribing.

Respondent: Mm-hmm.

Interviewer: OK.

Respondent: I think that’s the way forward, to better support people and to get through maybe the most difficult periods, the difficult times that they're experiencing. But maybe saying that, as social prescribing grows, hopefully that will materialise. And we are better educated, our training is better, we become more aware. But then as we become more aware of dementia and other mental health difficulties, we'll become more aware of cultural issues.

We're already using quite a lot of interpreters to establish needs and just to be able to communicate with people. So, yeah, there's definitely a case for – I don't know how you would suggest to go about it, really.

Interviewer: I think, as you say, it’s a work in progress. It's not the infancy stages with social prescribing, but it's definitely progressing. And as it evolves, certain specific areas that require specialism or developing will be highlighted, I am sure. I suppose also with time, potentially services will be able to accommodate their approach, depending on different populations. So this morning, just to give you an example, I was talking to another social prescriber. And she was saying to me that with people living with dementia specifically, sometimes it's very difficult to initiate rapport and create that initial trust when the relationship is all over the phone, for example.

So in that case, maybe the services will, in the future, be a bit more flexible to accommodate face to face visits. This is just to give you an example. And actually, I wanted to ask you what's your take on how services might develop, keeping in mind the population living with dementia and its challenges?

Respondent: Yeah, I think, definitely face to face meetings would help with dementia patients because of that rapport and that reassurance, depending on what stage they're at. But it can be a very, very unsettling time for a dementia patient going through those initial stages. There's a lot of frustration and the unknown of what's going on and how to deal with it, who's there to support them. So yeah, I think that's a really strong point. More face to face interaction would be really good, really good.

Interviewer: And this is not the case at the moment, right, within your service?

Respondent: Not the moment. We can't offer that at the moment because we're not doing home visits or anything like that. And our workload already is significant enough that it would be very, very difficult to facilitate appointments and those appointments running on time, just because it's so diverse. Because there's no specialist team. We literally just pick any client up with absolutely any need. So it's such a diverse range to be able to offer consistency. And also, we can do more than six visits or six contacts if it's a particularly complex case.

But the whole point is not to encourage dependency. So our service is really based around encouraging empowerment and, “Right, OK, we're going to signpost you.” So that means that the client is going and resolving their concerns and issues for themselves. Well, obviously, that's not the way to care for someone with dementia because it's going to get harder. So for us, if they're seeking referral and we have six contacts and that they’re declining, then they're going to learn that we're not there for continued support.

So maybe our timescale with dementia patients could be expanded, so we are able to offer that more. And I think more support as well, because we're signposting, I've suggested that if we can offer more support and have an understanding with people. So there's a very closely linked role to ours, which is a mental health and wellbeing coach. So we're quite closely linked. That's more obviously towards the support. So I think in some way we need more of those or if we were trained more, we could see patients for longer in specific ways.

I just think there's an element where there needs to be some middle ground. In a lot of what I'm saying, it's reaffirming that there's gaps within the services. We're accepting clients that need a lot more than we can offer because we’re just signposting. And then you're asking – it depends on the exact person. They might have anxiety concerns that are going to prevent them from picking up the phone and calling someone. They might be quite elderly and not really competent following links and going on to Mind and wherever we're signposting to. And then it's very much up to them whether they pick up on the signposting.

Interviewer: Yeah, but I take your point that because of the specific challenges that some people face, only signposting resources doesn't necessarily mean that they're then going to follow through to what you've signposted. And I feel particularly with people living with dementia, especially those who have less support from families who might live alone, who might potentially, being older people, might be from another country, English might not be their first language. You mentioned before translations, right.

So, these are all added barriers that, as you rightly say, require potential further support than signposting. And I see your point about potentially delegating that extra support to the health and wellbeing coaches and the care co-ordinator.

Respondent: Yeah.

Interviewer: I can see that.

Respondent: Care co-ors are good. But then again, they offer very minimal contact. So, I know care co-ors may be able to only go around maybe once a week, once a fortnight or something. I don't know. That's in mental health. I'm not sure what it's like in – I guess it's the severity of dementia.

Interviewer: Yeah, I wonder, let's make a practical example. Let's say that someone living with dementia is referred to you guys and you say that initially there is an expectation that you don't provide more than six visits, let's call them that way. Say, for example, that this person living with dementia is initially resistant to accepting your support, right. So you make a call, the initial call, they would just hang up or refuse to talk to you. How does that work? Do you keep trying because we know how people living with dementia are, what would be the approach in those cases?

Respondent: Again, I think it's the understanding of the social prescriber, the person who's involved with that client. Personally, we look at the background and the reason for the referral so we can check out the GP notes and look back at the case. But personally, yeah, I would really try and reassure the person, so I would leave a voicemail for them, say, look, we really are reaching out to them. We want to try and support them in any way that we can.

And, yeah, do a few follow ups. I would probably wait till the fifth appointment. I would call them. If they don't pick up, I would say, “Look, I'm going to call you again at such and such a time at that date.” Please can they answer the call? Then I would do that again. I'll probably go five times before I discharge them.

Because they can seek a re-referral at any time once they’re discharged, they can come back to the service anytime they want. So yeah, I would be quite consistent and persistent in trying to engage with a client.

Interviewer: I suppose in many instances, these referrals are requested, I suppose, by a family member or a caregiver. So I wonder whether caregivers, you can actively try to include in these initial conversations, family members as well.

Respondent: Yeah. It can often be the family member or the caregiver that also needs some support. Because they're firmly involved in the care so they have needs as well. So, they might be stuck for things to do.

Interviewer: Yes. What do you do in that case? Do you refer them to the service or do you informally support them or signpost them, how does that work?

Respondent: We would signpost as like a package. So they're part of the care network. So, we would support all of them. I would anyway, because ultimately without supporting their care network, then they have nothing today, do they?

Interviewer: Yeah. So they wouldn't get a formal referral to social prescribing. You would consider that as a package?

Respondent: Yeah, because they would come together. It would be the caregiver that’s seeking the referral initially for the patient. So we wouldn't split them up and try and treat them separately. They would come to come together, so we treat them as one. It's often the case where I get cases already where it's a family. So you might have a child, for example, that's having difficulty and that's affecting the parents. So we try and find things that the parent wants to do, but also how it's going to help both, you know, bring them together.

Interviewer: Yes. And [anonymised], let me ask you, do you think social prescribing is effective with anyone living with dementia? Regardless of the stage, there is always an element that this approach could be beneficial or is it just for specific stages? What's your take on social prescribing effectiveness for dementia people?

Respondent: Yeah, I think it's hugely effective because people who come to our service and come to the GP with a problem literally have no idea of how to go about accessing what's out there, and they have no idea what's out there. And we've got a huge database of organisations and services that we can signpost to. So that that alone has a big impact. But then it's when they’re able to access those organisations effectively, is where it makes an enormous difference.

And you can be talking about so many different, different elements. They might have difficulty doing things. Yeah, they want to go out and do something and they might be aware of activities, but they might not be aware that they can go to the council and apply for a taxi card and get discounted or free transport and then they can go and attend those groups. Or they might get access to support funds or other planners that can help them financially. They might have debts that, “Oh I can't do anything because I've got these debts.”

So, it's enormous, I think it really is. I had no idea when I started this job that all this was available and it's amazing, the resources and the support that's out there for people. It’s quite incredible for so many different challenges and difficulties. So yeah, I really do think it's a huge benefit. Coming from my mental health background in secondary care to this, this is really results. This is gaining a huge amount of results for people. And sometimes they might not think it and they might not be aware, but I think it does. It makes a huge difference.

Because sometimes you get clients that you signpost them and then you never hear from them again. They’re obviously fine. They're going off doing their thing, doing whatever they want to do. And it's like the GP, whoever goes and thanks the GP? You never do, do you? You never go back. You never go back or ring them up and say, “Oh, thanks for that, you really helped.”

Interviewer: The blaming game is always very much there but not the thanking part. No, it's refreshing to hear that your experience has been positive in terms of the benefits across different, groups, social prescribing might have. I was wondering, because you approach such a wide range of conditions in people, say for example, you personally referred a person who is from a specific group you don't know anything or much about. How do you go about and find out what resources are available for that specific group?

Respondent: It would be through our colleagues that we’ll be working with. We've got a group chat, so we're very active on there. So we're constantly being updated and that can come from, “Right, OK, I'm working with a patient, I'm aware of this new service.” And you might just see it in the supermarket or something, so a resource is available or we might get it through the GP practice, from the PCN or myself. Because I still work in mental health. So we can think about things and make them happen.

Like, just for example, I used to work with someone who we used to work together in secondary care in the units doing activities on the wards for mental health patients. That person was a very good artist and runs their own art classes outside of work in the community. So I've put her in touch with our PCN to see if she wants to create groups for clients that can access our classes with them. So it's continuously evolving, it's word of mouth. Maybe we're working with a client and they let us know about something that they attend or that they found beneficial or the carer or so on.

We're continuously expanding the services and ways of how we find out about different things as well. We're very keen to explore ways of finding out about things as well.

Interviewer: Yes. So it's both kind of a team sharing effort, but it's also from clients. It's really from word of mouth as well. Do you happen sometimes to do some research on the web about groups or things going on?

Respondent: I haven't personally, but I know others do. I’ve only been here for two and a half months or something like that. But there's other people that have been here longer, management, that kind of thing. And we have people within the organisation that are looking for services and continuously find out.

Interviewer: Yeah, OK.

Respondent: Yeah, we've got people, I wouldn't know what their job titles are, but yeah, they work in the office over at Age UK that continuously are being aware of different services and groups that has been set up. Like someone might get some, an organisation might get a new batch of funding. So we're notified of that, “Right, OK. They've got intake at the moment.” One organisation might be on hold because they're overwhelmed. I don't know if you know, but up in Mind at the moment have like a 12 month waiting list. So we can hear about other services that can have a faster intake. So we have people looking out for things constantly.

Interviewer: And I suppose my last question for you because I'm aware of time is, thinking about this potential website that we're going to be developing with resources to support a social prescriber working in dementia. What do you think off the top of your head might be some of the things you'd find helpful to be included in this website? What would be helpful for you?

Respondent: I think the most helpful things is keeping it clear and easy to navigate, very, very clear and easy. So what is most sought after for people is easily reached, sort of tabs like community groups, support, understanding. I think giving people the ability to gain understanding of what's going on for the client and the different stages. But if it’s complex, I think that's why it's important to keep it simple, what you're trying to achieve and what’s available.

Interviewer: So mostly it's around some sort of sections that would raise awareness around dementia, what it is, how it presents and all the complexities. But also potentially another section with the actual resources that people can use.

Respondent: Yeah. Yeah. And also I think rolling out, quite soon I think they're going to roll out a self-referral. So you won't have to go through GPs. So I think in that respect, maybe that might be happening at the time, you might have the website then. That would be amazing because then the focus could be on the self-referral and then the main like bullet points of what's there.

So access to finances, access to groups, access to activities, what the main points that people want to access are. And understanding and support. I think the terminology you use is very, very important. And as soon as people see understanding, support, you're kind of asking questions. And if you ask a question, people will inquire about it. But if the focus is on finances and bills, no-one's going to want to know. No-one's going to ever read that, are they? If it's all about difficulty, no-one wants to know. But if it's about, “Right, OK, support, we can help you. Fun, enjoyment.” That's what I learned from mental health is it's crucial, what I learned about mental health was that the approach at the moment is still very old. And it's very much about everyone's talking about CBT and it's CBT is from like the 60s. And it's about finding out about your problems and understanding your problems and then, “Right, OK, we figured it out now.” But it's not about what we're going to do in the future. It's, “Right, OK, we've figured out what the problem is. Learn from that.”

And I want to be like, “No, we've done with our problems. Let's move forward and let's make our lives positive and be empowered in what's going on and enjoyment.” And that’s fundamental, I think, to mental health is moving forward. Because if you just always looking at the past and thinking of the past, you're never going to get out of it. So we want to move on from that and enjoy, especially with difficulties like dementia, the reality is about enjoying what you've got now.

And I think fundamentally, if we focus on positives and enjoyment and moving forward, then people will more likely engage in that.

Interviewer: It's about positive psychology. It's about the importance of language and underlying messages that really get the people on-board. Because at the end of the day, that's what you’re trying to do. So no, that's a crucial point. And I thank you for that. Because it's important to embed that positivity and optimism on a website, I think, if you want people to engage with it. So I've taken enough of your precious time. So what I'll do is I'll thank you for your time and I wish you all the best.

Respondent: Sure. No, thank you. And if you want to catch up or if you want anymore information at any point and you want to collaborate on anything, anytime.

Interviewer: Yes, interestingly, I'm about to put a research proposal bid for funding for social prescribing with the LGBT community. So I might come back to you at some point around that.

Respondent: Yeah, absolutely. Anything at all. Let me know because I'm very keen, I like what you're doing and my view is very much to progress and move forward and improve. It's very much needed, isn't it? So, if I can help in any way, let me know.

Interviewer: Thank you so much, Respondent. I appreciate that.

Respondent: Great meeting you.

Interviewer: Take care. Bye.

Respondent: Bye.

[End of recorded material 00:40:25]

**SP07**

[Start of recorded material at 00:00:00]

Interviewer: Background about what you do, if you've got experience in dementia, in what kind of capacity you support or have lived experience with dementia, just really to get a bit of background info on what you do.

Respondent: Yeah. So I work in public health, I work in the older adults team, so that's 50 plus. Although older adults are generally conceptualised as 65 plus, the years between 50 and 65 are years where frailty can get embedded if people are really sedentary. So getting to people a little bit earlier can be really, really beneficial for the later years of life, allow more time spent living in good health. All of this, I'm sure you already know.

So my lived experience of dementia is very limited. My aunty has dementia but she lives in [anonymised], I live here. We weren't close. I know that she now just recently jumped off her bed, has a broken hip. I know the prognosis there, I understand it, so it's not great. She has a very slow acting type of dementia. It's been going on for about 10 years. So no real understanding of actually the challenges of living with someone or caring for someone with dementia.

Within my work, I've been working in the young adults team since December, so nine months, maybe. And I have not been working on dementia all that time. I kind of got brought into it during the absence of my team leader, to stand in for him on the dementia steering group and dementia action group for the city. So we've just released a new dementia strategy for [anonymised] which I wasn't really part of.

I came in right at the last stages of that, but I played a big part in organising Dementia Awareness Week, discussing the kind of things that we're doing, raising awareness of dementia. I've rewritten about half of the web pages, web pages that will soon be released for [anonymised] for the BCC on Dementia and design their layout. We're still in the process of just finishing that off, part of the reason why I'm really interested in your web pages.

And we're kind of doing it the other way around, probably due to limited staffing and time and that we're probably going to release them and then get feedback and then adjust them. Although, I do take the way you're doing it is a better way of doing it. I'm currently working. It's very, very new as in the last week, to try and harmonise dementia with frailty and falls and end of life in compassionate settings. Because if I were to draw a Venn diagram in your mind, you would see that there’s definitely overlap there.

And the areas where the overlap happens are where you can get the best value for money when you're trying to do interventions and hopefully reaching the most people where it's an awful lot easier to work in silos which is problematic, of course.

And we've got a dementia pathway for the city, but there's no data on how it's actually utilised. So I think I was just successful in just before this actual meeting, in pushing for case studies where you follow people through the current pathway and work out why they went left and why they didn't go right. And what they missed out on and what works for them and what didn't work for them or who accesses which parts of the pathway and what intersectional characteristics they have. Whether it's ethnicity or sexuality or gender or genders, anything. Or health literacy, for example, to understand how it's being utilised and by whom.

Because we have an underutilisation of those pathways in the city. We want to change that but before we change it, we need to know, I think, in my opinion, how they're being utilised currently to look for the problems to inform the changes.

Interviewer: Yes. I am completely with you on this one. In fact, you're probably aware of the methodology of a realist evaluation.

Respondent: A friend of mine is using this actually in her PhD.

Interviewer: Yeah. It's brilliant. I've used it in my previous project, not this one. But essentially, it's very helpful when you want to see what's working for whom and under which circumstances. So if you want to untangle really why there is issues or interruptions in service use based on certain demographics. You mentioned gender, I'm completely with you on that, particularly with sexual minorities. I do research in that area, so I've got a bit of familiarity, particularly when it comes to older cohorts and dementia as well.

But just to say, yeah, a realist evaluation could be interesting. I'm veering off. But yeah, take a look on what's out there. There's some decent work. It's kind of new in a way, so not lots of literature has been published. But I think it's apt to what you're looking for probably. It's interesting you mentioned the dementia pathway in your area, in the [anonymised] area. I was wondering on your experience whether social prescribing fits somewhere within this dementia pathway is something that people are aware of? Is this something that people are utilising as a service? What's the current status in that area?

Respondent: OK, so I also work on social prescribing, but not specifically around dementia and not specifically from an older adult's perspective. And I'm more trying to help link up some of the services around social prescribing. I don't know the current statistics, but I have a feeling that the knowledge around social prescribing is like 20% of the patient population.

And of those that have heard of it, being able to define what it means, even people in public health can't define what it means. It was my job to understand it and then present it to my colleagues, which I'm about to do. I got a little bit carried away and I'm involved in other aspects of it. There is a clear link between, in my opinion, between social prescribing and dementia where it comes to getting people together, chair based exercises, dementia and frailty, clearly a link there. Having an area where the carers can also interact and the people with dementia can be taken off to do those chair based exercises to give a small break and to swap.

Because people who care for those with dementia, those people who have dementia, they can benefit from learning from each other as well. I went to a dementia group. It wasn't run by a social prescribing link worker. It was run by what we call a community network support officer which is essentially, a social prescriber. A different title outside of the NHS, GP, PCN footprints. But it was a dementia group. It was run at, I think it was [anonymised] Football Club.

So it was supported by them and by donations from the football fans, and gave them space to do different activities and to socialise. I think, especially at the beginning, if you get an early diagnosis, which can be quite difficult because some people are resistant to the notion that they or their parents have dementia. Or they'll put up the symptoms of dementia off to something else, then it can definitely help.

I believe it could help navigate the system by bringing different people together to exchange stories and information. I think that is how I would envisage social prescribing working. But that's not exactly co-produced and I've not had experience with the systems. So there may be other ways social prescribing could benefit people with or around dementia.

Interviewer: Yeah. You've said some quite interesting things. I'll try to remember a couple of points. The first one you mentioned was the resistance in potentially getting a diagnosis, which I think is actually quite key. As you rightly said, to make sure that from the start you've got that system in place but also knowledge and awareness. And one thing that I want to ask you, maybe as a follow up question later on, is living in a high density, diverse area such as [anonymised], how important it is in terms of culture sensitive approaches to be able to support the population of people living with dementia and caregivers within social prescribing, obviously?

And then you also refer to dual, in a way, pathways to access social prescribing services which is the traditional route through the NHS and referral from the GP. But also you mentioned an alternative non-primary care based route. Are those both in place in your area? How does the referral to social prescribing from the second route, because the first I'm familiar with, work?

Respondent: So there are 10 localities in [anonymised], no, there are 10 constituencies in [anonymised], need to use the correct language. There’s five localities, north, south, east, west and central, and there's 10 constituencies based upon political boundaries. Each constituency has one community network support officer. So we're talking about a 100,000-ish people per constituency. So you can see the issue here. It's been a successful initiative. There was a desire to double the number. Funding was difficult to come by, it hasn't happened yet. I don't know where the conversation is at the moment.

What happens is these people are generally quite knowledgeable about the area they're working in. They get to choose where they want to work. So for example, I know one lady works in a food bank, she works for [anonymised] Football Club, she works in the local library. So they will set themselves up there and they will see people that come to them. If in that place, they don't have the appropriate footfall, or what they think is enough footfall, they can go somewhere else and try a different place. So these places should be, if you're going to do it, what I would consider to be intelligently, have easy to access from public transport, have no issues with access for people who have disabilities or other issues. Have generally high footfall, and in my opinion, anything that's a community hub, such as social workers, such as housing, such as DWP, Department of Work and Pensions would also be a really good place to base yourself.

Food banks clearly see a lot of people as well. So you're looking at those people who are potentially suffering from inequalities within a system. So that's how they operate and then they will give advice to anyone on anything that they can.

Interviewer: So, say a person accesses these hotspots, and forgive me for the inaccurate language, and they are found to have social needs unmet. Or not necessarily social needs, because as you say, social prescribing is really a bit of everything. So they have a need, so once they are identified as having a need, what's the referral to social prescribing? Is the person they're directly providing or signposting the services that are relevant to those needs for the person?

Respondent: So I don't interact deeply enough, but a lot of the needs, the social needs are quite frequently on housing and benefits for obvious reasons. So I know this lady, in particular, has gone away, researched the case, phoned other agencies, talked to other people, and then gone back and fed back that information. She runs a dementia café, so clearly she can recruit people to that. And she knows her area, so she's embedded within what we call the neighbourhood network scheme. Do you know this? You were nodding.

Interviewer: No, but I get the rational and I love that. I'll tell you why, but please continue.

Respondent: So we have a neighbour network scheme. It's funded by adult social care. There were 10 of them within the city, one for each locality. So she's linked in with that. They have a register of all of the civil society or the voluntary BCSE, I think it is, organisations. And they have a website for that so that she can also refer if somebody has a specific interest to an organisation as well. Does she go and hold people's hands where they need that additional level of help? I'm not sure.

There is only one person per area. My guess is it's a little bit more signposting. I can find out but I don't know. I imagine in some cases that may happen, but in more complex cases, maybe it's passed to a social worker once it goes past a certain threshold.

Interviewer: Yes, I think, [anonymised], regardless, the notion of having these places in the community that really ensure that those that do not access primary health care services to get referred to social prescribing, for the most various reasons, and we mentioned some of those, is really important. That's why I was interested in knowing that dual pathway to accessing social prescribing. Because from interviews that I've done in other localities, what came up is that obviously not everybody goes to their GP.

And again, here comes up the issue of culture and background, and you mentioned some people do not even want to hear about dementia and all that. So this is a great idea to have a system in place. Of course, it needs developing, as you rightly say, but to make sure that those people do not fall behind in a way. And maybe particularly, in very diverse areas, it's even more important which probably brings me back to that question about cultural sensitivity.

One thing that came up in my interviews is that there isn't preparedness on the part of social prescribing link workers, generally speaking of course, there's variants, in addressing specific needs of specific communities. I'm thinking LGBTQ plus communities, for example, is just one, South Asian community, Another classic example. So what is your take on that? What's the current status on what could be done to improve anything?

Respondent: I don't really have enough information to talk with any authority. So I'm going to talk quite generally. I know within the NHS, certain people are employed because they speak multiple languages, so they can serve South Asian populations. I was in [anonymised] talking to them physically face to face. And I know they have people who can speak multiple languages there. So clearly, this is something that is being thought about.

Something that, I wouldn't say I'm researching it, I'm looking into it because it's interesting and it falls under the dementia and communicating about health issues is the use of metaphor and similes in health language. And I think this is more significant when there's a significant divide in health knowledge between two participants. Because if you have someone with very low health knowledge, they can't use that kind of medicalised language. And I think metaphor and similes are almost certainly a human construct, not a specific linguistic group construct. So it probably exists in all languages, whether you're talking in English to someone whose second language is English, or whether you're talking Urdu to someone who's using Urdu as a first language.

So you have this area where people may describe their symptoms using these ways of speaking. And that, I think, links into specific metaphors with specific communities where translation is one level but then you also have the additional barrier of how you describe symptoms when you're not part of that biomedical model of health.

So you have that. Some areas in the city have greater diversity than others, [anonymised] is what's called a superdiverse city. Although interestingly, it seems that we only refer to superdiversity in terms of ethnicity and not anything else. So it's a very reductive way of using diversity. So, I think from other work I've done, if we're looking at ethnicity and nothing else, people respond better and open up to those who share their same characteristics.

That would definitely be recognised in some places, but given that, I believe the DES contract, if I'm right, stipulates you only need one social prescribing link worker per PCN. You’re not going to get that representation across the system. So that's obviously going to be an issue. I know as of March this year, 2023, there were 55 full time equivalent social prescribing link workers across the [anonymised] system, so PCNs. What that number is right now, I don't know what the updated number is. Does that help answer your question?

Interviewer: Yeah, it does help. You've raised some really important points. One was when you discussed your side interest on language and metaphors and similes. There is a big issue in communicating diagnosis to people, communicating in all respects effectively with this population. The classic example is that there isn't even a word for dementia in many other languages.

Respondent: Yeah, especially some Asian languages.

Interviewer: Yes, and actually Italian is one of those languages, so if you were to say it –

Respondent: Really? There is in Spanish, dementia.

Respondent: There is. In Italy, dementia is a derogatory term, interestingly. So, the word dementia does not exist. You've got Alzheimer's, you've got the different types but you haven't got an umbrella term. But just to say that sometimes it might be unexpected and the issue of communicating within these different groups is important, particularly when it comes to dementia where there is lots of initial resistance to accepting care. Both on the part of the person, for obvious reasons, cognitive impairment, being unfamiliar with a stranger coming to you or phoning to you for social prescribing. But also, as you said, on the part of caregivers sometimes given different circumstances.

So the issue of communication is certainly prominent. And in fact, in Forward with Dementia, the other website, we devolved a huge area, well, huge, a section of the website to communication, on how to properly communicate with people living with dementia, different strategies. I don't know if you've ever heard about using white lies, which is sometimes another way of getting the initial co-operation from the person.

There's a whole sheet on the Alzheimer's Society webpage about how to ethically use white lies. But also around how to communicate sensitively with different communities. So we also have a section on that. And from what we've heard, it's really applicable to the challenges that social prescribers are experiencing, given the fact that, as you say, the numbers are limited. You can't, at the moment have, specialism because people I've talked to are supporting all sorts of communities. And even more importantly, people at the intersection of different communities.

So, I wanted to ask you about the notion of specialism, what you think, is that the way forward to ensure that cultural appropriate services are provided? Or is it just too difficult in the current services?

Respondent: I'm going to backtrack slightly and just raise one point so you can capture it. Maybe it's useful, maybe it's not .And it's the use of dementia compared to the use of dementias, where dementia is a mass noun that captures all those different types under it. And dementias becomes a countable noun that emphasises that there are multiple types. And then the use of metaphor and simile based upon the old concept of dementia as a single entity where it could be reductive and reduce the actual complexity and understanding. And maybe not be fit for purpose within this particular landscape.

Interviewer: Yeah, very interesting.

Respondent: Yeah, I think of that as well. Anyway, when it comes to specialisation, I agree with you. Social prescribing is not on its feet yet, at least not in [anonymised]. There are some places where there were really good systems. Generally, they're smaller, like places with one or two PCNs, for example. [anonymised] is quite a large, complex system, [anonymised]. I know in London, there are areas in London that have the balance of social prescribing systems and others that don't really have it. We don't have that. We're not there yet. We're trying to organise it now and it's kind of started, from my experience, over the last three months where larger partners within the system have more clout, more influence starting to get involved.

I'm trying to help a collaborative called Flourish in West [anonymised] do some work. But it's really been driven by them, not being driven by public health. So specialisation is something that I think we need. But when you get into discussion of specialisation, you get into the discussion of continual professional development, CPD which that at the moment depends on a third party providers. And you have third party providers in the system because a GP surgery can't hire easily a social prescriber. Because if they work one day a week in five GP surgeries, they get destroyed on tax on their wages.

So the PCN hires them from a third party provider. And the PCN doesn't have oversight of the training mechanisms, the continual professional development mechanisms within the third party provider. So you end up with differences in the same system. So then you have the safeguarding as well of the link worker, of the people that he or she sees and of the organisations that they refer to, in addition to the funder prescriber, not the prescription model which further complicates things.

I obviously think that specialisation would be fantastic. At the moment it doesn't seem very realistic with the continuing reduction in funding. Because there are so many ways you can specialise people. I think the best way would probably be children and young people, working age adults and older adults and do your specialisations along those lines. Children and young people clearly need a completely different system and cannot just copy across what's happening in adults. I think the best work there probably happens in schools along with the school nurse.

But it's something I've looked into a little bit, but it's not something, again, that I'm an authority on at all. Does that help?

Interviewer: Yes, it does help. And, of course, I'm interested in hearing your personal views based on your experience. So don't worry too much on the accuracy of what you're reporting. It's really interesting to see what you think, given your line of work as well. And I agree, specialism at the moment is not on the horizon of things, given the complexity and also the stretched resources that everybody is working with it.

It is interesting to me that there's that a bit more fully developed, and I'm thinking, you mentioned London. And I actually had conversations with people in [anonymised], where they have a fairly good model, and they are increasingly observing that social prescribers start to specialise in some client groups. So that when the referral comes through primary care, because that's primary care, the social prescribing work team shares the referral based on the prescriber's background and experience and preference as well and all that, hence my question on specialism.

But it is a challenge. So, I'm just asking people to see what they think which brings me to my next question. Maybe if specialism at the moment is not visible, there is actually room for a website for people to access so that they can get some, at least, knowledge on potential areas that they should be trained on when it comes to people living with dementia. So my question is, this is a general thing, what would you think it would be helpful to include on a website targeting the workforce when they need to support people living with dementia in terms of maybe content? Anything that comes into your mind based on experience.

Respondent: I guess, you said workforce, so I'm going to focus on that. The NHS website has, I think, lots and lots of training modules for social prescribers. They have a lot of information on alcohol. I haven't gone into the dementia area of it. But I'm going to assume they have training modules on dementia, if you have access to the NHS website. I know I do, but maybe not everybody does. I don't know how broad that access is. When it comes to what they would need training on, then I'm not going to be able to nuance on this greatly, but understanding symptoms initially, what it requires to be a carer and how difficult that can be. Understanding personality changes that may come with dementia, understanding where you can get support from, I think, is very important, whether that is financial support, emotional support, other care groups.

I'm just trying to remember what I wrote on all the dementia web pages. And somewhere to increase people's health literacy so they can understand what the type of dementia they or the person they're caring for has and what it means. Time spans, what the prognosis is if you train people in what to expect, it can help them to process it and give them a timeline to work to.

The timeline probably won't be correct in 100% of the cases but it's better than nothing, I think. So, we have new dementia strategies. I think it's preventing well, supporting well, there's six strands. There's preventing well, supporting well, I forgot the other one, is it caring well? There's dying well, there's a few different ones. I’ve broken up into the different stages and I can't regurgitate them unfortunately. So because I'm a part of the system and therefore affected by my own system's thinking, I'd probably organise it around each of those. Because it represents a natural stage in the process of dementia.

Interviewer: Yes, what you just mentioned in terms of the topic areas that might be potentially relevant for social prescribing link workers is in line, that's really interesting, with what already have on [anonymised] for home care workers and social care workers. Things you mentioned around a bit of awareness to different types of dementia and to recognise the symptoms from the get go in terms of support from the carer, in terms of what we discussed around communication, supporting the person. So, what I'll actually do, if you don't mind quickly, I'll share my screen.

Respondent: I’ll put it in the chat so you’ve got those different sections.

Interviewer: That's very helpful. I just want to show you really quickly [anonymised]. So you go in the sections for, say, healthcare professionals. The list of different topic areas that we have here is sort of in line with what you said. It's about reading the symptoms and giving a diagnosis and each section by the way has got a list of other articles. But in terms of the structure and the topic areas, I think it's not too far from what you said. There's also explaining dementia and helping the client come to terms with it.

Then it's also about symptoms and changes. And here, I guess, that’s the section you mentioned about prognosis and all that different types of dementia as well. And then it's about support for both physical mental health and wellbeing, and then also thinking about the future. So everything from lasting power of attorney to all that sort of stuff. So I'm wondering, and maybe I can turn this question to you, if actually potentially integrating this website with a section for social prescribing link workers might be a way forward thinking about the structure that I've sort of shown you.

I'm just thinking about whether actually a brand new site is needed at all because it's about dementia could be integrated into this one.

Respondent: I think my initial thoughts are two-fold. One is you're moving into the area of specialisation with trying to get social prescribing workers to get on-board and understand this. And two, is there a responsibility, like a statutory responsibility or legal responsibility? What happens if you get it wrong? I'm not sure who in the system actually has responsibility for doing these things. Quite often, you have admiral nurses or Alzheimer's UK or Age Concern that have people and have helplines dedicated to this.

So I think until you get to the point where you have specialised social prescribers, I think you're moving outside of the boundary of what they should do. Because my conceptualisation of a social prescriber is that they link people up with a group or a service. When it's a service, I have housing issues, I have debt issues, whatever it is. When it's a group, the activity is not important, it's the network that they can tap into that is central for the activity.

The activity is like secondary, that's of secondary importance. But that network, the social connections, I think is what is really important for a lot of people. And you give them a group of people who have similar experiences who they can draw upon as a source of knowledge and support. So I think by putting social prescribers into position where they're helping you navigate the system, you really want to have care co-ordinators which are also part of the ours roles, additional role reimbursement. That's my opinion.

Interviewer: Yes, I think it's a great point. In essence, what you're saying is that at the moment, the system envisions the role of social prescribers purely or mostly around signposting to networks. And then it's the responsibility of the person as well as of the network or the service they have access legally as well, to really make sure that the person is well taken care of. So the social prescribers at the moment is this signposting role.

Respondent: Yes and no. I think a good social prescriber does more than signposting. I think they will work with someone to understand them and what they need. Signposting is a basic element of social prescribing. I think a social prescriber should be a little bit more involved because a lot of people have complex needs. And obviously social prescribers can only deal with a certain level of complexity. But it's also that whole element of going with people to their first session of social prescribers, going to people who host sessions to see if that they're happy with the sessions that they do. And that’s a safeguarding issue.

So signposting is important, but I think social prescribers should be more involved than that which also means not overloading them.

Interviewer: Yes, but particularly with a population group, like people living with dementia, just signposting will not do the job, particularly when you've got resistance or the person lives alone. And there's so many circumstances where I guess, what you said about that, going with the person at least to introduce them to that network is quite key, I feel.

I'm thinking about other interviews and finding it interesting that we share this view. But I've also heard that in many services, because they're quite strained, they are encouraging social prescribing link workers to only work over the phone. Which means that essentially, at the moment, what they're doing is literally signposting, potentially follow up to ask, “Have you been there?” how it’s gone.

But of course, what they're saying to me is that people living with dementia are not always reliable when feedbacking information. So it's quite challenging with this specific group.

Respondent: I think that could also be due to the way the system has been organised where maybe there's a financial incentive to push more referrals through the door in comparison to the quality of each referral. I think you need to decouple those two things. If it's based on throughput, you're going to get phone based call centres of social prescribers. And that's not that beneficial.

Interviewer: Yeah, it's almost like the 111 call centre, something like that. And it's different from the ideal of social prescribing, at least as envisaged in initial NHS documents, etc. But what's interesting about this system is that it's ever evolving. So, it's also quite interesting to see where it's going. It's really going into very different directions based on different localities again, So it was really great to hear from you about this dual pathway that you guys have in [anonymised].

Because potentially, again, I think it's a good model when it comes to certain population groups, particularly dementia and undisturbed communities in general. Because primary care won't do the job alone, I think. In fact lots of primary care services I’ve spoken to, they do outreach work in the community. But again, it's when you have a GP or a representative from primary health care services. Again, there might be that resistance, even if you're doing the outreach community work. So it's great to hear that. I don't want to take too much of your time, [anonymised], so I was wondering if you have any final points that you want to highlight before I wrap it up.

Respondent: I guess we can touch back on specialisation and I haven't looked at the current literature, not the literature and everything. But I still think there is for some a divide between GPs and how they conceptualise social prescribing and how successful social prescribing is in their surgeries. And until that basic element is fixed, specialisation is simply so far from the horizon, that I don't think it is a conversation worth having. I think there's a lot of basic elements that need to be fixed.

I know social prescribing has been interpreted different in different countries. So I know the Netherlands, it is very centrally controlled. So it's a lot more harmonised. And when it comes to the people doing social prescribing, I know that, for example, there's a really good GP surgery called [anonymised] and they have [anonymised] working with them. And the [anonymised] itself does a lot of social prescribing type activities. As a service that helps the GP surgery and also as a standalone service. So there are lots of different players that are quite small in the system that are often invisible which I'm sure you're aware of, but still do good work.

Interviewer: Yeah. In fact, do you happen to have any links to the specific surgery? I was thinking it might be worth talking to them because if the models work in there, it might be interesting to untangle also what is it that is making it so efficient, other than what we've discussed.

Respondent: It's a unique geography. The surgery, [anonymised], who I believe runs a surgery, is a big proponent of the social aspect of health, has been for 21 more years. He has right next door, literally right next door, a folly, which is like an old tower, in which there's an Organisation called [anonymised], and they do a lot of social prescribing, arts based health, arts and health based stuff. And then they have very close by, [anonymised] that's been running for, I'm not really sure, 10, 15 years or so.

So they have a small environment or ecology of groups that are really involved in social health with the anchor of that GP surgery. So that is just one, let's call it blueprint. I'm sure there are other examples where GP surgeries are functioning really well for different reasons. I do have links there. GPs are quite often very busy people, but I can obviously ask if he would like me to share his details.

Interviewer: Yeah, or even one of their prescribers or anyone really from –

Respondent: I don't think they have a social prescribing link worker.

Interviewer: They don’t? Ok.

Respondent: They went for some time without one. I know they weren't particularly pleased with the last one. I don't know if they've got one since, I haven't talked to them for a month or so. I talked to the [anonymised]. So they were operating without a social prescribing link worker, but they still have these two organisations they could refer into. And then they had further connections within the community as well. So you can see how that works without a social prescribing link worker.

Interviewer: Yeah, that's a very interesting model to be honest. And it also goes to show that when there is a certain culture within a surgery, and I'm talking about the GP, you mentioned the social model. As opposed to the traditional medical one, it does make a change. Interestingly, one social prescriber I talked to last week was saying to me that sometimes the GPs need educating from them as opposed to what one would expect potentially. So it's confirmation that sometimes it's also a top down kind of process and culture has an impact within the practice as well. So, do you have any final points or are you happy with what we've discussed?

Respondent: Yes, I think that's interesting.

Interviewer: OK, so what I'll do is I'll stop the recording, so I'm making sure I don't lose the transcript.

[End of recorded material 00:40:34]

**SP08**

[Start of recorded material at 00:00:00]

Interviewer: Question about what you do in your role and how you support people living with dementia, if you do support people living with dementia. And also maybe your link to social prescribing in general, if any.

Respondent: So the role that I do, I work at a charity. It's a county wide charity in [anonymised]. I'm the head of service. And so as part of our wellbeing and care services at the charity is a dementia support group. So we run three of them in the county and have done for many, many years. I've only worked for the charity for about coming up to 18 months. But the charity has been running those groups for a long time. Initially, they were set up to be – they're called care and share.

And they were for somebody to come along with their loved one who has dementia. So sometimes it's husbands and wives, mothers and daughters, that sort of thing. And the idea was that once the person who's got dementia, and it's early onset dementia that we're discussing, if they've got a lot of needs, that this group isn't for them. But they can come along to the group and as long as their loved one is comfortable and happy in the group after a few sessions, the idea was the carer could go during the duration of the session, which is three hours. It's a three hour group meeting. session/activities and things and could get a bit of respite then.

In reality, what has happened, sorry, I'm going off on a tangent really, so you're going to have to pull me back because this is just a bit about our groups. But it doesn't completely answer your question. But what we find is that the carer actually gets quite a lot out of coming to the group because it's people who are facing a similar challenge. So from the other carers, they form friendships and support. And sometimes somebody's found a new something to support people with dementia and they'll share it with the group and, “Oh, I didn't know that.” So they'll go off, so they support each other.

And so those are the groups we run. So we run three of those groups. We have run more but we sometimes struggle to get attendance to keep them going because we are a charity. So at the minute, we've got three. So North, Central and South. Our connection to a social prescribing interestingly, in [anonymised], you may know this. But the way social prescribing is delivered is the council provide the Healthy Lives advisor, so the social prescriber in the GP surgery. And in the voluntary sector, in the charity sector, we provide, and we get funding from the PCNs, to have community development officers.

So when the Healthy Lives advisor is working with the patient and looking for activities to refer them to, it's the community development officers out in the field that are employed by the voluntary sector that are finding those activities and putting them on a database so that the social prescribers can find them. So they work very closely with the social prescribers, but they do a different job.

So in this county, that role's been split so that Healthy Lives advisors can focus on the patient and the activities are populated for them. And the community development officers go out into the community and speak to different organisations or other charities about offering activities. So we have quite a close connection with social prescribing. So again, in this county, those community development officers, there's five of them, but they don't all work for the same charity. So two of them work for us. One of them works for a charity in the north of the county. One of them works for a charity in the south of the county but we work together.

They're like a virtual team, although they've got different employers, different charities employing them, they work together to do that work and support each other. And they run meetings that are called community connector meetings. So it gives organisations an opportunity to come together. I think it's once every other month and talk about services that they're offering so that other people that are at the meeting are aware of it or people come along to find out what's going on in the county because they're supporting people in different ways.

And so those community connector meetings also give the community development officers an opportunity to find out what's going on. And so they might kind of go, “Oh it's interesting that you're doing that because did you know that such and such organisation is doing this?” And trying to make those connections that help the county and help the people in the county.

Interviewer: That's a really interesting model. I've been interviewing people all over the country. And, of course, we know that social prescribing is in the development stage and there's huge diversity out there in terms of how the services are structured. What you're saying to me, [anonymised], seems to point out a good integration between primary care and the social prescribing link workers and what's happening in the community.

Respondent: And the voluntary sector, yeah, that's right, particularly the charity sector. That's what we're quite proud of in [anonymised] is that the voluntary sector are taking on that role. So really being linked to that health care, because so many of the charities in the county are doing preventative care activities for people in the county. And in some cases funded by the local authority, in some cases not. But it's the recognition that actually they have such an important – we have such an important part to play in making that social prescribing project work.

Interviewer: And I guess it's also beneficial in the sense that what my experience is, is that the social prescribers, at the moment, they support so many different populations that they're not necessarily knowledgeable or aware about what's really going on in the community to the full extent, right?

Respondent: Yes, correct.

Interviewer: Whereas you guys, obviously being in the field and being those grassroots organisations, you guys know.

Respondent: Yeah, absolutely, yeah.

Interviewer: So it's smoothing out that process.

Respondent: Yeah, you got it. You got it, [anonymised], you got it in a nutshell.

Interviewer: That's really helpful. So let's talk practically. Let's take a case scenario where a person goes to their GP presenting with some unmet social needs and they have dementia. Maybe they go with their caregiver, because I suppose that's usually what happens. The GP refers the person to the social prescriber. What is then the route in linking with you guys?

Respondent: You mean, how would they find out about our –

Interviewer: Yeah.

Respondent: Well, we have, in the county, we have a directory. So the community development officers will put activities on that directory. And there's also an area of a SharePoint site, an internet site that the council runs that the social prescriber can find that. So if somebody came to them and dementia was the issue that they needed some support with, they could go onto the database and type in dementia. And our activities and also Age UK in the county where I'm at, Age UK run activities and support for dementia. So those things would come up and they could, obviously dependent on location, refer them to the correct activity for them really.

Interviewer: So thinking about the social prescribers, because that's the target population that we're trying to support. Because as I said, they seem to be, I wouldn't say unprepared, but definitely overwhelmed with the work.

Respondent: There's a lot they have to know, yeah.

Interviewer: It’s a lot, yeah. So what do you think in your experience at the moment is necessary or it's important for them to learn about other than obviously that –

Respondent: In terms of dementia?

Interviewer: Yeah, other than the actual activities that are going on, which seem to be the case within your locality. They can learn through this kind of directory, yeah, but other than that, in terms of supporting people living with the condition.

Respondent: Well, it seems to me, and I'm guessing in your research role that you're aware that the Alzheimer's Society do like a dementia awareness course, which is only a couple of hours long. I think it might even be like an hour and a half. And I know that the people that run our dementia groups, they do a whole dementia awareness course. But for a social prescriber or a Healthy Lives advisor, I would have thought like a 90 minute, two hour online course which just takes them through the basics, about how you speak to people who've got dementia and how it affects people, just to take a bit of that mystery out of it because not all link workers have had experience of dementia in their career or families.

And some may have experience of dementia in their families or whatever else, but some might not really. And so they don't really know a lot about it, you can't know everything about everything. But I would have thought it would be useful for them to have, as part of their induction sort of training, a simple course on the basics, as it were, of dementia.

Interviewer: What seems to be the case at the moment is that people do not receive any mandatory training around dementia. So it's mostly down to either personal choice of getting on the training but it's more reactive as opposed to proactive, if that makes sense.

Respondent: It does.

Interviewer: And also, as you rightly pointed out, it raises all sorts of barriers to engaging with the client. You talked about communication, so the classic example is social prescribers saying to me, “I've sometimes closed referrals because on the first contact with the client, they were refusing care.” Because we know it's part of dementia. You have somebody unfamiliar talking over the phone to you because phone contact unfortunately is most of the cases. And you haven't got that awareness about communication issues with the population.

You need to be persistent and you need to – and they don't know. So communication, you touched on a core issue. What would you think in terms of communication, since we touched on this, it’s quite important to learn about dealing with people living with dementia for somebody who's unaware of that?

Respondent: Yes. What do I think about it? How important do I think it is?

Interviewer: Mm-hmm.

Respondent: Yeah, I think it's got to be, isn't it? I guess the people that do that social prescribing role have been taken on because they've got certain skills around communication in general. Because one would hope that's what they're doing. They're spending a lot of time with people, finding information out and relating to people. So I would hope that they have those basic communication skills. But just a bit of information about particular, not just dementia, I guess, speaking to people who have got mental health issues in general and things. That should be something, I guess, that they get some top up training.

And I would think it should be mandatory and it should be part of their induction, you've completed that basic course just to have an understanding, why not? I guess some of the issues they have are they're part time hours, and so they’re very, very busy, they're over-subscribed. But when they're starting out, I would have thought there needs to be some proper induction. So that should be part of it.

Interviewer: Yes, completely agree with you on that one. Other than communication in your wide experience supporting people living with dementia. What are some other points maybe that a good website should point out that are important in supporting these populations?

Respondent: Yeah, what I tend to hear, particularly from the carers, is that when somebody gets a diagnosis of dementia, their loved one, they sometimes get bombarded with information. So they get all sorts of leaflets and bits and bobs about all sorts of stuff. But there's like a process until they're ready to get to that point where they're looking for the support. I guess initially it's the change curve, isn't it? They're going through all those emotions. And I've got a bit of background as a counsellor, so I guess part of the website could have things that sort of say, “It's perfectly normal to be thinking and feeling this, that, and the other relating to dementia,” just to de-stigmatise it, that people don't think that they are going crazy or whatever else, that it's perfectly normal to feel what you're feeling.

So, I don't know, maybe there's areas around how to deal with your emotions, and then there's practical support or things that you could find. And then there are maybe our support which is, I don't know, peer support and that kind of help. Because that's really what it's about, I suppose. They come along, they have tea and cake and they do quizzes and play games. But really what's happening is they're getting some peer support and feeling part of a community.

So I suppose, peer support, maybe there's a section, I don't know, that people can chat to other people, it doesn't even have to be in their area. It could be people all across the country, almost like a bit of a Facebook area where people can put in questions about, “I'm trying to do this and I don't know how to do that,” or, “Where can I find support with this?” might be quite useful.

Interviewer: Yeah, it's interesting because in our previous project that helped us develop Forward with Dementia, when we interviewed lots of carers, what really came out of contact with peers was the crucial information sharing. Interestingly, they were saying to us that most of the information that they learn about in terms of resources, availability out there is learned through conversations with other carers.

Respondent: Yes, that's right.

Interviewer: As opposed to learning from services or what not. And also, the importance of social inclusion in sharing your journey with somebody who can really understand you.

Respondent: That's right.

Interviewer: And all the benefits that that generates So, I suppose what we're understanding here is that potentially the social prescribing link workers should see the client living with dementia but also the caregiver as a couple package in a way, right.

Respondent: Yeah, I would sort of agree. Yes, I would agree with that. We do have in [anonymised] the Admiral Nurses and they are all about the carer, as you know. So, that's being rolled out throughout the county as well. So the carers in particular are getting support. But I would have thought that in a lot of cases the person with dementia is there with, as you said earlier, with their caregiver. I guess they tend not to be on their own. But I suppose it varies, I guess.

Interviewer: So is it important for the social prescribers to view the caregiver as part of those conversations when exploring?

Respondent: Yes, I would have thought so, because the caregiver could be accompanying their loved one to an appointment with the social prescriber. But I would have thought the social prescriber in some way should check in with the carer as well. So, we're talking about Fred and what we can do for Fred and what kind of support there is for Fred. “How are you doing? And what kind of sport are you getting at the minute? And is there anything that you feel you want?” I think there should be a checking in, shouldn't there while they’re there?

I don't know if time allows it. I'm not quite sure how they structure their appointments. But it would seem to me that a good social prescriber would check the whole situation out, wouldn't they?

Interviewer: Yeah. From what I've heard from interviews that I've done so far, it seems that while the person living with dementia is the referred person to the service. However, as we're saying, conversations involving the caregivers are always in place, inevitably. Also because sometimes information provided by the person might not be accurate 100%. So yeah, if we're looking at needs, at what's in place, potentially in terms of social care or benefits, it is always good to involve the carer. Because also of the accuracy of information but also to learn about needs, maybe hidden needs.

So potentially including on the website, some sort of guidance as to how to potentially involve the caregivers in that process of care as well.

Respondent: Yeah, and maybe even a caregiver section of the website, I suppose because there's a support for the person that is experiencing dementia. But maybe there's a page on the website or a section of the website that's specifically for carers in terms of the support that they need. And almost there's a bit of something about self-care as well, “Are you looking after yourself whilst you're busy looking after your loved one?” Because obviously if they burn out, then they're not able to look after anybody really.

Interviewer: Yes. In fact, what you’re saying is so interesting. So what I would like to do, I'll share my screen very quickly with you to show you Forward with Dementia. Can you see it?

Respondent: Yes, oh great.

Interviewer: You were saying, you see different sections here and in fact, if you go into the carers section, you will see under the different headings, there's a section on emotions as well. You touched before.

Respondent: That does look great.

Interviewer: You almost –

Respondent: We're simpatico.

Interviewer: Yeah, absolutely.

Respondent: Well, you guys have obviously done your research to do this. You've had the feedback and the information and you've done it which is great.

Interviewer: Yeah but it reflects what you were saying before that potentially for these new projects as well, it might be important to have different sections, maybe in a caregiver section, showing or illustrating the potential benefits of social prescribing in linking with you guys as well. And making sure that you're interconnected within your own peer community. I suppose that is something that we should think about. But this was just really to show you that again, these conversations have come up in previous research and they seem to be relevant in social prescribing as well.

Can I quickly ask you, do you think that social prescribing really is all about the social or is it more than just the social?

Respondent: That's interesting. I don't really know is the honest answer because I've not really experienced it myself. I have met or spoken to Healthy Lives advisors, I know how the process works. And I've seen some of the stats that come through about what's the main issue that people come or are referred to social prescribing for. But I don't really know what actually happens, I suppose, in the section and what their experiences are themselves.

So, I guess in some cases, some of the referrals might be for more practical things. So it's not all just about social. But I still think it's the right thing, I still think there's an opportunity to pursue non-medical interventions to support people.

Interviewer: So you would say that the terminology that's been used so far in terms of that social emphasises that it's non-medical and anything that's not medical?

Respondent: Yes, and I've used that to describe it to people. I’ve said, “You go to the GP and sometimes you get a prescription, but sometimes you don't need a prescription of medication. You need a social prescription, something else.” And I think people get that.

Interviewer: Yeah, I suppose it's to help people also understand that difference.

Respondent: Yeah, why it exists almost, yeah.

Interviewer: And do you think that, based on your experience, people at the moment know what social prescribing is or that social prescribing exists as a service?

Respondent: Yeah, it’s funny you should say that, [anonymised], because I was just talking to one of my team. We were having one to ones, one of the team members who was a community development officer. And I was saying to her that when I started in this job, so 18 months ago, and I would ask people if I went to a meeting or an event to talk about what we do, and I'd say, “Have you heard of social prescribing?” I'd get a lot of, “No.” But 18 months on, when I ask that question of groups, there's quite a few that, “Yes,” they nod.

So I think personally, that's very anecdotal, I have no proof of that. But I do think that social prescribing is becoming more well known, certainly in this county. Because I know the community development officers do a lot of work going to events and talking about social prescribing and explaining what it is. So I do think it is getting out there. I think people do understand. They've either got a personal experience.

I've come across the odd individual that said, “Oh, I've actually been referred and I've used it.” Or they know somebody who has or somebody in their family has or they're involved in their GP surgeries sometimes as like a patient advice group. And so they're aware that the surgery offers it and what it's about. So I do think it's getting a bit of traction.

Interviewer: I think you touched on a crucial point, which is Outreach work. I think an important strategy to let people know that there is this service is actually go into the community, go to these events and really share the gospel of social prescribing. And I suppose it's actually quite important as well in terms of letting people from underserved communities know that there is this service. Because I suppose, the GP route is the way to access the service at the moment.

Not necessarily all communities are very open to go to their GP because of historical and cultural reasons. So that community route is even more important to let people know.

Respondent: Yes. You can self-refer in this county. I don't know if others –

Interviewer: Oh, can you?

Respondent: Yeah, you can, yeah, on the literature. The literature that we print, and we've got some of the literature here, that the community development officers take out when they do events. It explains about social prescribing and how to access it through your GP surgery. But there's also a number you can ring if you would like to self-refer.

Interviewer: That's really novel. Some other people I've talked to have discussed about a potential for self-referral in the future. But because they've told me that the services at the moment are quite strained, they fear that it might put too much pressure through self-referrals.

Respondent: Yes, well, that's a good point. We don't run the Healthy Lives advisor network. That’s the council that does that. I've never really asked that question. I know that the Healthy Lives advisors, they're booked up and they've got caseloads and they're really quite busy. So I don't know if the local authority regrets that decision to allow self-referrals but at the minute, you can.

Interviewer: It’s happening.

Respondent: Yeah, you can do it.

Interviewer: Do you think there's benefits to self-referral?

Respondent: I think so, yeah, because I think sometimes, depending on the person, so talking to the GP might be great but sometimes if you know, if you understand what social prescribing is and you think, well, actually, I wouldn't mind having a having a chat with somebody about what else I could do, then I think, why not? It's just saves some time, doesn't it?

Interviewer: Yes. And actually, if we're thinking in terms of the original idea of social prescribing which is actually to relieve pressure from GPs, self-referral actually saves time as well.

Respondent: It does, doesn't it? Of course, yeah, of course. It's interesting as well because I think, this is from my counselling work that I've done, a lot of people that come to counselling, so there are people with anxiety and depression and all those things, they seem to all be on antidepressants. Now I'm not against antidepressants because they have a role to play. But it feels a little bit to me, and I'm not having a swipe at the GPs because they're great. But it does feel a little bit like if you go to the GP, the first thing they'll do is give you a prescription for some medication and then might also refer you to the social prescriber.

But everybody ends up with medication, and I think sometimes it's not warranted. Sometimes, people have said, “I've got the prescription. I haven't filled it in. What do you think? I'm not sure I want to go on it.” So I think sometimes if you could bypass that, and maybe that's another area that Healthy Lives Advisors, and hopefully they do, are able to recognise maybe more significant mental health issues that if I came through the self-referral route and they spoke to me and they thought, “Actually, maybe you should see your GP as well.” Presumably that all happens as well.

I'm sure the Healthy Lives advisors refer into the GPS as well if somebody comes self-referral wise. So yeah, it has the potential of just being a really good integrated system as it sort of grows and becomes the way.

Interviewer: Yeah, and it's in line, what you're saying right now, with what I've heard from another service. This was [anonymised], where actually the social prescribing link worker was saying to me, “Sometimes it's about educating the GPs as well,” which was really interesting. Because she was saying they have this medicalised model whereby if you go to them, they will prescribe because that’s the model.

Respondent: They do, that's right.

Interviewer: Yeah, whereas we are adopting that social model of dementia in this case, which potentially does not require medication or, as you'd rightly say, it might be at a later stage. Or it might actually be recognised by the social prescriber, which can then refer the person back to the GP, if required.

Respondent: At the right time, yeah.

Interviewer: So there's education on different sides that needs to be implemented. A very interesting point and it echoes what I have heard already which is great. What else? Is there anything before coming to this interview you thought could be important to discuss or just to report to the study team, given your experience? You touched on mental health as well which is actually a background that I haven't had with other participants. So maybe from that perspective, is there something relevant to dementia and mental health that is important, you think?

Respondent: Well, yeah, I guess it's just when people first get that diagnosis, before perhaps the dementia is necessarily really evident, but you know that you've got dementia, as it were. I'm sure there is need for mental health support, as people go through that change curve, it's not good news, let's face it. So I'm sure there's something that might be necessary there just to help people come to terms with the diagnosis.

And there's also a dementia steering group that we have within the county and the chap who heads that up is a person who's experiencing dementia. He's obviously got a dementia that's quite slowly progressing. So he's able to chair a meeting and does it very well. So I suppose almost there might be a benefit to almost case studies of different people's experience just because it doesn't necessarily mean that in a period of time, you're not going to be able to remember anything. You know what I mean? There's a spectrum, isn't there? There's a spectrum.

And so perhaps there's just a bit of education to help people get their heads around the fact that they might have this diagnosis now and what does that mean for them? So that might be useful. And similarly for the carer, I suppose, so people understand what may or may not happen now that they have their diagnosis.

Interviewer: So are you hinting to the fact that potentially having on the website case studies, examples of specific case studies, might actually show that diversity and potentially even reduce the expectations that dementia is a set journey.

Respondent: That's right.

Interviewer: There's variants to it.

Respondent: That's right. Yes, that's right because that's been that experience for that individual. And there's another lady in the county who runs a support group, a dementia support group but for professional people. So the one that we run is a little bit more community homespun. for want of a better word, like I say, husbands and wives of a certain age, mothers and daughters, that sort of thing. Whereas the one that this woman runs is for professional people. So people have had a diagnosis of dementia, may still be working or when they were working that they were doing roles in a certain area.

And she also is able to run that group. And despite having a diagnosis of dementia, she manages the group and she gets speakers to come in and they meet monthly. And so again, that's a slightly different version of the disease that maybe people are when they get the diagnosis. And it's not like to say, it's not great news, I don't mean to diminish it. But there are varying journeys that you could possibly be taking. And it might be just interesting and help them understand it if they understand people’s specific journeys that aren't all of doom and gloom.

Interviewer: Yeah. And again, it goes back, I guess, also to reflecting the diversity in terms of cultures as well. Because obviously, different cultures experience dementia and access to services and support in very different ways. So I guess it's important to show that diversity on any resource. Do you think that in terms of mental health, because you mentioned that for all dementia support and services, you guys are there to compile these lists and directories that the social prescribers can use. When it comes to mental health, do you think that a social prescriber can access such helpful resources as well in terms of looking at what's out there?

Respondent: Yeah, they can in this county because those sorts of activities are also on the directory. So there's various charities and organisations in the county that support people's mental health and they will be there on the directory as well.

Interviewer: And I suppose within the system, without going too much into the specifics, but I suppose you can search based on postcode, right, so that you know?

Respondent: Yeah, absolutely, yeah. You can search as well, this is getting into the nitty gritty, but the people that have put it together, these community development officers, they have made it searchable. You can put depression in and it will come up with things. If it's dementia support that you're looking for, then by all means type in dementia support. But if it's somebody that's experiencing anxiety, you type in anxiety and your postcode, where you are.

And it will come up with all sorts of different organisations that do all sorts of different things. It depends on what you're looking for then but you can then scan through and find what you want.

Interviewer: That seems to be a really great model to share with other localities as well. Do you think once the person is signposted to an activity, a resource, that's the first step? Are there any steps then to actually engage with that support for this specific population?

Respondent: Yes, I think so. Yeah, because I do get people that call up, they see a leaflet or maybe they find out about it through social prescribing about the groups. And so they ring me up and I speak to them and I tell them about them and they go, “That's really interesting. That sounds like what we want.” And I say, “Why don't you try? Come to a session and see if it's for you.” And they never turn up and we don't hear from them again.

And it's always a sensitive thing. Sometimes after, I don't know, a few weeks of not hearing from them, I might ring them or email them and say, “Oh, hi, just wondering if everything's OK, if you needed any more information.” But I guess for whatever reason, I don't know. I remember one couple in particular, it was the husband who made contact and his wife had dementia. And I think he was quite keen to come to the group because I think he was at that point where he wanted that peer support. But I don't know, because I never spoke to her, I haven't met them. But for whatever reason, she didn't really want to go to the group.

So as a result, they didn't go to the group, but they haven't yet. You never know, they sometimes pop up a little bit further down. So, I guess, you give people information, or they can come on to the website or whatever else. But they might be at different points of readiness to engage. And it's difficult to gauge that really because everybody is different. I guess there's a certain element of you've got to – I guess the website is there, so the website is not necessarily going to reach out. But I suppose you could subscribe to little reminders or something, I guess, that sort of pops up in your email and says, “A new resource has been put on the website or a new this or a new that,” that might re engage.

Interviewer: Do you tend to think that facilitating that engagement with the resource is within the remit of a social prescriber or do you think that it's actually not? Do you think that, in other words, to make this service really effective it should be part of that follow up process or it's just maybe out of control?

Respondent: Well, I'm going to guess that, again I don't know the process exactly, but I'm going to guess the Healthy Lives advisors do follow up. That they don't just leave. So somebody comes to see them and they have a chat. I think there's a certain number of engagements that they have, meetings that they have. So I guess they do check back. But I don't know that they have the time, bless them. I'd love to say, gosh, they should have some sort of reminder that says, this person hasn't done what we've referred them to and it's been 3 months, give them a ring and see how they're doing.

I don't know. They may have that. I suppose their resources are quite strapped, but it would be useful if there was a way that they knew that people haven't taken up whatever the activity is that they've referred them to and then they could be followed up. Because I think particularly, I don't know, post COVID, people have retreated into their shells a little bit more and it is a little bit more difficult to get people out and about sometimes.

Interviewer: Yeah. And I suppose, in particular, with specific groups and thinking about, for example, people living with dementia who might not have caregivers living with them, who live alone or might have mobility issues. There's all sorts of different underlying barriers which I think, ideally, as you say, would be addressed by the social prescribers. And maybe, the service is developing, we will see in the future that it's actually costing resources to have that follow up but maybe it's an investment in return.

So we will see how it develops, but at the moment it's just really, Sue, to get your feedback, your views around these issues as they present. I suppose my final question is, was there anything else that you wanted to add that you think might be helpful? Or at this stage, are you happy with what we've discussed so far?

Respondent: I'm happy with what we've discussed and I think maybe the only thing I would add, and it's probably not a surprise, because I belong to – there’s a group called [anonymised] in the county. And I think that we're doing our bit to try to support people with dementia, but I don't have dementia and I don't have anybody in my family with dementia. But listening to the people that have actually got the experience of living with dementia or caring for somebody, I think they don't feel that there is enough support around dementia.

And I guess that's just a general comment about the understanding across the board really, of people, not just Healthy Lives advisors but just in general, I suppose. People don't necessarily have an understanding of it. They know a bit about it, they hear a bit about it. but it's a scary thing, so people don't really maybe focus on it. They’re just kind of, “Oh, I don't really want to know.”

But I suppose if there could be more of that just general awareness raising of dementia to the wider community, it would probably make everybody's job a little bit easier. Healthy lives advisor jobs would be easier. Our jobs would be easier in terms of getting people to come to our groups and things. So that would be my only other comment but that might be out of scope really, but that's just a few issues.

Interviewer: It shows that there is that sense of isolation and loneliness in getting a diagnosis. In fact, one of the reasons we started off with Forward with Dementia was to really try and raise a bit that awareness. Sometimes people are isolated in their homes and we were finding ways when it's not possible for people to – or they're not willing maybe initially to be in the community to find ways in which we could reach them. And we thought a website, obviously, there's issues around IT literacy, there’s issues around Wi Fi.

Respondent: Accessibility.

Interviewer: And all that, yeah. But it's finding different ways in which really you can reach out to people who are in the most isolated situations. And I guess, what we said before about the importance of community outreach in terms of raising that awareness about social prescribing as well is quite key. So, I'm completely with you on this point, and actually it's part of what we're all trying to, I guess, in our own little steps.

Respondent: Yeah, we're all just trying to look after each other.

Interviewer: Yeah, but anyway, thanks for your views and your insight. It was really helpful. Take a look at Forward, see what you think. It maybe might be helpful for you guys as well. And I'll keep you posted on developments for this website as well. So maybe, you will be able to share it with people when it's ready.

Respondent: Yes. And when you study is finished, will you communicate out to people who have participated to say what's happened? Yeah, that would be really interesting.

Interviewer: Of course, yes, actually, we're looking to potentially do a public engagement final event. So, I'll keep you posted on progress as we go along, so that you're in the loop with different phases.

Respondent: Yes, great, lovely, thank you. Well, it's lovely to speak to you and good luck with it all.

Interviewer: You too. Thank you so much and if you know of anybody else who might be interested in giving some views, just give them my email address. We're actively recruiting.

Respondent: Fantastic, I will do.

Interviewer: Thank you so much. Take care, have a lovely weekend, bye.

Respondent: And you, bye.

[End of recorded material 00:39:45]

**SP09**

[Start of recorded material at 00:00:00]

 Kind of a general one, um, to really get a sense of, of your line of work. So what you do, where you work, and maybe, uh, what's your experience in terms of social prescribing and or dementia. Yeah. Um, so I work for a charity in the north of [anonymised] called, so Community and, um, I've been a social prescribing link worker for about, it'll be two years in December.

And the way we do social prescribing, it's quite different to how other people may do it. So our model of social prescribing is quite different. So for a lot of social prescribing link workers, they probably work long term with people for several months, whether we don't really get The opportunity to do that within my role, just because of the amount of referrals we receive with social prescribing.

Well, with my role, it's not dementia specifically, if that makes sense. It's just 18 with any social issues or social need. I've recently been doing a lot more dementia work due to being involved in a dementia review project. So I work for a charity, but I'm also linked with a primary care network. And, um,

With the charity that I work with, I, I do work with, um, a lady called [anonymised] who sent you the email. Yeah. And she is our, um, dementia development worker. So I've been working in collaboration with her and a GP to do this dementia review. And it's a new style of review. It's basically a pilot. The GP wanted to do a dementia review in a community setting.

So we used one of our, because we've got a few buildings, and so we used one of our

Um, my community buildings and then either myself or the GP or [anonymised] and the GP met up with patients and the style of the dementia review was a lot longer. Um, it, well, not longer, more times allocated for the patient and the carer compared to a traditional review. So with a traditional review with the gp, you might get 10 minutes if that, and it may be the GP just kind of doing tick box question.

Mm-hmm, and that's pretty much it. Whether this style of review, they're allocated half an hour with the GP and then half. And now we're allocated with myself or [anonymised] 's there and we, as well as um, covering like the medical side of things, we cover the social side. So the GP's there to do like the medication review, blood pressure check, um, any symptoms.

Some changes weighing the patient and things like that. And then we do the social side that they're getting all the support. So I think it's been a really good style of review because it just, it's so person centered and it allows. the person with dementia and also their carer and loved one to get support, but because we allow a lot of time, we can also support the patient that attends, which I think is just important to kind of keep that carer from kind of, you know, experiencing carer stress, which I think is quite common when you're looking after someone with dementia.

So that, from doing that review, that has opened up my experience, you know, to working with a lot more people with dementia. But previous to the review, when I started, the review wasn't developed yet. We've still got referrals for patients with dementia, but another thing that was quite tricky was, um, with our role, we don't really do home visits, it's telephone triage, so talking to someone with quite advanced dementia over the telephone can be quite tricky.

So again, a lot of conversations are often with the carers also. So I hope that's kind of answered your question a little bit. No, it's, it's really, really helpful. And you know what, [anonymised], um, I interviewed somebody, a social prescribing link worker last week from [anonymised]. Um, you may know her, I don't know.

Um, but, uh, regardless, um, she was talking about dimensional review as well in terms of this holistic approach. So they're developing as, as you guys are doing. a, again, this kind of more comprehensive and quite importantly, um, a bit lengthier chunk of time in terms of that dementia review, again, to make sure that not only the medical side of things, which is, you know, undertaken by, by the GP.

but the social aspect and, and, uh, not just social, I would say the, the, the kind of everything non-medical aspect is kind of looked at by the, the, the social prescribing link worker. So it seems to be a model that's working. It's got potential and it seems to be. on the rise in terms of implementation. Can I ask you if, are you already doing this kind of more holistic approach to dementia review?

Yeah, so we started this style of review review we originally started it.

Um, it, it would definitely last year, uh, I'm sorry, I've not got a specific date, but, um. But you're already kind of doing it, essentially. Oh yeah, we've been doing it well for quite a few months, we've had to take a pause currently just because of the GP being on annual leave and with kids holidays, because one thing that has been quite tricky is time, really, because.

[anonymised] and [anonymised] where we are at capacity and also the GP works part time and obviously GPs are very busy. So finding that time for it is being quite tricky, but because we all believe that this style of review is the most efficient for patients, we want to carry it out if that makes sense. Yes, yes.

Let's see. The way it began was I think a GP had a discussion with a few other GPs and they talked about they'd like to get into the community setting more to help patients and they got into contact with [anonymised] initially because she is specifically just dementia focused. Social prescribing isn't in her title, but pretty much that is what she's doing.

It's just another form of social prescribing. And, um, she agreed to be part of this review. And then she asked if I wanted to be part of it, which I agreed to, because I wanted to develop more experience. with dementia patients. And then from that, we pretty much just arranged the review amongst ourselves.

So all the planning, um, was just done by me, [anonymised] and the GP, which again were quite tricky because we're limited for time. So we had to find, um, days that would be best suited for our schedules and the GP schedules and. Yeah, it was quite tricky to begin with, but I feel like now we've planned it a lot more better.

There's still a few areas that we need to improve on in terms of making it the most time efficient for us staff, if that makes sense. But, um, and we also struggling with a booking process. So we're currently um, in the process of developing a better booking scheme for

GPs, you know, to book the patients in for a second because, because that's really interesting. I wanted to ask you, what's the kind of referral process for the dementia review? Do you contact people who have a diagnosis or how do you outreach? Yeah. So to be honest, the referral process has been a little bit all over the place because we've just been trying to work out, you know, the best way to get people referred in.

So initially it started off by the GP basically inviting and booking patients in. But then she realized she didn't have the time for that. And she didn't have time to chase up the patients. To check that they were coming and all the admin stuff. 'cause we'd tend to send out letters as well. So we'd do a phone call, send a letter, and then we noticed that, um, when we started doing that, a lot of the patients and the carers were quite anxious of coming to the review.

And we didn't want that. We wanted it to be something quite informal. We wanted the patients and the carers to feel supported and not anxious when they're coming in. Because I think that the word review kind of creates a bit of that expectation, doesn't it? Yeah, yeah. And I think if we've spoken to the patient directly and they've not been able to kind of tell their carer fully what it means, I think they then get worried.

So the, there was a lot of anxiety and confusion to why there was there. So what we ended up doing was we created a bit of a, a sheet to, um, make patients and the carers aware of what to expect and kind of the things that would be covered just to prepare them. Yes. So with that, it was just too much for the GP to book.

patients in, understandably. So what we ended up doing was, um, we have also got a care coordinator. So she is linked with the primary care team that I work with. So she started supporting us in terms of booking patients in. So she had, um, I think she was using the surgeries quaff list. and seeing who was due their annual review.

And then she was contacting them to get them booked in basically. Um, but again, she was at capacity. So she had to kind of, she helped out a lot at the beginning with getting patients booked in, but then eventually it would just. it was too much on her workload. So she had to take a step back. So then when I noticed there was someone with dementia on, um, my like normal social prescribing waiting list, I tend to take them off, prioritize them and get them booked in the review.

Um, So, I did that a few times and then also, um, [anonymised], because she's already working with people with dementia, she was able to check with them if they'd had their review and if they hadn't, she'd also book them in. So, the referral process was just basically us trying to find people who hadn't had realised that it's just...

We'd be better if we had like a normal referral process, like something set, something formal, this is what you have to do to book patients in. So we had a meeting a few weeks ago with some of the GPs and the clinical directors. And I think now we're looking at getting, um, I think they talked about getting it out of hours type of service where the admin staff basically book the patients in for a review, which I think will work a lot better.

So we've not trailed that out yet. We are due to be trialling it out in September. So we are hoping that's gonna be our new referral pathway. So we're going to, 'cause we've all, we've already kind of told the surgeries. Cause I, I don't wait for just one surgery. I think there's about seven or eight that we get the referrals from, and that can be part of the dementia review project.

So we've got to kind of do a relaunch again and go around all the surgeries and. basically inform them how they can book into book their patients into the review and then their admin staff are going to book it in and then we're just gonna meet them the patients at the setting basically yeah yeah yeah okay so it's it's kind of uh a learning curve because it's a new thing and you're trying to, from what I understand, to sort out ways in which this could be, uh, sort of embedded as clinical practice within the different surgeries.

But what's interesting and what kind of echoes what I've heard before is the issue of capacity. Um, and yeah, and I wonder just as a final question around this, uh, dementia review, because I know it's a bit. Off from the main topic of social prescribing, but I think it's it. It sounds as if it's quite important also for people to as an opportunity for people to learn about social prescribing because people do not necessarily know, but also for them to be to be referred.

And that's why I'm asking a bit more about this dementia review, because I think it could ease the process. Um, but what I was, um, final question was, do you envision in the future that this dementia review, given the, the current issues with capacity, do you think you'd, you'd, you'd be able to undertake?

the workloads that will inevitably come with that holistic dementia review process. Because there's going to be lots of people, I suppose, who are going to be needing the dementia review. So for you guys to have that section with the patient, is it going to work out in terms of the capacity you have? I hope so, because this model of the dementia review, it's so, It just makes sense, like, for the patients to be able to access that, and I think, although there's a lot of work to do beforehand, in the long run, it will be better, because hopefully once the patients have attended this review, a lot of their needs will be addressed in the review, because A lot of time has been allocated to talk through what's available both through a medical perspective and a social prescribing perspective as well.

So I can see it being challenging if you've just, if it's just one social prescriber and the GP doing it. So, for example, myself and [anonymised], because we were both pretty much at capacity with our workload, we... alternate the weeks that we go to manage the workload, if that makes sense. So the dementia review, it's, um, once a week.

So we do it once a week on a Tuesday for a few hours. So it's not all day. I think it's about four hours. Um, and she does one week I do the other week just alternate. So that works for us, but I can see somebody who hasn't got that option. It would be quite tricky. Um, and obviously we won't be able to see all the patients at the surgeries by doing this style of review.

That's something also to consider. So, because there's only four hours once a week to do these reviews, we're only limited to the amount of patients we can see if that makes sense. Yeah. So I suppose with that, that does kind of help a little bit with the capacity because you know, you're not going to be seeing like hundreds and hundreds and hundreds of patients at a time just because we've only got a few set amount of hours.

So, you know, you can basically get done the work during that day of you doing the review, if that makes sense. Yes, so, um, doing this style of review because you're, allow more time for the patient and the care and more things do unravel. So for example, we've had quite a lot of safeguarding that we've had to obviously deal with, um, because there's been more time to talk about issues and concerns.

So often that has brought up a lot of challenges, which again, You need to safeguard people but it does add that extra layer of work to your workload if that makes sense. So for the GP and for um, obviously myself and [anonymised], but I do think even though it is tricky with capacity and it does put a lot of more workload on your shoulders, it does get the GP to understand your role a little better as well because they're seeing it.

face to face and seeing what you're able to offer. I think that is helps the GP fully understand what social prescribing is the

need. uh, actually for some culture change within, uh, GPs. And, and part of educating them seems to be within your, uh, spectrum of a thousand, a thousand responsibilities that you have, because I've heard about this. And, and I, I agree with you that it could be a way for them to really understand what you're there for and how you can support them.

But I also understand what you said in terms of investing. Time and resources in that dementia review, because then in the long run, it will save resources and time to this potential to the social prescribing work team, because I suppose that dementia review will somehow work as. Almost a social prescribing session in a way where maybe you can address some of those needs immediately without necessarily, you know, following up the patient.

So I can see that. And I agree with it. Um, so that's very helpful because again, it helps me understand what's happening in different services because it's this huge diversity out there. But I see that the dementia review. Thank you. holistic approach is kind of developing so that that's really interesting and it's a model that maybe could be shared across different, you know, um, PCNs.

Um, so now I will probably pass on to discuss a bit about dementia patients because that's the core thing, isn't it? So you said. You're a social prescribing link workers, which is great because I would really like to ask you, maybe let's think practically, let's think about a person you've supported. Uh, living with dementia.

Can you sort of describe maybe, uh, the process from, you know, the initial referral, maybe the first contact, what some of the challenges were, and how you then developed a kind of tailored plan to support the person, just so I have an idea of what the challenges, the barriers are, so that we can think about resources that you guys might need.

Yeah. So from the initial, well, the initial process, uh, when we get the referral, they get added onto our waiting list, which is quite long just due to the amount of referrals that we receive. Um, so often, sometimes when you're making that initial contact, by the time you've contacted the patient, they may, have forgotten about the referral.

So they may be a little bit confused to why you're ringing. Not, not in all cases. And we prioritise referrals in a matter of urgency. So, urgency would be a person with dementia who is homeless, has no food, no gas and electric. Then, we have high priority, which may be a person with dementia who hasn't got attendance. We tend to put them on high priority just because how long the process takes to claim. And then we have moderate, which would be people that are interested in social groups or activities.

So you guys already kind of know or you decide. No, so we decide, so we've got our own referral criteria and our own, um, assessment for in terms of urgency of referrals. So we would assess, because sometimes we get referrals from GP saying urgent, urgent, and we're like, well, that doesn't meet our urgent criteria.

So it may be urgent to you, but we have a criteria. So, um, so say if, so for an agent for us, it would be homeless, no food, no gas and electric, which it's very, I don't think we've really ever had anyone. with dementia in that situation. But then after urgent, we have high priority, which it may be struggling financially.

Um, we tend to prioritize, um, sometimes we get referrals for people with dementia and they haven't got attendance allowance. So we tend to put them on high priority just because how long the process takes to claim. Um, So, and then we have obviously moderate, which would be people that are maybe just interested in social groups or activities.

So we do tend to get a lot of referrals for people with dementia from GPs. Um. That are socially isolated or really low in mood or may have had a bereavement and they've got dementia. Um, I'm just trying to think of referrals we get. Um, we also tend to get a lot of referrals. For the carers of people with dementia as well.

And again, that's another thing, um, in the process to say if we make contact, um, for someone that has got dementia, a lot of the time it's the carer that we speak with, right? Yeah. Um, that, that is. I'd say maybe eight times out of ten is always the carer that you speak with first. Um, just because we do get a lot of patients referred to us with dementia that's quite advanced and they might not be able to communicate properly on the phone and they may struggle with that.

Can I just ask you, since you're talking about this very interesting point, do you ever get a referral for a patient living with dementia that really... Is a referral for the caregiver, if that makes sense. In other words, do you think that it is the caregiver that has actually initiated the process because they need social prescribing or support?

Mm-hmm. But only the person that's been referred to you, the person living with, is that that? Does that make sense? Yeah, I think so. I think we have ca we have had cases like that, and I do also think we've had quite a few referrals. I think the GPs and also family members tend to do this for social prescribing in general anyway, as well as people with dementia.

But we do get a lot of referrals where the person with the GP

has thought, Oh, I know what's good for them. Still wants to go to a social group, I'll send it to social prescribing. Mm-hmm. Or again, a lot of, um, the caregivers or the family members with people with dementia will think, oh, I think they'd enjoy this. Um, I want them to get support. And when you speak to the person, they don't actually want that support, if that makes sense.

Yeah, it does. Completely makes sense. And, and how do you actually work with, with that patients if you, if you really realize that, It's, it's about what other people want as opposed to what they want. Well, you just have to respect that the individual's decision. So if that person with dementia doesn't want to go to a social group, for whatever reason, you can't force them to go to the group.

Obviously, you've got to respect that person's wishes. And yeah, what I tend to do is I do explain that to the family member and the person with dementia. Um, sometimes the person with dementia might not want to go because they may be a bit anxious. So again, you'd have to try to break down them barriers first and maybe find out the reason why they don't want to go.

And then if it is, they just not very social. They've never been social, but the family member thinks a social group would be good for them, which is the case a lot of the time. You'd just respect their decision and, um, give them kind of information and advice on what they've been referred for. But if they don't want to take it, you've, you've got to respect.

Then of course, I think sometimes the caregivers and the family members that are looking after someone with dementia often see that person with dementia as they've got dementia that takes over their life. If that makes sense, they do think about their personality that they once had or the things that they enjoy.

They just tend to think, Oh, that they've got dementia. We've got to do this and we've got to do that. So say for example, they've never been social. They've never liked. to socialize and then they're starting to lose capacity and they've got a family member looking after them and the family members trying to make them go to a group but they've never enjoyed socializing.

It, it's, it can be quite tricky. Um, we have had experiences during, well, we had a case during our dementia review where we didn't actually get to speak to the patient because they didn't attend, it was just their family member that attended because they couldn't get the person with dementia to attend the review.

But, um, The family member ended up, um, talking about quite a few concerns which did end up having to be a safeguarding incident because the person was self neglecting and then we managed to get some carers support in place for the person that attended. So, I think we do. deal with a lot of barriers. I think a main barrier to our referral process when we're not doing the dementia review is just with it being over the phone.

Um, cause we don't really do home visits, which I think the GPs don't always understand. So we will try to explain to them, although a lot of social prescribers do. We can't offer home visits. We're just with our capacity. We're not able to do that. And if we offer it to one patient, we'll have to offer it to all patients.

So it's not something that at the moment you can, you can do. And I suppose the challenge here as you, as you, you. It's over the phone to have a conversation with somebody who's got dementia, potentially, you know, even a bit more advanced stages. And really gauging over the phone what is that they want, if it is what they want or somebody else wants for them.

Um, so translating this into practical. advice, um, for this resource for social prescribers who, by the way, from what I've heard, the majority of does only telephone, um, you know, consultation. So that's, that's the case. What are some of the, of the pieces of advice you'd offer to somebody who needs to understand what the person wants over the phone?

A person with dementia, what are some of the things that you do? Practically some of the strategies, maybe, um, Well, I try to, especially on the initial call, try to build a bit of rapport up with the person with dementia. So even if it's just that you have a bit of like a general chit chat first to kind of get that person engaged with yourself.

Also making sure that you've actually got time to listen to them, because sometimes it can be quite a long call. So making sure you've actually got. A good set of time to speak with and talk about different topics and, um, Also, I've noticed as well, if you feel, say if we've got a referral for someone with dementia and they need an attendance allowance and you talk to them about the finances, a lot of the time it's a carer or their loved one.

That is supporting them with their finances and the person with dementia might not necessarily know kind of what they're already claiming, things like that. So making sure you get consent to speak to, you know, the loved one or the carer that is, um, obviously looking after them and their finances. Um, I'm trying to think what else it's difficult because when you're in the moment, you just kind of have to adapt yourself there.

And then, um, I would recommend like trying to do different. Types of training as well on dementia, so there's lots of different things out there, which that's what I've been doing to kind of understand better about people with dementia and kind of, that's what I've found has helped break down the barriers is doing the different types of training that are related to dementia.

Do you guys get any training in terms of like basic or mandatory training or is it just down to you? to get that training. So the surgeries don't provide the training. I'm lucky that I work for a charity that are very, um, encouraging of trying to do lots of training and lots of development. So, um, I've done all the dementia stars training, which was done through the charity, not the surgeries.

Um, so I can see if someone. If a social prescribing link worker is just linked with the surgery, I can imagine it, it being quite difficult, you know, to access training because we, because I'm linked with a charity, we get a lot of information about the courses through staff at the charity, and whether if someone doesn't have access to that, I can imagine them being quite limited to accessing training.

So I've done that training. I tried to do, um, like dementia related webinars when I can. Um, I know, I think it's an organization called [anonymised]. They tend to do monthly like discussions around dementia and it's got lots of people that work with dementia talking about like best practice and best ways to support people with dementia which I found really useful.

I think, I think your position is quite lucky because as you say you work with a great charity so that kind of compensates for it and I'm wondering whether because As you rightly pointed out, not all social prescriber link workers will, will be this lucky. Um, I suppose maybe in this resource that we create.

We first obviously need to say training is very important, as you're pointing out, to really understand those barriers, added barriers that you have with this population, but also maybe putting some links to potential trainings, be it online or, you know, offered by, you know, different organizations, but maybe it's something that we, we should, um, we should, um, signpost, um, people to, because it's important it sounds from what you're saying.

Oh yeah, definitely. Bye. Okay, okay. One, one thing that came up in other conversations is that sometime when a person living with dementia gets a phone call from a social subscriber the first time, there's often that kind of rejection of care in a way, because they're scared. They don't know who the person is, why they're phoning.

So. What happened in lots of these instances is that the case, the referral was, was closed because there was that no and, uh, but, but we all know that with dementia, you have to work around the nose because it needs to, to have that trust in place and to build that trust, you need time. So it's all these things you mentioned are quite important while working with this population to make sure that it's not just, you know, a phone call and then you close the referral because it was a no.

Yeah, it's tricky as well because I've had a case where I would try and get in contact with this lady because we also have a rule where we contact someone three times whether that be telephone, email, letters, we're sending emails. Especially to older clients, we send, we tend to send like, um, contact letters out and she wouldn't answer, but then she'd ring me at night

and I thought, oh, like, why is she not answering the day, but she's ringing me at eight o'clock at night. On the referral they didn't mention she had dementia and it was only until I eventually spoke to her, she told me, well she, I don't think she knew she had dementia, she described the symptoms of dementia of what a GP told her and I, I worked, it all clicked together and I thought oh she has got dementia.

And, um, I think she struggled with the concept of time. So another barrier is when GPs refer, they don't, some do, some don't, but some tend to, they don't put enough information on. So, which again creates another barrier. So in that case, with that lady, I didn't know she had dementia because it didn't mention on the referral, it just said she were interested in a walking route.

And if I knew beforehand she would have had, that she had dementia. I would have put more things in place to make the service more accessible, if that made sense. But because of the lack of information until I actually spoke to her, I wasn't able to do that. So I'd say that's another barrier as well, like lack of information actually on the referral.

Yes, and maybe it was thanks to the fact that you had that basic dementia training and awareness that you were able to quickly identify that that particular woman might have dementia in the lack of an official kind of diagnosis. So that hence again, the importance of being able to really, um, you know, understand those signs that tell you that, that maybe the person has dementia because at least something that came up, uh, based.

Kind of reflects what you're saying is that lots of social link workers to really don't know that the person has dementia. In other words, they're not referred. Um, the, the clients that they're, they're referred, um, do not have either an official diagnosis or the GP just didn't tell them. It's something that's coming up.

So hence again, the importance of being aware of reading, you know, between the lines, those signs that might tell you, well, maybe something's going on with this person, potentially even referring the person back for, um, diagnosis. to the GP. Does that ever happen in your practice? You know, figuring out that something is not, is going on, that the person might have dementia, so you refer the person back to the GP to get an official diagnosis.

Well, we... I've never had that. So, in this case, that lady had already been diagnosed. Right. It's just they didn't put it on the referral, but we do have a lot of referrals for people that haven't been diagnosed yet because of the waiting time for, to be seen for a diagnosis. But, so... In [anonymised], I think if you're awaiting for a dementia diagnosis from the memory clinic, you're waiting months and months and months, sometimes like six to nine months.

So we tend to get a lot of people referred that who, you know, haven't actually been given that diagnosis yet. But another thing, a good thing about the dementia reviews that we've been doing is the GP that attends the review, she can give, she can't give an official diagnosis, but she can do, I forgot what it's called, but that she'll basically confirm.

Yes, you've got dementia, but then the issue is with that is not all GPs feel comfortable to do that. I think it's only because again, that GP's had a lot of training around dementia. So she's comfortable to do that. Whether I don't think all GPs are, if that makes sense. It does. Yeah. Yeah. So it's, uh, again, the importance of that dementia review is that it actually might sort of put in place a diagnosis.

Yeah, unofficially. But, but you guys work within that framework in mind that this person actually has dementia. Yeah. So it helps, it helps the whole process. Okay. That's really, really helpful. Uh, I'm just really wary of time 'cause it's 45 minutes and I don't want to take any longer, um, from you because.

Uh, you, you've told me a lot, and by the way, I'm also, I'm also talking to your colleague. So what I'll do with her maybe, is we started to explore together, me and you, the initial referral process and the first steps of conducting the patient. Maybe I'll follow up with her on the following steps, as in putting a care plan in place.

Signposting to activities that, that will give me, you know, very kind of a holistic, um, you know, perspective on what you guys do. So, um, was there anything final, [anonymised], that you wanted to point out that maybe is helpful? Um, or are you happy with what we've discussed? I think another thing that's helpful is just kind of knowing the support that's out there because also sometimes the cases that you get with dementia can be quite upsetting.

You can get, especially with like safeguarding concerns or difficult cases, I think also Making social prescribers aware of like the support that you can get if that makes sense. So for example There is I don't know if it's just in [anonymised], but we have um a social prescribing like Peer support meeting once a month and you get to talk to other social prescribers about challenges that you may be having best ways of practice um, advice and I find that quite useful to get advice around like patients with dementia or just any patients really and also kind of a bit of emotional support.

It can be quite challenging and tricky dealing with people with dementia due to the vulnerability and kind of the tricky complications that can come out. So I think it's good to know what support is out there for social prescribing link workers in that case, if that makes sense. Yeah. And also, I suppose it's an opportunity to share information on other potential important stuff like training or, you know, pointers activities going on in the community.

It might be helpful to know it's, it's really something. interesting, and I didn't hear about this happening in other localities, um, but maybe, yeah, maybe I didn't ask, which, which actually prompts me to ask to, to other people I interview, because I think having that emotional, uh, and practical support in place for you guys is quite important because you support all different kinds of populations with all sorts of challenges.

So it has an impact on any person. Um, that's, that's, that's great to know. Okay. All right. So, um, so again, thanks for, for your time. It was really helpful. Oh, good. If you've got any other questions, I don't mind if you want to email them in. I can always answer them over email if there's anything else you wanted to know.

Yeah, what I'll do is when I analyse my data, I may come up with some further follow up questions on some of the details that we discussed, in which case I'll, yeah, I'll email you and we can take it from there. Um, so for the time being, thank you. Thanks a lot. And, uh, and, uh, have a lovely week. You too. Nice to meet ya.

You too. Take care. Thank you. Bye. Bye.

**SP10**

[Start of recorded material at 00:00:00]

The first question is a bit of a general overview around what you do. So maybe if you could tell me what your kind of role is and also maybe we'll get into how the referral pathway is structured within the social prescribing service that you provide. Sure. So, um, yes, I'm a social prescribing link worker, uh, based in [anonymised] and, uh, we work within, uh, primary care networks.

Um, what that means is that we work within areas of clusters of GPs that are separated by, uh, postcode. And, uh, so. Within, uh, where I work, we receive, uh, referrals from a number of gps in that, uh, primary care network. And, uh, these referrals are for those over the age of 18 and cover a variety of different, um, areas.

So we receive referrals, things like finance, housing, um, anything mainly that's non-medical, that's more of a, a social issue. And I guess, um, part of the social prescriber's job is to address the wider determinants for health, which is, um, you know, the wider social determinants. Um, so in my day to day role, I, uh, receive the referrals from the GP.

I then, uh, start a short term, um, support with, uh, patients. This is generally looking between six to ten sessions, um, and it's very holistic. Very person centered and tailored. So that could span from, you know, two weeks of support to months and months of support, depending on what the patient does need. Um, the primary basis of the support that we provide is to signpost, refer patients and let them know and bring, I guess, awareness and improve access to community services that can support them further.

We work on a plan, um, you know, what matters to you, what's your goals, what's your concerns? Then we go through that plan and try to address, um, whatever the issue is, um, or the goal is to improve their health and wellbeing. Um, we sort of try to collaborate as much as possible with the GPs as, as well as community services so that we can, um, support the patient as holistically and all round as possible.

Yeah, I suppose that's very helpful. I suppose, um, this kind of holistic approach is particularly, um, helpful when it comes to population living with dementia and, um, what I'm trying to get at is kind of involvement of, of caregivers as well of the family in that kind of, uh, a process of setting up goals and the whole package, I suppose.

So it really much makes sense what you're saying about adopting that, that holistic approach. Um, would you, um, normally support people living with dementia within your role? How often do you get to support people with the condition? Um, yeah, so we do have a, you know, we do have a number of patients that come through that need support with dementia.

I would mainly talk to family members or carers, sometimes the dementia patient themselves. Um, I would say the question of how often is a bit of a tricky one because, um, When I mentioned that we're based in [anonymised], so we have a team of social prescribers, the 22 in [anonymised]. And I know that from my colleagues, certain areas have kind of more, let's say a higher, uh, elderly population.

And so they will see more dementia clients. Um, I think the area that I'm in now, um, we'll probably see. maybe as little as 10 percent of patients that come through with, uh, you know, dementia needs and, um, you know, sort of care and support around dementia. Um, and what's also interesting as well is that sometimes I will receive referrals for, um, you know, older patients and whilst I'm in support of them, that's when the diagnosis of dementia has, has, has come up.

So, um, Yeah, so I don't know if that answers your question. Yeah, and actually it kind of confirms some sort of feedback that I've had from other services in one, in the sense that there's huge diversity out there in terms of the population that you guys support. So within your service, it seems as if, you know, people living with dementia form a kind of minor, small, relatively small minority.

of your whole, um, um, you know, kind of patient, um, poll. Um, so that, that's one, something that also reflected in other interviews. Um, I suppose another point that's worth mentioning it, is that it seems that you guys have a fairly large team of social prescribing link workers, as opposed to other localities where there's even like one or two people who really struggle, um, you know, with these systemic kind of issues and resources.

So that's something that also came up in previous conversations with people from [anonymised] and in comparison with others. And, um, there was a 3rd point, um, in my mind, which is just slipped. Um, who is that? Oh yes, the point you made about, interesting point, about people coming to you guys without a diagnosis in place, an official one, sometimes it's because, you know, times are kind of very long before you get a diagnosis, so there's that gap, but you're supporting the person in the meanwhile.

But also, interestingly, what some people told me is that sometimes they... they're referred through the GP's, but actually somehow it's you guys potentially spotting the signs that something might be wrong with the person and referring them back for kind of a memory assessment or an official diagnosis.

So this is kind of reflected in previous conversations that I've had. Um, I thought what I could ask you is you started to, um, hint at some of the challenges in supporting this specific population. I wanted to ask you based on your experience what some of these are because it starts, you know, kind of making me aware of the practical things that we would want in a resource for, for, for, you know, you guys to support people with dementia.

Yeah, I think one thing that you, I think one thing that you just mentioned, I think was just one of the biggest challenges when they don't have the diagnosis yet. And so, um, and, and then that can split patients into what I commonly see into two areas. One that they are aware that something is wrong, you know, with their memory and, you know, their perception and they, they recognize that.

And um, you know, they're in this sort of limbo of waiting to find out or have a confirmation of what is actually. going on with me. And then you've got the other side of people who don't recognize that there was something wrong, but it might be their family members recognize or I recognize or, you know, people around them, but they themselves are not recognizing, um, that there is something wrong there and that both of those situations present, you know, different and unique challenges in itself and in trying to address.

Um, or, or trying to link them to support, um, trying to find access to that support, you know, we know of, um, certain community, community organizations and charities that help with dementia, but if you haven't got that diagnosis yet, what, you know, how can we get you that related support? Um, and so, there are some, there's definitely some challenges there, um, I've, I receive a lot of time.

Can I just chip in with a quick follow up question based on the point that you just mentioned? Do you ever come up with client, with patients, where it's sort of, they haven't got a diagnosis, they might be aware that something's going on. But they actually are a bit resistant to getting a diagnosis because they might have, you know, certain background in terms of culture, in terms of, uh, all these, you know, other, other aspects.

And when it does, how do you really work out to, to get to the tailored approach to care? Yeah, so that, that is, um, it's a difficult one. Um, I think one thing that is really as helpful as possible is just having family involved or, you know, um, people that are close to them involved. But if you have the, If you have an isolated patient, for example, who comes from a certain cultural background that doesn't really, is not really open to that type of diagnosis and support, um, you know, I think the best thing is we do have to listen to them.

We do have to take into account how they're feeling, but at the same time for their sense of safety and, you know, for them to get the right support that they need. For example, because of the lack of dementia diagnosis, they might be neglecting themselves. There's an issue of self neglect there. What I tend to do is try to look into the community to see where we can get them support in.

something that caters to their cultural background, for example, or that that's in that context, or try to find something as close as possible to what they recognize that can give them that support, that can, um, explain it to them maybe in that context that they are in, um, and try to just call in as much help as possible.

Um, for that patient. The only thing is, this is the limitation when it comes to social prescribing is that, um, we need patient's consent to be able to, to go forth with certain things, unless it's an issue of safeguarding. Um, you know, we would need the patient's consent. So unfortunately if a patient does not consent to that, um, you know, that's where it does get a bit difficult.

Um, I've seen, I have a patient, actually, a previous patient before and, um, you know, they didn't consent to, to that type of support. Um, you know, they did have some, what, from my point of view, some there could have been some issues with mental capacity. And so what I have to do is they don't consent to support.

So, if I see there could be issues with mental capacity, I close the case, let the GP know and I push forward for a mental capacity assessment. This would enable us to instigate power of attorney or appoint a mental capacity advocate for the client*.*

Um, if they do, if it does find that they do have the mental capacity, we just have to respect their decision. And sure. Yeah. I suppose the whole thing about social prescribing is that you guys can signpost, you know, opportunities, but you can't really make the person engage with those opportunities. So it's consent, you know, it's, it's a, it's a wide kind of, um, spectrum of consent that you There's only so much that you can do that.

That's what I'm saying. So it's, uh, it's, um, it's always keeping in mind that, you know, your role and your remit and your goal kind of ends when the person hasn't got that, um, you know, willingness to engage or can't really engage with the whole process. Yeah. Also, you touched on another point about the importance of having these conversations and getting the consent from the person and their engagement.

I was wondering, um, and you'll be able to tell me, um, if you support people, mostly Yeah. Absolutely. remotely, so through the phone, through phone conversations, what are the implications and the barriers that dementia presents when you have to build a report and get that consent, and how can we address them?

Yeah, so, um, I've had, I've actually gone through, uh, some difficulties with, um, trying to support remotely, uh, a dementia client and, you know, This is where sort of, um, adaptability does have to happen just because maybe their perception, um, and how they're receiving the information on the phone. If they have a dementia diagnosis, it can be really difficult.

And so, you know, trying to meet up with them face to face at the practice, um, maybe, you know, speaking to the GP, if you can join in within have an extension of a GP appointment and, you know, sit in with them. Um, again, speaking to family members is really helpful. I try to, if it comes to a vulnerable person, um, with their consent, I like to involve family members, um, you know, or close.

Relations as much as possible if they are comfortable with that, if they are happy with that, because it is really helpful to have their support to relay that information, you know, there's some dementia clients that I've supported before that have a language barrier and they may have been, they've may have gone to a point in their life where they speak great English, but because of the dementia.

then now, you know, reversing back, it's hard for them to understand and so having a family member there or having the GP there to be able to help really relay that information has been really helpful. Um, but what I tend to do is offer face to face appointments, um, at the GP practice. Okay. Um, yeah. So it seems that within your service, there is the option.

to have a face to face appointment. And that's great. Yeah, that is new. That is actually new because, um, with the COVID, everything was, you know, everything was remote. So slowly, maybe from 2022, we started to, uh, offer back face to face appointments for those who do really need it, for those who find it difficult to be able to have a remote consultation.

It sounds great. Um, and to be honest, from experience with interviewing other people, I've often seen that social prescribing link workers are sometimes discouraged to go face to face, which kind of, again, raises all sorts of barriers in relation to how do you really Get to develop that report and understanding the needs of this population in particular, when, you know, a remote contact raises all sorts of barriers.

And so it's great that you guys do have that opportunity if needed. And I was wondering, [anonymised], when it comes to having that chat with the person, be it in person or remotely. How do you work out with a person with cognitive impairment, what it is that they really want? Um, do you have any particular strategies?

I've talked to some people who use motivational interviewing, but that's just one of the strategies that people use. Um, in your experience, how can you understand as a social prescriber, what is it that they really want? It's a good question. That's a very, very good question. Um, so we do employ techniques such as motivate, motivating, motivational interviewing, um, you know, things like coaching methods, but in my experience, I find that that's not always helpful for, you know, dementia, uh, patients.

Um, I think one thing that's, helpful from my background. And this, I think is more specific to me as opposed to social prescribers is that I used to be a support worker before for, um, for older people, um, including those living with dementia. And I think I was able to draw on that experience of, um, creating a safe space for, uh, for patients exploring how they feel being very patient, very, very, very patient.

And. Active listening is very, very important. Um, sometimes, you know, you might be able to recognize that in their mind right now, they're not in that space with you. Their mind is, is somewhere else at that moment. And I think it's about trying to understand where they're at instead of trying to just abruptly bring them back to reality.

Um, I think that helps to build a rapport is to understand, you know, them feeling safe with you, them feeling, you know, and understanding that you're here. for them. And, um, sometimes it will take more than one session just to get there. You might not get their goals or their support needs in that first session.

Um, especially if it's more of a, an isolated patient who doesn't have the support of their friends and family, it's just, um, they're just talking to you. They don't have that extra sort of, um, uh, feedback, you know, it may take some time, but, you know, you start to. You start to build up to it, build that trust and then start to approach it as sensitively as possible.

And then, you know, start to ask questions about things they might find difficult in a, in, in day to day, or how was, what does your day look like? You know, talk to me about your day and, um, and certain things. And I do believe that they do start to open up. Um, I think adaptability is important as well. So you might ask that question first, if they don't, if they're not receptive to it at that time.

Okay, leave it then, ask it another time, you know, and, and see how it goes. Um, mood is important, be able to read their mood, you know, um, some people with dementia can be very, very frustrated at themselves for even having dementia. And so bringing up the topic brings up a lot of frustration for them. And, you know, just being able to recognize and be sensitive towards that, I think has been really helpful.

But this was something that I had picked up from my previous work experience. I know that social prescribers, my own colleagues, find it very difficult to have the conversation and be able to navigate. And so I think, um, I think for social prescribers having that type of, I don't know, uh, training maybe, or, or conversational workshopping or these tips.

shared across the whole social prescribing board would be really, really helpful, particularly for those who don't have a background in supporting older people or supporting people living with dementia. Yeah, I think you touched on so many important points and it really shows that you are kind of facilitated in your role with this population because of your experience, because of your previous experience.

And I agree there's lots of other social prescribing link workers who might have a different background. They might have worked in mental health, they might not have worked in any area that's really particularly relevant to dementia, and it's important to, to provide them with sort of, um, awareness about all these implications when it comes to building a report with somebody living with dementia.

Um, I've. Interviewed people who've told me that they didn't know better and they closed referrals after a session because the person wasn't responding because they were rejecting, um, care. And that happens, you told me, about, you know, with people living with dementia. It's not always a yes at first, uh, you know, um, opportunity that you have to interact with them.

And you have to work through those no's and those high and lows in terms of mood as well. And you know this. stuff, but we're trying to create something that support people who haven't got that background and knowledge to be able to not, you know, just, um, shut it down for people living with dementia because sometimes that happens.

Yeah. So, so, you know, all the things that you mentioned in terms of having, you know, a bit more time to dedicate to, to that person to really understand them. What we're thinking about is sort of potentially creating, uh, just as a starting point, almost a checklist type of thing where there's, you know, in terms of, say, first session, have you tried to do this?

Have you done this? Have you tried to go back to the person? Have you checked what the mood might be like? Something like that, so that somebody who hasn't got experience at all at least has some kind of tips or hints as to what might happen in the sessions. Do you think that is a good way, potentially?

Absolutely, I think that would be really, really, really helpful. Um, yeah, definitely. I think that would be okay. Okay, I'm just trying. to gauge with these interviews that I've done so far, whether we're kind of progressing towards something that could be practical because, you know, we just don't want to, you know, give people, um, you know, hyperlinks to dementia training.

when maybe they don't have the time or they're supporting like a thousand different people with different conditions or intersections of different, uh, things. So we want something really practical, which isn't time consuming, but at the same time we want it to be informed by evidence. And, and what you're giving to me is that kind of evidence of what potentially should be in that, in that checklist.

Is there anything else that maybe could be helpful to include in something like that, you think? Yeah, I think it's. So I think that's such a great idea. And I think even within the checklist, it's just, it helps us to reflect. So even reflection points on us, like how is this person feeling today? You know, and then we just note that down or it helps us to be able to ask those questions as well.

Yes, I think just being as reflective as possible, having that checklist. I think that would really, really help. I'm trying to think of anything else. Um, what do you think? Can I just chip in with a question? Because you brought up something about reflectiveness, which I think is quite helpful. And I wanted to ask, um.

Do you guys have a, like a venue or, or meetings with each other where you can actually reflect on your own practice and share with others? Yes, so we do it in [anonymised] social prescribing. I think each week we have a peer support group. Um, we also have drop in sessions as well. Um, and that's for us to present cases to reflect on practice for each of each other to, you know, sort of support each other.

on those type of cases. Um, that might present. Uh, so I think it is quite a good time for reflection. We also do have our supervisions once a month, which is more about reflecting on ourselves as opposed to the cases that we get. Um, So yeah, we do have moments for reflection. However, I do think when you're going in the daily schedule, you just go through the hustle and bustle and, and don't, might not have time yourself to actually reflect on, you know, what, what's actually going on right now in that, in the patient's headspace.

Um, especially when you're. Time constraint, because that's another, that's another issue, is time constraints. Um, you've got this schedule, you've got all these appointments booked in, you might not have the time, you might just want to address the referral reason, the actual goal, the actual concern, and not actually, uh, have that time to actually

So we're going to that patient is at that point, you know, to, to take on what you're saying, how did you find speaking to that patient? We just a lot of times don't have that time to think about it. So key moments like our peer support group. is important because it gives us that space as well as the one to one monthly.

Um, but I think having something like with the, with the dementia patients, having something like a checklist, because dementia is so huge. It, in terms of like the spectrum and how it, um, affects patient is so wide, it doesn't affect everybody the same. And, you know, maybe having that awareness. I don't know how, maybe in a sheet or some sort of something just to, I don't know how, but just something, being aware of that, um, could really, really help.

Yeah, I agree with it, with this point in the sense that we tend to think about dementia about, you know, memory problems. The person will present with this and this and that. Yes, it might be the case in potentially like 80%, but there's, as you say, huge diversity out there. And as we said before, even like the intersection with culture, background, it changes the ways in which dementia is acted out and the behavior and the response to your care also changes.

So it's important, I agree, maybe in that kind of resource that we're creating, if it's a checklist, to make sure that people understand that there is not one way to go. With a patient with dementia, um, dementia, you know, it's like a great term, but it doesn't really encompass the diversity that's out there.

So we need to make sure that people know, um, but also what you said before about, uh, peer support, um, it, it, I think it's important and I was talking to another person this morning and they had this system in place and she was saying that it's also important in terms of potentially sorting out um, sort of emotional responses that you might have when you're supporting people with very difficult situations, um, because you're time constrained.

And sometimes they say you don't have that time to even reflect on your own responses to situations. Does that happen in your peer support groups and all that? Oh, yeah. We, it's so funny because we have a little, uh, we have a little joke that we call it almost like the rant club. Because sometimes, sometimes it's time to just, you know, sort of rant about how this made you feel.

I mean, not all patients are. easy to work with. Some can be very difficult. And so just being able to express how that made you feel, how somebody made you feel, how that, um, approaching that sort of case made you feel, um, it's really, really important. So we do have that sort of space to, to sort of, um, outlet emotionally.

Yeah. There's one thing I wanted to mention. Um, oh, so I think, um, one thing that I realize and see in practice, not just in social prescribing, but I can see on a wider spectrum is that oftentimes we see dementia patients as we sort of label them as, oh, you know, They're vulnerable, they're not in the space to be able to be at the centre of their own care and support.

Sometimes we have family members driving that too. So family members will want to speak to you and speak on behalf of the patient. The patient's right there, um, in the background hearing exactly what the family member's saying and this can cause all different types of feelings and I think one thing that I can add that could be reinforced to social prescribers is try to I did say including family is really important, that is true, but also try to put the patient back in the centre of the support.

It's meant, social prescribing is meant to be patient led, and so we tend to forget sometimes if a, if a patient with dementia is, is struggling, um, particularly with mental capacity and memory, we just... I just tend to think that, you know, we, we should just speak to people who have the sort of mental intelligence to be able to answer the questions and drive the support and just exclude them, which is, which is not nice, which is really, really awful for them.

So I think I just wanted to include that point as something that I've noticed. across the board sometimes with healthcare, community care, you know, it's all about trying to bring back the patient to be involved in potentially the outcomes of their health and care. Yeah, yeah, no, I, I, I love that. And to be honest, I've had lots of social subscribers come to me and saying to me, um, sometimes it's difficult to understand.

If the person is being referred because they want to be referred, or is it because the caregiver, you know, needs some respite, or they think they know what the person wants. So, hence my question before about how you really have those honest conversations where the person is at the center. So, it all makes sense, and I completely agree it's important to involve, you know, the family in conversations.

Also, in terms of getting kind of reliable information, because sometimes the person might not remember, they might report, you know, not necessarily accurate stuff, but at the same time, it's in keeping in mind, as you say, [anonymised], that this is person centered care. And it's patient led. And I suppose it's difficult to find that balance sometimes with this population, but maybe it's, yeah, maybe it's the same with other population groups you've supported.

I'm wondering, you know, intellectual disability might be another group. Absolutely. Absolutely. It's very difficult. And I think part of it also just comes through a lot of social prescribers that you've probably spoken to. We use the same system, um, sort of like the referral system from the GPs. Now that referral system isn't as detailed as Could be.

So what happens is we get a referral, the person's name comes up and then it says referral reason, dementia support. Right. That is wide. That could be like you just said, you know, the person needs a, it could be carers needing respite. Um, it could be so many different, somebody needing a dementia related activities and it's so wide.

And, um, I think maybe having more. specified knowledge from the GPs, from the referral partners of what actual support. It will help us then to be better prepared to approach that type to, to coming, you know, prepared and ready, um, for, for that type of support that we're going to give. That's great feedback.

So what would you normally do, [anonymised], if you were in a situation where you would just get, you know, dementia support? Would you, what is good practice to really know more? Would it be to go back to the GP or maybe they don't have time? How do you really understand in that situation? Yeah, so, uh, what I tend to do is I'll see that referral and then I'll go straight on to their medical notes and see what generated that referral.

Right. There'll be, it'll be great sometimes when the consultation, you, you get to see the whole consultation and why the referral was made, then you get that understanding. Sometimes, not all the time, probably not most of the time, sometimes we have the referral partners, GPs will just refer a patient, um, without their consent or without the patient's knowledge.

And so now we're calling the patient, like, this is the referral that we received from your GP and they're like, I have no idea what you're talking about. I've already told my GP I don't have this type of issue, you know, so then there's a bit of resistance. Um, yeah, so I think, I think calling when it comes to dementia support, if you don't have the background information, try to get that background information.

If you cannot try to call with, uh, at least some, Some sensitivity as well and just explain why you're calling and whether the patient agrees with the referral I always ask have you consented to this referral? You know, do you agree? Um, even if they haven't consented, I'll say okay Well, this is what we can do.

Would you do consent going forward now? And that's up to them to say yes or no If they say no, then I'll also go back to the GP and say, look, this is the conversation we had. Um, you know, if you believe that they really do need the support, I would recommend that you speak to the patient again and, you know, give them the full gist of social prescribing, why you believe they, they want that support.

And ultimately again, it's up to them. Yeah, it's, it's interesting what you're saying about, um, some patients not having consented to being referred to you. And maybe one of those points in the checklist to begin with is make sure that consent is in place. That's very important. I think it surprises me that, you know, GPs sometimes do these referrals without consent, but to an extent, because what I've heard also is that GPs themselves really need to be educated.

Hey, there's. around social prescribing, what it means, what it can do, anything that really concerns social prescribing. So it surprises me, but not too much. Well, touching up on that point with GP's consent, I actually had a patient where she actually consented to the GP and forgot that she had consented.

And so she said to me, Oh, I have no, I don't even remember having this conversation. And then that's when it can get a bit difficult because she's told me the social prescriber she has not consented, but the GP. So we, I have this conversation with the GP. What I tend to do is I'll probably call back.

Maybe they might remember them. or I'll try to remind them. Sometimes it's adamant. I did not have this conversation and I don't want to upset them further. So I just, you know, I would have to just discharge them at that point. Um, would you, I was wondering, would you, in the case of a patient with dementia, would you sort of tend to take ongoing consent in the sense that you would sort of.

re go through consent in different sessions to make sure that it's still in place? Yeah, I think that's actually really important. “There have been times where I have to remind clients who I am really exactly, what conversations we’ve had and revisit those things over and over. So, I start every consultation with a summary of the previous session and end it with recap notes. I ask them to write these and suggest putting them somewhere visible or where they can remember. I also check with them ongoingly that they know that they are with you and that they agree to your support in this journey. I think, so it's, there is about. It's about trying to find the ways to make it as easy as possible. So I think starting every conversation, every consultation with a reflection of the last thing that we said, ending each consultation with that reflection as well.

And if they can, only if they can, cause I know it's difficult with more the, the aging population, the older population. You know, uh, sending some sort of written format or, you know, an email or something where they can see it, you know, or sometimes I might ask them to write some things down. It's so much easier than just sending a text for dementia patients.

You know, you can send a text easily forgotten about. But if you actually ask them to write down this signposting information or write down this number and I might say, okay, so how, where, where can you put it that, you know, you, you go to all the time. So next time we'll speak, I'll ask them to go to that place and then they'll find the letter and they'll find where it's written down.

And. That already helps to jog their memory and so it's just, I think it's just little techniques we can employ to help. It's the little strategies that again, you know, because you have that background and experience, but it's, uh, it's great to hear them from you because again, I can compile this kind of list of strategies for other people.

So this, this was really, really helpful. Thank you for that. I'm, I'm wary of time because I don't want to save too much time for you. Um, so I was wondering, is there any final points that you thought might be important or, or, or, you know, if they come up later, drop me an email. Um, but just to make sure that we've discussed and that you're happy with, with.

What we discussed. Absolutely. I think I'm really happy with what we discussed. Um, I think with the resource that you're going to provide, I'm really looking forward to it. And I think maybe just putting as much also community links and support as available. I think. If, if social prescribers and professionals had a place that they could call for advice, um, you know, an advice line on, okay, I'm working with this dementia patient and they're presenting these things that, you know, I don't know, somewhere that they can call for that support, um, that would be really helpful.

Yes. Yes. And, and in fact, you touched on this and I thought in the, the kind of, uh, resource, this should also be some sort of, uh, links to, as we said before, support in terms of peer support, but also emotional support, because I think it's important. It reflects also on the type of quality of care that, that people can provide.

So I think I, I'm in line with you on this one, and I'll make sure it's in the checklist. But what I'll do is, as we progress through the study, Uh, maybe I'll give you some updates just to, maybe I can show you what we've coming up with, uh, at some point so that we can have that, um, open because, because this was really helpful.

So thank you. Thanks for that. Oh, absolutely. Happy to help. Thank you so much. No, thank you. So what I'll do, I'll just stop the recording, [anonymised].

**SP11**

[Start of recorded material at 00:00:00]

Interviewer: Do you want, [anonymised], to give me a bit of a background about what you do? Because [anonymised] touched on it, but it would be helpful for me to know your line of work.

Respondent: So my name is [anonymised]and I work for [anonymised]. [anonymised]is based in [anonymised] and is a community hub project, a charity of some 20 years existence, came out of a local GP wanting to make a difference that not all People Keeping Well was about medication. A lot of it was about socially prescribing, linking into other things, broadening your own resolution to good health, exploring diet, exercise, all those sort of things, all lift people's spirit, reduce isolation and things like that.

So Dr [anonymised], based on experiences and things around the Peckham experiment that was done back after the war about community, he's been very orchestrative in the area of [anonymised] in making a difference. And embedding other things like this charity, because he started it and the allotment, the community allotment. So I do dementia on the allotment, for example. So I have a dementia group up there which is obviously timely around seasonal, so we just concluded last week.

So, my role is paid for by [anonymised] under the People Keeping Well contract. So for that, there's some hours to do a linkage to meeting people who are newly diagnosed, those on their journey, those who are not yet diagnosed who GPs may refer in via colleagues for social prescribing. It might be something else that's picked up around other illnessses. So diabetes and wellbeing come and see [anonymised], [anonymised] and the team around one to one social prescribing. And out of those conversations, there's things about linking to carers in the wider sense and the condition, for which we know there are many dementias. And Alzheimer's being one of them, it's an overarching terminal illness.

Interviewer: Am I right in understanding that the way it works is you're linked to primary care?

Respondent: Yes.

Interviewer: Remind me, does [anonymised] work for the actual GP?

Respondent: Yes. So, [anonymised]employs [anonymised] and he is also linked to our guru through the PCN to [anonymised]as well. So he oversees some of that wider strategic higher up as well as the social prescribing team here.

Interviewer: So is it fair to say, and I'm talking about a practical example so that I have an understanding. A person goes to the GP and the GP potentially identifies social unmet needs. Although we know it's not just social, you just said it's a holistic kind of thing. He or she, the GP identifies this person needs referral to social prescribing. Would they refer then the person to [anonymised] potentially, and does [anonymised] then assess the person and signpost the person to you potentially? Is that how it works?

Respondent: Yes. If it would be something with dementia, it would come to me. If it was part of, I don't know, diabetes, it might be to the worker that's linked and does the diabetes. If it was smoking cessation and things like that, we have a team that get together and deliver workshops and information sessions and things like that. So part of my remit can come from community. So it can come from somebody seeing my poster for the cafe today and thinking, “Hmm, I'll take mum there. That might be somewhere where we can go.”

It may be [anonymised] picking it up that the person who you've just said has some health determinants, but it's also a little bit about the doctors dealing with that. They're sending them for the scans and the blood. But what also comes up in conversation is they're a carer, their loved one is going to the Memory Services or not yet got there. But, “Can I bring my loved one back because I'm having a terrible time, for the 40th time this morning, I have said good morning. but to that person, it’s the first time they've seen me.” And understanding what that actually means rather than going, “That's 40 times and I'm doing my head in here.”

So it's a little bit about breaking that down. And if I can be as honest as I can, [anonymised] is a very diverse cultural area. So we have got, I think in our local children's centre, 34 community languages spoken alone. So if we look at that and we translate and we look about our understanding, and I think back, I'm 62, I was 62 last Friday, [anonymised]. That's why I was late in getting back to you, but that's another thing.

Interviewer: Happy belated birthday.

Respondent: Thank you. So all that aside. When I think back to growing up and if anybody was given a diagnosis of cancer was almost talked like this, we whisper about it or we go, “The big C.” We're not going to talk about it. So it's been a hidden unspoken about condition that we've got a lot better at being able to be a little bit more open about it, a little bit more talking about it. So on one hand, if that was the diagnosis I was given today, and I'm quite happy to find out what's going wrong with me, because I understand something's not right, and I've got that, then I'm accepting of that, and I'm going to take the decision on looking at what are the options. So it might be chemotherapy, radiotherapy.

On the other hand, I'm going, “I have not got cancer, cancer is not me, I have not got it, I don't want to hear that word, I am not going down this path per se, I'm shutting up. I'm going to look in that corner now and not even engage with you.” So we do not know people's understanding of what that means, their choices and how they want to handle their treatment or how they're going to internalise those messages. So dementia very much falls into one of those two categories. Total denial or the accepting of it and, “Well, let's see what we can do.”

There are people obviously who are too late on and caught too late on in their diagnosis that they don't actually understand what that means. Obviously, given the context of [anonymised], so one of my cafes, I have three volunteers and members of staff who come, who have got community based languages. So I've got a Somali speaker, a Bengali speaker and an Urdu speaker there. So I'm trying to break down the stigma that dementia is not naturally understood or translated. There is not an understanding of it and there's also a stigma of it in that community.

So, it's understanding how that also plays into people's responses, how people do not, hidden carers do not see themselves as a carer because it's what you do for your family. And I've got to say wider than the British population, there is more of that family care in there across all the cultures.

Interviewer: It's interesting because you're mentioning this. Just last week, I was talking to a social prescribing link worker who also operates in a very diverse area and she was pointing out how interestingly she was finding that the clients or patients from traditionally more white British background actually for her presented a bit more intensive support needs. Because of the lack of that infrastructure that you just mentioned. But also I was curious to ask you in relation to ethnic minority communities and communities where potentially that culture background has an effect on the process of acceptance and engagement with the service.

How do you actively address resistance and what can be done that I can include in this resource for people who haven't got that experience that you have? To actually support them to support clients to be a bit more accepting of the whole process.

Respondent: I think that's where I’m very flattered and very lucky to work in an area where we have got a wide base in our own team. So we badged it as Bay McCafe and I do not like to badge anything with anything. It's open to everybody. However, that is the area, [anonymised] is the more intense area where there are people who ethnicity is an issue. And it's a little bit about me being able to say or use those colleagues to help me in talking. And normally it's talking to families in English, the wider set. So it's the elder generation, but it's actually them being able to communicate if they do come out.

So somebody may have come here with English as a second language, learnt, fulfilled their life. And as we know in dementia journeys, things fall off the bookshelf first. So we've perhaps lost that additional language. So it's a little bit about how we can build a resource in to help that and help the stigma that it's not just madness. It's not just somebody going mad or it's an age thing. Because, as we know, dementia cuts across all, it doesn't define itself with one person, one gender. It doesn't take people and people's journeys into their dementia are so unique and personal as well.

You can't just say, “It's a verruca,” and you go and get verruca care and you just dab it on your hand and it'll burn it off and it'll fall off and it's gone. It's not like that. I get where I talk, I'll tell you a bit about that through the referral. I'm just writing Age UK down here to promise. It’s a little bit about helping people understand what it is because if it's one of those, “We don’t’ talk about it,” then it's not understood. Dementia doesn't naturally translate in a lot of languages. And some people do not see themselves as carers in certain cultures. They’d say - this is what you do for your family. Or in a lot of languages there isn't a word for dementia, so it's not necessarily understood what it is. So therefore, number one, it's not understood what it is.

So it's seen as people have gone mad or we’re just not going to acknowledge. That impacts the wider community in retrospect of how that's used and supported. But I do have people come out and it is a very labour intensive two way thing. So from referrals, so referrals I've told you about coming from [anonymised] and the team, by people self-referring into the service, which they can do. But also under People Keeping Well, Age UK make the referrals from people who come from the Memory Services. So these are people who have been on the journey, been into services, had tests done and have got some form of diagnosis, whether it's mixed dementia, frontal temporal lobe, lewy bodies, whatever that dementia.

And it's just helping them, people to know that one, so my hard job is that flat based paper comes through. So that referral comes through and it tells me your name, [anonymised], that I can talk to you and this is your home number, your next of kin, if I need to talk to your next of kin. Or it may tell me, don't talk to [anonymised], talk to next of kin, this is their number, that has been consented to.

Interviewer: And again, sorry, just to chip in very quickly, you get this information regardless of where the referral comes from.

Respondent: It comes through via the Age – if it comes through from the Memory Services, yes, it comes through via Age UK, through the portal, that way. In the referral, it tells me who's made the referral, their contact details and usually a little bit about the situation. It is very dry, doesn't tell me where the person is accepting of it, whether he wants to talk, whether he's going to come out, what his prognosis is, if there's anything else. So then it's a flatbed call.

So what we do is we would send a letter first, so they know who we are, why we're ringing and when we intend to ring. If they've got something in writing, they can pop that in the calendar. If not, we would hope that a family member might pick that letter”. And when we call, we would say “as part of your referral to me, you've consented to me making quarterly telephone calls to help you and give you advice which is an ever changing thing. So I would always encourage you, get a piece of paper, write it down. If it goes out of your head, then next time we call, if you haven't called me and I call you, we can pick up. We need to look at power of attorney. We need to look at how you get your council tax refunded.”

It may come through on that blurb that benefits is an issue, “Can I sort benefits with you? Well, no, I can't because I'm not a benefits advisor.” But what I can do is exactly what the person can do themselves if they've got the information is ring Citizens Advice. Having said that, the caveat to that is beyond COVID it is now the waiting lists and trying to get through is shocking for people. So this is where the system, we've had a dry spell for the period of COVID because not a lot of people were seen and given diagnosis. So the people that are coming, some are very newly on their journey, but some have been in the system and had these without any assistance and been battling. So there's a carer balance there.

We've got to have a healthy carer in order to look after. We've also got to have a carer that if they go down, they know what can be done to secure a passage for their loved one while they're going through their tests or minor surgery or whatever. So that's where my role comes in, in being able to refer into something again, the integrated short term intervention service where –

Interviewer: Just a quick follow up question on what you said about this first call when you introduce yourself. I just wanted to ask, because I suppose it's a phone call, do you ever get any resistance on the part of either the carer or the person living with dementia? And if you do get a bit of an initial resistance, how do you work around it?

Respondent: So you and I, I'm your carer, and you're newly diagnosed. So you and I have had a conversation and I've said, “[anonymised], if anybody rings you, don't be telling them anything. Signpost them to me. Don't tell them your name, don't confirm your address or your bank details or anything like that.” Naturally, the carer wants to protect. So, obviously they want to protect themselves as well because they too perhaps are feeling very, “What does this mean for me and my loved one?”

So when I make the phone calls it's sometimes very dry at first and it's going nowhere. They’d say - Oh, thank you very much, but I’m alright. I don't need anything. So I would say, “OK, so what I'm going to do now is, thank you for talking to me today. I'm going to send you out a pack in which we'll have our activities, posters for the cafe, how to contact me and my name and number is on everything that I send you. Because I've rung you today, my number will show on your telephone, so if you want to save it, and then after this phone call if there's something you think of that you want to ask or you want some advice with or signing on to, like the Carer's Centre. I'm going to put the Carer's Centre information in there, the carer's card. That will all be in my welcome pack which will come to your door.”

Now I'm thinking of [anonymised], I would say to the carer, “Now I've got both of your details.” Sometimes I haven't got the address for the carer. “So shall I send the pack to you and can I send one to [anonymised]?” A lot of people say, “Don't send it to the person with dementia” because they file it straight to the bin. It doesn't even get opened so they don't get to see things. So that's a problem at their end. So what I often do is if it's a yes, I'll send a card to the person with dementia saying hi, “I'm [anonymised]. I'm sending you this. I've spoken to your lovely, whoever it might be, daughter. We had a lovely chat and have a look at the cafe because I think you're coming for a piece of cake.”

So I'll put that in a card, it goes in the pack or I'll just say, “I'm going to be in touch with your daughter,” my telephone number, who I am and a picture of me. Because that's the way that we all like to see who we're talking to. I had no idea what you look like before you came online and you had no idea what I look like. But a great thing about the work that we do and what I do and why I feel my cafes are successful is people get to know this face.

On the phone, you only hear the voice, so if I do that, you've no idea what I look like, who I'm calling. But when you walk in and you see that familiar face, even though it might be two weeks since you saw me last, it starts to mean something in retrospect of, “If only she's going to feed me.” So there's that big thing about trust. And I do think that approach, well, it's the approach is what I'm told is second to none. The level to which I go and do my work is what makes the difference.

So one of the commissioners came out recently to visit the cafe and I said, “It's going to be a quiet day. I have had 16 apologies.” So 26 came, followed by another eight. So the commissioner said to me, “I thought you said it was going to be a quiet day.” And I said, “Well, it was, and we've actually got 34. But today I would have expected the 50.” So I have 13 volunteers who support and I said, “This is the difference.” I said, “If you think this is not quiet, this is quiet to me. What is quiet when you go on other visits to like-minded, like funded projects like ours?”

And she said, “Oh, we can have four people, we could have eight people.” So my cafes are successful. So Monday’s café, we did All the Fun of the Fair. So I did a coconut shy, we did a roller coin, we talked about bagatelles and the old games and the old rides at the fair. They had popcorn and candy floss, the group theme, my sessions, and then I build around that theme. So, yeah, so we looked at all the postcards and the things that bring back memories.

Interviewer: And it's an interesting model because it seems, and correct me if I'm wrong, but it seems that the social is actually the means to the rest as in discussing things around maybe social care, around benefits and all that. It's the key to access people's trust and put emotion in all those conversations and openness, I suppose.

Respondent: Once you get this, I'm not paid to casework. But [anonymised], I know when I come back, I have often eight pieces of work to do because somebody said, “[anonymised], I don't know what I'm going to do. I've just been told by the doctor I've got throat cancer,” and this is a current one. “I've got cancer. I've got to go in and have some more treatment. They're going to do some chemotherapy. My loved one, our daughter lives away. She can't do anything. Our son has nothing to do with us.”

So then you start on the, “Right, what do we need to get in place?” So we need to get a referral into social care. We need to get a referral into the integrated services team for people with dementia. And they then start to look at, are you getting the right level of benefit? Which care home are we going to put your loved one in? And these are the difficult conversations that go back to those dry ones, isn't it? The facing of the eventuality that this is a terminal illness and that the loved one that you married and has now been on their journey for some five years, doesn't actually recognise you or will only recognise you first thing in the morning. And how you are then empowered to look after that.

This person who I'm saying, they made a pact. He said, “Oh, she can't go into care. We made a pact. We made a pact.” “So when you made your pact, how old were you when you got married?” “We were 21, both of us.” So I said, “Well, that's 21 years ago. And you didn't sit there and say, well, we'll make this pact that neither of us will go into care. We'll look after each other, not knowing that these conditions and this situation was going to arise. I often find myself saying - we can either sit and do nothing, or we can start looking at the options. And that puts the person in much more control. If we're proactive, we start to look at what are the options. You're in charge of making those decisions, you're in charge of choosing which facility, whether you can then go and have lunch with you. Because it's temporary, we're not talking permanent here at the moment but we're starting to look at the ways in which you can take control. So you're feeling better about that control and you know you've got it in place.”

“The other hand is, on the other hand, it's taken away from you, because suddenly, today, right now, you're off to hospital and something's got to be put in place as an emergency. You're getting no choice and no control over that.” So it's far better to be in the present and able to try and orchestrate something around this. And that puts the person in much more control and we do some support. I go up and beyond, I've got to tell you, in my role. This is not just a job for me, this is a vocation. I've been a community worker for a long time, having a children and families charity, for which I also dealt with grandparent carers and dementia was an issue there as well.

So, yeah, so care, into education, into from education. In 2000, well 1999, I started a Children and Families Charity in [anonymised] working alongside [anonymised], for which I ended up with nine workers and the model of a children's centre before the government con Children's centres. So personal care, centred care activities that bring people together. You realise that you're not alone. There's other people to share on the journey and we're better by learning from each other because we have an open dialogue in a safe environment.

It can be tricky. There can be times where the person that comes through the door is your next door neighbour and I don't know that you fell out about somebody chucking water on your cat, so you douse them in water. And I pick up that flack and I will pick up that flack and have done.

Interviewer: Well, it sounds as if you have such lengthy experience and expertise in this area that most people, let alone social prescribers, obviously don't have. So my next question is, if we're thinking in terms of very practical advice or pointers that a person with your experience can give to somebody who potentially hasn't got that background of working with people living with dementia. What are some of the practical stuff that people need to know in order to be able to work with this population? Maybe in other words, what are some of the expectations that you have on your own workforce to be able to provide as effective support as you do?

Respondent: So the key for me is the information pack that I send out following those. So if somebody rocks up the café today without a telephone call. There is a pack in my bag already made that I can personalise for that person. Within there is an information sheet, so all the numbers. And it's done in two ways, for you, the person with dementia and for your carer and the impact on that. So what you can do for yourself around power of attorney and what you need to do. Don't recommend any specific organisations as in solicitors to go to or anything like that. Nothing is known, but generic contact numbers for the carers centre, for finding out about the two powers of attorney.

And then what you can do and what you need to do as a person who is a carer or the next of kin for that person. So, where all the local things are that you can signpost to that are up and running. And again, these packs need to be live and current because as we know, the reality is things are here short term, aren't they? The funding that I have got, the last funding I put in takes me to 2027. So I was funded for five years from 2022. So I know my cafes, whether or not, and I'm saying my, because I might finish work at Christmas. That would be nice, won’t it?

But the model is there. And I think the packs and the process of how anybody coming in to do the job, I would show them how [anonymised] does it, the resources that we've got. So it's information central and it needs to be very easy read and contact numbers and useful numbers. You talked to me about the thing about paper based and website based. So we're just doing a dementia survey in [anonymised] Council for the dementia strategy. I have, through my last three groups now, now four groups because it was the allotment, I have got 39 paper based copies because my people do not use the web very well.

So comfort base is paper. I'm 62, I'm of that ilk. I wasn't brought up on computers. I was brought up on an old manual bang, bang, bang typewriter. So my default is, why did I bring my colleague in here? Because Teams never works on my machine. I'm almost never able to get heard when I'm at home. So I brought in my comms person and said, “Just come and watch what I do and that I can talk to [anonymised] today and he can hear me.” So it is all about that.

And I think there's something about if it is a paper based resource, electronically it can be updated quickly, can't it and printed? So it can be done and physically given. It can be given to the person in a format that they can understand and it can also be given in a format that the carer can understand. It can be translated into the natural community language. Although, I've got to say that is my next real step, but I want to do that, [anonymised], when the time is right. And not put energies into things when I'm paid 18 hours and I'm doing cafes and things like that.

My time is precious. Not only that I know today when I come away I'm going to have picked up at least four or five things I've got to refer on. Maybe do a referral, do a referral because somebody's told me they've now got some sort of creams that are flammable. So I'm going to get the fire service to go out and do the home check and talk about how you store these meds.

So it's all of those little bits that all build the bigger picture. And what I could do, I could send you a copy out of what we've got already that we use.

Interviewer: Yes, what would that be?

Respondent: It would be the paper based thing. I could send you a welcome pack.

Interviewer: Yes, yes, that would be really helpful.

Respondent: With my posters and stuff like that.

Interviewer: Yes, please, [anonymised], that would be really helpful. But I take your point about paper based being more accessible, definitely, to people we're trying to support. But also the added benefit, I guess, of having that piece of paper wherever you go. Because there's something about computers, where you have to physically carry a laptop if you need to access a web based page but also the WiFi. So it's not wherever you are, you can access it. With the paper, it's physical with you, it's light, it's usable.

Respondent: Yeah. And as well, you haven't always got WiFi where you are. And in a cafe, if I meet the person who I have said is going to go and have some chemotherapy, it stops the nature of the cafe. So I wouldn't do this in a cafe. When I do the feedback at the end of the session with my volunteers, I ask them to note down anything that they think. So if they had a conversation with the blue chair today and the blue chair said, “I'm really, really struggling. We're not sleeping, we’re not sleeping because the person's now, the clock's gone upside down. We've not only knocked the sun down and know whether it's morning, afternoon or evening, we're actually really struggling.”

So I'm going to ring that person outside the group. So not only is the one to one calls when people come in, there's an expectancy on the grant to ring every three months or every six months, depending on what is detailed at the outset on there. That's not only to update but also check, do a check in, do a temperature check, see how things are, if things have deteriorated, if other things have cropped up and just put that useful.

That's why it's lovely to have had a relationship and have at least somebody see the face because then you know who you're talking to, even the carers. Because a lot of people get very lost in this and it's about keeping everybody well to be able to flourish and not be knee jerk reaction. However, that does happen, doesn't it? But the more we control and the more we plan and the more we think out, the better we are because we know we've got things in place.

Interviewer: Yes, I suppose [anonymised], my wrap up question is, given all these support and services that you offer to people. I wonder within your own locality and based on your experience, has social prescribing got a place? Do you know what I mean? What is social prescribing doing in addition to what you've been doing over the past 20 years or so? What is the place for social prescribing?

Respondent: Right, so forget what I've done over the past 20 years. Because whether you're somebody that's just come from university, it's your first job, whether it's somebody who's a volunteer who's going to get some peer work or some hours to support the charity, everybody needs a starting point, don't they? There is definitely a role for this. It is a one to one role. It is a unique starting point for helping somebody. People not only, I've mentioned cafes and coming up to the allotment and stuff like that, not everybody will come. Not everyone's experienced that, as much as I said, anyway, I'm going to make a reference to.

So if I said to you, “What is your favourite band?” So tell me your favourite band, [anonymised].

Interviewer: I would go with Queen. Queen would be.

Respondent: Queen. So Queen is one that I think a lot of people have heard. And also go have a history. If I said, “My Morning Jacket,” or, “Alan Jackson,” or somebody like that. It might be something that not everybody is mainstream with. So the little bit about this analogy is if we know that there's a connection there. So I can say to you straight away, “It's a brilliant tribute act, and he looked and sounded like, if you closed your eyes, by gum, was he the lead singer? Absolutely. Pitch perfect. It was unbelievable.”

So it's actually having that, isn't it, as a way in? So that's what becomes unique about this role. You find out, the referral comes in, you have this one to one, whether it's face to face. And so for some of our social prescribers here, it is face to face. You have a named go to person and that is key. Absolutely key in a community and a time where social care, the council have been strapped and pushed to the extent. Those waiting lists beyond waiting lists for things. So if you've got somebody that may be telling you that, “Go to [anonymised]'s café,” or, “You need to go and see CAB but I'm telling you it won't be for about six weeks. Their waiting list might be six weeks. But meanwhile, we can do your blue badge for your parking.”

All these things are on that list of what you could look at to start to make a difference. So you go down your physical checklist. “Oh, we've already got a blue badge. Oh, we've already got, we've been put in touch with the carers centre, so I know about carers centre. I know that [anonymised]'s starting on the 7th of September, [anonymised] is starting a carers and cared for group.” So I'm trialling something where I have carers on one side and their loved ones on the others with volunteers.

And I've got somebody joining me from [anonymised]. I'm a Dementia Friends ambassador, so I do the training. So our pathways, all my volunteers follow a pathway. They do the Dementia Friends, the bronze, silver and gold award in dementia. All my volunteers are in that process. So, the little bit about the link worker is I would want that link worker to have done Dementia Friends. I would have wanted them to have it done bronze, silver and gold and have a little bit more of an insight, not at a medical level. But almost have that capacity to talk to people and know an empathy of whether it's affecting, going to affect speech or movement or whatever. To then be able to be better placed and to have conversations with people.

So the link worker is absolutely paramount and they are the signboard with all the, go this way, go that way. Because it's not the client that they meet first, their 10:00 appointment isn’t going to be their 11:00 appointment, isn’t going to going to be their 1:00, 2:00 appointment. So it's being able to be rightly placed to signpost out to what is there.

Interviewer: Yes. I also like what you said about being that one person, that the person living with dementia or most of the times the caregiver can really use as a reference point. I think something that I learned through doing research last year, we did a research on social care in dementia and 99% of people we've interviewed felt lost and didn't know who to turn to for information or anything at all. They would often say, “I feel like I'm in a dark tunnel, I don't know where to go, I don't know what to do.” It's just too complicated and bureaucratic and this tells me that maybe the role of the social prescriber is actually that, as you say, the core person you can go to and talk to.

Respondent: And that's my role. So I say, “I am empowering you with this information when you have – and there is no silly question. But we can start to look at.” So I'm taking today to somebody who said to me, “Thank you for helping. Will you come with me or can you help me find the local care homes?” They actually said, pre caveat to that, “Which is the best care home?” And I said, “I'm not in a position to actually tell you which is the best. These are the ones in the geographical area. This is your next way around because you don't drive. So for ease of access, that first row of three, you can walk to. They're in our community. The others are a bus ride away.”

“So it's a little bit about you going, take your loved one, go for a cup of tea, go and see what the activity co-ordinator's doing, go and get a feel for the place, look at the size of the room, can you go and have lunch there? You can go in at 8:00 and come out at 6:00, or you can go.” COVID skewed everything with people not being able to go and be with their loved ones. It was shocking, a shocking time.

I saw a lot of loss and a lot of damage there with people not doing. But I think it's an imperative role. It is a personable role, and it is one that if you get it right, given you can't be right for everybody, it's not just one person. So it could be [anonymised] today, but when you come back with your workshops, because you're going to invite me to your workshops, [anonymised]'s moved on. But there's [anonymised] in place, so [anonymised] can come along. There's my model here. Sorry, that is not a real firearm. It's a test, if you can hear it. I haven't just vacated the building.

Interviewer: I heard that. But it's the idea of the model and how it's transferable. And what learning points you can actually teach to other localities based on the work that you've done. And actually part of this study that we're doing is also that sharing information and good models of practice. Just yesterday, I told to another social prescriber who's working on dementia review. And how to actually integrate within the annual dementia review, a chunk of time dedicated to the caregiver and the person living with dementia talking to a social prescriber.

These days, dementia review is mostly a 10 minute appointment with the GP. They're looking at new models of having potentially a 30 to 40 chunk of time where you have a dedicated time to spend with a social prescriber so that, again, you can open up those conversations. And letting the person know that there is that reference point in there. So it's an area that's developing over time. But again, part of this study is also to create those links between different models. And it really seems, [anonymised], that the model you just described today in this interview is something that's really transferable. And so again, thank you for sharing with me.

Respondent: Yeah, no worries. I’m very interested in what you do and the outcomes of this.

Interviewer: Yes. In fact, come autumn time when we're going to be doing the workshop, I can get back to you, see if you're interested in taking part. If you're not there, maybe somebody from your team, as we were saying before. But definitely, this is a work in progress and we're looking to involve people throughout to come up with something meaningful. And just on a final note, [anonymised], if you're happy to share that package with me, that would be really, really interesting to, to look at.

Respondent: So your address, is that in the email? I can't remember seeing an address on that.

Interviewer: I'll send it. I'll just send it again.

Respondent: Send it with your postal address because I'm going to do it as a postal one, given the fact that everything is on electric system, so it can be done electronically. I do have some next of kin and carers that do get it electronically. But I do like to send it to the home, so then, I say you've got a talking point, it's like having me with you. “Oh mum, come and have a look at this, that [anonymised]'s sent me this information through. Oh look at this poster, it's lovely and colourful isn't it?” “No we're not going to do that,” “OK, we're not going to do that.” She's actually said about this, “Let's go up to the allotment and if you come up, they’re going to do some storytelling and we're going to do some drawing. Well you're really good at drawing and painting, do you think you might like to do that? Right, so that's at the centre, we'll book on that. OK, so we're going to look at this.” And on we go. So it's a talking point.

Interviewer: It's the physical, it's having a physical copy of things. It's just different. And I can understand how for older generations, it's even more important. I'm 40 myself, but I can still see the difference of reading a book physically as opposed to a Kindle or whatever that is. It's just different, isn't it?

Respondent: There's no comparison, is there? It's the tactileness as well.

Interviewer: Even the smell, even the smell.

Respondent: The smell, the paper, the ink, yeah, definitely.

Interviewer: And for people who are losing cognitively all those skills, the smell and the sensation becomes even more important, doesn’t it?

Respondent: And it just might be one of those things that harks you back. So if you've been a bookworm all your life, that's etched in what makes you feel good. Now the whole thing throughout this, as you regress, this stays very strong. So we've got to appeal to this nature of what makes that person feel good. Because in their journey, we want them to be as well and feel as well as they can.

Interviewer: Yeah, and for example, smell being one of the strongest memories that we can have, it's just maybe the smell of those pages that bring you back to that time, and as you say, gives you comfort and it gives you happiness. So I'm with you on the physicality, that's so important for people living with dementia. It's for everybody, but especially for this population. But this is beyond the point, but by all means, yes, I'll send you my, my postal address. And I guess, [anonymised], I'll let you go and I'll keep in touch with progress with the study and what we're developing. Is that OK?

Respondent: OK, sure. Thank you very much, yeah, really good.

Interviewer: Thank you for your time.

Respondent: No, thank you for making a difference. I'm working with the University of [anonymised] with the music department and musical memories as well. So I've had focus groups here about sliders and the group making music. And when you're newly diagnosed, if we know Queen is your go to that makes you feel this happy, then throughout your regression and when you hear all the tracks as well as other things that that stays with you. And that's transient across whether you're living at home, whether you're going to hospital, you've got something that is a comfort to you and music is a great way of doing that. So I've been working with the [anonymised] on that.

Interviewer: That’s great. Thank you so much, [anonymised]. It was lovely meeting you and, as I said, I'll keep in touch. But for the time being, enjoy the rest of your day and week.

Respondent: Thank you, and you too.

Interviewer: Thank you. Bye bye.

Respondent: Bye now.

[End of recorded material 00:50:54]

**SP12**

[Start of recorded material at 00:00:00]

Interviewer: Everything that you say, [anonymised], is anonymous and confidential. So I will never use your name on any of the documents, just a code that will not identify you, so feel free to be honest. I guess the first question, just to really have an overview from your side on what your role is and in what capacity you support people with dementia.

Respondent:	Yeah, so, obviously I work for SOAR with [anonymised], as you've kind of – Yeah, so she's a social prescriber and we've told you all about that and then getting referrals in through the GPs. So although I work alongside her, I'm a different role. So my role is funded by the council, the [anonymised]. So we cover about a quarter of [anonymised] and then the rest of the areas, there's other people that cover it, that do a similar role but very differently.

We all operate very differently. And the thing about [anonymised] as well is she doesn't cover our whole area. She just covers one primary care network. And they're one of the more active ones. So they're really engaged with us and with the voluntary sector and developing projects and they put their resources into that. So all the stuff she was talking about with dementia reviews and the care co-ordinator, that was quite specific to that PCN, if that makes sense.

Interviewer:	Yes, so you're less involved in that respect, is it fair to say?

Respondent:	Yeah, a little bit, because I'm not based with the GPs. So although I link in and we cross refer, I'm not getting the referrals direct from the GPs most of the time. Sometimes I do pick them up like that, but most of mine come through, what they call, the DAS service which is Dementia Advice Service which is part of the same contract as mine, and the council fund it and it's Age UK that run that.

So professionals can refer in, so most of the referrals come through from the Memory Service which is where people with dementia go to get diagnosed. And then they send them out to us to triage and work with. So my role's not funded by the GP, but then I'm doing that dementia reviews project. I won't go into detail if you've spoken to [anonymised] about it. But doing that with the GP, with [anonymised], so that's been good. But it's a very different experience in different PCNs.

Interviewer:	So, the dementia review we've explored quite a bit with [anonymised], quite in depth and it was great because it seems to be something that's already happening in [anonymised]. And what was interesting, I was telling her that other localities are picking up a bit in terms of expanding the dementia review. But they're not there yet. So you seem to be at a bit more advanced stage but this is about the mentor review. In relation to your specific role, I do see the difference.

It seems that, and correct me if I'm wrong, you have more of a liaison with community organisations and you look more at referrals coming from memory clinics through Age UK. Is that correct? Where she's more probably picking up those coming from primary care services. through the GP in a way.

Respondent:	Yeah, there's crossover, we pass back and forth but it's the referral pathways that are quite different for us. And so she doesn't just get dementia, she gets all sorts, I'm just dementia.

Interviewer:	That's also a very interesting difference because you say your experiences is different from her based on these kind of nuances. And I wanted to ask you, in which respects do you think it's different, to the way you live it basically?

Respondent:	Yeah, as well, I don't just do the triaging and that kind of work and the signposting and all of that. I also run groups. And also we subcontract out to groups, so I work with those partners as well to help them develop their groups and to get the word out. We also do Dementia Friendly [anonymised]. So we just get people together to make the area a bit more dementia friendly. So I'm out in the community like that as well. In terms of referrals, I guess, because I'm quite specialised almost, I see a lot of the same stuff coming through, I guess.

And I'd say, I know the pathways really well. Also, everyone's got a different journey with dementia. So if one person told me something three years ago, and another, it's the first time someone else has said it to me now, I can think, “Oh, well, so and so said that helped.” Whereas, obviously, [anonymised] is quite time constrained. She's got lots of referrals to do, so she might not have spoken to someone with that problem because she's also dealing with the rest of the referrals, I guess.

So that's where we talk to each other. She might come and say, “I've got this situation, can you help?” Or I might go and say, “This person is not looking for dementia services. Could you help see if there's any other groups or anything that might be useful?”

Interviewer:	And do you see any differences in terms of different groups that are referred to you through Age UK, as opposed to the traditional referrals that potentially she gets?

Respondent:	Yeah, so the, the referrals that [anonymised] has come in through, the patient or the client, whatever you want to call them, has to consent to that referral with the GP. So they have to go to their GP and say, “I have a problem. Can you help me?” So the ones that I get through, so they do come from different places and people self-refer, particularly now because I've been doing it for four years. I get a lot of self-referrals.

So a lot of people finding our website or coming back to me after two years, needing something else. But a lot of the Memory Service ones, you still got to go to the GP and ask for help with your memory. So the people coming through are already quite good at engaging with services, I'd say for us.

But a lot of the time, it might not be the person with dementia that's consented. It might be their carer. So sometimes it'll say, “They don't want to talk about dementia, but can you give them some support? Can you not use that word?” Or it might be, “Can you talk to the carer?” So I think we probably pick up a few more people that way, if that makes sense.

Interviewer:	It does make sense.

Respondent:	Yeah, you wouldn't say, “I need dementia help, I'm looking for it” kind of thing.

Interviewer:	Yeah, which brings me to my next question, always thinking in terms of what would be helpful for this workforce to know to be able to work proactively with this population. I suppose you sometimes might get some resistance or initial maybe hesitation in people to get your support. What is some of the strategies that you use? Let's start maybe thinking about a person living with dementia specifically. Then maybe we can talk about carers as well, who has a bit of an initial resistance. Based on your experience, how do you work around that?

Respondent:	So I'm not here to push my support on anyone. You know what I mean? We’re a service, people have got to engage to an extent. They've got to want something out of it. I get quite a lot of referrals through where it just says dementia on it or there's not much information because it's coming through from the Memory Service. They're quite busy, they don't tend to put a lot of details. So I have to be a detective almost and do it.

I do have quite a few conversations where I say, “Oh, I'm ringing up,” and they're not quite sure what it is. Or particularly if they're on the waiting list and they've not had a diagnosis, they're often wanting that medical diagnosis before they get other support. So I just tend to leave them with my number. I might send them out a leaflet. So I've got a leaflet that's got all the groups in the area on and some links. And it’s got a page that's a bit of a checklist of things they might want to think about.

And then our service works for the dementia side of things with the council. So, I just close the referral. But I let them know that they can always come back to us if they change their mind or that we can schedule a six-month callback as well if they want it. Sometimes people just want to know there's that someone if they need it.

Some people, a lot more complicated and complex but that's kind of how we do it, leave it in their ballpark. They've got the number, maybe post out a leaflet and then they can come back when they're ready, I think.

Interviewer:	Sometimes it's about that reassurance of having someone there if you need it. It's not that you necessarily need that intensive support or want that intensive support. And as you say, quite importantly, it shifts across time, doesn't it? It changes a lot. So you might call on that specific day where they think or they do not want any support but it can change. So I understand your perspective of leaving the number, obviously this empowers them as well, doesn't it?

It leaves the choice to them because it's all about that. What was my follow up question? I was thinking, so first contact is mostly done through the phone. Is that correct to say?

Respondent:	Yeah, mostly just because of numbers. I have a lot of referrals coming through and something like 400 in the last year, I think, which is a big chunk of what we do. So I think there's something like 2000, 3000 referrals through. So for one person, it's a big bit. So I do do face to face if I get the feeling that they'll engage a bit better and it will be useful. I sometimes meet people at groups, so if they're not too sure about it, I'll say, “All right, we'll meet at the group” kind of thing. So I do a little bit of face to face, but mostly over the phone.

Interviewer:	What's the group thing? Is it like a session where you explain what the role is and what you can offer?

Respondent:	So it's memory cafes. So we have a lot of them. That's kind of the model we work with in [anonymised] because I guess it's quite cheap and quite effective outcomes.

Interviewer:	And social as well.

Respondent:	Yeah, and the concept is that people would get pushed into the groups. That's what I meant to do is encourage them and then they can get support at the group. So they can find out about services and meet people and chat and that kind of thing. So often there's a barrier there with people being quite nervous or not sure what it is or whatever, that kind of support into it is quite good, I think.

Interviewer:	Yeah, it, in a way, could function as a means to an end because it's through the memory café that people potentially get some more information. They get some initial social contacts. It's all a domino effect in a way if they do access that that context, I understand it. What is the kind of population that you feel is most willing to access memory cafes? And I'm thinking about potential minority groups. Do you feel that they also are quite happy to access memory cafes? What are your thoughts around it?

Respondent:	So our demographic is mostly white British in the cafes and often white British women. So my first part carers group is generally the women are the carers and the men have dementia. So we split up into two, we have a carers group in one room and a memory cafe in the other. I think there's something about men not always being very good at asking for help, particularly at a certain age. And then I get a few more referrals for the one to one support that are different communities.

So in our area, we've got quite a few African Caribbean people, quite a lot of Southeast Asian people, Roma people. I don't think I've had many Roma referrals through but we're quite a multicultural area. And so that's not reflected in the referrals, but there’s definitely more referrals than people coming to groups. But we fund an Urdu and Punjabi speaking group that's run by one of our partners.

So that gets a few people coming through, but they found it really difficult getting people to the group to begin with because they were finding it was women that would mostly come in. And they were finding that women of a certain age in the Pakistani community have worked really hard looking after everyone all their lives, reach a certain age or ill health and think, “Well now I get to be looked after. So I'm going to stay at home and sit and have a nice time with my family.” And don't necessarily see the value of getting out and going to a social group.

So she spent quite a few weeks just trying to get them to see that value and really encouraging them and them coming back. So I know that's been a bit of a barrier. And I think language is often a barrier. So I'm not social prescribing, so I don't have access to the GP interpreters. And even the interpreters aren't always very good to be honest. Language is a big barrier, particularly for older people who may be first generation.

I’m probably bad mouthing the council here, but there's no specific funding for engaging ethnic communities in [anonymised] with dementia. It's just left general. I think there's a lot of stigma as well, people always talk about with different communities. Also, there's been an LGBT older people's group that was set up. I was a little bit involved in that. That's more city centre now. But there's not many people going, there's not really much provision for LGBT people just generally in [anonymised], let alone older people.

So I think. that's quite a big barrier as well. I've not had very many through it all that are out of the closet as such that have told me.

Respondent:	Yes, I think you touch on a very crucial aspect in the sense that minorities are mostly looked at in terms of ethnicity, maybe because it's more visible, maybe because I think it's visibility because older LGBTQ plus people tend to be out. And potential self-advocates a bit less than the younger generation, so that's an issue.

But I do see that there is definitely an issue in terms of representation of sexual minority groups within dementia services. In fact, this is kind of a side thing, but I do lots of research around LGBTQ plus communities in terms of older people within those communities. Whether it's dementia or not, I'm currently doing a proposal around social prescribing for older LGBTQ plus communities. Because I see nationally, there is a gap in the search which translates also into a gap in service provision and visibility is part of that problem. And hesitancy and all that comes with culture and prejudice and stigma.

Respondent:	But even with the visibility and openness and if social prescribers know exactly what they're doing and where to refer to, the services don't really exist either.

Interviewer:	Yeah, do you think it's an issue with demand for these services that is low or offer or is it both?

Respondent:	I think it’s a lot. I think a lot of it's funding. So part of the issue, so this group is maybe going to be shut down because not many people have been coming. It's had two or three people come in. I think they've done a six month trial with it that I helped to set up. And at that point you're kind of thinking, “Well, is it there's not a need? Is the word not getting out?”

Interviewer:	Is this dementia specific?

Respondent:	Yeah, so it started off, so it's LGBT dementia specific, and then they changed it to just be older persons. But dementia friendly is still that focus as well.

Interviewer:	Yeah, it's the same thing that I had to do with my proposal. I started off submitting a proposal for LGBTQ plus communities and dementia. But the funders interestingly found it was too niche, so I opened up to be a bit more inclusive of all older people. Hopefully, they'll not find this to be little value for money, but I see the issue with funding. I can see that.

Respondent:	It came actually from one of my clients who's a very big advocate. He's got dementia and, well, they. It's tricky because I know them in the general sense and then they came out to me and it's very difficult because I do work with them in many different guises. So I have to change what pronouns and names I'm using depending. But they're only out to a handful of people. And it's them that came to me and said, “Can we start an LGBT group?” So it came out of that demand. But I think part of the issue is there's not any resource or any support. So to say, they do up to 25 for young LGBT people.

But nobody's got records of LGBT people. Nobody really collects that demographic data. So nobody's got a list where they can send a leaflet out, for example. Or they can ring people up and say, “Can we do this? Can we do that?” And so, I think that's been quite a big issue, so like I was talking, I run an older, improving quality of life for older LGBT people network meeting.

I'm not meant to, my manager doesn't really like it. But it's just a place for professionals to get together and just have a chat and there's been some good links made through it. But again, none of us are funded for it. So we have an hour where we talk and share information. But someone from the council was sharing their new housing strategy and they're saying in a housing development, I can't remember, like an association or something, let's say it was like 4,000 people. They had four people that had said they identified as LGBT.

Claudi8o:	And this is within [anonymised] which is a fairly large city. So I can only imagine what happens in more rural areas in terms of supporting this population. This is a bit beyond the point of what we were discussing but not really. Because I suppose a proportion of people who are living with dementia are indeed from the LGBT community. So I guess my question to you is, we talked about poor provision that is culture specific. And you mentioned all the barriers with different communities. I suppose, specifically looking at the LGBTQ plus community, how do they really get support when they have dementia?

Is it only informally through families choice or whatever? What is the social prescribing workforce really able to do with these communities?

Respondent:	Not a lot. I think, they're people, aren't they? Older LGBT people are people and have many needs around that. So I support people. It might be they want help with housing or they might want help with the Memory Service or whatever it is. They still want the support that everybody else wants, I guess. So we don't tend to get people ringing up and saying, “I'd like to know about LGBT groups,” or, “I want support around this, that, or the other.” And there's a lot of people I think, who are in the closet, even in their families or they just won't share that with the service because they don't feel like the service needs to know that.

So if you're going to ring up and ask for help about the Memory Service, would you say that you're LGBT? So the person I was talking about earlier, I can maybe link you up if you're not already linked up with them, I think they're really good.

Interviewer:	I'm not sure whether I'm linked up with them. Because of this proposal, I've been linking up with lots of people nationally, but potentially, if they're in [anonymised], potentially not. I haven't, but if you do have contact, please put me in contact. Because if I do get the money for this study, I'm looking to involve lots of people from different sub communities within the umbrella LGBTQ plus community. Because it's going to be a co-design research, because I really want to do something that's meaningful for the community of which I'm part of.

So I really feel that it needs to be co-designed with people. So yeah, if you can, that would be amazing.

Respondent:	Yeah, I'll do that.

Interviewer:	So going back to the point you were just making, does it mean that really, because social prescribing is all about what the person wants, does this tell us that maybe people from that community, from those generations, most of them potentially, do not really want to come out? And hence, maybe there is no place for specific LGBT dementia activities?

Respondent:	Potentially, but I don't think anyone's asked that question, at least not in [anonymised]. That’s not been asked, that's not been researched. It's the same with the ethnic minorities and dementia. So there was a bit of work done. We've had the race equality commission that came out a couple of years ago in [anonymised] or last year, maybe. And it made the council look really, really bad. So they were doing a bit of poking around after that, but nothing much changed. So nobody's saying, “OK, we've got a lot of white groups in the city for dementia. Is that OK?”

Is it that other communities are being well supported by their family and everybody's happy with it? Is it that there needs to be different services? Is a group not appropriate? Is it a one to one service or a service where they do work with the family? There's not that digging into what it is and if there's a problem and if something else can be done. And I think it's the same with LGBT people so I don't really have an answer for you, if I'm honest.

Interviewer:	It’s a question that's not been posed and therefore we haven't got the evidence to suggest either option actually. It's interesting because I was talking as part of that proposal with a transgender woman who lives in [anonymised] in quite a rural area. And we were discussing casually about stuff. And I actually asked the question, “What do you think is the way to go in terms of service provision within your area?” And interestingly, they were saying to me that they don't really want LGBT specific activities because for them, inclusivity means actually mingling with the wider community.

And therefore what they do is to actually try and be integrated into all sorts of community activities. So that was interesting because sometimes the assumption might be, at least it was a bit on my part, that everybody from that community wants LGBTQ relevant activities.

Respondent:	It’s the same with dementia as well, not everybody wants to go to a dementia group.

Interviewer:	But it's important to ask, you have a point in that, because if you don't ask, you don't know. And also that point you raised about visibility is the core issue that then cascades all of these other barriers in knowing what people want. If in primary care services, you don't ask the question about your gender identity, routinely, you don't identify people and therefore you don't ask the question and so on and so forth. So I get what you're saying.

Respondent:	I think particularly when it's intersectional, so I've got two gay uncles who I'm assuming probably wouldn't want to go to a gay group. But one of them is potentially showing signs of dementia and is refusing to go to the doctor. And both of them are being quite closed off, quite secretive about it which you often get with dementia because it’s quite stigmatised. And so there's two things going on there, isn't there, and everything else as well? I think it just gets very complicated sometimes.

Interviewer:	Yes, and we tend to forget sometimes that, as you say, there's the intersectionality of different experiences or characteristics that creates complexity.

Respondent:	I think as well, when you're talking about social prescribing, it sounds like this big, fancy thing. But actually, social prescribing is me and [anonymised] and the rest of [anonymised]'s team referring out. So there's maybe six of us there. Most of us are white. I'm the only one on the dementia service. I've got trans friends who have tried to access or historically who found it very inaccessible. And it's people and particularly for small organisations in the voluntary sector don't always have great training.

Workplace culture can differ from different places. And I think that has a big impact on how people access the service and how they find it when they're in it. And I think that has a big impact when we're talking about things like getting into services and who social prescribing is helping. It very much depends on that team of people and that workplace, I think.

Interviewer:	And I suppose that's why the work that you're doing in your specific role, particularly in the community, in terms of outreach, in terms of letting people know that there is support for them. As opposed to pure access through primary care services, it's really important because there is that resistance. That's why I started off by asking you about whether you found resistance from certain communities.

Because historically, having been stigmatised for decades, even criminalised, thinking about homosexual and the gay community. I understand that the route through primary care is not the only one that can exist because otherwise you're excluding, by default, a chunk of the population.

Respondent:	I think digital literacy as well plays into it. So one of my clients has been trying to ring the Memory Service and hasn't been able to get through. I was able to send an email for her because she can't do online. I think that's a bit of a barrier as well when we're talking about services.

Interviewer:	Particularly, I suppose, with people living with dementia who may live independently, who have less support potentially from family and caregivers. And that we go back to particular communities, I suppose. So the way you do things in terms of sending out physical pieces of information through the mail. I suppose, that could be one strategy to at least combat some of that digital health literacy issue.

Respondent:	Yeah, I think it's being able to do things in a variety of ways. So getting self-referrals, getting referrals through the Memory Service and the Dementia Advice Service, getting referrals from the GP, getting people coming and talking to me when I visit groups. Supporting people in groups, all of that is different ways for people to get in. So out of that, I feel like most people, if they wanted to, could find their way in to the service which is good. And I think it's the same as sharing information. Some people love an email, some people want to talk in detail on the phone, some people want a leaflet, some people want face to face. Some people want that in a group, and being able to offer that, and then I think it encourages a lot more people to engage.

Interviewer:	And I suppose in this respect, the work that you guys are doing in [anonymised], it's reflective of that diversity in terms of access to services and support. Because I've learned through interviews with other people in other different locations that sometimes the access is only through primary care. And as we said before, you're already excluding lots of people because they will resist going to the GP or maybe seeking help. There's all different processes going on.

Respondent:	Well, I've only been doing the job for four years, and I only found out recently that you can do a pragmatic diagnosis. So stuff like that, if you're not in the know, it makes it really hard to suggest that.

Interviewer:	How did you learn about all this stuff? Was it through your own research? Was it through mandatory training? How does that work for somebody who, like you, works in this area?

Respondent:	I think, so I think it's the same for the social prescribers, someone comes to you with a problem and if you don't have an answer, you go and do some research.

Interviewer:	A bit of a reactive thing?

Respondent:	Yeah, but also there has been training. So in [anonymised], again part of the same council contract, they call it [anonymised], there's a company called [anonymised] which is someone called [anonymised]. So she delivers a bit of training as part of that. So we did a sex and dementia workshop which was really interesting, and stuff like that. We do boundaries training and stuff. So recently, different bits of training. I think you pick up different things and we have a link worker meeting as well.

So we have all of us doing a similar role in [anonymised], there’s about 10 of us. We meet up and share information sometimes, so it would be, “I found this service. What did you think of it?” or, “Have you heard about this?” or, “This is the problem we're having.” So I think quite a lot of information comes there. I tend to be quite good at knowing where to go for information as well. And because I've got that development element to my role, I talk with services quite a lot. So people come to me and I'll be part of the conversation about how to get people more involved or whatever.

So I tend to just have my finger on the pulse a little bit there and I've got a newsletter that has 160 people signed up to it.

Interviewer:	I suppose based on what you're saying, there does not seem to be mandatory training. Am I correct in saying that? However, as part of your role, you're pushed into doing training, if you really want to support your clients the best way.

Respondent:	There's not really much where it's like, “Here's everything that's on offer.” But Age UK do a lot of the information gathering, so they've got a [anonymised] Dementia Directory that they have. So stuff can go on there, so they tend to let us know if something new is popping up as well.

Interviewer:	Yeah, and I suppose because the role that you guys have is so wide, it's almost difficult to actually pinpoint what the training should be. Because you could be asked potentially anything by a client, right?

Respondent:	Yeah.

Interviewer:	So I understand that being reactive and potentially, having some background knowledge and connections, as you do. But also being reactive to the client's requests, in the sense that you find out when the situation comes up in a way.

Respondent:	And I don't always know things. There will be new dementia groups popping up in our area, and if nobody's told me about it, I don't always know either. So it's not like I know everything.

Interviewer:	Yeah, so it's an ever evolving problem panorama anyway. You touched on sex and dementia and I was wondering if you know, or if you've heard about [anonymised].

Respondent:	She comes to our Improving Quality of Life for LGBT people meeting actually.

Interviewer:	I was about to say, I can give you the contact but you have it, there you go. So [anonymised] I think I have a very good overview of what you do. And I was really happy to discuss a bit more in depth about particular communities because it's important, I think.

In fact, I was thinking if you have an interest in this sort of stuff, we can keep in touch, see how my proposal progresses. And if I do get the money, maybe there's going to be room for us to potentially do something together or discuss stuff?

Respondent:	Absolutely, we've got someone else, one of the link workers raised it recently because the council are asking for feedback on the dementia services. And they were wanting to feedback about particular ethnic minorities. But I think we're going to talk a bit about LGBT as well. So there's other people with that interest as well. So it might be good to get the [anonymised] lot together almost, if it goes forward.

Interviewer:	Yes. What I can do is I'll keep you posted how it goes. And maybe through Sharron as well, we can potentially start conversations and see where we can go with this.

Respondent:	Yeah.

Interviewer:	But just to say, let's keep on each other's horizon so that we can do something meaningful because it's needed.

Respondent:	Yeah.

Interviewer:	In the meantime, if you do have the contact from that person you mentioned before, to let me have it. I'll be happy to have a conversation with them.

Respondent:	I was just going to say, I know you're asking about information to put up on a resource website almost, wasn't it?

Interviewer:	Yeah.

Respondent:	So I think part of the issue is that when people get diagnosed at the Memory Service, they get given a massive pack of stuff, like really big. It puts a lot of people off. So that's where my role was created from. The idea was that it was someone to help sift through it when you're ready. But I think part of the issue is there's so much information in different places. So I guess my question would be what would be different than say the Alzheimer's Society website resources or Age UK or Dementia Voices?

And sometimes it's just there's another resource website and you're like, “Well, I've already got plenty of resource websites.” And a lot of people want quite local information as well, they don't often go more national. So I think that's just what I'm thinking in terms of like a resource centre.

Interviewer:	I can see that. And our previous project at UCL, which actually give us the idea to progress into this one, was actually about creating a website with resources for social care workers and home care workers. So we have previous experience of that. And this issue of not replicating what was out there was one of the big ones when we developed that website. And the way we went about it was that that website that we developed had a unique feature, which was a toolkit. That's what we call this, which was essentially a personal area on the website where the person could store articles they were interested in and use the articles.

So you had actions linked to a specific article topic. Say, the article was around driving with dementia, it was about a list of actions that the person could potentially do around driving. So for example, contact DVLA to learn about whether your driving license needs renewing or needs to be cancelled, all that sort of very practical stuff.

And just to translate this into this project, we are constantly thinking how to really make this practical and usable as opposed to again, representing loads of information that people already have and are potentially overwhelmed by. So it's important through our interviews to really start thinking about the elements that can be translated into practical stuff for people, which then during the workshop, we will really consolidate. So that we can come up with something that's a bit more meaningful.

Respondent:	It sounds like carers would really appreciate that as well. I can see a lot of my carers where I could give them that link and they could sit and do that action plan almost, isn't it?

Interviewer:	Yes, in fact, what I can do, [anonymised], is when we're finished, I'll actually send you a link to the website that we've developed. Because actually, it's for people living with dementia who have just been recently diagnosed and carers as well. You got separate sections. Maybe you can look at it, see if it might be helpful for you guys as well. But what I'll do is when autumn time comes, and when we’re going to be starting organising the workshops. Maybe I'll get back to you so that you can see how this is progressing. Potentially you can participate in one of the workshops and again, create something that's usable for people, OK?

Respondent:	Yeah. It sounds really good.

Interviewer:	Awesome. So I'll let you go. I won't take any more of your time, but I really thank you for the discussion, that was really interesting. And I guess we'll keep in touch with stuff we're doing.

Respondent:	Sounds really good.

Interviewer:	OK, awesome.

Respondent:	Keep up the good work.

Interviewer:	You too.

Respondent:	See you later.

Interviewer:	Take care, bye bye.

[End of recorded material 00:40:21]

**SP13**

[Start of recorded material at 00:00:00]

Interviewer:	 So the first question is really about yourself, what your line of work, what you do, and if you have any capacity in supporting people living with dementia, how you support them.

Respondent:	Right, so I am myself at the moment, I'm a part time social prescriber, and also part time deputy manager for the service. I have been doing social prescribing for a total of almost four years to be fair. It's coming to that mark now. And I started as a full timer. I've been in the same area throughout the past four years. The way that the social prescribing is provided here in the area that I work, which is [anonymised], we work on a short term basis providing guidance to people, depending on their non clinical needs. Guidance to what support is available in their local community.

I primarily work from home, so a lot of the contact that I have is via telephone with people. And I support people really from a wide range of different backgrounds, different needs, different stories really. But essentially, the main questions we ask is what matters most to them? What is it that they'd like to prioritise, either improve or achieve together? And then we co-create an action plan for them to get from point A to point B. The service, it is an adult service, so we only work with 18 plus. And there's a wide range of referral reasons.

And I guess, well, that joins up really well with more the dementia side of things. So one of our referral reasons is for dementia support. That's, I guess obviously very, again, individual based. But I think what I've noticed more that we get, well from my experiences, we get more people that have a caring responsibility for those living with dementia. I think in my line of work, I've had probably about literally only four people that I've spoken to directly that had a diagnosis of dementia.

Otherwise it's pretty much their family member or their main carer basically that lives with them. Again, it's just very individual based, in terms of the input that I've had. It could be whether they've just had a recent diagnosis, so sending any information for them to understand that a bit better. It could be looking at what benefits or financial support they're entitled to now that they have this diagnosis. And that could be attendance allowance, it could be council tax reduction, etc.

Or just general support. If you have someone that's more, I guess, has more complex needs, more complex dementia, I guess, it could be that they would benefit from a bit of cognitive stimulation. And that could be being at some day opportunities or actually doing cognitive stimulation therapy, depending on their complexity as well. So, yeah, just very dependent on the need.

Interviewer:	Yeah, I suppose it's in line with what social prescribing intends to do, which is really look at what the person wants. So I can understand the diversity of clients that you get. I suppose my next – well, there's lots of questions that came to, to mind after you explained your role. I suppose the first one is, just a point to confirm that also what I've heard from other services is that in the majority of cases, it is the caregiver who actually prompts the referral process.

And I suppose my follow up question around this tiny bit is, do you find in your experience that it is more around the caregiver's expectations of social needs for the person? Or is it really about what the person wants when they're referred to you? Do you see what I mean?

Respondent:	Yeah, that's a very interesting question actually. I think it probably is around the expectation of what the person who has those caring responsibilities have. Because I think, in reality, the conversations aren't one to one with the person that has the dementia diagnosis. So you don't actually get to hear what their thoughts are sometimes. So yeah, it could be the carer's expectations. But I think as well, what we get sometimes as well is that carer burden too.

So it's like a joint approach in terms of looking at things that can support the person with the dementia diagnosis, but also support that person that is the primary caregiver for their own wellbeing to ensure that they continue giving that good care.

Interviewer:	Yeah, I can see that, it's a partnership, isn't it?

Respondent:	Yeah.

Interviewer:	No-one is in isolated silos from the other one. So it comes as a package and it makes sense that you don't, and correct me if I'm wrong, but you don't go down two separate referral pathways. But you actually see holistically what the pair needs.

Respondent:	Yeah.

Interviewer:	I understand that. Yeah. And it makes sense also in terms of saving resources and time. It just make sense to have two separate clients. So the follow up question would be, because you said most conversations are over the phone, right?

Respondent:	Yeah.

Interviewer:	To initiate rapport and follow up. Do you find any barriers in keeping it through the telephone only when it comes to this population or is it working quite well, do you think?

Respondent:	So, as I mentioned, in terms of my line of work, I've only had three or four people that I've spoken to directly that had a diagnosis of dementia. And I did find to struggle over the phone. I think that there were certain communication barriers, obviously. But to be fair though, again, it's very individual based. So most of the people that I had were of elderly population. So perhaps they had other problems like hard of hearing or other sensory kind of issues.

So I did find I struggled a little bit and I think it was also a bit, how do I say this, a little bit difficult from their end as well. Like sometimes they would get a little bit agitated not knowing who I was and why am I calling on a private number. I'm saying that I'm from their GP practice, but am I really? There's also that fear of scams as well for a lot of people.

So yeah, there was a bit of agitation. In terms of speaking primarily with the caregiver, it has been OK, actually. It can be a lot better sometimes via telephone, because there's so many appointments involved within their care sometimes and that admin side of things where they have to take them to the hospital.

Sometimes having that outside of home trip can be difficult just for a social prescribing appointment where I don't need to physically inspect the person. But then I can see the advantages of that as well because then you're face to face with the individual themselves So there's pros and cons to both.

Interviewer:	It's a mixed bag, but I can see the advantage of not having to travel somewhere. I see this in research as well when I interview caregivers, it's their preference usually to go remote because they can attend to the person they're caring for. For example, if they're staying at home, and it saves resources and time to everyone. So I can understand the advantages that come with that.

It's interesting what you pointed out about when talking to somebody living with dementia as a client, the potential resistance sometimes that you get or hesitancy to engage with you. And I wonder, because you seem to have quite a bit of experience for years, it's not a little time. What are some of the strategies that you can use when you find that initial resistance when dealing with the person living with dementia? Do you find that something works a bit better than something else?

Respondent:	No, that side of it, I can’t honestly think of something really from the top of my head now. Something that's worked actually, I'm currently supporting someone living with dementia. I think whenever they hear their GP practice and the specific name of their GP practice rather than saying, “I'm calling from your GP,” they feel a little bit more at ease to discuss things.

I think sometimes as well, describing your role rather than saying the name of the role can be helpful because for a lot of people when you say social prescribing the first thought is social worker. So, they get a little bit worried, like what does that mean? So sometimes saying my name, GP practice, what I do for the GP practice can be helpful.

But I have a client at the moment that I'm supporting her, and she does have a diagnosis of dementia or suspected, I think. And it's helped, she's quite Internet savvy so she can use the email and she's got that skill and she's able to communicate by email as well. And that's helped a little bit because she can see the email thread that we've had. She can remind herself of the conversations before as well.

Interviewer:	It's interesting because one would assume that, or maybe it's the case that most people from that generation, particularly when they develop dementia, they're not Internet or computer savvy. But it's interesting to see how for this specific client, it actually played to her advantage in terms of having a thread of what was discussed, having it all in there. So I can see that. And also, I agree with the other example you were making about being very practical about what you do as opposed to presenting just your title. Because lots of people do not know what it is.

And this is beyond dementia. Social prescribing, as a terminology per se, doesn't really reflect what you guys do which is so wild.

Respondent:	Yeah, completely.

Interviewer:	Yeah, and people assume it's social care or some people told me they assume it's about prescribing medications because of the prescribing word. So I think it's a good way to go, to be specific about what you can do for them. And I'm asking you these very practical questions because thinking about the resource. One potential way for us would be to create some sort of, in a way, checklist with strategies that somebody who hasn't got the experience that you have already with clients might have hints and tips as to what could work to get that engagement from the person.

So once you've established contact with your client, how do you understand what the person wants? And I'm asking because I suppose sometimes the person might not even know what they want or verbalise what they want. What is the process? Do you use any techniques or just maybe describe me how do you go about asking someone what they really want?

Respondent:	What they really want? Of course, the first starting topic of discussion is just the initial reason of referral. So just hearing from their side, whether they agree with this and really reassuring that this is what the GP has brought to my attention. However, it may not be correct or it may not be what you actually want to prioritise. So, discussing that in more detail, if they agree with it and they think that's the primary kind of reason, fine, we can go into more detail about it.

So asking for specifics, is it more the social aspect of things, the emotional aspect or actually is it more financial, housing, etc.? I think, there is a little bit of – just in general, like speaking with any client, because of the lack of knowledge around social prescribing and really just the lack of personalised care sometimes within healthcare in general. You find it that a lot of people are a bit like, “I don't know what I need. No-one's asked me this before. I have no clue where to start.” So it's always like the first bit of discussion, just hearing their background story. Just hearing what's been happening for the past six months, if anything or just having that like verbal vomit sometimes can help them.

And for me to summarise things helps. So when they do speak for quite a bit, and then I notice certain themes within what they've brought up, I always summarise it. And I ask them whether they agree that these things are there or whether they feel like they need to correct me in any way. So, if someone's talking about feeling low or things like that, I'll be like, “OK, so there's a need clearly for emotional and wellbeing support. Do you agree with that?” That kind of thing.

Interviewer:	It's sounds a bit like almost therapy in a way, counselling. The kind of way that therapists use that technique of almost reflecting or mirroring what the client suggests when they're telling their story. Something like, “Oh, from what you're saying, I can hear that maybe you're a bit struggling in that area, is that correct?”

Respondent:	Yeah.

Interviewer:	Sort of that reflection process. And I really can understand you saying that the first thing is to ask for them to tell their story. Because obviously, sometimes what I hear is that in referrals you actually have no details whatsoever. Sometimes it's just really basic and therefore, for you to be able to understand how you can support that person is really asking them about their overall story.

So I think it's a great strategy. And interestingly, one thing that you mentioned about really that reflection process is in line with a bit of motivational interviewing. Another social prescribing link worker I talked to, I think she's from – what service was that? I think [anonymised]. They actively use motivational interviewing in the process of really understanding what the client wants.

And part of those four steps, I think, is reflective practice. So it's in line with that. And the follow up question is when you talk to a client with dementia, when you hear their story and you hear information about them, have you ever encountered, in your experience, maybe with your current clients, instances where you're a bit like, “Hm-mm, is this information reliable? Is this accurate? Is this what's really happening? Or is this part of that cognitive deterioration? Maybe I should talk to somebody else.” Does that happen?

Respondent:	It does sometimes. But I think more in the sense that they state, so they will state that they don't need any help at all or that they're absolutely fine and they can manage everything. When a week ago, when the GP did a home visit, for example, they saw clutter, for example, or they clearly needed that support. So I think that's when it's a little bit tricky to have those discussions where they clearly state they don't need any help. But it's been brought to the attention that they do from other people

And that could be again caregivers. It could be neighbours. It could be GPs, etc. So I do have with this lady that I'm currently supporting, so as I mentioned, it is I think suspected dementia. At the moment, she doesn't have a diagnosis. But her family members are very extremely worried about her safety. There's instances of neglect and just home neglect as well. And when I first spoke to her, she just said she doesn't need any support. All she needs is a cleaner.

I had to do a bit of digging around because the next call, she then said that someone turned up at her house unannounced. She didn't know who she was, didn't know where she came from and who organised it. But stated they’re a cleaner and they want to help her. But didn't realise that she had dogs and the whole thing basically, didn't do the work. So I had to do a bit of research behind in the background to find out where that came from. And it was done through adult social care, and it's actually a care agency.

But the terminology that they're using is to ease her in, so it is just primary for cleaning. But instead of saying, “I'm a carer,” saying, “Actually I am a cleaner, I'm here for the purposes of cleaning,” she's accepting that help and that support. And actually, she's very pleased about it now, where she wants for that person to come in every day if they could. So it is, I think, just using terminology a little bit differently fit their needs.

Interviewer:	Yes. And was this person who went to provide support arranged through you or was it –

Respondent:	It was through adult social care. The GP did a, I believe, a safeguarding referral just because of the clutter and the neglect.

Interviewer:	And it's interesting that somehow you were not up-to-date in relation to that. So the GP didn't communicate that.

Respondent:	Yeah.

Interviewer:	OK, that's interesting. It's part of what I hear about the organisational issue with GPs as well, in terms of educating them. It’s that part of the process that I hear in different services. Do you generally feel that GPs have embraced social prescribing, that they know what it is, what it can do?

Respondent:	In our area, I would say definitely. There's 26 of us at the moment.

Interviewer:	That’s lots.

Respondent:	Yeah, there’s a lot So clearly they like us. But we always try our best to actively engage and to show the value of the service. Whether that is attending practice meetings, answering any questions or sending updates to practice managers. I think as well, what you'll find, of course, I'm sure when in other discussions, there's loads of staff changes within GP practices that happen quite quickly sometimes. So it's always being aware and raising that knowledge. So we have flyers and we have leaflets that the practices can have for their staff member to remind themselves of what we do.

I think the one thing that I will say, sorry, just on the topic, I know it might be a little sidestep. But just on the topic of communication and speaking to the organisational communication. I think one thing that I do struggle when it comes to referral for a client with dementia is it often reflects how secondary care and primary care aren't as well linked as they should. So, for example, you might find no information around the actual input that the memory clinical service has had on that client. And you could end up duplicating work.

I think as well, what I've tried to help anyway, especially in more of the caregiving side of things is, so I've had a meeting with the Memory Clinic. And I took notes literally step by step what a person should expect as soon as they're referred. Because the issue is people are on a waiting list and they don't know what to expect next. So that's helped the caregivers have a reassurance and understanding of what the next steps are, what support they're entitled to, as well as the person living with dementia. So yeah, sorry, that was just like –

Interviewer:	No, it's really, really important to know. And actually, just to clarify. The referrals that you get can only come from the GPs, is that fair to say? Or can Memory Services refer people to you guys?

Respondent:	No. So it has to come from the GP practice. But that could be any member of staff within the GP practice?

Interviewer:	I'm asking because in other revision models, [anonymised], they have the possibility to have referrals from secondary care services as in memory clinics as well. And in fact, in some services, self-referrals as well from people. So they have either a website or people can just drop in and self-refer. And that's a question for you, do you think that somehow that could sort of alleviate these issues in terms of lack of communication between primary and secondary? Or actually do you think that that would only increase workload for you guys in terms of, do you guys have the resources to then attend to all these referrals coming from different sources? What is your take on this?

Respondent:	So just like you said, the answers will be anonymous, so just transparently speaking, just from my own experience here, where I work now. There has been an opportunity to have a self-referral pathway. And we did pilot it. We did that within one specific GP cluster to see how that affects uptake capacity, all of that. And there hasn't been a massive increased workload. There hasn't been this massive capacity But I think that's because it's been a pilot, it was still very much kept within the GP practice.

So in terms of marketing and advertising, it was just still done quite internally. For example, in waiting areas, rather than doing it, sending it to the [anonymised] Memories Clinic, for example. Now, we’re very positive towards self-referral. I think it should, me personally, I think it should be there for every GP practice that I work for. I think that gives a really good opportunity for people that fall within cracks or that don't necessarily engage well with their GP practice to still get the right support.

But the conversations behind doors in terms of the actual GP practices and their opinion on self-referral is they don't want it. They don't want to open up the doors to the service being used by secondary care. Because financially, they're the ones paying for it in that sense. It's not secondary care. So there's been very apprehension basically towards the self-referral or opening it up to different services.

Interviewer:	They've been very, very cautious because of financial aspects as well. I can see that. I also totally agree with what you said before in terms of self-referrals somehow compensating for the fact that, particularly in certain communities, the reason that willingness or openness to necessarily engaged with primary care and GPs. So in the examples that I've heard from other services, that was a way to look at needs from particular communities.

And I know [anonymised] is a very diverse area because your colleague touched on that as well. And I was wondering, my question at this point is how well do you think you guys, given the current pathway to referral, are able to attend to the needs of diverse communities?

Respondent:	As in, sorry, it that more –

Interviewer:	Yeah, this is in relation to, say, ethnic minority groups or it could be LGBTQ plus. Although from experience, when it comes to dementia, those generation are less open to come out. So maybe it's less about sexual minorities but maybe more about ethnic minority groups. Do you feel that the services is prepared to support people living with dementia from those particular groups?

Respondent:	Honestly, no, I don't think it is prepared, just complete transparency. Because I think only just now, as more of a wider conversation or discussion, only just now it started to focus on culturally appropriate social prescribing. And how to have those conversations with people to see what they need in terms of being culturally sensitive. So those discussions where I work have only just started. But I think just from knowledge in terms of services, so in general, services that I know of that are available in [anonymised], yes, there are some support services that are specifically provided for ethnic minorities.

But in terms of dementia support, it's more just wider support rather than for a specific ethnicity or for someone with a specific, I guess like protective factor in that sense.

Interviewer:	Background, yes. I understand what you're saying. It's essentially if a person is referred who's from an ethnic minority, they would still be conveyed toward mainstream dementia.

Respondent:	Exactly, yeah.

Interviewer:	Support in the community.

Respondent:	Yeah, so the conversations are starting internally but I think there needs to be an external conversation as well in terms of the provision of the services and how they support each person and their individual need. So, yeah, because I haven't come across, maybe I just didn't do my research properly. But I haven't come across a specific dementia service for one group of people.

Interviewer:	So maybe it is, as you say, a wider conversation, a wider change in terms of community provision for specific groups. It's not necessarily down to social prescribing or primary care. Because I suppose the approach in some respects is the same because you look at the individual. So when a client comes to you, regardless of their background, you're looking at what they, as a person, need. So it's then down to what's provided in the community, actually, whether it responds to their unique needs or not. I can see that point.

In your experience, have you come across lots of people or anyone from different ethnic backgrounds? Or actually, let's expand this to not necessarily ethnicity, but any minority group in general. And how have you supported them, if it happened?

Respondent:	Do you mean specifically with people living with dementia?

Interviewer:	Ideally, but if not, maybe let's think about the wider population.

Respondent:	So just one, I guess, example to give of a person living with dementia is someone from a different cultural background, that their main spoken language is not English. And they struggle, so that's a barrier for them. And the support that I was offering was through their son, so the primary caregiver. I referred onto adult social care and I ensured to say that they would need a carer, if possible, that speaks their mother language, basically, to ensure they get the right support. And that communication is there basically for that person.

In terms of the wider aspect of social prescribing and again, like being culturally sensitive or just in terms of a minority. So I think sometimes I struggle to bring up the conversation myself. So, most times I expect the client to bring it up. So, let's say, I've had people, like you said, from the LGBTQ plus community that has openly mentioned it to me. And then that's when I would prompt to ask whether they'd want to look for any activities or support from that community.

And then they'd say either yes or no. We do get a lot of asylum seekers and refugees within our area. However, I would say it's 50%, I'm not saying this is exactly the correct percentage. Just from experience 50% of clients are willing and happy to engage with community service that are part of their own culture. But 50% of them just say no. And that's because a lot of them are obviously fleeing conflict, they're fleeing trauma. And they don't want or they don't have that trust towards their own community sometimes.

But yeah, this is why I said we've noticed that there was a lack of culturally appropriate awareness within our team. And we're going to put on a bit of training on how to have these open discussions with people to see whether they want to engage with something particularly from their group or not. But I do find it, myself personally, I do find it a bit of a struggle sometimes. It's quite difficult. I've had sometimes, people, this is a bit of background thing. But during COVID, I supported the vaccine hub and I was doing the admin side of things.

And one of the questions was, “What is your ethnicity?” And some people just got a bit – they were like, “Why? I don't want to answer that. Why do I need to answer that? You don't need to know that about me.” And it's the same way of like if you ask someone, “What's your religion?” They sometimes say, “I don't want to give you that information,” which is fine. Sometimes I struggle to bring up the conversation myself, so most times I expect the client to kind of bring it up because you don't want to cause that kind of conflict from the beginning.

Interviewer:	No, I understand that. I find myself in a similar situation with demographic questionnaires. You filled one out for my study. So we ask that question routinely and I've had lots of participants not answering certain questions, particularly around ethnicity. Sometimes around gender and sex, if they're included. So I understand that it's sensitive topics for people. And because of that, it is difficult to be proactive and bring up the topic, particularly in the context maybe of an opening session over the phone with somebody who doesn't know you and you don't know them.

But at the same time, I also think sometimes it might be the crucial information for you to support that person. And hence, I understand your point about potentially getting some training in the future around at least knowing how to maybe approach those conversations. Because I agree it's important. OK, [anonymised], I think I have a good overview of how the service operates in [anonymised] because I've talked to other people.

And it’s been really an eye opener because I think you guys are different to other services, particularly in terms of the workforce, how large it is and how it operates. So it was really great. Do you have any final points that you wanted to mention before I let you go?

Respondent:	No, I don't think so.

Interviewer:	If anything comes to mind, drop me an email. But for now, I think we can probably stop our transcription. Let me just hit the stop button.

[End of recorded material 00:37:16]

**SP14**

[Start of recorded material at 00:00:00]

Interviewer:	So, can I just ask you, as an opening question, a bit of a background about you, what you do, and what’s potentially the pathway within your service, to refer somebody living with dementia to social prescribing?

Respondent:	Yes. I mean, I’m a consultant psychiatrist for older people, as well as Clinical Director for Older Adult Services. So we’ve got – I mean, we don’t call them link workers, but what we have is slightly different. So, we’ve got [anonymised] Well-being Services, so where there is a care navigator, so they will navigate the person with dementia as well as the carer in between the services, and help them with some of the social activities, in joining groups, and also sometimes creating some activities for these people, if they don’t already exist. So, that’s the purpose. So, they’ll talk about dementia, what support is available, where they can access support, make the referrals if needed, get them in touch with various social prescribing groups, or social activities that are going on.

Interviewer:	I see. And what is the pathway for a person to be referred to the service? Is it only through primary care? Is it different ways?

Respondent:	No, I mean, anybody can refer people with dementia to this service, but in memory service, we’ve got these people actually sitting in some of the memory clinics. So, when the person attends the clinic, so they have a chat before the clinic, and also after the clinic, once they have been diagnosed with dementia. But, because of the limitation of the number of posts, they used to do it at most of the clinics, but now they only do it for some of the clinics.

And, for the rest of them, we make – it’s like an internal referral. We’ll let them know that this person’s been diagnosed with dementia, and then the care navigator, the [anonymised] Well-being Service care navigator will get in touch with them and then have a discussion around dementia, what are the support services for dementia.

Interviewer:	OK, and are the care navigators part of primary care services? Is that a role within primary care?

Respondent:	It’s beyond primary care, because it extends to all the services, so it’s not part of primary care itself, but it’s a kind of a specialist service which has been created for people with dementia. So, they’re not part of secondary care, they’re not part of primary care as well, but it’s more like a voluntary sector organisation, but obviously, in terms of funding, it comes differently.

Interviewer:	OK. And I suppose the idea is that they will know what’s going on in the community, and be able to refer the person to –

Respondent:	Oh, yes, yes. They’ll work with all the organisations in terms of health and social care.

Interviewer:	OK. Do you find, that this kind of system works within your area? Do you know any other systems, and do you find any differences in terms of how smoothly it operates?

Respondent:	I mean, it’s very smooth, because as I said, they’re based, and they come to our clinics, so they speak to the patients. So, when they come to a clinic, they don’t need a formal referral, they’ll just go and speak to the person with dementia, as well as the carer, and then get them in touch with all the social activities, social groups that are there. And even when they’re not there, it’s easy to refer to the care navigators so that they can get in touch with these people.

Interviewer:	I suppose it relieves some of that pressure that, sometimes, services has, because it’s sort of automatic, almost.

Respondent:	Yes. Yes.

Interviewer:	I understand that. Based on your experience as a psychiatrist, and I suppose you work with people living with dementia, so is that –

Respondent:	Oh, yes, yes, yes.

Interviewer:	What do you think are some of the barriers that are distinct for this population, and when it comes to supporting them? And I’m thinking both presentations of symptoms, I’m thinking communication, all those sort of issues, that it’s important for the social prescribing workforce to know.

Respondent:	Oh, yes, yes, definitely. I mean, they’re aware of these kind of challenges that exist with these people, so they are happy to visit them in their own homes as well. So, they make contact over the phone, but then make a home visit if needed, and then get in touch with them.

Interviewer:	OK, so the system actually allows for in-home visits as well.

Respondent:	Oh, yes, yes, yes.

Interviewer:	Do you know if there is a time limit to the support that you provide to people living with dementia?

Respondent:	No, no, there is no strict time limit as such, so they can be in contact say for a few months, or longer if needed, so depending on the complexity of needs.

Interviewer:	OK, that’s great.

Respondent:	And even if they’re not in contact, the person with dementia or the carer can get in touch directly, and then get help in future.

Interviewer:	And I suppose that the kind of support that’s provided to people is holistic in the sense that it involves the caregiver as well.

Respondent:	Oh, yes, yes, yes, yes, absolutely, yes.

Interviewer:	OK. Can you identify if any, of course, any kind of gaps or limitations within this system of care, particularly relating to social prescribing?

Respondent:	Yes, I mean, in terms of social prescribing, obviously we’ve tried to create some holistic groups, so not just dealing with the physical health aspect, but also with the mental health aspect, so that’s where we did what’s called as Healthy and Happy classes, so that focusses on both physical fitness, but also on other things like relaxation, tai chi, yoga, those sort of things. But it's not widespread, so there are some pockets here and there where it happens. So in terms of organised activity, which is structured, and then you measure the outcomes, in these Healthy and Happy classes, the outcomes are measured that it has gathered, but in other areas, it could be in a dementia café or in a day centre where this happened, but it’s not measured, it’s not structured. So, we’re not sure how much of it is physical activity, how much it is other relaxation, mindfulness, those things of activities.

Interviewer:	So it’s difficult to measure impact in some other of those support services.

Respondent:	Yes, yes.

Interviewer:	What kind of outcomes measures do you have specifically for this group that you’ve created?

Respondent:	So, they’re called physical health outcomes, so they measure things like blood pressure, pulse, those sort of things, I think they measure well-being as well, so when we were doing it, the well-being was measured, in terms of carers.

Interviewer:	OK, and it’s also for the caregivers, is that correct?

Respondent:	Yes, caregivers, and the person, yes.

Interviewer:	Yes, OK. Do you find that – because in some of my other interviews in different services, what we’re finding is that sometimes in terms of attendance to these groups, it’s difficult for particular communities to have access or attend these groups. Do you find that happens in your groups as well?

Respondent:	Oh, yes, yes. I mean, it is a problem, so commuting can be a problem, so sometimes a person may refuse to attend, so that’s a problem. So, some of them – I mean, the retention can be a problem, so they might attend for a few weeks, and then stop attending then.

Interviewer:	OK. Do you have any particular strategies or advice to potentially increase attendance?

Respondent:	So, we’ve got the voluntary organisations who can help older people, so if they have problems with transport, or if they need any additional help, then they can go and speak to the person and help them in terms of these activities.

Interviewer:	OK. And do you find there are any cultural barriers to people being willing to join the activity groups?

Respondent:	Yes. Yes, I mean, some people obviously, they don’t like mixing in groups, so they prefer to be on their own. So, what we said is that when we created the Healthy and Happy classes, there’s a quiet hour as well, where they can do activities on their own, but there’re group activities if they want to join the group, so it’s a combination of both. And then, obviously, for group activities, there’s language barriers, so some people don’t speak English, so they might feel left out. So, I mean, that’s still not ironed out, but they can join the quiet activity, they can join the quiet hour, but obviously joining the groups, that’s still a problem.

Interviewer:	Still a bit of a challenge, OK.

Respondent:	Yes.

Interviewer:	I suppose, do you find that sometimes the caregivers are a bit more skilled in English, and therefore they can sort of translate and support the person with dementia? Does that happen?

Respondent:	Yes, I mean, where possible, the caregivers will go and they will help out, but obviously the caregiver, if they also can’t speak English, then it becomes a problem.

Interviewer:	Yes.

Respondent:	So, we’ve tried to establish some activities for ethnic minorities who don’t speak English, but it hasn’t yet happened, but hopefully sometimes in future, we’ll be able to generate some activities just for ethnic minorities who don’t speak English.

Interviewer:	I see, OK, yes. It’s an ever-evolving landscape, isn’t it?

Respondent:	Yes, yes.

Interviewer:	So, it’s a process of looking where the needs are. OK. Is the area where you operate quite a diverse area in terms of population living with dementia?

Respondent:	Yes, yes, I mean, in terms of the diversity, it’s not huge when compared to London or Manchester, or from some of those areas. Probably it’s a bit similar to UK as a whole. So, yes, we have got about five percent ethnic minorities, probably, living in [anonymised].

Interviewer:	I see, OK. OK, this gives me a bit of an overview about the kind of service that you provide, so that’s very helpful. I’ve been speaking to other people from your locality specifically, so I have further in detail information about the kind of service that you provide. From your own perspective, was there anything that you think it might be helpful to add to these conversations? Because, I’m quite happy about what we’ve discussed, and I don’t want to take much of your time.

Respondent:	Yes.

Interviewer:	So, purely from your perspective as a psychiatrist, is there anything that comes to mind, or you’re happy with what we’ve discussed?

Respondent:	I mean, I’m happy with what we’ve discussed. Now we are trying to focus on dementia prevention, so that’s become part of the strategy, so we are identifying people at high risk groups, and then trying to provide social prescribing activities for that particular group. So, that’s now under the planning, so hopefully we can have these activities for dementia prevention as well, not just after dementia.

Interviewer:	That’s very interesting to hear. So it’s a bit more proactive work.

Respondent:	Yes.

Interviewer:	What are some of the activities that you envision might be part of that prevention programme within social prescribing, for example?

Respondent:	Yes, I mean, it could be – it’s basically targeting the risk factors for dementia, so based on physical health, based on – some are based on mental health like depression, some are based on social isolation, social factors, and then it could be hearing impairment, sensory impairment, because they’re all risk factors, so addressing these sort of things. So, obviously, if you have classes that target both the physical and mental health risk factors, then you will be able to address those risk factors, and hopefully reduce the risk of dementia, may not be able to completely prevent dementia in some cases, but obviously looking at reduction of the dementia risk.

Interviewer:	Yes, which will have an impact on service pressure as well.

Respondent:	Yes, yes. Yes.

Interviewer:	So it’s kind of an investment in a way, because it will pay back.

Respondent:	Yes.

Interviewer:	I see that. OK. That’s new to hear, and that’s quite important. All right, so Arawin, if you’re happy, I’ll just stop the recording.

Respondent:	Yes, yes, that’s fine.

Interviewer:	OK, so let me stop this. OK.

[End of recorded material 00:13:22]

**SP15**

[Start of recorded material at 00:00:00]

Interviewer:	Can I ask you, as a first question, a bit of a background about you, what you do, how you support people living with dementia, if you do support them, just to get an idea?

Respondent:	Yes. So I’ve been a social prescribing link worker for about 2.5 years or so. I started as a care coordinator, so that was just to kind of link in with all of the different services that were involved in somebody’s care, identify any gaps, and help them to engage with the services. Pull MDTs together, so pull everybody around a table to discuss the individual’s support, and again, identify any gaps, and for people to take away actions so the person was front and centre of the support at all times.

I then became, about a year ago, a health and wellbeing coach, so that’s kind of my remit at the moment. I work now fully remotely, and I leave in December, so December 31^st^ will be my final day. Only because I’ve relocated. I’ve relocated to [anonymised], so –

Interviewer:	I didn’t want to intrude, but thanks for clarifying that.

Respondent:	Yes, no, I relocated to [anonymised], and so everything is remote now for me. I would say my involvement with people with dementia, I haven’t had that many, more your Parkinson’s, that would be more people I have supported with, because I think there is a lack of – I suppose there is the lack of training and the lack of knowledge of really, we would see what other support was in place for them, and signpost them to other places, because actually, we don’t really have that much knowledge and skillset around supporting people with dementia, or what that looks like.

I think we tend to really talk to the family members instead of that person, because generally, at the point they come to us, they don’t really have capacity to make those decisions. So already, when we get the referrals sent through, or the ones that I’ve experienced, it’s been the daughter, the son, the partner or parent has permission to speak on their behalf, which is ...

I mean, I’ve got a gent who’s just been diagnosed. He has frontal temporal dementia, and I have them on – I speak to his wife, but he’s in the background, and then she has it on loudspeaker. So as much as possible, I try to include him in the conversation, but he’s really difficult to understand because it’s over the phone, so probably, maybe an in-person visit would’ve been better.

So, yes, no, I find that I speak to the relatives more than I speak to the individual which, I don’t know, I think mainly the referral should be in for the person who’s support – for the carer, because we tend to discuss – I think it has to be a wraparound care thing, rather than it being an individual being referred, maybe as it’s the family, or that initial person who’s with them 24/7 to do them as a couple, or as a twosome, almost.

Interviewer:	Because, I suppose there is a bit of a tension there, because the service is supposed to be pretty much person-centred, but as you say, because it’s the caregiver who actually does the talking, and potentially a discussion on what’s needed, it’s actually probably a client service for the caregiver.

Respondent:	Yes.

Interviewer:	I see your point, and it’s come up with other people I’ve spoken to. So, because of the remote nature of the service that you’re providing, and by the way, this is sort of in line with other people I’ve spoken to from other services, do you think it is effective with this specific population, and if not, is there any room for effective social prescribing for people living with dementia?

Respondent:	I would probably sit with not as beneficial as maybe a face-to-face or a home visit, or a visit in the clinic, so you can see the person. It’s really difficult to understand the gent, but I think there is, if I was in person, I could see his lips move, and I could maybe make out a little bit more, or also, have him – his wife has him write things down, that help to tell her, because I think she struggles with him as well, so I could have that. I don’t know whether it would be more of a communicating online, maybe. I don’t know. It depends again, doesn’t it, with dementia, how computer literate can somebody be with dementia, and what that looks like?

Interviewer:	Yes.

Respondent:	So, yes, I think remote working over the phone is probably more difficult than face-to-face. I think the benefit is their face-to-face, and I am very lucky with my team, I could say, “Actually, I think this gent needs to be supported face to face, and transfer over.” But then you get the issue there of being transferred to too many different people, and that can also be unnerving and unsettling for people who need a consistent base, really.

Interviewer:	Yes. So, correct me if I understood wrongly, but you’re saying that if a person comes to you and is a client living with dementia, and you realise that there’s more intense needs in terms of face-to-face support, do you refer the person to one of your team, and then they would be able to visit? Is that what you’re saying?

Respondent:	Yes. Yes, that would be what I would do, if I felt that the gent I’m currently supporting – it’s all right, because I have his wife that speaks very well for him, and that’s fine, I don’t think that needs to happen, but I think if, first of all, when we get the referrals through from the GP practice and it states dementia, I probably would not take that person moving forward over the phone. It would be discussed as a team, and then whoever could see them face-to-face or have them in their clinic, then that would probably be allocated to this individual.

The reason this couple came through was more of a signposting anyway, with regards to benefits and stuff, of what they were entitled to, rather than his care, because actually, his wife’s happy with the way the care is for him, but they were just – weren’t sure about accessing benefits, and what that needed – what that looked like. So, I suppose also it depends on what it is that they need support with, as well. A bit more intense would be face to face, but that signposting of speaking to the loved one, is easily enough done over the phone.

But again, I do think, I always do feel, not just dementia, but anybody who has a lack of capacity for whatever reason, where you’ve got to then speak to somebody on their behalf, or people who don’t like to speak on their own behalf, I always feel like, actually, I’m not offering that person-centred – I feel like, I don’t know, it feels a bit cheeky, talking about somebody rather than them already being involved in their own care.

Interviewer:	Yes, no, I get that. Going back to a point you were just making before, you were saying that there’s different types of support that you provide. It seems that when it’s more kind of light touch as in finances, and that sort of things, it’s one type of support, and then there’s a bit more of an intensive support. And I’m talking not just people who are living with dementia, I’m just trying to understand if you feel there’s different types of support levels that you provide depending on the person’s needs?

Respondent:	Oh, yes, definitely. I think sometimes we get just a simple signpost where, for example, for the benefits example, if they don’t really know where to access, or they’ve never claimed benefits in their life, then it’s a case of having a conversation. It’s still very person-centred, and I still do a full, holistic, wraparound overview of where they are, what their support network is, what they like to do, and get a feel for the person, but actually, what matters most to them at that point in time is actually. “I’m really struggling financially and I need to know where I claim benefits.”

And then it would be a signpost, or get Citizens’ Advice to give them a call, and get that ball rolling. For other people it can be, especially with health and well-being coach, it can be, actually, somebody who’s really entrenched and wants to lose weight, but doesn’t have a first clue where to start, and actually, sometimes, it is baby steps, really. The first goal that we might agree is that they'll answer the phone to when we make an appointment, and that really is a lot sometimes. I mean, we have some people, the GPs will refer through, for people who are not engaging with their annual reviews or are not filling their prescriptions, and we need to understand why. So again, understanding what’s going on for that person, and what’s going to help remove those barriers to access their medication or to access their annual reviews.

And, we work really closely with the GP practices in that respect, of saying, “Actually, this person doesn’t want to attend their annual reviews because you always invite them on the morning,” and actually, it’s an afternoon, but they don’t feel comfortable enough, or actually can’t get through to the GP practice, because that’s also a barrier sometimes, so they just put it off and just think, “Ugh, I’m not bothered. I’ll not bother. I’m not doing it.” So, yes, it can be really – we get a lot of people come through with substance misuse, so a lot of that is to help them engage with the services that are out there, and identifying any barriers, and really walking alongside them, offering just that support, to say, “Actually, we’re here at the end of the phone, or we can meet in person.” Not me, but the team.

Yes, and just, again, it’s just based on what matter most to that person, really, because sometimes you get a referral through, and it’s not – what is most important to that individual is nothing to what the referral speaks of, because actually, OK, they need to fill their prescription, but actually, the reason they can’t fill their prescription is because they can’t afford to get the bus to go down, so actually it comes back to finances, so let’s explore that. So, it really is, no matter what they get referred into, the overview of that holistic, wraparound care, of everything that’s going on in that person’s life, I dip into and have a conversation, and then we go, “Right, OK, so what is important? What are the goals? What are we going to work towards?” Irrelevant if that’s just a simple signposting.

Interviewer:	Yes, you’ve made two very interesting and novel points that I haven’t heard before. One is quite interesting, you were talking about the example of somebody not engaging in the annual review, or talking about a person who’s just not engaged with the taking medication process, and it’s interesting because it kind of feels a bit medical, in a way, as if it’s the GP’s remit. But it also feel a bit social, because as you say, you discovered that the actual reasoning is then on your side of things, if that makes sense.

Respondent:	Yes.

Interviewer:	So it’s difficult, what I’m saying, sometimes to actually decide what’s medical and what’s not medical, I feel. But also, an interesting point that you were making is about trying to, for some people, to promote the engagement in services. And, you mentioned the example of substance misuse. I wanted to ask you, given your experience, when do you feel that the legal responsibility of a social prescriber, or a health and coach well-being, care coordinator ends and starts, particularly when it comes to these serious health issues? What’s the boundary? When do you feel that you’ve done your job, it’s down to the person or the service they’ve been referred to? How do you think about that?

Respondent:	Yes, it is a grey area. I think, so for me, I’ve had a couple of gents who have had serious alcohol misuse in the past, and they’ve really wanted to get some support, but actually, first of all, are not knowing where those services are, so my job was literally to explore with them the different alternative services in the northeast, and then once they had all of the information, which one, made a decision, who did they want to be referred into? And, I placed the referral on their behalf, and then they were in touch.

And then for me, then there was just a follow-up conversation were planned in for, like, two weeks’ time, when I knew they’d have been in touch with them, followed up with the referrer just to say, “I’ll just make sure you’ve got the service, to make sure that you’ve got the information, and that somebody’s been in touch.” “Yes.” And then, I speak to the individual to say, “Right, I’m aware that they’ve been in touch,” because some people will say, “Ooh, they haven’t bothered.” Like, I know they have, so again, what are the barriers to you engaging?

Or, actually, it’s just a simple check-in to say, “So, you’re involved with NTRP. NTRP have been in touch and you’ve got your first, initial assessment with them. Are you happy then for me to step back? And then, if you do need any further support, in future, you know where we are, you know how to be referred in.” A lot of people are happy with that, because actually, they don’t want to speak to multiple people about the same thing over and over. That’s boring and repetitive and annoying. And then, they are aware how to be referred back in, in the future, if they feel they need it.

There’s a couple of people who’ve wanted an extra check-in just after the assessment, so they’ve said, “Actually, once I’ve had the assessment, are you OK if we check in after that?” And then I’ll explain to them, “Yes, but once you’re engaged with NTRP, that is the service that you need to be engaged with, and actually, everything I would do, they will do. So, they look at finances, they look at housing, they help with all of that. So actually, it’s just basically, you would have, essentially, two social prescribers involved –

Interviewer:	Yes, it would be – yes.

Respondent:	And I think for me, it’s just being really honest and upfront with the individual that you’re supporting. Being really honest that we’re not here for long term support. That’s not what we are. “It’s about being really honest and upfront with the individual that we're not here for long term support. We're not mental health professionals, counsellors, or the befriending service. My job is to help them in to engage with the services that are specialised and other professionals in that field, so I'll be doing them a disservice if I just keep them on long term. That's really harsh to say to somebody who is isolated, but really important.

Interviewer:	Yes, I would agree, the risk of dependency could be there, right?

Respondent:	Mm.

Interviewer:	Particularly thinking, again, to people living with dementia, for example, I’ve been involved in studies where the intervention was to provide physical health and exercises in the home of participants for a period of time, and by the end of the support time, they were kind of developing those dependency issues, and anxiety relating to the termination of rapport. So, I can definitely understand where you’re going with this. I wonder if you – I know you haven’t had much experience with clients with dementia, but I’m sure you can think of some particular barriers when it comes to having that population engaged in whatever you’re suggesting, based on their needs, and what’s some strategies that you would use to actually motivate, in a way, them to engage, if you’ve got any pointers?

Respondent:	I think for me, and not just with people with dementia, but I suppose that’s your cohort, isn’t it, that’s who you’re thinking about?

Interviewer:	Yes, but don’t worry. Don’t worry –

Respondent:	I think with anybody that I try and engage with, I try to make – I explain the service right from the beginning, and what it is I can and can’t help with, what my parameters are, but also, I get to know that individual, not as a personal way, but because we try to build the rapport, and use a lot of open questions, and hold a lot of space for them to explain what their current situation is. But I’m very clear, we’ll find out what’s going on for you presently, we’ll find out what needs to happen in the future moving forward, and we will come up with some goals, and we’ll have a look and see what you want to work towards. And, my job is to help work alongside you, with that.

But, I think by showing a little bit of humanity and kindness, and a little bit of an in, so for example, if they’ve got a dog, I’d ask about the doh, oh, what’s its name, what kind of dog is it. And then in the next call, I’m mindful to say, “How’s [anonymised] doing? Have you been out on a walk,” just as an in, so it’s not specifically like an interrogation, it’s more like just having a laid-back chat with somebody about what’s going on for them, but actually, how we can work to improve that.

I do have a strong boundary, as well, and be like, “Right, OK, we’ve made these goals, and this is what we need to work towards.” So, sometimes, the first goal that we make, that we agree, is that they’ll answer the phone to my – when we put an appointment in, they answer the phone, because we get a lot of people who don’t answer, and DNA quite often. So, that’s the initial small goal sometimes. And, I think barriers to it, for me – was that the question? What was the question, about the barriers?

Interviewer:	Yes, if you find particular barriers that you’ve struggled with when it comes to having people motivated and engaged in the process.

Respondent:	I think the main ones are people who are, “Been there, tried that, I’ve done that. I’ve done that before,” and so my question is –

Interviewer:	Disillusioned, a bit disillusioned about the whole thing?

Respondent:	Yes, so there’s some people who just want the, you can wave a magic wand and they’re going to lose six stone overnight, that kind of, don’t want to take accountability for, actually, their diet is poor and they’re not exercising. So, with them, it’s about exploring in a motivational way: how long ago did you try? What's different now? Would you be against trying that again? Let's have a little look at amending what you tried in the past. Let's have a look and try. Or, amending that, adapting it, adapting it slightly,” whether that’s walking. “Oh, I can’t run.” “OK, then, what can you do?” So, people who are entrenched and not motivated will look for any excuse. Actually, OK, you can’t do X, Y and Z, but could you do A, B and C? What does that look like? And, let’s have a look.

Try and have people to understand What would that give them? What would their life look like? What would the difference be for their families? What would they observe if everything was different, and how they wanted it to be? And that sometimes is the clincher with some people, because actually they don’t think about how it affects their friends and family around them, and they haven’t actually sat with that for a little while, but actually thinking, what would my loved ones – what would my husband see in me? What would my wife ... ? Or, how would it benefit our relationship?

So, yes, I think it’s just finding an in, some common ground with people is important, and building that rapport is really key. Phoning when I say I’m going to phone. I’ve worked with people in the past who don’t, they don’t keep appointments, and that can be really disheartening for somebody who’s trying their hardest to engage. So, when I say I’m going to ring you at two o’clock on a Monday afternoon, I’m going to ring you at two o'clock on a Monday afternoon. And I’ll be available, if you can’t make it, we can rearrange. And, I think that flexibility for people as well is really important.

So, when I ring for the initial – to book the initial call in, I always say to them, “If you need to rearrange, that’s absolutely fine. You just send me a text message,” because some people don’t want to speak on the phone, either, so I think offering alternative forms, whether that’s WhatsApp or text message, and offering them that, saying, “If you need to rearrange, then just reach out, and I’m flexible with my diary. We can plan in another time.” Obviously, I don’t have that happen too often, and if it’s somebody who rearranges multiple times, then I know I’ve got to have a very different kind of conversation with them. But, I think just offering that flexibility of approach as well is important.

Interviewer:	Yes. I suppose your experience as a counsellor, because you said you’re one as well, I suppose it really helps. There’s lots of social prescribers who potentially don’t have that background. And I can see in the strategies that you mentioned, that there is your background as a counsellor, in terms of motivational interviewing, almost, to build that motivation, in terms of almost kind of Rogerian, person-centred approach, where you validate the person, and you start with building on small goals, and then you build up, and you build up.

Respondent:	Yes.

Interviewer:	So, it’s these things, [anonymised], that are very helpful, because you use them, but some people might not be aware of them. Or maybe they use them, they can’t name them, so it’s important to, yes, to come up with these strategies that people can learn to use, so it’s very helpful. I’m just wary of the time, because it’s five to, and I’m really sorry that it was so short, but it was helpful because you’ve given me a lot.

Respondent:	Good.

Interviewer:	So, I thank you for your time, and maybe there’ll be an opportunity for us to have a bit of a catch-up or a follow-up as we progress with the study. But, I’ll keep in touch via email, so I can update you and we can maybe find another time to continue, if that’s OK?

Respondent:	Yes, that’s absolutely fine. No problem.

Interviewer:	Awesome. Thank you so much, [anonymised].

Respondent:	Take care.

Interviewer:	Take care. Bye bye.

[End of recorded material 00:21:53]

**SP16**

[Start of recorded material at 00:00:00]

Interviewer:	... ask you to begin with a bit of a background about what you do, what your role is, and maybe if you support people living with dementia, in what capacity. If you don’t, don’t worry.

Respondent:	OK. So I’m one of two social prescribers at the [anonymised] PCN, so we support three surgeries in the area. Dementia-wise, we do get referrals, but it’s mainly, “Do we know of any groups that family members can take their loved one to?” or support for the family member that’s looking after the person with dementia. So, a lot of our role is, it’s kind of generally researching what’s available to pass on to family members, give them information like Dementia UK, where they can talk to an Admiral Nurse, that type of thing. It’s not one of our top areas for referrals. I would say mental health would be.

Interviewer:	Is a big one?

Respondent:	Yes. Weight management, that sort of thing. But yes, I suppose if someone, a loved one wanted respite care from the person with dementia, we can refer to the relevant services, that kind of thing. In rural [anonymised], because [anonymised]’s massive, there are prescribers that do work more with dementia patients because there are probably more of them. People go to retire up in rural [anonymised], so they – I know the girls up there do put groups on for these patients. So, our contact, I suppose, is quite limited. It's basically referring on.

Interviewer:	Yes, it’s more about signposting, isn’t it?

Respondent:	Yes. Yes, definitely.

Interviewer:	And, you would say, [anonymised], that one of the reasons is because, as you said, there’s probably a smaller population living with dementia in your specific PCN compared to more rural areas.

Respondent:	Yes.

Interviewer:	That’s quite interesting.

Respondent:	I think we’re one of the smaller PCNs, as well. A lot of the other girls, they’ve got six, seven surgeries, but there’s just me and Jo with the three surgeries. I mean, we’re busy enough, but yes. There is one day service that has closed, where we did used to provide information, so the nearest one would be in [anonymised], so that’s about 15 miles away. So, there is a lack of groups, day groups that people can go to –

Interviewer:	So, based on your knowledge, I suppose, say for example caregiver of somebody living with dementia is referred to you as a social prescriber, and they’re potentially interested in some social activities in the community, are you saying that you would potentially struggle to find something local for them?

Respondent:	Yes, there’s a couple of groups. There’s a singing group, but again, I think it’s Age UK used to run one but that’s not available anymore. So it’s just, there’s this one that people can go to There was a day centre that used to do sessions for half a day, and there’s one other group at the local library on a Friday.

Interviewer:	OK.

Respondent:	Yes, but it’s not much.

Interviewer:	Is it fair to say that if a caregiver comes to you, before being a caregiver, they are a person, and I suppose you could signpost them based on their needs, to not – not necessarily to dementia-focussed groups or activities or resources, I suppose, right? Say they have needs around, I don’t know, mental health or respite, there’s always ways in which probably you can signpost them to other services, that’s what I’m saying.

Respondent:	Yes, yes. Yes, definitely. Yes.

Interviewer:	And another point that you raised that’s quite interesting, [anonymised], is that you mostly get referrals, correct me if I’m wrong, for the caregivers as opposed to the person living with dementia, is that correct?

Respondent:	Yes, I suppose, so the caregiver might’ve gone to the doctor, or district nurse, and said, “I’m struggling with my loved. I’m quite low because I’m providing all the support. I don’t know what’s available.” So then, they would refer on to us and say, “We’ve got this patient who’s struggling with their loved one with dementia.” So, then we would give then a call, we can go out and have a chat with them in the home environment, and just see, get a better picture of what’s going on at home, and again, either refer on to – they might need adapting equipment, so then we can refer on to social services for an assessment.

Because, sometimes it’s quite a – my stepdad had dementia, and my mum just had no idea what to do, and that was down south, and there really wasn’t – down in [anonymised] on the coast, and there really wasn’t much of a pathway for her to say, “Right, your husband’s been diagnosed. Here’s some information.” A lot of the time it’s just a shock, I think, for the loved one. They don’t know what to expect, or what financial support might be available. So, again, we can then give them information, and ...

Interviewer:	I completely agree with you. I’ve interviewed lots of caregivers in my previous study, and what was coming up over and over, particularly as soon as diagnosis happens, is that people just feel lost, and really don’t have a clue what’s out there for them.

Respondent:	Yes.

Interviewer:	So, could it be maybe, within your PCN, you’ll tell me about that, that people might not necessarily be aware of social prescribing? Do you think that social prescribing is a thing that people know about?

Respondent:	No. No. We do as much marketing as we can. We’ve got boards up in all of the surgeries, it’s on the website of the surgeries, but yes, a lot of people don’t – or they hear the word, “Social,” and they think we are a social –

Interviewer:	Social, yes.

Respondent:	– as well, so it’s quite a long job title, social prescribing link worker, so normally, we just say, “We’re the link worker from the surgery,” and go from there. Yes, but I wouldn’t say – yes, we still get people who are like, “Oh, we didn’t know you were there, we don’t know what you do.”

Interviewer:	Yes. I suppose even if people might not know, if they do have an issue and they go to the GP, it’s usually the GP that would refer them, and they will learn about the service, by default, in a way.

Respondent:	Yes.

Interviewer:	Yes, and it’s also interesting, what you said about terminology, social prescribing link workers, because more and more, people are telling me that this kind of title doesn’t really make much sense to people.

Respondent:	No, no.

Interviewer:	Who confuse it with social workers, social care workers, but also with prescribing medicines sometimes.

Respondent:	Yes.

Interviewer:	So, what would an ideal title be? Some people tell me community navigator. You said yourself, “Link worker,” right? How do you –

Respondent:	We just say we’re the link worker, because basically, we’re here to link people in with services. We’re not support workers, which a lot of people think we are, but it’s hard not to get dragged in, and if we get someone that doesn’t have anyone, you know ... So, we do – it’s a bit of a – it evolves, I think, sometimes, the job role, with new things, that probably, we get involved where you shouldn’t, but you can’t not support someone, if they’ve got no one. If we’ve got a couple that, their loved one does have dementia, or their family, as families live all over the world, don’t they, now? And, they’re kind of, their friends tend to disappear, and that sort of thing, because they feel uncomfortable, or don’t know what to do to help. It can be quite frightening. So I think it’s nice for them to know there are services, be it limited, that can support them.

Interviewer:	Yes. But this makes it tricky for you guys, doesn’t it? Because obviously, you’ve got limited resources, being two people for three PCNs, with a wide range of populations and patients to support. And, I suppose it makes it a bit difficult, as you say, to draw those boundaries, and not overdo, because through human empathy, we’re drawn to help people, but then you also need to draw those boundaries.

Respondent:	Yes.

Interviewer:	How do you explain that to people? How do you – do you actually draw those boundaries?

Respondent:	Yes, we are getting a bit harder now. Now we’re getting – we’re out of lockdown and we’re getting really busy, whereas during lockdown, it was a lot of welfare calls, checking people were OK, they had food, they had medication. So now, it’s kind of, that is, when we get referred someone, when we do our home visit, or if they come into the surgery, we do say we’re not a fulltime support service. We will link you in with other services, and then you have to engage to move on with them. So, yes, I think we’ve just go to be a bit harsher, I suppose. But then, there is the odd person that doesn’t have anyone, no friends, relatives, you know, that you might do a little bit more for.

Interviewer:	Yes, I suppose in some respects you assess the situation, and you maybe prioritise those cases that need a bit more intense support.

Respondent:	Yes.

Interviewer:	I can understand that.

Respondent:	And I think it’s looking at that person as an individual as well. And I think it’s quite a caring role, being a link worker, because you get to read the background information on where they’re at, from all the information at the doctors’ surgery, and some people do have horrible journeys, and then you have to help as best you can.

Interviewer:	Yes. In terms of the information that you get on a referral, I’ve heard from different people, it’s again very different from services to services.

Respondent:	Yes.

Interviewer:	Sometimes you get lots of details, sometimes you get the word, just, “Dementia,” and there you go, and you have to figure out all the rest. What’s the situation within your PCN?

Respondent:	So, we just, the GP or whoever, will just send a task from their patient notes. You click on task, most of them are all right, brief, but you know, yes. They’re all right. There’s a couple from one surgery that are quite limited with their – like, dentist, well, what does that mean? So, yes, but generally it’s all right, because we send regular updates on what we’ve supported with, and reiterating what we can support with. Like, we don’t support with PIP forms, because they would just take forever. So, it’s just – because we’ve had a few referrals where the patient’s said, “The doctor said you would help me,” well, no. It’s kind of just rewording it as well.

Interviewer:	Do you find that sometimes there is an expectation on your role, and potentially the support that you can provide, that’s a bit – I wouldn’t say unfair, but that there’s an expectation that doesn’t really match reality of what you can do for the person?

Respondent:	Yes, sometimes.

Interviewer:	OK.

Respondent:	Yes.

Interviewer:	Because, I’ve heard about that as well, so that’s why I was asking. In terms of the referral that you get from the GP, I suppose it’s not necessarily good to have just a few words, like dentist, for example, but I also suppose, sometimes, what people have told me is that you may get referral for a reason, and then by talking to the person, you realise maybe the need was not that superficial one, there was something behind it.

Respondent:	Yes, yes.

Interviewer:	So, I suppose sometimes it’s difficult even for the GP, within those few minutes, to get what the real reason might be, or maybe they just have to take it at face value, what the patient tells them.

Respondent:	Oh, definitely. Plus, you might get someone that’s just having a bad day, and when they’re going to the doctor’s, and the doctor says, “How are you?” a few patients said, “Oh, I’ve just let it all out.” So, at that time, that patient was really upset, but by the time they’ve been referred, and we call them, they’re like, “I’m all right now. I just had a moment.”

Interviewer:	Yes.

Respondent:	But, you’re right, yes, GPs don’t get long to assess, I suppose, in great detail. It’s just really what that person’s telling them at the time.

Interviewer:	Yes. And when you said you get this kind of, I wouldn’t say refusal, but you call people and they tell you, “I’m OK, I don’t need support. It’s all fine. It was just that day,” how do you understand whether that’s the real reason, whether there’s a bit of resistance that needs working around, based on your experience, how do –

Respondent:	Yes, it would just be, yes, talking to the patient, I suppose. But, I suppose we do have the luxury of looking back at patient notes as well, to see how often they’re in, what their major health conditions are. So, sometimes you just, you can, I suppose, delve a little bit with the phone call, or say, “Look, let me send you – I can text you my contact number, and you can just give me a call if ever you’re feeling you’re going to have a wobble again, just give us a call,” which people do. You’d just get a random call.

Interviewer:	Yes. I suppose that’s a good strategy, because it will let people know that there is support if they need it. You give them your number, they know that it’s there, you’re available. Maybe gives that reassurance that –

Respondent:	Yes. Sometimes people just know there’s – need to know there’s someone there if they need, and then quite often, people don’t. But it’s nice having that fallback, I suppose, if someone’s having a really bad day, they can give us a call, and we can either day, “I’m coming out and have a chat, or you can come in and have a chat,” see if there’s anything we can provide for them, information or a referral.

Interviewer:	Yes. Yes, yes, I think it’s important. And I was wondering, in terms of the referral process, within your PCN, do you only get referrals from GP surgeries, or can people self-refer?

Respondent:	No, they can self-refer, yes. Yes, we’ve got, there’s a database up here, it’s called [anonymised] frontline, so what people do it, they register and they put what service they’re looking for, and their postcode. They click on the link, and it bring up all support service. So, we’ve registered on there, so people can refer through that database as well.

Interviewer:	OK, so I guess my follow up question then is, do you see any differences in terms of the kinds of patients that you get through self-referral, as opposed through the GP, in terms of diversity of different groups? Do you see differences, or not much?

Respondent:	We don’t actually get many people self-referring, if I’m honest. It might be – the receptionist sometimes here comes out and says, “I’ve got this person who’s upset in reception,” and we’ll go down and have a chat. There’s a few oldies that come in, and they’ve got their appointment wrong, and it’s a different day, and they get upset. And I suppose it takes the pressure off the girls downstairs, as well, so if we’re free, we can just go down and have a little chat with them and see if there’s any other issues, as well.

Interviewer:	That’s an interesting role as well, because I suppose –

Respondent:	Yes, we do quite a lot, really.

Interviewer:	Yes, wow. You’ve got that supportive role for the receptionists as well, I love it. Social prescribing for the receptionist.

Respondent:	No.

Interviewer:	Oh, gosh. What are some kinds of skills that you then need to be able to do this work that you do?

Respondent:	What, particular skills?

Interviewer:	Yes, like personal skills. I mean, obviously training always helps, but I was wondering, because for example, the receptionists sometimes resort to you, you must have that added skillset, and I was wondering, what are some good skills that social prescribers should have?

Respondent:	I don’t know. Just be flexible, I think, because you never know what referral you’re going to get, and just being good at researching as well. [anonymised] and I are quite good at – [anonymised] and I have worked together for years. We worked together at our previous job roles, after just – we’ve been here since 2020, so –

Interviewer:	Together, that’s nice.

Respondent:	Yes. Yes, yes. She’s my wing woman.

Interviewer:	Nice. How lovely. Maybe team effort as well, maybe that really helps.

Respondent:	Yes, exactly, you’re always bouncing ideas off each other and saying – or you’ve supported someone as much as you can, and then there’s physically nothing else you can do, they might not engage with services you’re referring on to, and then I can say, “Have I done everything?” And she’ll be like, “Yes, you’ve done everything.” Because sometimes you can’t. There’s only so much we can do, if people aren’t going to use the information that you give them, then you can’t. That’s all we can do, really. We can sort of – we’ve both got a couple of patients that kind of, rather than would call their social worker, they would call us to have a meltdown, but perhaps that’s just because we’re nice.

Interviewer:	Yes. Yes, yes, yes. But I suppose you’ve got a point, in the sense that there’s only so much that you can do, and at some point, it’s the patients’ responsibility to actually follow through.

Respondent:	Yes.

Interviewer:	And it’s not something that you can instil in them if it’s not there. But, I also think, quite importantly, what you said about bouncing off ideas and giving each other potentially an objective perspective on what’s happening always helps.

Respondent:	Yes.

Interviewer:	Because, I’ve spoken to people who work a bit more in siloes, a bit more isolated from each other, for example from home, there isn’t that rapport with colleagues, and you can tell that sometimes they would need that support, just a shoulder to cry on, or just somebody to chat with.

Respondent:	Yes, definitely.

Interviewer:	So, yes.

Respondent:	Plus, we have monthly meetings with the other social prescribers via Teams, just to say, “Are there any new groups that we need to be aware of,” or it’s just sharing information amongst ourselves. We meet, we have a six, seven-weekly meeting over at the library, the community – just seeing what’s going on in the community with the girls over there. So, there’s quite a lot of information sharing, or we might get an email, “Girls, I’ve got this patient who wants to access, I don’t know, a reading group, have you got any information?” And vice versa. We’re always asking them for things.

Interviewer:	Yes. I suppose it’s very helpful when you don’t necessarily know everything about every little service that’s local.

Respondent:	Yes, yes.

Interviewer:	So, I can see the importance of that. So, you said you don’t necessarily support lots of people living with dementia, because of the locality you’re in. I was wondering, [anonymised], do you have any contacts of people who actually work a bit more rurally that might support more people living with dementia?

Respondent:	Yes. I can email them to you, if you want.

Interviewer:	That would be –

Respondent:	I need to – [anonymised]’s back tomorrow. Her memory’s better than mine, and we can go through and just email you some names if you want, yes.

Interviewer:	Yes. Yes, if you could, just because I think there’s lots to be learned from people who support populations also rurally, because I’m in [anonymised], and we’re interviewing all across England, but we’re a bit underrepresenting rural groups. So, it would be great to hear what these guys do, so yes, if you have some emails, I’ll drop an email, ask them if they’re interested, and we can take it from there. That’ll be –

Respondent:	Yes, we’ll ask them first.

Interviewer:	Yes, yes, sure, yes, yes, absolutely. Yes, but thank you for that, that’s very helpful.

Respondent:	No problem.

Interviewer:	I suppose I have an idea of how your service operates. I’ve got also an idea on the types of populations that you support a bit more than people living with dementia, the referral process. I have a good sense of what I wanted to discuss with you. I was wondering, was there anything that you thought it’s important to tell me before I wrap it up?

Respondent:	No. I think just, always, it’s just lack of funding for services, isn’t there, standard over the whole of the UK?

Interviewer:	Yes. Do you feel that, within your PCN, you’re strained because it’s just the two of you, or do you work quite OK at the moment?

Respondent:	No, we’re OK, yes. We’ve got enough to keep us busy. I like being busy, so yes. And we also do – we do everything, weight management referrals, we started doing –

Interviewer:	You do lots of stuff, yes.

Respondent:	Yes. We started doing those in lockdown, or coming out of lockdown just because we weren’t that busy, so we offered, and it’s kind of just stayed. But it’s fine, it doesn’t take long to just do a quick referral.

Interviewer:	Yes. Oh, one thing I wanted to quickly ask you is, you touched on lockdown as well. Is the support that you provide mostly through the phone? Do you do face to face as well?

Respondent:	I would probably say majority via the phone, but we do, like, I’m out, I’ve got two home visits tomorrow, and I’ve got three I think on Wednesday. But then, I might not have any for a couple of weeks. So it’s just an option, I think, because some people don’t, I think especially if they’re elderly, they struggle to hear, or they might have speech problems on the phone, so we will just say, “Look, if you can’t get in here, we’ll come out and have a chat.” So, we’re quite lucky, we have that option, yes.

Interviewer:	Yes, there’s the option, that’s very helpful, particularly with some groups. Not all services are – have that option. Or, they do have it, but they’re discouraged from giving the –

Respondent:	I think with some people as well, you get a bigger picture of their life, when you go out to see them. So, you might go out and they might be hoarders, it might be a freezing house, and they can’t afford to put the heating on, or something. Something they might feel embarrassed to tell the doctor, so we can go out for one reason, but we’ll come out, we’re thinking, “Oh, right, well, this is why you’re low mood or you’re isolated,” or you know ...

Interviewer:	Yes, it gives you so much more in terms of assessment.

Respondent:	It does, yes.

Interviewer:	Yes.

Respondent:	Yes.

Interviewer:	Yes, OK. Well, that’s very helpful, [anonymised]. If you’re happy with what we’ve discussed, I’m going to wrap it up, if that’s OK with you.

Respondent:	OK.

Interviewer:	Yes?

Respondent:	Yes.

Interviewer:	So, let me just stop the recording here, so I don’t lose everything.

[End of recorded material 00:25:44]

**SP17**

[Start of recorded material at 00:00:00]

Interviewer:	An opening question, a bit of background info on yourself, what’s your role, what you do, and if you have some experience in supporting people living with dementia, if you could just tell me a bit about that.

Respondent:	OK, so my role is a social prescribing link worker. Previous to this role, I never had any experience of dementia or dealing with patients who have dementia, or living with dementia. So it was an eye-opening experience when I got the training and started supporting the clients that we get from the GPs. Yes, that is a very – now, I have the experience. We got training, we have resources in our team that is really, really helpful.

Interviewer:	It’s nice to hear that you feel more confident now given the support and resources that they were able to provide you with. Let me just ask you, in terms of the basic training that they gave you when you started in this role, what was it about around dementia? Was there anything specific around dementia that you got trained on?

Respondent:	So, when I started this role, the training was around being a social prescribing link worker. So, a social prescribing link worker is, we approach a holistic approach to our patients, and we deal with anything that is non-medical or non-clinical, information and advice. So, we cannot discuss the dementia or the medication, or things around that, but we are essentially a signposting service. So, we know a lot of organisations in our area that the patients are unaware of.

So, the training at the beginning was around being a social prescribing link worker, how does that role help our patients in the GP’s, so that was the first one. Then we had one training back in December 2022 around just dementia, and that was really helpful, actually, because it taught us how to speak to a patient who has dementia, or the carers. I didn’t know about memory clinics, I didn’t know about ... We just signpost them. We used to be like, “OK, this is an organisation that you can contact or we can refer you to,” but we didn’t understand what happens in those places, so that was really helpful.

Interviewer:	Yes, so they basically gave you a bit of a crash course on what services are out there that you can signpost people to.

Respondent:	Yes.

Interviewer:	Which, I think, is quite crucial because, as you say, that’s kind of your role, is to refer people to people that can then support their needs, so that sounds really helpful. In terms of the referral process, how does that work? Who do you get people referred from? Is it only through the GP? Do you have any other pathways?

Respondent:	No, just the GP, or now we’ve got the self-referral link, where patients themselves can – it’s either the patient’s referred themselves to us, or the carers, such as the son, the daughter, the husband, yes. But normally, 90 percent of our referrals come from the GP itself.

Interviewer:	OK, but it’s interesting that you started with self-referral because I’ve spoken to other people from other services, and self-referral is something still quite rare compared to the primary care pathway, which is the traditional one. Do you see, because that’s quite interesting, do you see any differences in terms of the people that are accessing your service, based on the GP, and based on self-referral? Are there any differences that strike you as being interesting?

Respondent:	So, the ones from the GP, because obviously we have access to the GP records, you can see what’s being said during the consultation, and what prompted the doctor to refer them to us. Self-referral, we don’t know what’s – why the patient have – it just says, “Dementia support or carer support,” so we have to obviously book in a consultation with them and really understand the reasoning behind their referral.

Interviewer:	That’s really interesting, yes.

Respondent:	That’s the main difference.

Interviewer:	So, basically, you’re saying that potentially when it’s through the GP, you have a bit more background info.

Respondent:	Exactly.

Interviewer:	Whereas, in the other case, it’s just, boom, just there, and you had to really understand the situation yourself, as you haven’t got –

Respondent:	Yes.

Interviewer:	That’s really interesting. Do you find it more interesting, more challenging? How do you find that?

Respondent:	Honestly, both times, even when I read the notes on the GP, I like to let the client lead the conversation because what they said during that consultation with the doctor might change. They might need something else. So, I always have open-ended questions for my – I introduce myself, my role, how we work, and then I just ask them to tell me a little bit about their needs and concerns, and then we take it from there. And I always ask them what is the most – because, sometimes they come in with so many issues, so our role is to fine-tune what they need. So, what is the most important thing to you right now, that you need to work on? That really helps them, so they’ll tell me, yes.

Interviewer:	OK. So is your model to actually prioritise the need and focus on one only throughout that referral?

Respondent:	Let’s say the most important thing for them, I’m going to give you an example, so I’ll get someone that says, “I have just been diagnosed with dementia. I’m really, really scared about the future. I don’t know what’s waiting for me. I don’t want anybody to know,” so on. So, in that scenario, you don’t know exactly how to help, what is it that they need. So obviously, you empathise with them on all of that, and then just say, “What is it that is the most important thing for you right now to know about dementia?” And, take it from there. So, they’ll say something about, “I just want to know if there’s any support out there for people living with dementia.”

So obviously, I’ll think of an organisation in my area that can give them that information. So, you go to the website, you see, you either have to refer them or just give the patient the phone number to call. Keep it simple, and ask them, “How would you like me to communicate with you? Through email, through text?” Sometimes, they just like to write it down because they want to see a visual to remember. So, you always give them the – I can’t find the word. You know, they have control over how they want to be communicated with, and you know, like that.

Interviewer:	It’s an empowering process, isn’t it?

Respondent:	It’s empowering them.

Interviewer:	Yes, yes, yes, I think that’s quite important, because what I know from experience about people living with dementia, is that particularly, as you say, when the get the diagnosis, they really feel lost, and they really feel like they’re losing control over their life, because essentially, that’s the progression that’s going to happen. So, I think it’s crucial, as you say, to give them that power of making then the move to either contact you, or actually engage in the process, because my question to you is also, do you ever find that sometimes people – you signpost resources in the community, but do you ever find that people do not really engage, or resist a bit that process?

Respondent:	Yes. So, you gauge. You gauge how they react, so that’s why I like to give them options even down to how to communicate with them. And, not just giving them one option, giving them several options so there are – I always fine tune it to three, so three links, just check them, have a look, see which ones you would like to engage with, and then we’ll take it from there. So, we offer up to six follow-ups, so they know that I’ll give you – would you like me to call you in two weeks’ time? Would you like me to call you in three weeks’ time? And that way, they have, again, they have that control.

So, I send them links, I don’t refer them straight away. I don’t take that option away from them. Give them time to digest all the information, then the next consultation will be like, “Did you have a look at the organisations that I’ve sent you?” Sometimes they’ll say, “I did, but I’m still not ready to make that decision yet,” then you offer another consultation. Let’s say you offered all the consultations, and they’re just not willing, I mean, not ready to engage, you just let them know, “Don’t worry about it. Here’s the self-referral link if you ever need us again, in the future.” But, that gave them the idea that there is help out there. There is us, and there’s the organisations, so they’re just armed with all this information.

Interviewer:	Yes, it’s knowing that, should they need it, there is somebody there, and that makes a difference, doesn’t it, particularly when you’re overwhelmed, and you might think there’s nobody you can seek help –

Respondent:	Yes.

Interviewer:	Yes, I understand that. I think it’s quite important. Do you ever have instances where the person asked you, “Can you help me go to that first appointment,” with the service you’re referring them to? Say, for example, I don’t know, there’s a dementia café, and they want to go but they feel a bit – they’re lacking confidence for that first time. Is part of your service to actually accompany them, or it’s just the signposting?

Respondent:	It’s just that. I think the remit of our role is very limited, where we just refer. Let’s say the dementia service in my area, you have to fill in a form. Again, you give them the option, would you like to fill in the form yourself, because there’s always a self-referral or a professional referrals. If they say, “I’m too overwhelmed, can you do it for me?” We do it for them, send it off, and that’s it. The remit of our role is to just chase up the referral. Let’s say they didn’t hear back in one week’s time, then I will contact the organisation, “Hey, what’s happening with this referral?” When will you contact this patient?” And so on, but that’s it.

Interviewer:	I see. I understand. Thanks for clarifying. Essentially, as you say, it’s a role of pure signposting. I guess, in some respects, it’s also to, as you said before, to leave the responsibility and the power to the person to make those steps, because if you do the steps, if you take the steps for them, in a way, it’s not productive for the future, when you’re not going to be there to support.

Respondent:	Exactly. We’re a short term service, and we make sure to let them know at the beginning, we’re a short term signposting service. This is what you will get from us. But, then you’ve got other people in the GP that can help, so for example, the care coordinator. I might ask the care coordinator, “Can you help with this?” So, we have direct communications with the doctors, the people that are in the GP. So, we’ll see which ones fit.

Interviewer:	That’s an interesting point. What would you say is the main difference between a social prescribing link worker such as yourself, and say for example, a care coordinator? Because you all kind of fall under the umbrella of –

Respondent:	I think we fall – yes, we haven’t really explored that. I always wondered what’s the difference between a care – I think maybe they more deal with the medical side of things. I think medication, they have a really face to face approach, more approach with the patient than us.

Interviewer:	OK. And do you mostly work remotely with your patients? OK, so it’s over the phone mostly?

Respondent:	Yes, but I always sometimes get requests for face to face. Again, it’s up to them. I offer them, but 90 percent of my patients prefer a telephone consultation or a video consultation.

Interviewer:	Oh, that’s interesting. And that applies to dementia as well, you find?

Respondent:	Same thing, yes.

Interviewer:	OK. It’s reassuring, because one would expect that lots of people living with dementia might be resistant to having a remote chat with somebody, but it’s good to hear. And do you find that in most of your referrals, it’s you talking to the caregivers mostly, or are you able to talk with the patient themselves?

Respondent:	My referrals have always been the caregivers. Yes, like one or two will be the actual person living with dementia, but the majority of the time, it’s the caregivers that are feeling very overwhelmed, and they don’t know where to go to get support.

Interviewer:	Yes, that’s what I’m finding. I’m finding that, more and more, with this particular population, it’s actually the caregivers asking for support.

Respondent:	Yes.

Interviewer:	OK. That’s another –

Respondent:	Because they feel very – and there’s an overwhelming feeling of guilt from them, that they’re struggling, that they should be able to know how to care, and they shouldn’t really look into, let’s say, getting carers for their loved ones that are living with dementia. And obviously, our role is to make them feel like it’s OK to ask for help. That’s what I get all the time.

Interviewer:	OK. And in part of this process of showing to them that they do have support, what are some of the good strategies that a social prescribing link worker can use when communicating with people? I’m just thinking in terms of what’s helpful for us to know, that we should include in this resource, maybe some tips and hints based on your experience, something that you find is helpful when you’re talking to someone who’s struggling?

Respondent:	Obviously, so my initial training really helped, how to talk to people, how to talk to someone who’s overwhelmed. Again, it’s all about, they’ve got all these issues. It could be a housing issue. It could be the house is not equipped for someone with dementia. There’s so many things going in their heads, our role is just to, like I said, fine tune. So, you’ve got this issue. Relay it back to them. Say things like, “OK, let me just understand the whole picture here. You’ve got this issue, you’ve got this issue, you’re got ... Correct?” And they’ll be like, “Yes.” And then out of all of these, let’s say four issues, which one would you like to focus on first?

Again, it’s just that, and I had a patient recently who was just crying over the phone, crying over the phone, but then it’s like, at the end of this session, this person was like, “Ah, it feels like a weight has been lifted off my shoulders.” And they know that I’m going to be calling them following it up, and so on.

Interviewer:	Because they are so overwhelmed, I guess one crucial point that you just made is that you actually almost tidy up their confusion, and start to make priorities, and almost a structure, right, to what they’re going to be doing?

Respondent:	Yes.

Interviewer:	In a way, it’s that tidying up of their mind, which I think really helps, and also maybe you touched on, really, a shoulder to cry on sometimes, just to really let out.

Respondent:	Yes. And they know that, obviously, they know that all the conversations that they have with us is confidential, unless I believe someone is at risk of harm, and safety, but just knowing that makes them open up a bit more, makes them just let it all out. Sometimes you will have patients or carers who come in and they’re like, “I just don’t want to speak about it anymore. I’ve just talked to the doctor. Why do I have to keep repeating?” They hate that. So obviously, you have to ask them, “Are you OK with me reading the notes to you from what the doctor said?” And sometimes they just ask for what they need. They don’t want to keep relaying information time and time again. So, sometimes I’ll write on the referrals, “Please make sure, patient does not want to relay the information. Bear that in mind.” That’s it.

Interviewer:	Yes, and I guess following from what you just said, do you ever have instances in which a person is so overwhelmed, a carer, that they really want support that’s not necessarily dementia related, because they don’t want to hear about it? So, other stuff in the community going on, which is purely social but not necessarily dementia café, dementia peer support, something that’s not dementia?

Respondent:	Yes, sometimes they need counselling. That’s an option, too. So again, you just have to have this resource. We have these resources, where this organisation helps just carers, or this organisation helps carers and people living with dementia. And then you have to really be knowledgeable in what they offer. You can’t just signpost people, where they need counselling, you signpost them to the wrong organisation. It’s just a waste of time for us, for them. So, you really have to be knowledgeable, because some organisations actually offer counselling for the carers, and that’s an option, too.

Interviewer:	It really requires that holistic knowledge on your part, because essentially, people can come to you with anything, literally from A to Z, whatever is going through their minds, and you have to be – because you work in a time limited manner as well, so it’s kind of challenging, I think, in a way.

Respondent:	Yes. It is.

Interviewer:	Yes. I’m wondering, in terms of the referrals that you get, do you think there’s diversity in the population you get referred, in terms of different communities? Because, I’ve spoken to people from different areas, very diverse areas, versus more rural areas, which are less diverse, and the general feeling that I have at this stage is that usually, with white British population, it’s interesting because people feel that the person living with dementia has less support from families, and potentially their children, and therefore, interestingly, they sometimes come with more intensive needs, as opposed to different cultures where there’s a bit more support from the family. So first, I wanted to ask you, do you get diversity in people who are referred to you, and if so, what are some of your ideas around that?

Respondent:	I think that’s very interesting, because I feel the same way. I don’t get – obviously you have certain ethnicities that are very willing, very open, “I’m struggling with this. I need help with this and that.” They’re more willing to be like, “OK, I might need carers for my mum or my dad.” They’re open about that. But certain ethnicities, the don’t want – they don’t even want to mention the word, “Dementia.” Sometimes I’ll get referred a patient from a certain ethnicity, and they’re like, “No, I didn’t – I don’t want any support, thank you, I’m fine,” and that’s the end of the consultation. But I think as we go more and more, people are reading online more, there’s access to the internet. People are more willing to reach out for help, if that makes any sense.

Interviewer:	Yes, it does.

Respondent:	But, there’s the sense of guilt from certain ethnicities, like, “Oh, I shouldn’t be feeling like this, I should be able to handle this,” and you know?

Interviewer:	Yes. That’s part of my cultural background as well. I’m Italian, and in Italy, there is such emphasis on family responsibility for the person living with dementia. My grandmother had dementia, and my parents would have never, ever sought for help, to the detriment of their own health, they would never do that, so I understand this point of view. And, when you actually come across this kind of rejection, what is your role as a social prescriber? How do you tackle that?

Respondent:	OK, so obviously, we offer the initial consultation, where it’s kind of like an assessment., And, we explain what we do, and how we work, and then we leave it to them. We have an option where it says, “Patient declined the referral,” which is fine. What we do it we let them know that, “It’s OK. you can always come back to us if you change your mind in the future,” and leave it to them, and that’s it.

Interviewer:	OK, so again –

Respondent:	You can’t be pushy, yes.

Interviewer:	Yes. Again, it’s part of leaving the responsibility to them, that the choice is theirs, essentially. OK.

Respondent:	And that happens. Sometimes they decline the referral, but then you see their name a month later when they’re ready.

Interviewer:	Oh, interesting.

Respondent:	They’ll come back, yes.

Interviewer:	It’s about readiness.

Respondent:	It’s how you speak to them as well, if you make them feel like it’s OK, obviously this is a normal feeling, a lot of people are not ready, but when you’re ready, or when you want our services again, this is how you reach us. That’s it. Simple.

Interviewer:	So, you would say that this is good practice when you get that resistance, essentially?

Respondent:	Yes, and I ask them, “Would you like some information about support for dementia in your area?” If they say, “No,” you don’t send them anything. If they say, “Yes,” you send them the links, and that’s it.

Interviewer:	Yes. Yes, yes, yes, yes. The way you send information, is it mostly through email, or do you send out also printed stuff through the mail?

Respondent:	It depends on the patient. So, obviously the older people like leaflets. Everybody now, you’ve got a phone, text messaging, emails are very popular. But on the rare occasions, we do have people asking for leaflets.

Interviewer:	OK. Oh, you obviously have that option if the person prefers.

Respondent:	Uh hm.

Interviewer:	Yes, yes, yes, yes. And, you touched before on the actual knowledge that you guys have about resources in the community, right? So I was wondering, because this is also kind of challenging, isn’t it? Because there’s constant changes and updates in what’s out there. How do you guys keep on top of it?

Respondent:	Our managers always send us, “Hi, did you guys realise there’s this new organisation that you can refer your patients to?” Because sometimes an organisation is so saturated, and the waiting list is up to three months for them to get in touch. So it’s nice to be up to date. Our managers are on point. “There’s these new people.” Sometimes we have meetings with them, and find out how they support our clients. So it’s nice to be – yes.

Interviewer:	So, in a way, you’re supported by your manager. I was wondering, practically speaking, say you’ve got somebody who’s interested in a specific activity, what do you do? Do you usually ask your manager what’s the options out there, or do you have a directory where you can look for the options?

Respondent:	So, we have so many options in our team, so we’ve got a WhatsApp group where we can, “Oh, I have a patient who’s this, this, this, did you guys come across anything?” A quick search on the internet. I’m so sorry, there’s house renovations.

Interviewer:	Yes, don’t worry, I can’t hear it, actually, it’s fine.

Respondent:	OK, that’s good. Yes, so we have our group, just social prescribers. We have a quick search on the internet, sometimes that helps. We have, as well, a directory, like these are the main places to go to. But, let’s say, I get this sometimes, an Arabic speaking patient, who only speaks Arabic, would like that support. So, that’s very specific, so we go on the internet, we look if there’s anything national. If I can’t find anything, I’ll ask my managers, or I’ll ask the team, and see which one responds first.

Interviewer:	Yes. Yes, yes, yes, that’s –

Respondent:	We’re kind of like a research – we’re researchers, basically.

Interviewer:	Yes. Yes, yes, in a way, you are. You’re a research team because you’re always on the look for what’s new and what’s available, and you touched on an interesting point that, sometimes, I suppose you get very kind of niche, yes, requests? So, that’s quite crucial. Let me think, do I have any other questions that might be – yes, maybe almost a final question is, because we’re developing this resource, and obviously we want this to be helpful to people like you, is there anything that comes to your mind that is, you think, really important to include in this resource, in terms of maybe pointers, in terms of hints and tips, in terms of information, anything that can help people out there, maybe with less experience than you have?

Respondent:	I think just, we just need – well, we did have that training back in December, and I have the slide of what dementia is, what types of dementia, how to speak to someone who has dementia. Before my training, I didn't know that some people don't want to hear the word dementia. I would take on a dementia referral, call the client and I would just say - hi, I'm calling regarding the dementia of Mr X”. So, maybe just kind of like a booklet for us, for new social prescribers that are just starting, to have knowledge around dementia support, for carers, for dementia patients themselves. Yes.

Interviewer:	OK, almost kind of a checklist.

Respondent:	Yes.

Interviewer:	That helps you through the process, and maybe the initial contact with the person, because I think that’s quite an important stage, because it’s where you build that trust and build that rapport. So, if you don’t know how to communicate with the person, maybe almost having a checklist of saying, “Have you tried this? Have you made sure that you haven’t used the word, “Dementia,” in the first place, and actually gauged at first what is the right terminology with that person.”

Respondent:	Yes.

Interviewer:	Almost that sort of thing, that people who haven’t got the experience can use. And then, of course, with time, they will get the experience, and they might not need that checklist anymore.

Respondent:	Yes.

Interviewer:	But yes, I can see –

Respondent:	Because, I didn’t know at the beginning, I would read on the GP notes, “Dementia care needed,” and I’m like, what does, “Dementia care needed,” mean? So it’s just that. I think that’s what’s missing. What does it mean, you know? Yes.

Interviewer:	I see. OK, that’s very important. And, I suppose some of the stuff that we’ve discussed today in terms of the strategies that you use can also be part of that checklist, if it’s going to be a checklist.

Respondent:	Uh hm.

Interviewer:	OK, that’s very, very helpful. What I’ll do – do you have any final points that you wanted to tell me, or are you happy with what we’ve discussed?

Respondent:	No, I’m happy with what we’ve discussed, really, yes.

Interviewer:	Awesome. Awesome, so what I’ll do is, I’ll stop the transcription.

[End of recorded material 00:29:47]

**SP18**

[Start of recorded material at 00:00:00]

Interviewer:	Just like an intro question, can I ask you a bit about your background, maybe what you do, in what capacity, and in relation to people living with dementia?

Respondent:	So, I have been, for the last almost six months, a social prescribing link worker based in a PCN. So, I have two colleagues, and we cover five surgeries locally, so it’s a wide range of adults, but including people with dementia. Previous to that, I have worked in another social prescribing role based in a voluntary organisation. And then, I’ve also worked, in between those two roles, I worked for a voluntary organisation who recruited, trained and placed volunteer befrienders for older people. So I bumped up against difficulties with getting people with dementia to access that service, and that kind of thing.

Interviewer:	OK. Can I just ask you, you mentioned the previous job that you did within this charity? Was that for people living with dementia?

Respondent:	No, it was, again, just adults.

Interviewer:	Adults, OK, yes, yes, yes. It actually makes sense, because from what I’m hearing from people, there’s actually no specific social prescribing service or specialism for people living with dementia, so it makes sense that it’s all adults. So, OK, this is a bit of your background. Yes, another curiosity I had about a PCN versus charity, because you were supporting from the side of social prescribing in both jobs, any major differences that come to your mind?

Respondent:	The most important difference is that, based in a PCN, I can see people’s medical records. So, before I meet them, I examine people’s medical records to get a much clearer idea of what their diagnosis is, and I know whether they've got consent for relatives to speak for them, whereas working in a voluntary organisation, you don’t have access to any of that, and you’re very reliant on what people tell you, which is, people tell you their truth of the situation, which is not always the whole truth, and it’s –

Interviewer:	And I suppose, yes, particularly with people living with dementia, there is also an issue with reliability about information, I suppose.

Respondent:	Yes. Yes.

Interviewer:	So, that’s that massive difference, actually. It’s interesting because I just had a conversation yesterday with another social prescriber, who mentioned the very same thing, however this was in relation to referral from the GP versus self-referral, which is something that they do also within their PCN.

Respondent:	Yes.

Interviewer:	So, do you guys do such referral?

Respondent:	Not exactly. I mean, if we’ve had contact with someone, or maybe we’ve been out to an event, because we go out to a lot of community groups and stuff, people can just come and talk to us, or they might get our number from someone else. But mainly, our referrals come from the GP or other health professionals, sometimes from social workers, sometimes from other voluntary organisations, but mainly from the GPs and other practice staff.

Interviewer:	OK, I see. In terms of the background information that you get from the medical records, do you find this is helpful? And, I’m asking because, again, somebody told me in other interviews that, sometimes what the real need of the person is, is either not the superficial or the recorded one in the clinical notes –

Respondent:	Yes.

Interviewer:	Yes?

Respondent:	Yes.

Interviewer:	Does that happen?

Respondent:	Yes.

Interviewer:	And how do you find that out, actually?

Respondent:	I think you have to ask the right questions, be nosy, and have enough time to listen, and just pick up on things. I like to go and visit people at home. You learn a lot more that way. Sometimes that’s not possible, but yes, it’s definitely about listening, and even if I’ve looked at their medical records, and I may have – I’m going to see a lady after this, and I’m going to have a little look, and I would’ve looked before I rang her to make the appointment, and we might have had a little chat on the phone, but I will look and I might write down some things that I think are relevant, or some things I might think I’ll be signposting her towards, but when I get there, I ask open questions and let her lead where the conversation’s going. If the GP sent her to me with a specific request, I would try and steer the conversation that way if she doesn’t mention it, but I would try and see if she’ll mention it first, and what she does mention, we can talk about.

Interviewer:	Ultimately, is it about what the person suggests that they want out of your support, or is it about what the GP pointed you toward, if there’s a discrepancy?

Respondent:	I would say you can’t get anyone to do anything they don’t want to, so it’s led by the person. But, if the GP has raised a specific issue or concern, I would make sure we talked about it. If they’re adamant they don’t want that, then that’s fine. That’s up to them.

Interviewer:	Yes, that makes sense, and in fact, it leads me to this follow up question, which is something that I’ve come across with people is, how do you – particularly people living with dementia, how do you work around the potential resistance that you get from people?

Respondent:	It’s tricky, and actually, I would say mainly I don’t deal with people with dementia on their own. And either that’s because they’re resistant to the idea of help, so they don’t let me come and visit them, and engage even when I call and offer to help. Or, I get there and they just say no to everything, or they have an excuse as to why not now, or what happens more often is, the family will be there, they will be the ones raising the concern to the GP, asking for more support. And sometimes, in fact, I would say probably half the time, I’m not meeting the person with dementia at all. I’m just talking to their relatives. And sometimes, the relatives prefer that because they can be more honest, whereas the person with dementia quite often would deny there’s a problem, or deny that they need any help.

Interviewer:	That’s really interesting, because I suppose social prescribing is underpinned by the ethos that it’s about the person’s needs, and I’ve often wondered and asked people like yourself, where do you think, in that case, you’re supporting the caregiver as opposed to the person? Or, is it like a package? I don’t know, what’s your take?

Respondent:	I think quite often, it is supporting the caregiver, and sometimes you have to be quite – sometimes they don’t want to accept any help either, or they say the person with dementia isn’t going to like it, so we won’t do it.

Interviewer:	Oh, interesting, OK. So there is a bit of gatekeeping sometimes.

Respondent:	Yes, definitely. I think the most difficult conversations, which I’ve had probably twice, I think, is where I have met the person with dementia and their relative, and the relative is adamant they want them to have or do something, and the person with dementia say, “I don’t want to. Please, don’t make me,” and that’s just horrible. It’s horrible.

Interviewer:	What do you do in that case?

Respondent:	I’ve had a very difficult conversation with a daughter over several phone conversations, where she was adamant her mum wanted something, and I was like, “Well, your mum said no to me very clearly several times. She needs to consent. Unless someone has got power of attorney and the person is deemed not to have any capacity, I have to listen to what the client is telling me.”

Interviewer:	Yes, so the bottom line at the end of the day, is that legal power of attorney, whether it’s in place or not.

Respondent:	Yes.

Interviewer:	Because that will base your decision as to whether you need to listen to one or the other, if there’s a conflict, obviously.

Respondent:	Yes.

Interviewer:	But it’s interesting, [anonymised], that you mention that sometimes there is discrepancy between what one wants and what the other wants.

Respondent:	Yes. Yes.

Interviewer:	It’s very interesting. And, I suppose it takes lots of skills and mediation on your part to sort out the situation.

Respondent:	Yes, and sometimes you see people multiple times over – they will go away and say, “No,” and then a few months later, they’ll come back and they’re a little more receptive to it, as nothing has changed, or it’s got worse.

Interviewer:	Yes. So it’s about readiness of people, isn’t it?

Respondent:	Yes. Yes.

Interviewer:	Sometimes the right time is not that one.

Respondent:	Yes.

Interviewer:	And what do you do? I’m just asking specific questions on what you do, because I’m thinking about that resource being filled up with hints and tips that people can use when you have these situations. So, I wonder, when you see that there is that resistance, and you say you can’t do much because it’s down to the person’s decision, how do you, however, kind of sow the seed to make sure that, if in six months’ time, they want to be back, they can?

Respondent:	I think I’m always very clear that everything is their choice. I can tell them about all kinds of stuff, but it’s their choice, and they can always try it later. I’m always available. They can contact me again. It’s tricky.

Interviewer:	It is tricky, and there’s such diversity of people.

Respondent:	And take the long view, and also, you kind of have to protect yourself as a worker, and not get too hung up on them having to achieve something.

Interviewer:	Yes. Yes, that’s a great point. It’s about emotional well-being of the worker as well.

Respondent:	Yes, yes.

Interviewer:	Yes. And, in fact, a point that been raised by lots of people I’ve interviewed is, what kind of infrastructure is in place to make sure that the worker’s well-being is safeguarded? Because especially with dementia, it’s very challenging, and you hear lots of difficult stories, so what is your – within your serviced, what’s the structure in place to make sure you’re well?

Respondent:	We’re a very supportive team, I would say. We can just ring each other up and have a little rant or a cry.

Interviewer:	That’s important.

Respondent:	Yes. But then we do also have, once a month, I have - it’s kind of like clinical supervision, but it’s not really, because the PCN employs a psychologist. We individually meet with him once a month to talk about anything, really.

Interviewer:	OK. So, would you say that this is psychological support, in a way?

Respondent:	Yes, yes. Yes.

Interviewer:	That’s great. I haven’t heard about this in other services so far.

Respondent:	No, I think we’re lucky.

Interviewer:	Yes, sounds quite good actually. It sounds really great. I was thinking in terms of translating information into this package, that I wanted to put something about making sure that you’re well in your work, so that’s why I’m asking about whether there’s some support in terms of your team. People have also reported they have sometimes WhatsApp groups within the social prescribing workforce.

Respondent:	Yes. Yes.

Interviewer:	So, it’s important to have something in place, I think.

Respondent:	Yes, definitely. Definitely. A lot of us are working at least partly from home, so it can be quite isolating.

Interviewer:	What’s your work pattern?

Respondent:	It’s left up to me. It’s very flexible.

Interviewer:	That’s great, too.

Respondent:	Yes, so a lot of the time I’m at home because that’s easier. I’m not disturbing anyone with my phone calls and stuff. But, we do have an office we can come into.

Interviewer:	An issue with a camera on Teams.

Respondent:	Yes, yes, yes, yes, yes.

Interviewer:	I see. What about in terms of supporting your patients or clients? Do you mostly do it over the phone? How does that work with people living with dementia in particular?

Respondent:	I like to try and visit people because I think you learn a lot more. I would say people with dementia, because it tends to be relatives, you do tend to do more of it over the phone. Some relatives like you to go and meet the person with dementia, and I always offer to, but sometimes, they don’t want to do that because they want to speak more freely, or they’re at the stage in their dementia where they are worried by people coming in, or ...

Interviewer:	Yes, io suppose it’s also the consequences of COVID and all that vulnerability issues.

Respondent:	Yes. Yes.

Interviewer:	It’s a bit of, yes, a tail from that very difficult period. You mentioned that usually you – ideally, you like to visit people because it’s easier for you to assess. Can I ask you what do you see are the main differences, why do you think it’s sometimes best?

Respondent:	You can tell such a lot about people by their house, I’m going to say. Ooh, you’ve disappeared.

Interviewer:	Ooh.

Respondent:	It’s another task has come in, so it’s overruled you because it’s so important [laughs].

Interviewer:	Let me know if you have to go, because this is –

Respondent:	No, no, no, it’s fine. It’s just the way it works. It pops up and fills the screen, so I have to say, “Go away.” Just, it’s so much easier to tell if people are coping, I think, particularly people with dementia. You can see whether the house is clean and tidy. Even just the fact that you knock on the door, you’ve arranged to come and see them, but they have no recollection of the fact. But, they still invite you in, they’re still quite happy to invite you in, that tells you quite a lot straight away.

Interviewer:	OK, tells you a lot about social isolation as well, I guess.

Respondent:	Yes. Yes, yes, and the fact that they’re just – I think it’s very common, in my experience, for people with dementia, if they’re not sure if they know someone, they will pretend that they do, almost, so as not to offend, so that they’re a bit more vulnerable and they tend to just let you in.

Interviewer:	Yes, which I suppose, in some respects, justifies the gatekeeping attitude that some caregivers might have, because obviously it’s for their own protection.

Respondent:	Yes. Yes. But also, if they’re talking about stuff like, that they’re coping well, and I ask very specific questions about what are you doing about shopping, what are you eating, those kinds of things, I have sometimes even gone so far as to say, “Oh, can I look in your fridge and see what you’re got?” Or they might offer to make me a cup of tea, and you kind of see anyway. I’ve had a person with dementia offer to make me a cup of tea, and then they’ve got the milk out the fridge and it’s all lumpy.

Interviewer:	Yes. It’s really telling. I agree it makes such difference, because it gives you, by looking at the environment, every single aspect of it, you really understand what’s the truth behind whatever people tell you, right? So, over the phone, I suppose they can tell you whatever. They can tell you that they’re fine, right?

Respondent:	Yes, yes.

Interviewer:	And maybe people do it out of coping mechanisms and stuff like that, just to protect themselves even emotionally. What are some of the clues that social prescriber link workers look for to say, “This person really needs the support”? You made the example of the milk, which is really –

Respondent:	Yes, yes. Yes, yes.

Interviewer:	Do you have any other hints and tips that you look for, maybe?

Respondent:	I mean, I did work with a colleague previously, who would ask a question at the beginning of the conversation, and then ask again towards the end, and see if they had any recall that they’d already been asked it, or gave the same answer. I took that away with me.

Interviewer:	It’s great to just double check.

Respondent:	Yes. Yes, yes.

Interviewer:	I suppose it plays to the advantage of the worker, the fact that they might not remember the initial answer if it was a lie, so they won’t remember, and they’ll be –

Respondent:	Yes, yes.

Interviewer:	That’s a good one, I think.

Respondent:	Yes. And I have another colleague who also, if you were going to meet someone a couple of times, she would take not of what clothes they’re wearing, to see if they’re wearing exactly the same ones again.

Interviewer:	Yes, that’s another good one.

Respondent:	Yes. And I think just that initial, have they remember you’re coming, the fact that you’ve made – I’ve always made an appointment not that long in advance, and do they remember that I’m coming, and what I’m there for?

Interviewer:	OK. Do you find that, if they don’t remember, that there’s something going on in terms of red flag?

Respondent:	Often, but not always, but then you can kind of follow it up with some other things, and see if they actually recall what you’re talking about. I do also ask people things like just that general chit chat, “Ooh, this is a nice house. How long have you lived here?” And, “Oh, is this a picture of your family? Who are they?” Those kinds of things, it just, you know ...

Interviewer:	I suppose in some respects you have to be somehow sneaky, in a way, to get the information that you need, the reliable, the true one.

Respondent:	Yes, yes.

Interviewer:	That’s quite important. I saw some time ago on the Alzheimer’s Society website, there was a whole article about using white lies with people living with dementia, in other words, lies that help you have them cooperate with you, but still being ethical in what you do.

Respondent:	Yes. Yes, yes, yes.

Interviewer:	So, yes, I can understand these strategies. How do you, when you approach a client, and this might be the caregiver as well, how do you support them? If they have, say, a variety of needs unmet, do you prioritise any, or is your job to really fulfil all those needs within that referral? What’s your take with clients in this respect?

Respondent:	It really – I would try and do everything that they’ve brought up if possible. Now, sometimes, that’s an awful lot, so I might say, “Shall we do this first, and do that later?” quite often, what I like to do is send an email afterwards just saying, “This is what we discussed. These are the things I mentioned,” with a link to information and stuff, which can work quite well for caregivers, and then follow up later with a phone call. But I think it’s very much kind of at the end of the conversation saying, “OK, so this, I’m going to do a referral to this for you. Is that OK? And, I’ll send you information about this, and you’re going to do that,” and just kind of go back over it and decide between us what we’re going to do, and in what order, if there’s too much to do at once.

Interviewer:	Yes. I think the idea of a follow up email with a list of the actions, it’s very helpful for people to remember, but also to structure their mind in terms of action plans, I think.

Respondent:	Yes, yes.

Interviewer:	So, that’s quite helpful.

Respondent:	And for my protection, I also put in if they’ve declined anything.

Interviewer:	Yes, sure, yes, it goes back to being safeguarded, yourself, because that’s important.

Respondent:	Yes, yes.

Interviewer:	I was wondering, in terms of the specific role that you guys have within your service, is it only about signposting people, in other words, once you’ve done the signposting, it’s their responsibility to take over and engage in whatever you’ve signposted them to, or do you, if there is a need there, do you also kind of facilitate the process of initial engagement? How does that work?

Respondent:	If we think they’re capable, we’d go for the signposting because it’s quicker and easier, and you don’t want people to get dependent on you, but if they need that bit more support, then we’ll do that.

Interviewer:	Yes, OK. The issue of dependency has been brought up quite often, particularly with certain populations. You’d mentioned before the social isolation, and that’s the typical mechanism that create dependency, because all of a sudden, you have someone there, and you don’t want that someone to go.

Respondent:	Yes, yes. Yes.

Interviewer:	Do you ever struggle with termination of rapport?

Respondent:	Sometimes. I think in my first job, social prescribing, far more of a problem, partly because we were much more under pressure to close things down and move on, and partly just because I didn’t really know what I was doing at that point, whereas I think now, I’m much clearer that I can’t solve everything for everybody, and I shouldn’t try because that just leads to me going a bit loopy, and not enjoying the job anymore. But, there are still people, where you can see they are really vulnerable, and it’s quite hard to cut them loose, but you do have to do it, and you do have to try and move them on to whatever the next service is that they really need to engage with. And the hardest bit is where there isn’t really a service for them.

Interviewer:	Does that happen?

Respondent:	Yes, where there’s not something suitable, or there’s not – they’re not prepared to go to it, and you still have to kind of say, “Well, I’m done. Get in contact if you need help in the future, but I’m done.”

Interviewer:	Yes. It’s interesting what you mentioned about some services not being available for their needs. Do you have any examples? I’d be curious to hear.

Respondent:	So, I would say that there are dementia groups and day centres and services and stuff, but some people just, they – it depends on the cohort of people who are already there, whether people think that they fit into it. If they go along and the people there are worse than them, they’ll feel like they don’t want to be there. And I think that’s the same for other conditions as well. I’ve spoken to people with Parkinson’s, and they’ve said, “Well, I don’t want to go there because I can see what my future is, and I don’t like it.”

Interviewer:	Yes, particularly with progressive conditions, I suppose.

Respondent:	Yes.

Interviewer:	Because you know that, inevitably, that’s the future. You don’t want to be confronted with it.

Respondent:	Yes. Yes.

Interviewer:	I thought, when you said that, I thought you were referring, for example, because that’s something I’ve heard, about particular people from different cultural backgrounds or ethnicity, that they don’t really feel what’s available is fit for them or their needs. Is that something that comes up in your work?

Respondent:	Personally, I haven’t experiences it a lot. I think I’ve had the – probably not people with dementia, actually, but other people that I’m dealing with other things, I’ve had it occasionally, they say that, “There’s no one else like me there,” and I do – if I do come across it, I’m kind of – people are people, and try it, see how it goes.

Interviewer:	Yes. And people are an intersection of different characteristics, so one characteristic does not necessarily define you because there’s so many layers, right?

Respondent:	Yes, yes. Yes.

Interviewer:	So, I can see your point. There was another question I had that came to mind, and now it’s just slipped away.

Respondent:	And maybe that’s also because this is probably quite a white area, so maybe that’s why it hasn’t come up as much.

Interviewer:	OK. How diverse do you think the area is, from your experience, of people who access the service, actually?

Respondent:	Older people, I would say it’s less diverse.

Interviewer:	It is, yes, OK.

Respondent:	Yes. I mean, that’s just my own impression of it based on the people I’ve seen.

Interviewer:	Yes, of course.

Respondent:	I guess it may be that actually, there are people there, and they’re just not wanting to engage.

Interviewer:	Yes, that’s a good point, and that’s something that was brought up in other interviews. It might be that there is a bit of a resistance to go through the GP route because, in some communities, there might be also stigma associated with seeking help if you’ve got family that should be there to support you.

Respondent:	Yes.

Interviewer:	So, I agree with you, that there could be a hesitancy there to actually go through that route. I wonder maybe through the actual self-referral route, or the outreach work that you guys do in the community, you think you’re able to potentially target, or access or reach to those groups a bit more?

Respondent:	Yes, maybe. I mean, I think a lot of the self-referral route is very difficult because most people don’t really know what social prescribing is still, so they’re kind of like, “What is that? Why?” Even people who’ve been referred over to us by the GP, you ring and they go, “So, what can you do? What do you do? What are you?”

Interviewer:	So, you have to explain over and over, I suppose.

Respondent:	Yes.

Interviewer:	Yes, because they mix it up with social care, that’s what I’ve been told.

Respondent:	Yes, yes.

Interviewer:	They mix it up with prescribing medicines –

Respondent:	Yes, yes, yes, yes, yes, yes.

Interviewer:	It’s not the most fortunate terminology, I think.

Respondent:	No, and that’s the one downside of being employed by the NHS, and having an NHS lanyard, is that I have to pretend to have much less understanding of medical things than I do, because otherwise you get into very medical conversations. So, I do generally go, “Oh, that’s going straight over my head. I’m sorry. That’s not my area.”

Interviewer:	That’s the GP’s area.

Respondent:	Yes.

Interviewer:	That’s really interesting, this point of view. Do you feel that GPs are ready to understand what social prescribing really is, to work alongside? Because, what I’ve heard from some people is that change of culture is still happening, the medicalised model of illness, if we want to call it that way, applying to dementia as well, so I wanted to ask what’s your take on this?

Respondent:	I think it just depends on the person. Some GPs get it, and some either don’t, or don’t see the need for it.

Interviewer:	Yes.

Respondent:	Or, misunderstand what it is.

Interviewer:	I had a conversation yesterday, and this person was saying to me that they have self-referral, and what they find is that it’s helpful because they see that if you go through the GP route, dementia, for example, the first thing that you get is medication. You also might get referred to social prescribing, but you will get medicated regardless. And that might not necessarily be needed, if it’s really social, whereas through the self-referral route, you can sort of bypass that in a way, so they were finding it interesting that it was a way for people to maybe get away from that medical model that we traditionally were used to. But, that’s just a point I found interesting.

Respondent:	Yes.

Interviewer:	In terms of what to include in this resource, was there anything else that comes to your mind that you’d think, “This might be really good,” for people who are maybe less experienced than you are?

Respondent:	I don’t know. That’s a tricky one.

Interviewer:	I know, that’s a very open question.

Respondent:	Yes, it is. It’s difficult, because an awful lot of the things you can actually send people to are very local, or even if they are things that are available everywhere, they will be run differently in different areas. And that kind of stuff is really invaluable, having that at your fingertips, feeling that there is somewhere you can send people, there is something you can give them, because otherwise you’re just listening and going, “Yes, that’s terrible, isn’t it?”

Interviewer:	Yes.

Respondent:	You need to have something to give them, so it almost needs to be kind of the overall resource, but then with links to stuff that’s local, even down to just how social services work in your county, because it will be different.

Interviewer:	Because it’s so very different, there won’t be, for us, an option to put links to local stuff, because it’s just impossible.

Respondent:	No. No.

Interviewer:	But, what we can do, based on what you are saying is, if we have, again, that kind of checklist with hints and tips, if one could be, “Make sure that you know your local resources, make sure that you have a group of colleagues you can share resources with, or ask for information,” maybe there is a repository, this is what’s happening in some services, where you can actually look for local stuff. So it’s the kind of overarching principle, if that makes sense, of saying, “You need to be aware of what’s going on, because that’s the foundation of what you’re going to be doing.”

Respondent:	Yes. Yes, yes, yes.

Interviewer:	Yes.

Respondent:	And I would say always check that services you’ve found by Googling still exist.

Interviewer:	Yes, that’s a good one. How do you actually keep up to date with all the changes?

Respondent:	It’s tricky. I think it’s partly knowing that you’ve – it’s good to go and look at places in person, because A, you know they exist, B, you know that they’re actually running how they said they were running on their website, and they’re not doing something entirely different, which I have found is true, because people will have their referral criteria on their paperwork, but then you go along, and actually, they’ll be taking a completely different group of people because they’re not getting enough referrals. So, that’s useful information to know. But also, it means you can sell the service to people you’re talking to, because you’ve actually experienced it, and can talk about Sue, who runs it, and oh, yes, you can definitely get in, there’s no steps, those kinds of things.

Interviewer:	It’s the firsthand experience, that you’ve been there and you can vouch for it.

Respondent:	Yes.

Interviewer:	And, I suppose it’s also about establishing the links with the actual organisations, so that they know you, and in a way, it’s a bit more facilitative in that respect.

Respondent:	Yes, yes, yes. Yes, yes, yes.

Interviewer:	Yes, OK, that’s another very good principle. I think I’ve finished with my questions, and I’m also wary of time, I don’t want to take you away from your important – the thing that popped up before, so if that’s OK with you, I’m going to stop the recording.

Respondent:	Yes, yes.

Interviewer:	Yes? Let me just stop this. OK. What I’ll –

[End of recorded material 00:34:20]

**SP19**

[Start of recorded material at 00:00:00]

Interviewer:	As a first question, can I ask you a bit of a background about yourself, so what you do, in what capacity, and if you’ve got some experience with supporting people living with dementia, that would be helpful to know?

Respondent:	OK. So, I think the way that I describe myself half-jokingly to people is a jack of all trades and master of none. I’ve had a very – I’m 65. I’ve had a very long and varied career. I’ve never worked in one place more than seven years, and for the majority, or the largest part of my working life, I worked in the advertising and design world, based in London. So, I’ve got quite a lot of communication strengths through that, and I was in sales and marketing for a lot of that time.

I’m not going to give you my whole story, but I then moved – I had a long period where I wasn’t in work, through my own choice, actually. I went on a long journey with my wife across bits of [anonymised]. We were lucky enough to have the money to do that. And then, when I got back, I got into working with young homeless people, and then went strangely into policing and became a beat police officer working in local communities.

And at that point, I started to realise that policing was dealing with a lot of social issues, and you could see that government were downloading responsibility on the quiet, to policing to deal with things that they were really not equipped to deal with. I don’t know if you’re aware of care in the community, but basically, they shut down a lot of the old, what I would call asylums, and pushed patients out into the community, didn’t resource that, and therefore, we were – the police officers were coming across people who really shouldn’t – who were basically left vulnerable and were becoming problematic within communities, and not doing anything about it. Anyway, that’s what I was discovering on the ground.

When I left policing after just under seven years, which was an experience that I really enjoyed, actually, I found it really, really satisfying, I then became a community navigator, which is a subsection of social prescribing, working for a charity, [anonymised], and dealing with people, elderly people coming out of hospital, who had been in for some time, and resettling them back into the community.

From there I then jumped, when social prescribing became an NHS project just into 2020, I became a primary care network social prescriber. I knew the particular PCN because I’d been working with them. They asked me to join them, and I joined with them. Now, by then, I had some experience working with, A, families and people with dementia, both recently diagnosed and chronic, and I was working with charities, third sector, that were dealing with. And so, walking into being a social prescriber brought me nothing new, just more of it, so that kind of gives you a background.

Interviewer:	Yes. It’s really helpful to learn about your trajectory into the sector. I was wondering, you definitely have lots of experience in dealing, or supporting people living with dementia, and because this is going to be a very practical kind of output that we’re trying to develop, I wanted to ask you specifically what are some of the unique challenges that you’ve found, yourself, when dealing with people with dementia? Because, you’ve supported so many different populations, I suppose in your mind, you have some pointers as to what could be some specific challenges in supporting people living with dementia, and their caregivers as well.

Respondent:	Right, OK, so the key here, for me, and I think for my colleagues, is by and large, we do not deal face to face very much with people with dementia. The issue for us is their families, and those that care for them, because once somebody has got dementia, there might be, in the initial phases, they have a bit of understanding because they have moments of clarity, but generally, by the time we’ve got to them, it’s their families that are in crisis.

They’re the ones experiencing the pain and the distress because they don’t know what to do. They are very unguided. They don’t know what the resources are. They are bewildered and emotionally distressed by the fact that the person in front of them was not the person they were a year ago, two years ago, three years ago. They are now something else, and that is an incredibly toxic and destructive place to be in. some people deal with it well, resign themselves to the fact that this is what’s going to happen, and kind of get on with it, and we then provide a kind of supportive mechanism, if you like, as and when they might need it.

And we will say to them, “Look, you’re doing everything that you can. We’ve signposted you or referred you in to other services that are more specific than we are, but please feel free to call us as and when you need, and if we can do anything, or just provide the listening ear that you might need, then that is what we will do.”

So, the issue for us is finding local resources to point people at, so that is daycare, that’s the Alzheimer’s society, that might be the local hospice. It’s whatever is in the locality, and for us, knowing about those things is really important, and keeping up to date with what’s coming along.

I think, and this is where social prescribing becomes a bit weird, because in its purest form, we are not therapists. We’re not there as therapists. We’re not there as social workers. We are there simply to say to people, “Understand your problem, you need to go here. You need to go there. Would you like me to refer you in to that or can you manage it for yourself?” But, we develop relationships that border very closely somewhere between professional and a friend, and you then become something different.

And, because we are in the primary care network, we’re there for some time. A social worker may come and go, and you can’t develop that kind of relationship. And again, with a therapist, they might be there one week, but they may not be there the next week. So, we are different to that. So it becomes quite a personal relationship, and what I’d really find difficult is the fact that people are emotionally hanging onto us, because we are seen as that one lifeline out there, often, to help me through this thing that I don’t understand and I don’t know.

So, for me, one of the things that would be really, really useful is – and it might be out there, I don’t know. It might be out there. I don’t know, it might be out there, and you guys might have come across it, is an easy to understand, well-written explanation of what dementia is, the different forms, the different general forms, not the odd ones, but the different forms it might take, and how to alleviate that real deep misery, that the person that you loved is not the person that is there anymore.

I’ve got a lady in case at the moment whose mother has been developing cognitive issues over the past two years. She lives with the daughter and her family. Mum has got a husband and she’s got two teenage children who are going through their own issues at the moment, GCSEs, puberty, all of those things. But in the house with her is her mum, an elderly lady, who is driving her absolutely insane because she cannot seem to understand anything that is said to her, and so everything is being repeated hundreds of times during the day, and it’s making this woman absolutely demented. How does she deal with that? Her intellectual – she’s a lovely woman, but her intellectual capabilities are not high. She’s not very well-educated. How do we cope with that? What do we say to her? Do we ... ?

I mean, I had a family once where – so, I had five brothers and sisters, they were in their 60s, mum was in her 90s, mum rang each of them every single day to ask the same questions. And, I had to go round there, and I had no clue as to what to do. The only thing I could say is, “Listen, your mum does not remember ringing you at all. What you might do is simply block her number, but ring her every day and just check in with her, because she has no knowledge that she’s rung you. She’s no knowledge that she’s rung your brothers and sisters. That might seem really harsh to you, but it’s not, because she doesn’t know. She has no idea.”

Now, that was my best guess at some advice, and actually, when I’d said it, you could feel a palpable sigh of relief in the room, that somebody with what appeared to be some authority, was giving them licence to do something that, on the face of it, looked like it was quite cruel or indifferent. But the reality was, it alleviated them of a problem that was interfering with their lives. They would still check in with mum once a day, each of them would still check in once a day, but the phone would not keep ringing hour after hour after hour after hour. It’s like a drip, drip, drip, water torture. So, these are the kind of things, the issues that many of us face, in my opinion, is dealing with family, not the person that’s got the dementia, because in some ways, they are not aware of what is going on.

Interviewer:	And based on what you say and my experience, is that, actually, they’re most of the times well taken care of in terms of their immediate needs, because that’s what their families are doing for them. So it’s actually almost as if the patient is the caregiver, and I can understand what you’re saying about find it difficult to set those professional boundaries when everybody, as human beings, we have a natural tendency to empathise with people, and a tendency to wanting to help them emotionally.

And I wonder, as a follow-up question, I wanted to ask you if you feel that the role and the responsibility of a social prescriber link worker is also in terms of that emotional support, or whether you think it’s, as you said before, the purer signposting stuff. In other words, where does the boundary sit? What is, for you, maybe that’s kind of a personal question, when do you say, in your mind, “This is my responsibility. From this point on, it’s the person’s or the caregiver’s responsibility to actually seek emotional support through the signposting that I’ve done,” or do you say, “It’s my place to actually emotionally support them?”

Respondent:	By and large, because of the lack of resources, I don’t give up on people because there is nowhere else to go, really, and if we – so, if you understand, and you do, the fact that we’re employed by GPs, we’re there to take the weight off GPs, right? Because, that’s where it’ll go if we don’t do it, then it’s on the GP by and large. People will be ringing in going, “What do I do about mum? What do I do about dad? How do I ... ?” And, it’s not a medical question any longer, it’s simply about management of the social issue within the household. So, I find that, essentially, I’m always there at the end of the phone.

Now, what you get is spikes in all of this. So, you will get a family turn up, and you go and see them, you hear about the problem, and then suddenly they become a big part of your life for maybe up to six months, and there’ll be a constant flow. And at some point within that timeframe, they will start to adjust to the situation and/or drugs might change how the patient is reacting, or they might plateau in their situation. And so, people sort of start to calm down because they understand where it is, and also, sometimes the trajectory of disease is so fast that the issue goes away because they die.

So, I’ve learnt that, whereas before, initially, I was anxious that this person, this family might be with me for the rest of my life, I now see it in terms of, there will be an initial introductory phase, and then there will be a spike of activity, and then it more or less disappears. And then, you might occasionally get a call maybe six months later or a year later, “What do you think about this? What do you think about that? Is there something that you can do about this particular situation?” And so you will kind of get it that way. So, whereas before, I used to have a bit of fear around, “Oh, my God, this person is becoming clingy, or this family is becoming clingy,” now I know that, by and large, we will see a spike in activity, and then it will disappear. But, you do need to be there for them.

Interviewer:	Yes. Will that occur naturally, the termination of rapport, because of different factors, or it is something that you guys as social prescribers work actively on, in terms of terminating that rapport after a while?

Respondent:	It depends on the – I’ve got to be honest, that’s very personal, and I think if I’m talking to my other social prescribing colleagues, they would all do it to suit themselves, their own way of doing it.

Interviewer:	Because, that’s what I’ve heard, that’s why I was asking, generally.

Respondent:	Yes, and I mean, here for instance, we have a very good local representative for the Alzheimer’s Society. She’s an ex-nurse. She’s very warm and empathetic. And normally, if she becomes involved in the case, because we’ll refer the family to her, she will take the load off of us. So, we take the load from the GP, we download it to her, and they will develop the relationship with her. And every now and again, I catch up with her, and she goes, “Oh, my God, I’m being driven mad.” So, we’re all dumping it down somewhere. But, I think she would probably say the same thing, that they come and go. So, she will then get the spike, and then it will start to dissipate, so that’s how it works.

I think, yes, I think updated information about treatments, therapies, local services, is really important. I think one of the big frustrations is the lack of clarity about what social services’ responsibility is for dementia, how they work, and particularly around the financial implications for a family of what might happen. Because the obvious – a lot of it is around, “We need to put dad in a home. Who’s going to pay for that? How does that work financially?” And it’s very difficult, because each county or authority seems to be different in its approach, and how it does it, although it’s mandated from government that this is what you shall and you can do, and can’t.

At the end of the day, it’s about money. It is about money. And that is a huge frustration, because it’s very oblique to us as professionals how social services work. We have good relationship with individual social workers, but at the end of the day, you still it there wondering how on earth did they come to a decision around the future care for dementia patients. And it’s not just dementia patients. Let’s be fair. That’s around the whole of the elderly population.

So, that can cause upset and angst, and the public can become very confused as to my role and a social worker’s role, and I might have to explain a number of times, this is what I do. I don’t do that bit. I can guide you at that bit, but I don’t do that bit, and I don’t do that bit, and this is the responsibility of social services. And then there’s the complaint, “I can’t get hold of the social worker. I can’t ... Can you help me get the social worker? What’s going on?” So there’s a lot of that kind of stuff in it.

Interviewer:	And I suppose, because of the issues that you’ve just said, the barriers to getting hold of social care workers many times, and I’ve come across this a lot, I was wondering whether you think that having that basic overall knowledge on this and that, for example, you mentioned financial issues, now, I’m sure you can’t have, as you rightly said, knowledge about the ins and outs of financial agreements and benefits within dementia, for example, but I was wondering whether you think that having a basic overall knowledge of how it could work, could help the person in need at that moment in time, when they’re waiting for that long waiting list from social care services, to be contacted and supported, whether do you think it’s a place for you to fill in that information gap here and there?

Respondent:	Yes, yes. So I would, if you’re thinking about a website for us to use, then a basic understanding of, if it exists, of what is out there from statutory services, would be helpful. Because, I can only guess at it based on my experiences, but I’ve never seen it formally written anywhere as to what can you expect from social services if dementia is within your family, something along those kind of lines. But, a simple to understand, you should be able to ask for these things, and then it will depend upon your circumstances, because there’s always this, have they got the minimum amount money within the family, or rather, that person?

And therefore, they either meet a threshold or they don’t. And, that’s where it becomes slightly complicated. So, it’s a bit difficult, and of course, legislation changes. So, if you’re going to set up a website, then the thing that I would suggest to you is, you need to resource it so that it stays abreast of whatever’s happening out there in the political, statutory world, because those things can change very fast, and we might not know about it, but it would be helpful to understand.

Interviewer:	So in light of what we’ve just discussed, I suppose one way of looking at how to potentially develop content for this website, let’s call it that way, would be to maybe actually identify some sort of priority topics or issues that caregivers come to you with, say financial, you mentioned the financial aspect, you mentioned, I suppose also potential residential care or accommodation, social care could be another one. You mentioned, definitely, the emotional distress and how to deal with that. And maybe after we prioritise those topics, come up with some sort of general guidance or basic overall guidance on these topics, could that be helpful?

Respondent:	Yes, and again, in amongst that, although you’ve sort of touched on it, respite. So, there will come a point when the family needs to go on holiday. They need a break. It might only be a week or two weeks. It might even be a few days. Whatever. Or it might even be, I need to go out and do some shopping and I need somebody to sit with my husband, my wife, whoever. What are the resources for that? Is there a statutory element to it? Is it all based on private care. How do I go about getting respite for somebody?

Because, there comes a point when you see, they’re right at the end of their tether and you’re saying to them, “I think you need to think about a couple of weeks out of this. You need to go away on holiday. You need to – or you need to be able to have your house back to yourself, or not turn up at your mum’s every single day at God knows what hour, and the only way we can do that is, you need to get them into respite, and how does that work?” Because, there are so many variations on it and they all have different implications in terms of cost and management.

So, what are the basics of that? And if you can say to somebody, “There is a route for you to have a few days out of this,” a lot of people will bite your arm off for it. So, it’s really quite an important thing. There tends to be, when you first meet a family in this situation, and by and large, they’re in distress, and so you pretty much know it’s going to be, “I’ve got to have time out of this. I’ve got ... How can I get my head together? I’ve got other things going on in my life that I need to deal with. What can I do?”

So, respite is really important. Finances are really important. Strangely, the care of the person doesn’t seem to be as high, in a way, because in some senses, they’ve kind of got used to it, but it’s becoming ... But, what they’re realising is, it’s becoming all-consuming. It’s top topic of conversation. It’s the top worry in their heads. It’s the fact that this person sitting in the chair repeating themselves hour after hour is now interrupting the fact that they can’t deal with their daughter’s GCSE issues, which are becoming extremely important, because there’s this battle. So, that’s what’s going on.

Interviewer:	Yes. I also find, interestingly, as you said, that the actual physical strain about caring for somebody is not a trigger for ill-being, as opposed to, as you say, the emotional strain, the continuous and perpetual repetitions and all the disruption that that creates in people’s lives. It’s something that I also observe. And I suppose, going back to thinking about the practical and usable hints and tips that you can give to the workforce to deal with all this stuff, the underlying thing, I suppose, we didn’t touch on it, but it feels that it’s there, it’s how to really communicate with somebody who’s extremely distressed.

Respondent:	Yes, yes.

Interviewer:	And I suppose this is something.

Respondent:	Very much so.

Interviewer:	Yes, so what are some of the tactful ways you use to actually delve into the topic of emotional turmoil, and how to address that? How do you approach those conversations?

Respondent:	Well, do you know what I think about that? I think many of us have learnt not to be very tactful, because actually they don’t want you to beat around the bush. They want you to be as open and honest as you possibly can about what they’re facing. So, by and large, people want a very honest conversation with you, and in some ways you might say things that they don’t want to hear, but they rather have it in black and white on the table in in front of them.

So I’ve learnt not to pussyfoot around some of these issues and just say, “Look, this is going to be really tough. I’m sorry, but there is no way round it,” because say for instance it’s the respite issue, yes, you can get it, but it’s going to cost you a shedload of money because you’re over the threshold and therefore it’s going to cost you X, Y and Z. There are a range of providers out there. These are the typical sort of prices, 1,500 quid a week. You’re going to have to find that money from somewhere.” Well, if your parents put money away, then it’s there for use, isn’t it? Actually, before I forget, there are two other things I just want to put in your head, so sorry for butting in.

Interviewer:	Yes. Yes. No, no, no, go ahead.

Respondent:	One is the issue of lasting powers of attorney, because a lot of people have got to this point and don’t have it. OK, so they’re screwed. They can’t get it because that person cannot now sign that form because they, you know, you can’t rely on it. You can catch it at a very early stage, where then there is a possibility that they could sign a LPA, but you’ve got to get on with it. And sometimes, you have to say to people, “If you want the best outcome, then this is going to make your life immeasurably easier to deal with a lot of practicalities whilst they’re going through it, so you have to do it.”

The second thing is a completely different issue, and that is about a couple, and we come across this often, a very elderly couple, one of whom is caring for the other with dementia, whilst having many of their own problems and maybe comorbidities. And I am staggered at how often those people, even if they’ve been seen by social services, are not being looked after properly. There is very little support for them out there in the community. There is very little statutory support for them in the community.

You might find an elderly man, for instance, and I think it’s in a strange way, because women are, by and large, better carers than men are, women get used to looking after their husband, who may be ill and they kind of deal with it. They’ve already, it’s programmed into them. God, if I sound sexist, I apologise, but that’s what it seems to me to be. But, if you have a man that is caring for his wife, he’s very vulnerable. Dealing with stress is really difficult for him. He will do his best. He’ll put his best face forward. He will try and do as much as he can, but we do see some fairly awful situations where they are locked into a situation, they struggle to ask for help, they don’t know how to receive help and it’s really difficult for them.

And then, their partner dies, and they are utterly bereft. Now, they’ve got nobody to look after. They’ve lost all of their social contact, their family might not be anywhere around, if they’ve got family, and therefore, it’s not merely when you’ve got a dementia patient, it’s what do you do with them after that? How do you take them through the grief? How do you take them then through all of the things that they might be going? And that is, you know, that is the after-effect and that also needs to be addressed.

Interviewer:	You touched on some cultural differences that are quite prominent, from what I’ve heard, across the spectrum. So, you talked about different help seeking attitudes that women versus men, particularly from the older generations might have, and I think that’s part of the issue in a way, the fact that older men, by culture, are expected almost to not seek support.

Respondent:	Yes. Yes.

Interviewer:	And that obviously sets in motion that whole vicious circle. And, continuing a bit on the culture relevant aspects of care, I wanted to ask you, [anonymised], if you notice within your practice, whether there is cultural differences when it comes, for example, to different ethnic groups, or other cultural aspects that might be affecting care, but also help seeking behaviour.

Respondent:	So, this gets tricksy, because the population that we have has got a tiny ethnic – are you still there?

Interviewer:	Yes, don’t worry. Yes. I’m just going to plug in my battery because it’s running low.

Respondent:	Oh, fine. OK. Yes, so, we have a very small ethnic population here. So in terms of dementia, we do not come across it very much at all. Every now and again, we have it, but it’s – I’m trying to think. So, we have, in the area we’re quite a big rural population for our PCN, and our PCN is very large. It’s 65,000 patients, which is a big one for outside of a city. And, that is made up of one large town, one small town/big village, you might call it, and then a couple of large villages.

Out in the rural side, there is barely any ethnic population whatsoever. It is pretty much exclusively white. In the one big town, which is [anonymised], where I am now, which is located near [anonymised], we have quite a large population here, but it’s youngish, so dementia doesn’t really affect it. We do have an Indian population. It’s not big, but it is there. Indian and Pakistani population, where very occasionally, we’ll come across a dementia issue.

But by and large, they keep it within the family. So, we don’t really deal with it. It’s hidden from us if you like, and we simply don’t have the resources. I know it’s on the agenda to somehow reach out into those populations and look at it.

Interestingly, over the past year, we’ve had a massive influx of Chinese, Hong Kong, Chinese. I think a lot of towns round London probably have, actually. They’ve come in under the scheme that Johnson put in for welcoming them away from the Chinese Republic. And, they are bringing – so they are young families, but they’re bringing mums and dads along with them, and I do foresee an issue going on there. But, we have to earn their trust, and that is a project that we have got going. We can see that coming along later. But, I’ve got to be honest, our experience is very, very tiny.

Interviewer:	And to be honest, that’s what I’ve heard from other services as well. Regardless, actually, of location, quite interestingly, so even within very diverse areas, what I’ve heard from social prescribers, is that they actually don’t have that much of a of a pool of clients with different ethnic backgrounds, which I think somehow goes back to what you were saying, about that trust in primary healthcare services. And I think what you said about outreach work in the community, that’s also going to be quite crucial to actually develop that trust.

Respondent:	Yes. Yes.

Interviewer:	So, I’m with you on that one. And, I suppose also what you said about the Chinese community, probably one of the issues that will come up as quite prominently, as you will start supporting that population, is lack of proficiency in English.

Respondent:	Yes.

Interviewer:	Something that I’ve come up with other localities is that, somehow when dementia develops, the person kind of reverts back to their primary language.

Respondent:	Yes.

Interviewer:	If they did speak English, they will start to forget it, and all the issues with translations, and support, and resources to be able to translate will come up.

Respondent:	Yes, yes, yes, very much.

Interviewer:	But this is for the future within your locality, I suppose, as just something to think about. OK. Well, you’ve given me lots of very practical ideas in terms of translating it into the resource. We touched on lots, and I’m very happy with what we’ve discussed. Was there any kind of final areas that you think might be helpful to address in a resource? I mean, we touched on conversation, we touched on emotional well-being. We touched on basic information on social care, finances, power of attorney, and how you guys should be aware of these basic knowledge. We talked about being up-to-date and knowledgeable about local resources, because you work locally, obviously. Was there anything important that comes to –

Respondent:	No, I think, really, I’ve covered the things that I thought were really important. For me, I’m really interested in your project, so please keep me up to date, as and when you can. And, if I can help out in any way further, just let me know.

Interviewer:	Yes, sure. What I’ll do, [anonymised], is I’ll keep you posted on progress, probably around autumn time. I may come back to you to see if you’re interested in taking part in one of those group workshops.

Respondent:	Yes, yes.

Interviewer:	Yes? And one final thing I wanted to say, I’m going to email you a link to a previous website that we’ve developed for people living with dementia. This is for post-diagnostic support, and it’s got some interesting articles in it about the basic overall knowledge we discussed about. So, maybe you can take a look and see if you find it interesting or helpful. There’s a section on, for example, finances, that explains quite in bullet points, quite straightforward what to expect. Since we talked about it, I thought you might find it helpful, so I’ll send that link.

Respondent:	Great. Yes, yes, yes. Yes, yes.

Interviewer:	And I thank you for your time. Thanks for the conversation.

Respondent:	And thank you. I’ve found it really interesting. Thank you.

Interviewer:	Thank you.

Respondent:	And best of luck with the project.

Interviewer:	Thank you so much. Talk soon. Bye bye.

Respondent:	Take care. Cheers. Bye.

[End of recorded material 00:40:28]

**SP20**

[Start of recorded material at 00:00:00]

Interviewer:	... on any of the outputs, so feel free to share what you think is important.

Respondent:	That’s fine.

Interviewer:	Cool. So, shall I start by asking you a bit about yourself, maybe your background, what you do in the area?

Respondent:	Yes. Yes, so currently, I’m Senior Project Lead at [anonymised], and prior to that, I was the Dementia Advice Coordinator for all our dementia projects that we run. And part of that role, but prior to that, I was part of, it’s called our [anonymised]. A lot of people think is stands for Dementia Advice Service, but it doesn’t, actually, but we don’t correct anybody on that one. So, that was set up in – and prior to that, I was an independent living coordinator, which is a little bit like a link worker, but we perhaps do a little bit more in that role.

So, I’ll tell you a little bit about our independent living coordinators. So, they would – the referral would be made to us from perhaps the GP, and it can be self-referral, and we would spend up to about 12 weeks working with that person and their family and carers. And, we’d look at all aspects of their life, which could be economic well-being, so looking at benefits, the social side of things, the home and medication, not that we’re medically trained, but generalised. Home environment, and trips and falls, and all sorts. So, that’s the background I came from, into the dementia side of things.

And, the role of the [anonymised] is, it started off in 2019, and it was just a telephone number for professionals ringing about any non-medical queries that they had. So, it could be, say, a care home phoning and saying, “We’re getting an influx of people that are living with dementia. How can we make our care home more dementia-friendly?” Or it could be, say, a hairdresser phoning and saying, “Mrs Smith comes daily. She thinks it’s her appointment day. How can I best support her? What can I do?” So, it was for those sort of people.

But we also then were connected with Memory Service and Neurology in [anonymised], and anybody that they were seeing new sent a referral through to us, which went into our People Keeping Well programme across the city, which is where our link workers come from. And, the process of that was that the People Keeping Well organisation in the postcode area in [anonymised] that was local community, would then receive the referrals through [unintelligible 00:03:04] a conduit, and they would contact the person and tell them about the resources in their local community, and city-wide, if they wanted, but it was keeping people more connected to their community, that were living with dementia.

That has worked well. Oh, and then we followed up with a six monthly well-being check call until the person was either – may have gone into residential care, sadly may no longer be with us, or they asked us to stop, but that’s ongoing.

It’s expanded from there, this time last year, to incorporate calls now from the general public. So anybody now can ring us with a non-medical question about dementia, which ranges from absolutely anything. So, that’s the DAS side of things, which ties in quite closely with our People Keeping Well organisational link workers, and our ILC services, which are probably just a little bit of a step up.

So, I still oversee that, but my role at the moment is overseeing any new [unintelligible 00:04:13] come in to us, which could be hospital discharge for people living with dementia, through our ILC and our hospital roles, or end of the services, where we’re working within our well-being centres and our memory [unintelligible 00:04:29]. So, quite a lot, quite embedded in dementia at the moment.

Interviewer:	Yes, that’s a lot, but that’s very helpful, Jo, thank you for that. I was wondering what is the link in terms of working relations that you have with primary care networks and social prescribing link workers?

Respondent:	For us, we are – for the ties for our department, and for me? Right, OK. So, for us, with the family carers, if we go out and see somebody, it’s a whole family approach. So, if there are any questions, queries, issues, needs, support needs from the whole family, we take those on board as well. It could be a simply signposting to [anonymised], which is offered basically to everybody we go out and see.

And the social prescribers, again, is a link through the People Keeping Well organisations. We have some of our team are social prescribers as well but not specifically for dementia. So, yes, we do work with our link workers to facilitate the memory cafés and our well-being centres as well, and our provision in the community.

Interviewer:	I see. I understand that, thank you. Now, is it fair to say, because what’s come up in my other interviews is, when I ask the question about what it’s like supporting somebody living with dementia when it comes to their social needs, or any non-medical needs, really, because that’s what social prescribing essentially is, and people often say back to me, “Well, 90, 95 percent of the cases were actually supporting caregivers as opposed to the person living with dementia,” which is kind of – it makes sense. But it’s a bit counterintuitive particularly when you think about person-centred support for a person living with dementia. So, I wanted to ask you what’s your experience in this respect, and how do you see [unintelligible 00:06:45] of social prescribing and person-centredness apply to this specific situation?

Respondent:	Yes. Well, as we go out, as our team go out, and they are with people, I mean, we always say, “The clue’s in the title, Independent Living Coordinator,” and that sort of thing, so it’s to make that person as independent as possible in their own home for as long as possible. So, I mean, we will look at the needs, because sometimes the barriers are there, but the barriers are because of the person that’s caring for them, doesn’t want to let go of the support they’re offering.

Let’s, for example, man and wife, they obviously need some support, both of them, but the person that’s, let’s say the person living with dementia, let’s say that’s the gent, and the wife says, “Well, I married him. That’s my marriage vows. I’ve got to do everything for him,” and we try and offer the support so they get some respite, but the person, again, is then getting something socially themselves out of it, so they’re getting a stimulation. So, we talk to a lot of people about [anonymised] where they could both go together, but we also offer things like our well-being centres, which is specifically for that person, so they get in the – again, as I said, the person-centred approach, because it’s about them. So, what we’re delivering is about them, and we don’t want the carer there, or the family member.

So it’s like, in a nice way, it's convincing the caregiver that this is their respite time, and that this person’s going to be stimulated so that they can be with us longer because the brain’s more active. And we sort of dress it up by saying, “Well, this person’s going to be away from you for the day, but then they’ve got something different to come back and have a conversation with you.” It’s often, as I say, the caregiver that puts that barrier in place, says, “Oh, they’ve got to go with me, they can’t go alone,” and when we eventually get that person on their own, we find that it’s something that they want to do.

And we do tend to find that, when we go out, when we’re talking to perhaps a couple, and we might get this person at the side of us that’s chipping in all the time, we’ll make sure to remember who the client is. Try to direct only some questions to those who can fill in the gap. Explain to families that they're also going to benefit from the person deciding what it is that they want out of it.

Interviewer:	Yes. I think this is a precious, practical tip for any social prescribing link workers, it’s a tendency, sometimes also kind of a protection mechanism that caregivers put in place, by kind of overstepping. And again, sometimes it’s out of protection, but it happens, and I can understand why you use this kind of strategy to try and address as much as possible the person there. Also, you touched on resistance, which seems to be a big thing in caregivers generally speaking, and a bit of gatekeeping there. I wanted to follow up with a question around, given your experience, how do you work around and work through that resistance? Do you have any tips? Do you have any strategies?

Respondent:	I just try and probably explain to them both that anything that we’re doing is in the best interests of both of them, really, and try and get them both on board, but try and convince the caregiver, the supporter, that they’re going to benefit from this other person being more socially active, getting different types of stimulation, being apart from each other. I do sometimes think that the caregiver thinks, “Well, you’re not here for 24 hours a day, and I am,” and I think that’s some of the, you know, “You can walk out that door and I can’t,” but yes, I try and approach it differently, where you’re offering a person, the person living with dementia something, but you’re then counterbalancing it, and it’s like it’s a gift for the caregiver, because we’re supporting that. I suppose I don’t think about it, because I’ve got a little bit of a gift of the gab, where I just take the situation and turn it slightly. Yes, I don’t know how I do it, I just think ... Yes.

Interviewer:	Yes, I think it comes with experience.

Respondent:	[Crosstalk], yes, I can just sort of turn things on their head and make sure that the caregivers, it’s not like, “Well, it’d be better. I suppose, in a way, it’s not just talking to the caregiver and saying, “Oh, well, this would be better for your husband if we did this,” it’s, “This’ll be better for you because your husband’s doing this,” so it’s –

Interviewer:	Yes. And I suppose it’s making sure that they know that you’re actually empowering them, thinking about a time where you’re not going to be there to support them, because I think it goes back to what you were saying before, about caregivers potentially saying, “Well, you’re only here for some time, and then you’re gone and I’m dealing with it afterwards.” And I suppose it’s, as you say, that mechanism of letting them be aware that you’re providing some tools that they’ll be able to use when you’re not there after.

Respondent:	Yes.

Interviewer:	So, yes, I understand that. Do you ever find any kind of – do you find that people develop these dependency issues on your support? In other words, do you find that, because you’re there and they finally have somebody who’s listening to them, a one reference person they can talk to, do you find that over time they develop a bit of a dependency, and that therefore it’s difficult at the end of your support time to terminate that?

Respondent:	Yes, and again, that does happen, and again, I always, in a light-hearted way, I’ll say to them, “The clue’s in the title. We’re an independent living coordinator.” And yes, you do sometimes get those people that do hang on, and then you get to the end of the service time and you’ve met all their needs, and then they suddenly think of something else. And what I tend to do at that point is, if it’s something that, I just think that they’re hanging on, I will signpost them and empower them to do something on their own, and then say, “Well, I’ll give you this information, but now we need to move on to helping somebody else. You can come back to us in the future.”

But, I always point out at that point, I’ll say, “You can come back to Age [anonymised] in the future, and we can always help you. It may not be me. It will often be one of my colleagues, because we tend to rotate,” and then that just gives them that little warning that I’m not on tap, and that they’re not just going to pick the phone up and get me every time.

One of the big things that we do do is, we try not to give our work’s mobile phone number out, so Everybody has to come through the switchboard Team, who are instructed to say - Oh, sorry, Mrs. X, you're not currently working with Y. Is there anything I can help you with? I can open a case up for you with one of our other team. We also tend to rotate to just avoid working with the same clients over and over.

Interviewer:	Yes, I suppose that sets some good boundaries that leaves the person independent, which is good for them, actually, in the long term, so it makes sense. I was wondering, in terms of what you touched on before, you said some people, they come towards termination of support, and they come up with a new issue, right? I was wondering, what is the kind of – how do you decide on the length of time that you’re dedicating to a person? Is it based on prioritising, say, one need, and once you’ve address that, it’s done? Is it several needs? Is it based on actually a time limit? How do you decide about length of support?

Respondent:	We don’t. We just have an average that says we roughly work with a person for around 12 weeks. We’ve two different services here. I’ll just start with this one first. So, we’ll just work with somebody for around 12 weeks-ish, but when we get there, we may have been – it may be a referral that’s come through to us, and it might say, “A benefit need.” So, we would go along, and when we get there, they might go, “Oh, no, I’ve got my attendance allowance now. It’s in place. But, I need equipment and adaptions. I need A, B, C, D,” and then we get onto a discussion about something like pension credit.

So, we would just work through all those – we would find out at that time what their needs were at that time, which may be something totally different to what’s come on the referral form, and just work with them for the length of time. And if it is a benefit and they’re waiting for an outcome, we might pop them on hold, the case on hold and say, “Well, you’ll not hear from us for a while. We’ve addressed everything else. We’ll just deal with what happens when we’ve got the benefit result through, and then we’ll pick up from there.”

And some people, again, it’s just your skill of closing the case, but some people, it doesn’t have to be just one need, it doesn’t have to be 12 weeks, it could be you’ve dealt with what they needed, you’ve got back in touch with them, and they’ve said, “Oh, no, I’m all right at the moment, thank you very much,” and you might’ve had two visits and it might’ve took you six weeks, or something like that.

But our other services, which are the people keeping Well link worker services as well, is we get in touch with them, and they may just have some basic needs. Address those, and then we make a six month ongoing courtesy call to them, so we’re keeping in touch with them. And we tend to find with those people, when we ring them initially, because that’s when they’ve been newly diagnosed, that’s like, “I’m just trying to absorb all this information,” that they don’t want anything to do with us.

So we’ll say, “Well, we’ll ring you again in six months, and you can contact us in the meantime,” and then when we make that six month call, it’s like, “Oh, yes,” reality’s sunk in, they now need help with A, B, C and D. We might deal with those, and then in six months’ time, something else might’ve arisen, so we have those. But that’s a little bit more of a light touch, yes.

Interviewer:	Yes. It’s really interesting, this point you touched upon, [anonymised], which is – and correct me if I’m wrong, sometimes at diagnosis point, people are just too overwhelmed to even accept support. They need to marinate a bit in the idea and settle down, in a way, which is interesting, because intuitively, one would say that is the moment when probably they need more support. But actually, your experience tells you that sometimes it’s giving them time, of course leaving numbers so that they know there’s somebody in the background if they need to, but it’s actually later on that you find it’s people need more support.

Respondent:	Yes. We tend to find, as well, that we have a system with our memory clinic in [anonymised], that anybody that is on their – they are, I’m just trying to think of how many weeks behind they are because of the pandemic. I think they’re something along the lines of 42 weeks in arrears of their waiting list to be seen for diagnosis. So, anybody that is put on their waiting list gets a letter from the memory service saying, “You are on the waiting list. We will be in touch. If there’s any medical queries,” blah-di, blah-di, blah, and then there is a section where they are directed to our dementia advice [anonymised] line for any other queries, non-medical.

And probably, more people prior to their diagnosis contact us because they want information about dementia than people, when we’ve made the call, when they’ve had the diagnosis, want that information then. So, I think, because people know they’re on a waiting list, they want to know, they want to be forearmed, so yes, we get a lot of calls saying, “I’m on this waiting list. I don’t know anything about dementia.” It could be themselves, it could be a family member. “What can you tell me?”

Interviewer:	Yes, and that’s sort of prep work, in a way, which will come in handy when that news just is officialised, in a way. Do you ever find that, pre-diagnosis, you’re asked to provide social prescribing support to people?

Respondent:	Yes. Yes, so we will direct those to things in their local community, again, just for support. And at that time, a lot of people might say, “I’m not ready for it yet, but as long as I know what’s there, and I know what’s out there.” I think it’s more, for us as well, a lot of people want to engage, and just have that conversation with somebody, and just perhaps get their anxiety and fears off their chest.

Interviewer:	Yes.

Respondent:	And sometimes, they’ve got no needs, they just want to talk about it, and again, that line for us is what we can do to support people, just by listening.

Interviewer:	Yes, I agree. Sometimes it’s about demystifying the whole thing. It’s giving a name to it, and knowing that there’s support out there if you need it for the future. It’s that planning ahead thing that seems to be so really helpful for people in general. OK, that’s very helpful. I’m wondering, in terms of social prescribing, obviously it’s across the spectrum, it’s different needs. I was wondering, in terms of potentially prioritising some of the most recurring areas that you’re asked to address, people mentioned finance, people mentioned emotional well-being for caregivers, and respite, which is sort of interlinked, in a way. Other social prescribing link workers mentioned social care as well, and all the difficulties with bureaucracy and that stuff, and other people mentioned the legal aspects, so power of attorney. Would you say these reflect the areas you’re asked to provide support around?

Respondent:	Yes, definitely. Definitely, on a daily basis.

Interviewer:	Yes, OK. Are there any kind of areas that, in your experience, people ask support for?

Respondent:	Any other areas?

Interviewer:	Mm.

Respondent:	It generally starts with those. It might lead on to other things, as you have that guided conversation, but generally, I’d say a lot will ring first for either finance or social side of things. A lot, we get a lot of calls about social care, and again, tied back with the finances of paying for the care. And, don’t even know where to start. I mean, I think there’s a lot of support needed, and again, this might help with the resources that you’re developing, is guidelines of where to go to even get a financial assessment. Most people, I think, until – especially, because we have a lot of people, we deal a lot with people with young onset, so people just don’t know where to turn. They’ve suddenly gone from working to now being unable to work through their general health and well-being, who’s going to support the family?

Interviewer:	Yes. So, would you say that, because you’re asked these questions by people when they contact you, would you say that – I’m always thinking about this resource – would you say that providing some basic guidance to social prescribers on, say, the structure of social care system, or potentially some of the structure in terms of the benefits and finances, just some basic knowledge, is helpful to know?

Respondent:	Yes.

Interviewer:	Because, I suppose one rationale would also be that even if you signpost people to social care or to finance advisors, sometimes the waiting lists might be so long that people might need that basic information there and then.

Respondent:	Yes.

Interviewer:	So, if the social prescriber knows at least the basics, they might be able to give those initial pointers.

Respondent:	Definitely. Definitely. I think that’d be really useful for a lot of people, because when we’re dealing with a person that’s living with dementia, or their family and friends, it’s OK signposting them to, like you say, a telephone number, and they can’t get through, but then they don’t know – that person doesn’t know the terminology to ask, or what they’re asking for. You can’t just pick the phone up and ring the local council up and say, “My husband’s got dementia. I need help,” and they’ll go, “What with?” And they’ll go, “Well, I don’t know.”

So, if we can have some guidelines, and then they can feed into the person that’s living with dementia and their family, it just makes it – I think it’s something as simple as a step by step approach of that – and like you say, benefits as well, and “You may be entitled to…” Yes. It would be [so helpful? 00:24:55]. We find a lot of people, a lot of our social prescribers in the city are OK with overviews of attendance allowance and things like that, but when it comes to how things overlap, and how things fit in with each other, that’s where they’re stumped.

Interviewer:	OK.

Respondent:	I mean, we’re lucky because all our team, we’re an alternative office for the DWP, so we’re tall trained in benefits advice for over 65s. When it comes to the younger person living with dementia, we all know about PIP. We’re not actually funded to deliver help with PIP, but we do. And again, it’s for everybody. It’s like, why should we not know about it? Why should we not – well, we know why, because the DWP want to hang onto their coffers a little bit longer, but we should be able to give people an insight into what could help them.

Interviewer:	Yes. And in fact, what you’re saying makes me think of a resource in terms of almost, you touched on this, almost such a checklist type of thing where, you go by areas, which are the ones that we can prioritise through these discussions with you guys, to see what the most important ones are. And then for each area, almost have a checklist type of thing, where you explain very practically and concisely the main aspects of that specific area. When it comes to finances, it could be around entitlements to benefits, around council tax exemption, that really basic kind of structure so that a social prescribing link worker might have, as you say, that overall basic knowledge to be able to answer some of those questions. And, I wanted to ask you, [anonymised], you said that sometimes the issue comes when there’s overlap between the areas. Can I ask you, practically, what you meant just so I have an idea?

Respondent:	So, was I talking about the young onset, then?

Interviewer:	Maybe, yes.

Respondent:	Right, OK. So, I was – this is what I was referring to, I think, was the overlap of under 65 age benefits and over 65 age benefits. Oh, sorry, I know what I was talking about. I remember now. So, I was talking about people receiving attendance allowance that then don’t really realise that they may be eligible for pension credit, and they don’t know the eligibility criteria for the pension credit, and the fact that between the attendance allowance and the pension credit, if they apply as a couple for carers’ allowance, they get the underlying eligibility that will give them more pension credit income. So it’s those bits, it’s the DWP stuff that’s hidden from plain sight, really.

So, we’re not maximising people’s income because we’re not all sure of where people can maximise that money from. And, it’s just knowing the system, the DWP system, really. And I don’t mean to say working the system, it’s just the fact that people can get so much more if they understand the route through. Before you even get – when you’ve got your pension credit and you’re then going to end up with a zero council tax bill, where if you’ve only just got attendance allowance, you’re going to get a percentage off your council tax bill.

Interviewer:	Yes, I can see the overlap, and how it affects the whole finance system for the person.

Respondent:	Yes.

Interviewer:	OK, no, that’s very helpful. Thanks for clarifying.

Respondent:	One of the other areas – sorry, one of the other areas I’ve just been thinking about, while we were talking about for perhaps resources is, there’s questions, I think, that are sometimes presented to the link workers about people’s health and well-being. Now, the only way I can tackle this is, we have a training programme, but not for customers, it’s for the team, like the link workers and things like that, where they come across different questions, I suppose, that’ve been raised by customers, which could be – we’ve been working in a hospital recently, and we’ve put on a training course about helping people who are non-verbal.

And, I think these people are going out and coming across barriers. It could be, we did a recent course on sex and relationships especially in care homes, we did a course on that, and people are coming across these questions, and they don’t know where to go to. So, again, that feeds into our service because then we put on the training courses. But, a resource where people can be directed, and maybe, if you want some examples of that, I can find out some of the titles of some of the courses that we’ve put on, which have come from the queries that we’ve had, which might help you to, if you think you go down that road and think that might be useful as a resource.

Interviewer:	Yes, because in a way, from what you’re saying, it’s been developed with the kind of framework that I have in mind. It’s stemmed from the priorities that people had in terms of the questions, and then it was developed based on those. So, it reflects what people are interested in.

Respondent:	Yes.

Interviewer:	Yes, it would be great, [anonymised], if you could, yes, if you could dig in and find some of those areas, that would be really helpful.

Respondent:	OK, I’ll do that, definitely.

Interviewer:	Thank you so much. Is there anything, because that’s also quite interesting, people have often told me that when caregivers come for support, it is actually rarely related to providing care to the person, as in the physical stuff that you do, it’s interestingly more about the impact that it has on emotional well-being and all that.

Respondent:	Yes. Yes.

Interviewer:	Do you find that, as well?

Respondent:	Yes, definitely. Definitely. I think sometimes when the person is – and I think sometimes it’s because, again, going back to that husband/wife situation, where they just care as a natural progression, because it’s what they’ve done all their life, and that’s what they married them for, and those sort of situations, I think when it comes to a case of where perhaps somebody’s continence has declined, and then they are starting to see things a little bit more, that it’s more than just general caregiving, then the questions will arise.

But, at that stage, I think they’re then already looking for professional are. They can manage the day to day stuff. But yes, when they come along, it is more about their own emotional and mental well-being, because they’re dealing with something that they weren’t expecting to deal with in their life, and they don’t know how to deal with it, because where do they go for the answers?

Interviewer:	Yes. Yes, and I suppose there’s also something about those cultural expectations and background, particularly with the older generations, and I see, particularly with the older men struggling to seek help because of that culture background that they do have. But, it’s interesting to notice that one would associate caring duties being heavier on the physical side, but actually that is kind of almost accounted for or expected, in a way, whereas with the emotional well-being, there’s lots of struggle.

Respondent:	Yes.

Interviewer:	OK. I just wanted to really bounce off this idea with you, but I see that you kind of agree with this trend. OK.

Respondent:	Yes.

Interviewer:	So, [anonymised]. I’m done with my questions. This was really helpful, and you gave me lots of really excellent pointers as to translate this into something meaningful. Was there any final point that you wanted to discuss, or are you happy with our conversation?

Respondent:	No, I’m happy with that so far, and if you need anything else from us, just drop me an email, and I’m happy to help.

Interviewer:	Thank you so much. So, if you could find some information about that, that would be really helpful.

Respondent:	Yes.

Interviewer:	Also, what I’ll do is, probably around autumn time, I’ll give you a bit of an update via email and see if you guys are interested in taking part, potentially, in one of the workshops, which involves also stakeholders, so Age UK would be really, really appropriate to be involved at that stage too. But, I’ll come back to you maybe in autumn time, about that.

Respondent:	OK, lovely. All right.

Interviewer:	OK, awesome. Thank you so much for your time, Jo.

Respondent:	All right. Lovely to meet you.

Interviewer:	Take care. You too.

Respondent:	Take care. Bye bye.

Interviewer:	Bye bye. Bye.

[End of recorded material 00:34:55]

**SP21**

[Start of recorded material at 00:00:00]

Interviewer:	Can I ask you, just to begin with, a bit about yourself in terms of your background, what you do, and your experience with people living with dementia, if any, of course?

Respondent:	Sure. So, I currently work as a senior social prescriber in [anonymised]. I’ve been here for about 3.5 years. I’ve been working across – I think I’ve worked in five different doctors’ surgeries, so I’ve got a very good picture of what a GP surgery – how they work. And obviously, in the one I’m working now, I’ve been there for about two years, so I’ve got a good – I think I’ve worked – I now have very good relationships, I think is the right way of saying, with the doctors, with this team. So, everybody knows what I do, and everybody knows, so I feel comfortable there. And, it works really well, I think, in a clinical setting, but they know what I do, I know what they do, and we can ping pong, I can email doctors, they can email me. It works really well.

My background actually is not – I used to work for many years with people with learning disabilities and mental health here and abroad, and more recently, I’ve worked with blind/deaf. So, I’ve done a lot of social care, that’s my background.

And, I would say that I was trying to quantify it, because I know it’s quite important to quantify, I would say I can’t really give you a number, but I would say that generally if I see or speak to ten to 15 people, or 20 people in a week, it’s hard to quantify, I would say at least ten percent of them have some connection to dementia. So, I think it’s quite a high percentage of people, and it’s definitely on the increase.

On a personal note, I’ve actually got a mother with dementia, but she doesn’t live in the country, so I’ve got that experience as well, with her carer, which does sort of, I suppose, indirectly affect the way I work. But generally, my caseload at the moment, I would say I think I’ve got about ten to 20 percent of my patients do have dementia at the moment.

Interviewer:	OK, so it’s quite a substantial number, from what you’re saying.

Respondent:	Yes.

Interviewer:	Am I correct in saying, and please correct me, but that’s what I’ve learned from other social prescribers around the country is that, when you do get a referral for somebody living with dementia, it’s actually most of the times to support the family and the caregiver, or if not, if that’s not the case, most of the times, you actually speak with the family, even if it’s about the person?

Respondent:	It’s interesting, I would say yes and no. I’ve actually got a good case study of a woman that, I was supporting her husband, he had Parkinson’s, she was recently diagnosed with a dementia about three or four months ago. So, I would say, yes, the majority are usually – I am talking more, but I do have a number of people that do have dementia that talk to me, too. So, I would say majority of yes, and some people, I do actually talk to people with dementia as well, so kind of a mix.

Interviewer:	That’s uplifting because obviously social prescribing is person-centred, so it’s great to hear that you do deal with the actual person living with dementia.

Respondent:	Yes.

Interviewer:	So, let’s think about the actual client living with dementia, and some of the learning points that are based on your experience. So, for example, because this resource needs to be very practical, so what I’m looking for is, actually, based on your extensive experience, because you’ve got lots, I wanted to ask you some specific things to be mindful about when it comes to people living with dementia. So, let’s start, for example, when you’re starting your first consultation with them, what are some of the things that social prescribers need to be aware about, some of the barriers, maybe, and how to tackle them?

Respondent:	I would say the first barriers, you’ve actually also mentioned, that you’re not person-centred. A lot of time, the doctor will refer you to the daughter, the son, the husband, which I think, actually, obviously for communication, it’s the way it works, but I don’t think it’s always so – there’s an issue there, that we’re not actually talking always to the person with dementia, we’re talking to the husband, the wife, the son, which I don’t think is ideal. I don’t quite know how you’d do it otherwise. But I’ve got some experience that, if you remind me to tell you something specific about that.

Interviewer:	Yes.

Respondent:	I’ve done a few experiences of – I’ve been to a memory service and I’ve been to a dementia café, and again, I feel that there’s something quite – I don’t think – the reason I was quite keen to talk to you, I feel as a social prescriber, I’ve learnt and I’m still learning, and I’ve got my own experiences, there’s definitely a lack of awareness and understanding. And on one hand, the GP’s very understanding, and he’ll write me a note, and say something, but I think that’s on the first, from the original – the referral is not a good referral, because as you say, it’s often to the person.

And yes, the system doesn’t seem to work well with dementia. I’m a bit confused. I don’t like the way I get that, and I find that if I want to talk to somebody, I’m kind of working backwards sometimes, because I’m trying to work out what information they’ve got. The only thing that I think is the magic of social prescribing is that we have access to doctors’ notes, so generally, as a social prescriber, I spend a lot of time reading the notes. So, I’ll go back to the original diagnosis, and I’ll read as much as I can. But I’m always lacking in information, and that’s something that is not good either.

Interviewer:	But that’s a great point, and let’s explore this a bit more. So, you’re say it is good practice, and it’s actually helpful to go back to the clinical notes and read them, but also you’re saying that, usually, they’re quite kind of vague, or they don’t give you a good picture. So, how do you take that picture after you’re read the notes?

Respondent:	I’m just going to give you one more sentence. The main issue is, I don’t know if you know how it works, but we’re working on EMIS, which is a GP case –

Interviewer:	Yes.

Respondent:	We don’t have access to the hospital notes, and this baffles me. I say this at every meeting, I say, “Isn’t it time that we share notes?” So, I do occasionally get a chance to see the letters, but when I have been involved with cases, I’ve always said, “Please, it doesn’t matter if it’s a person with dementia, involve me with as many people, or as many people that are involved with dementia,” because I think there’s a bit of an issue there, that we’re not getting the right information at the start. So, that’s me slightly digressing from the question, though.

Interviewer:	No, but it’s helpful.

Respondent:	I’ve forgotten what the question –

Interviewer:	Yes, so the question was, given the vagueness of the information that you’re provided with, how do you actually get to know the person? What are some of the good strategies that you use?

Respondent:	One of our best strategies, and I know this is something that’s really – not a lot of social prescribers do this – we’ve got a different role as well, which I don’t want to go into, but generally, we do home visits, and I think that’s really important, that I actually get to meet the person. I think if I can get to meet the person, or even to hear the person talking, it’s very important that I see the patient. I think it’s the missing link in the person-centred. If you haven’t got the person, I’m kind of missing it, and that’s something I really – I try to meet the person with dementia, or the family. I try to get more – I think you need to meet the person. I think it’s a problem when we don’t see the person, and we’re only hearing second-hand information.

Interviewer:	Yes, that’s one thing that’s been brought up by lots of people that I’ve interviewed, it’s that lack of face to face contact, is actually a big barrier to knowing where the person is, what is their situation.

Respondent:	I’m giving my example of my case study. I probably would repeat the same a lot, but she was diagnosed three or four months ago, because I’ve accessed her notes, I’ve read her notes, I’ve read her hospital, I’ve met her several times, we speak general every six weeks, and the first thing she’ll say to me, “[anonymised], can you see any difference?” And I’ll say to you, “No, actually, you sound as sharp as you were three months ago, and I think you could probably tell me how to do things, never mind me having ... ” But, I know that she’s been diagnosed. She’s had her scan, and she’s obviously got early dementia, but she’s obviously at the very early stages. So, it’s a bit of a challenge, I think, but I think definitely, me seeing the person is very important, or at least trying to have some sort of contact with the person.

Interviewer:	Face to face contact. Can I ask you,. [anonymised], because this is really interesting, and I think important, when you do go and see the person physically in their environment, what are some, based on your experience, what are some of the signs that you look for to understand where they are, how healthy they are in their environment, in their presence?

Respondent:	I mean, it’s interesting, part of what we do with other patients, we do the – I’ve forgotten the name of it, it’s not the – there’s a test you do, a memory test. So, we do a test. I don’t like the memory tests, because I think it just stresses people, and I don’t think it’s actually suitable, but obviously, that’s one of the things, if I’m doing a general assessment, then the nurse of somebody will have assessed the person, so already, I’d have got a kind of picture of what the person’s doing.

But, I think generally, I find with a lot of these home visits, it’s about trying to break the ice, because as a social prescriber, we do this over periods of appointments. So, I’m trying to connect with the person, I think, mostly, try to get to know the person, try to understand the patient, and try to explain to the patient what I’m trying to do. And I’d usually go for the more basic things, though, I’m here to help you get a benefit, or I’m here to help you with that.

But, I think what will come out in the conversation very quickly, I know from a home visit yesterday to somebody with Parkinson’s, very quickly, once the person realises, as the trust is increasing, they will tell me how they’re feeling. They will say, “Oh, I’m feeling a bit confused,” and very quickly, you find people do actually tell you about the dementia. They do actually talk about their situation, which is interesting.

Interviewer:	That’s very interesting. Do you ever encounter any resistance from people, or mistrust initially? And if so, how do you combat that?

Respondent:	Yes, yes. I start by asking questions like – How was your day? Talk to me about your day – Humour also works well. Or if they've got a dog, I'd ask about its name, and the next time I’ll remember the name. If you show a little bit of humanity and interest, clients open up. But, I have to admit, there is issues. It doesn’t always work. It’s not a guaranteed – there is always this kind of initial mistrust, which sometimes can take sometimes several phone calls or several meetings before people actually do become ... I think, though, it’s less, though. I think by the second visit, usually, when they realise that you’re reliable, and you’re trustworthy, they do start talking to you a bit more, yes.

Interviewer:	Yes. So it’s the initial resistance, but then they seem to develop that trust. And I wonder, do you ever encounter situations where, on a second visit, for example, or on a third visit, they will not remember who you are? Or, do they usually remember?

Respondent:	I think, because I’m usually – in that kind of situation, I’d probably be talking to either the wife or the person that’s caring, so I find, again, this is one of the issues, that there is a possibility that they won’t remember me. I have got patients that I’ve spoken to several times, and I have to remind them, but in a nice way. I think it’s just a gentle reminder. I’ll mention something I mentioned the time before, if it’s a picture on the wall, or there’s something memorable that will suddenly – and that will kind of click, and they will suddenly click back. I do realise that, if it’s somebody with a bit more advanced dementia, it is a bit more confusing for them. They don’t always remember you, and that is a bit challenging. And, that means I’m usually working with the carer or the wife, or the son. And that isn’t ideal but I think that’s probably the only way you can work, if they a bit confused.

Interviewer:	Yes. And, you mentioned the memorable thing that helps something click. Can you give me an example? That’s very helpful.

Respondent:	Usually, I always find I end up talking about either foods, so it’s something we’ve talked about last time, we’d mentioned, is it a cup of tea or the biscuit, or the cake, or ... ? It’s something very small, but it’s something that will suddenly, they will remember it. It can be anything. It really is. It can be very general, but usually, I use food, actually, as one of my reminders.

Interviewer:	Yes. OK, no, I get that. It’s something that’s very relatable for everyone, isn’t it?

Respondent:	Yes.

Interviewer:	You find that connection, yes, midground, I understand it. [anonymised], how do you, when you start a conversation with somebody living with dementia, how do you understand what their main need or priority to work with is?

Respondent:	I think I’ve noticed with the couple of people that I’m working with at the moment, they are quite keen to talk about getting their matters – or what’s the expression? Getting their situation organised. They’re already, a lot of the times, they’re already worrying about what’s next, so I say to them, “We need to sort this out, we need to check out, we need to plan ahead,” even though I’m taking it more into the words planning, but they will say, “I need to talk to you about something,” because I think they’re very concerned, it’s an ongoing concern, you can feel that, that they want to talk about what’s going to happen if, when ... And it’s something that comes up a lot in the conversations, and that’s more like the fact of what’s the benefit or the care, or what’s going to happen, those kind of questions come up quite a lot.

Interviewer:	Is it fair to say that, that’s what I’ve heard from other social prescribers, it is more around the planning, it’s more around finances, it’s more around information and power of attorney.

Respondent:	Yes.

Interviewer:	And less about actually the physical stuff about dementia?

Respondent:	Again, I think it’s quite mixed. I don’t think – it’s very hard to [crosstalk] because I think every person with dementia I’ve met is different. I’ve learnt that as well, that everybody’s got a different thing. And I think that sometimes it can be something like that, but it could be something else. It can be something very basic to do with their day to day life. It’ll come to me in a minute, of an example, but I can think maybe it’s just something about even just going out, and saying they want to go out more, and that’s not really related. So, I think their need to still get out if they can, or if they can’t, then they won’t be getting out, then it’s something else more specific, maybe.

Interviewer:	OK, yes. It’s very varied, as dementia is such a wide –

Respondent:	Yes, yes.

Interviewer:	Yes. I get that. And do you ever encounter any sort of lack of motivation to engage in the plans that you make with the person?

Respondent:	Yes, yes.

Interviewer:	And, how do you address those?

Respondent:	Again, unfortunately, I feel that we’re kind of getting away from the person, and we’re ending up, again, talking to the carer or the mother or the partner about what we should be doing. I find that it’s a little bit – yes, it’s a little bit upsetting that you’re kind of – it doesn’t feel comfortable, the way you’re focussing, if the person in the – which I think isn’t ideal, but I think at the end of the day, if it’s the carer, the wife, then that person’s spending a lot of time with that person. So I think it’s OK. It’s not ideal, but it’s OK to be talking, I guess, to the carer or the partner about practical help that they might –

Interviewer:	Yes. I suppose they know the person in and out, so they also know the mechanisms maybe through which to engage the person in the process, I suppose. I was wondering whether you think, in that case, is it a case of actually acknowledging that the person doesn’t want to engage, and we need to be person-centred and just leave them be, or is it a case of actually listening to the caregiver, and what they think should be done?

Respondent:	Yes, no, no, we do – I do have – you are listening to the caregiver because the caregiver is the one that becomes the person’s voice. Not always perfectly, but at least, I think they would know what the person needs or wants, at least they’d have a good idea. I’d like to believe that. I’m not sure always, but the caregiver is probably the person you’re going to be talking to, yes. And I think that’s just realistic. As much as I’d want to talk more to the person, I think the caregiver’s the one we do communicate with more.

Interviewer:	Yes, I suppose the right word that you just said is being realistic, and pragmatic, and I think based on your experience, maybe you find some commonalities with people with an intellectual disability.

Respondent:	Yes, and no. It’s interesting because I will mention it, I did actually recently go to a memory, Alzheimer activity. I was curious, and I wanted to refer people. And, I did notice the carers taking a very prominent role, but they were always trying to make the person try also to give their – so it’s kind of like trying to share it, I guess, how they’re going to share the voice, as well, I guess is the right way of saying it, sharing the voice or promoting their voice or their opinions through –

Interviewer:	Interesting. Interesting. So, you find differences with intellectual disability.

Respondent:	Yes. I think it is different because I think with learning disabilities, my experiences was, yes, there’s always that feeling that the carer is talking for the person with the learning disability, but I’ve found, I think, over my years of experience, I think the difference is, with someone with learning disabilities, they can actually have a voice, and they become more vocal, where – so it’s probably the opposite, because they’re gaining a voice, whereas the person with dementia is losing their voice, so there’s probably quite s difference, if that makes sense.

Interviewer:	Yes, no, it makes perfect sense, and I really like the picture that you’ve used, one is gaining the voice, the other one is losing, because actually, that’s what’s happening. It’s really helpful, this way of putting things. Another question that I had is, because you’ve got such extensive experience, and also because you work in [anonymised]., which is, London is very multicultural, I wanted to ask around whether you have experience of supporting somebody from different cultural backgrounds?

Respondent:	It’s interesting, that activity that I went to, I was quite surprised that the majority of people were from the Caribbean background. There was, I would say five people were Caribbean, one man was from Syria, it was quite obvious. One person was white British, and one person was also from a Middle Eastern country. So I would say majority of Caribbean, and quite a few Middle Eastern, and very few white British. I find the white British in general, I know they have it, but I don’t – they seem to be the ones that we don’t seem to see at these kind of activities, so I noticed that before.

Interviewer:	That’s interesting. How do you explain that?

Respondent:	I think, because generally [anonymised], are quite multicultural to what – and I think there’s definitely, you can see that even from the staff, the meetings I talk to, there’s definitely – I don’t know what the statistics are. There seems to be more people from different ethnic backgrounds, and less from the British. Or the fact that the British people, the white British are less involved. I don’t know. I can’t always put a finger on it, or maybe it’s just it's the majority of the community, It’s hard to actually know exactly.

Interviewer:	Yes, so would it be fair to say that if you were referred a patient from a different cultural background, would you feel comfortable knowing what are some of the activities that might be more relevant to them?

Respondent:	I think I’ve learnt, because I think before I came here, I didn’t have – I had different kind of ethnics, I had actually more focussed – it was actually with different ethnicities, whereas here, I’ve learnt a lot, a good percentage of people are African, Caribbean, Indian. There’s a larger majority, and I think I’ve learnt. It’s part of the things that I’ve learnt. I’d like to have learnt more about it, but I’m learning as we go along, their cultures, and they will tell me about their cultures, and I can hear in their voices and in their stories, there is differences, quite a big difference between each culture, the Muslim culture, the Christian culture, and then the Jewish culture. It’s quite a big – there’s very different cultures, I can see from the way people talk to me. They don’t always say it, but they say it probably indirectly, about their culture, they will talk about certain things that, obviously, religious culture.

Interviewer:	Yes. But, from what you’re saying, based on that experience of the dementia café, I wonder whether it’s actually important to have culture specific services, or actually a mix of people from different cultural backgrounds is the best way to go?

Respondent:	That’s a big question, because we talk about it all the time. I know with some – it’s funny, because we were talking recently about talking therapy, and I find with talking therapy, for example, generally people are very – they don’t like the idea of talking, they don’t like the idea of therapy, and it’s generally cross culture. Can be any age group, and it’s not just ethnic minorities that – it’s generally a ... Whereas I think with this, with the dementia café, my feeling is, I think – I don’t know, it’s a very good question because I want to give an answer, and I’m never quite sure what the – I want to believe that we should have a mix, and I think it works well.

But I think with dementia, struggling anyway with your day to day things, I think it might be an issue for people with dementia, if I am a white British, and I went to a meeting and everyone was Caribbean, and I was the only – I think that person might ... It could be a barrier, but I don’t think we should be separating, so it’s a kind of – I’m not sure what to do. I don’t think we should be separating things, either. I think we should be mixing in general, because I think there’s pros and cons to having both cultures, but I think probably mixing is still the best.

Interviewer:	Yes. But I do take your point on board, that especially maybe the older generations, they do – used to live in a world where it was more culture specific, or culturally isolated from each other, so they might feel a bit more uncomfortable in certain situations. I think that’s a great point. I was wondering, since we’re talking about different cultures, do you ever have patients who do not speak English as the first language, and if so, what do you do? How do you support them?

Respondent:	We use [Silent Sounds? 00:22:24] translator. I really don’t like it, I have to admit. I don’t think – social prescribing through a translator is really challenging, even if [crosstalk] can be as helpful and knowledgeable as they want, but I think we very quickly – I don’t find it – I struggle to use a translator with social prescribing, because again, it’s like I really want to talk to the person, I don’t really want to talk via the translator. It kind of loses things, but we do, I would say a good ten percent of my patients do need sometimes translators, and I don’t always encourage it. What I do encourage, if they’re going to come, I’ll say to them, “Please come with a family member and we can have a chat,” whereas I find the translating not really benefitting me or the patient.

Interviewer:	What is the difference, you think, in terms of advantage of having a family person versus a translator?

Respondent:	A family person has their issues, too, because obviously they have their ongoing, whatever issues there are with the person, and it could be positive or it could be negative, the translator is good because they can say things, and they don’t have the family, so they’ve got an opportunity to just say things. But I don’t think that always happens, even if it is a translator. I’m not keen on translating. I use the system, but I find it very confusing for me and the person, and the patients. And if it’s a confusing multi – if it’s a housing benefits, emotional, mental health case, I just find myself getting a little bit lost because of the translating. You spend more time –

Interviewer:	I just wanted to clarify, is that a person translator, or is it a system, an IT system?

Respondent:	It’s [crosstalk], so it’s a company that offers Arabic or whatever language they want, translators on the phone. So, they come on to – you have a joint phone call together with –

Interviewer:	So it’s a person. It’s a person, basically.

Respondent:	Oh, yes, it’s a person. Yes, sorry, sorry.

Interviewer:	OK, no, I understand that. So yes, you were just saying that you prefer to have a family member, that makes things easier. OK. Can I ask now about the final part of your support to the person? Do you ever encounter any issues with dependency, people getting anxious that you’re going to be terminating?

Respondent:	Yes.

Interviewer:	How do you work that out?

Respondent:	I mean, generally as a rule, we’ve always said that we’d generally try not to terminate, we try and keep the – if I’m supporting a patient, there’s no real reason – I don’t have to speak to them every week, so I can speak to them even once a month. And I say to people, “You have my mobile number. You have my email address,” I generally don’t discharge. Or, I’ll say to them, “If you want to call me, call me,” because I think also they have – sometimes they might say to me, “Is it OK that I’ll call you when I need you?” I think that doesn’t always work because I think they get so caught up with their day to day lives, they don’t always get a chance. But, I think they don’t like being discharged, no. I think they’d like to have ongoing support, and we’re quite happy to continue. I don’t have that many patients that need ongoing support, so I do speak to people quite a long period of time.

Interviewer:	And, I suppose sometimes it’s just having that reassurance that they can call you if they need to, that actually –

Respondent:	Yes. Yes.

Interviewer:	And, maybe because of that, they actually don’t call, because they know you’re in the background anyway.

Respondent:	Yes, definitely. Definitely.

Interviewer:	OK, that’s really interesting. [anonymised], actually, I think we’ve explored a bit the whole pathway, so from beginning to end.

Respondent:	OK.

Interviewer:	So, I was wondering, do you have any final points that you think might be helpful to know?

Respondent:	I definitely feel that I’m lacking in knowledge for dementia, and I’d like to learn more. I think whatever we need to do, we do need to have – I think any kind of training on dementia is welcome. I’ve done very limited training. I do have some very good communications and meetings and chats with people from the Alzheimer’s Society, they’re very quick to answer the phone and give answers, but I think there’s definitely a lack of who to go to. I’ve never had any NHS training for dementia, which I think is a bit disappointing. I’ve asked for it constantly, but nothing’s ever materialised so –

Interviewer:	Well, actually, you don’t get any mandatory training at the moment around dementia.

Respondent:	And even voluntary, I wouldn’t mind if it wasn’t mandatory, we don’t get any. Generally, training with social prescribing is very hit and miss, and every service offers different services, some signpost, some offer more. So, everyone’s very different. There’s not one size fits all, but I think an NHS-based training session would be really, really welcome, for obviously people once they’d got dementia experience, I think would be really welcome for my side.

Interviewer:	Where do you currently get your training from in [anonymised]?

Respondent:	Again, it’s very hit and miss. We get, generally things come, sometimes we get things, we don’t always – there is a social prescribing newsletter that comes out. Sometimes we join training. Training is very hit and miss. I don’t think it’s as well as it’s – you’d think we would have better training in the NHS, but we don’t. We don’t seem to have. I don’t think we have enough structured training.

We’ll get a mental health training, and there’ll be talk about dementia for half an hour, which is fine, but very rarely do we get a dementia training session or something that’s dementia-based, which I think is a shame. And the dementia café, the memory café, I actually was the one that reached out to them. They invited me to their activities. They were happy that I’d come, but they were quite surprised I’d come, as well, so I think there’s a bit of a – it works both ways. I don’t know. It’s a shame. I think more training would be welcome.

Interviewer:	Yes. From what I’ve heard, it seems to be more reactive than proactive, in terms of the training.

Respondent:	Yes, [crosstalk]. I think proactive is a missing –

Interviewer:	Yes.

Respondent:	It’s always reactive.

Interviewer:	It’s a missing concept.

Respondent:	Yes. No, no, there’s no proactive, it’s always reactive, which is a shame. I think it’d be nice if it was proactive.

Interviewer:	OK. That’s very helpful, [anonymised].

Respondent:	I’d be very happy to join any kind of discussion groups. I feel that I want to know more, and I know that it’s a big part of my caseload, so I’d like to learn more. I’d like to meet other colleagues. I’d like to meet other professionals. I’d like to be much more involved. I don’t know, obviously, it’s not from your point in this, in this obviously newer part, but it’s something that I’m very keen to link up. I find that when, a lot of times, reaching out to the organisations, very rarely getting responses. I’ll get some very odd responses, asking me to pay for the training, which I think is a bit odd. I think some of these things really –

Interviewer:	Yes.

Respondent:	So, I think there’s definitely a big gap in the market for dementia training, support, discussion groups. And I think that, why am I going on about it a lot? I know that if a patient calls me and says to me, “[anonymised], I’ve got an issue, X, Y and Z,” I can call the Alzheimer’s Society, I can go back to the GP [unintelligible 00:05:26], I can try asking the nurse, there’s definitely a big lack of somebody that we could be able to contact, obviously not just you or not just a website, but I think there’s definitely a need for some sort of dementia hub of some sort that would be able to actually support people.

And, bearing in mind that most people don’t want to come out of the house with the dementia. They’re very housebound. A lot of the activities are – the dementia café is not for people that are housebound, so there’s a big – I think there’s a big gap in the market for more home-based, telephone-based discussions for people. I think that’s definitely missing.

Interviewer:	What I can do from my perspective, based on what you just said, is remember at the beginning of the session, I talked about these group workshops, right, that we’re going to be doing?

Respondent:	Yes.

Interviewer:	Yes. If you’re quite keen, and you seem to be, what I can do is, I’ll invite you to those because that will be the place where you’ll be able to link up with people from all over the country to discuss about these issues. And also, that will be people with lived experience of dementia, and caregivers. It’s going to be a large group, very interesting, I think, based on your interest.

Respondent:	Sounds excellent. I’m also very aware that you reached out to me, I mean, I found that a lot, there’s a general issue, I think, with people – I spend my whole live sending emails to people, trying to reach out, not always getting responses, so I was quite excited when I got your email, because I thought, “At last.” It’s happened to me twice in the last year, that two universities have contacted me about social prescribing. I think it’s great. I think it’s really – it’s something definitely you can pat yourself on the back, or you’ll be able to say that it’s really something that social prescribers, I like doing. I think it’s a great – it’s really good that you reached out to me.

Interviewer:	Thank you, [anonymised].. I really appreciate the positive feedback. I really appreciate it. What I’ll do is, I’ll keep you on my radar for when we’re organising the workshops, so you’ll be invited. I’ll make contact with you via email, and I’ll let you know a bit more when we’re getting closer to that, yes?

Respondent:	Sounds great, but it’s been really interesting talking to you. I’ve enjoyed it.

Interviewer:	You too. You too. Thank you so much. So, I’ll let you go, and I’ll speak soon, OK?

Respondent:	Take care. Thanks again, [anonymised]. Bye bye.

Interviewer:	Take care. Bye bye. Take care.

[End of recorded material 00:07:30]

**SP22**

[Start of recorded material at 00:00:00]

Interviewer:	Do you want to give me a bit of a background about what you guys do, what’s your specific role in working and supporting people living with dementia?

Respondent:	Sure. So my personal background is that I’m a community artist, so I’ve been working with people for over 12 years on participatory arts projects, working with different communities. And that has led me to working with people of all ages, but often I’ve done a lot of residencies in care centres, care homes, and through that work, that has meant that I have worked with quite a few people with dementia. And so then, that has led me into the role that I’ve now been in at [anonymised] for a year. So I’m [anonymised], so my job, basically, I manage a programme of creative sessions and live events and creative workshops, particularly catered – which are dementia inclusive.

So, the criteria of our programme, so we have basically creative socials, which are arts workshops in the afternoon that run in locations across [anonymised] every month, and then we have a programme of live events, of live theatre, live performances, screening, singing, all of that, and that runs every other month, and we call those afternoon events.

So, [anonymised], so we’re a small arts and community development charity based in [anonymised], and we’ve been working with people living with dementia for long before my time, like over ten, 12 years, so we’ve got a real background and a lot of specialist expertise within the programme. Sorry, within in the team, and obviously within the programme. So, the work that we do now really comes on a very strong foundation of what’s come before, in terms of working with people with dementia.

And then, yes, just to say a bit more specifically, so my work as [anonymised], as well as managing the programme, I’m also looking at trying to build us much more solid links with social prescribers and healthcare connections. I guess it’s a given for me, but obviously there’s so much research to the benefit of creative engagement for dementia, even specifically singing, music-making, dancing, movement, but also those practical skills as well. And we know the benefit of it, but often, there’s a lot of this provision out there, and it can be really difficult to get it in front of social prescribers, to let alone build the relationships and the trust that’s needed.

So, my role was brought in specifically to do that bridging with social prescriber link workers, and the art programme that we’re running. So, I also, within my role, I also sit at a hot desk as a local council here called [anonymised], and that is where the area’s social prescribers sit. And so, my role has been working with – looking at, yes, social prescribers, but from a slightly different angle, but doing that on the coalface engagement with people with dementia. So, I hope I’m able to offer you maybe almost like an in-between person, between the social prescriber, and the people working with those living with dementia, if that makes sense.

Interviewer:	I think, no, it’s very helpful actually, because you’re right in saying that – well, within this study, I’ve mostly spoken to primary care networks, people, social prescribers, but actually I think we just had another participant who’s working from the side of those organisations, actually providing the activities in the community, and it will be interesting to explore with you how you are finding it potentially difficult or easy. You’ll be able to tell me.

Respondent:	Sure.

Interviewer:	In terms of liaising with the social prescribers.

Respondent:	Yes, yes, absolutely.

Interviewer:	Because, yes, from what I’ve heard from the social prescribers, I’m not sure – I take what they say at face value, so I take it for granted that it’s the truth.

Respondent:	Of course.

Interviewer:	But it seems that what they try to do in terms of primary care services is to engage in some sort of outreach work in the community. So, lots of people have told me, “We tend to go to community events and introduce ourselves, so that we can present what we do, what social prescribing is, and make those links in the community, to let people know that we exist, but also to potentially create those liaisons with the actual organisations in the community.” So, that is from that side, but I want to ask you, what is from the other side of the coin, the reception that you seem to have from primary care services and that social prescribing workforce? What’s your experience in terms of that?

Respondent:	Yes, it’s really mixed, and it’s difficult because what I’ve learnt is, so much of it is really reliant upon personal connections, personal relationships, knowing that person. And that’s why they developed this role specifically, just because it needs one person completely dedicated to forming those relationships. And so, it has taken me a while, and I have begun to build that up, but it has been really difficult, and I’ve found that coming from the arts perspective, yes, it’s not just that – I’ve done a lot of work to form those personal connections and ensure that I’m informing those social prescribers as regularly as possible about what we’re doing.

But, I also find that a lot of it is dependent on the person, and what’s their personal connection. Like, I guess – what am I trying to say? I guess they’re biased, in a way, it’s like do they believe in the arts as a justifiable, important offer for that person on their journey? And so, it’s really reliant upon, yes, the social prescriber as a person. What I’m trying to do now to better that is not only trying to inform them regularly and get that information in front of them, but I’m also trying to do a lot of work of personally inviting them to come to our sessions, because I think that that is going to obviously be a lot easier if they personally experience it and see firsthand, and then they’re able to speak about it. So, I think I’m really hyperaware of gaining their trust, because I can completely understand that they only want to signpost someone to something that is going to be appropriate, it’s going to be accessible, that’s going to be high quality, and that’s going to be suitable, and so it’s really important that I do that trust building, I think, with them.

Interviewer:	Yes, no, just to chip in, it’s interesting what you’re saying, because I completely agree that, actually, in some respects, social prescribers are gatekeepers, aren’t they?

Respondent:	Exactly. Well said, yes.

Interviewer:	Because they are, yes, the bridge between the person and you guys, and if they, one, don’t know about the activities, but two, maybe even more importantly, if they don’t believe in the effectiveness of what you’re proposing, then they are kind of gatekeepers to the actual patient. That’s really interesting, and it’s something that you say comes from speaking to somebody who’s on the other side, because it’s new insight, so that’s great. So, you do feel that it’s – do you feel there is resistance, there is lack of knowledge about the benefits of arts in dementia?

Respondent:	I can’t say I’ve come across that. What I come across is lack of capacity. I feel like everyone is – there just doesn’t feel like there’s enough – I mean, it’s the same in the arts, but it just doesn’t feel like there’s enough social prescribers, and they’re all at capacity with their caseloads. And I’ve been able, I think, to get maybe one or two social prescribers to actually come to a session, but I’m just so hyper aware of how high their caseloads are, because like I say, I do this – I’m able to sit with them and hear about the wider picture, which is a very interesting angle.

And so I’m aware of – and it's the same with the arts, often people are working part time, and it’s limited capacity. So I think that’s where I find it, it’s just, yes, keeping that sort of – shouting at them that, “We’re here. We’re doing this,” and reminding them, because they’ve got so much going on. They’re got all these other services.

So, I find that something else that helps is when there’s more collaborative approaches. So, I don’t know if you’ve come across this, but there’s voluntary care sector collaborations called VCSEs in different areas, and I tap into a couple of them that cross [anonymised], and I find that really helpful to sit at the table of other service providers, with social prescribers. And so then, sometimes on those collaborations, there might be caseloads that are discussed, and you’re able to offer, “This is a service we might be able to help an individual.”

So I think I’m now finding that if we can position ourselves not just as an arts organisation, but as just like one of the other service providers, like I’m in the middle of trying to set us up on a referral system, for example, for [anonymised], and I think if we position ourselves in that way, I’m just trying to make the workflow of a social prescriber easier all the time, to get to us. But it’s a strange dance to try and position yourself in a completely different landscape, which is this healthcare landscape. Yes, it’s so complicated.

Interviewer:	Yes, and sometimes it’s a difficulty to kind of almost assert yourself when there is such a medicalised approach to dementia within healthcare. And, you’re right in saying that it’s such a different culture, especially I suppose, coming from the arts, with that social model of disability and dementia as well. So, it’s difficult to step into that world and make yourself known and accepted as a viable and effective way of supporting people with dementia.

Respondent:	Yes. Yes.

Interviewer:	I completely agree with that. What you said before about workload, absolutely a point that’s been made over and over, to the point, actually, that lots of social prescribers currently support people living with dementia remotely, so that’s a big barrier, because obviously, you’ve got somebody with cognitive impairment, and if support, starting from day one, only occurs via the phone, obviously you’ll have resistance, lack of engagement, motivation, and all of that. So this systemic issue is affecting also rapport between the actual social prescriber and the patient, let alone when it comes to coming to your events. I can only understand how frustrating that might be, in a way. What are some of the ways in which, within this resource, we can support social prescriber to better engage with you in the light of how overloaded they are? Do you find something has proven particularly effective with some of the social prescribers?

Respondent:	I think, to be honest with you, I’ve just thought of something on the previous question, and my head’s dipped back there, but I will come back to –

Interviewer:	Yes, yes, yes, no, go ahead. Yes, yes, yes.

Respondent:	I’m thinking of something else that is a barrier, is that social prescribers sit under so many different umbrellas. They’re not all coming centrally through the NHS, and so because it’s so complex, and the areas in which they sit under don’t sit under the healthcare or the county lines – I don’t know if that’s across the country, but certainly in [anonymised] –

Interviewer:	It is, yes. It is. It is.

Respondent:	That’s another thing that can make it really impenetrable, is how complicated that is. And, because our funding as an arts organisation, and the [anonymised] is, fortunately, we are able to access funding that way, but it would make much more – it would make a lot more sense if we were able to secure funding from healthcare systems, because that’s what this work is. But, I mean, that’s a bigger conversation about people higher up, people allocating money within the NHS, funding the arts, and seeing the justification in that. And perhaps that’s a different – a whole conversation for another time.

But, I think it’s that you’re just aware all the time that there’s this central healthcare systems, we’re trying to penetrate it, and then there’s some social prescribers sit under it, some are local councils. The landscape’s just so complex, and even more complex than I thought it was going to be a year ago, when I started this work. But, yes, just to put that there.

Interviewer:	Yes. Before we touch on the other question, actually, I wanted to ask you, sorry, before I forgot, you mentioned a self-referral system that you’re trying to implement.

Respondent:	Yes.

Interviewer:	Now, would that be the case in which a person can directly self-refer to one of your activities, skipping the whole primary care route, and directly liaising with you?

Respondent:	Yes. So, what I’m trying to do is have as many options as possible. So, at the moment, we kind of, I think we informally take referrals which, we’re either passed on somebody, or somebody contacts us, or our session – I mean, our sessions are also completely drop-in as well. But, like I say, to evolve this further, I want to position ourselves in that referral sort of workflow. So, I’m on the cusp of setting up that we’ll sit under – I think it's called NCAN here, which is – oh, there’s so many acronyms.

Interviewer:	Oh, don’t tell me.

Respondent:	But yes, anyway. Anyway, it’s like the [anonymised] referral, voluntary community healthcare organisation referral system. And so, I think we are about to sit under that so that social prescribers or other organisations could refer in. But, I’m also keen that we have a digital referral form for somebody, for example, who is a social prescriber, they just wanted to fill it in on our website landing page, or perhaps a care – often we work a lot with carers, and that’s a whole thing in itself. But, perhaps a carer could refer someone. But yes, I think it’s really important that there’s also a place where it’s an easy, accessible – most important word, “Accessible,” and simple form, that someone could self-refer as well. So, I think for us, it’s about having as many doors open to try and get people into our service.

Interviewer:	And it’s been proven quite effective in some other areas that have implemented this, and in fact, if you’re ever interested in talking to someone who’s been on this route, I can link you up with good people. I think one was in Northumberland, or something.

Respondent:	Oh, yes, please.

Interviewer:	But, yes, I will certainly do that. I’m always keen on liaising with people. But, what was interesting, they were saying that this also kind of facilitates access to activities, to communities who might be a bit hesitant to liaise with primary care services, and going through that route.

Respondent:	Uh hmm.

Interviewer:	So, for example, communities like ethnic minorities or LGBTQIA+ communities, they’re finding that the direct route to services kind of balance out a bit, that again, that kind of hesitancy that communities might experience because of historical stigma and whatever. So it’s a model that’s working because it’s an alternative.

Respondent:	That’s good to hear.

Interviewer:	Because as you say, it’s giving options to people to maximise, yes, uptake.

Respondent:	Yes, yes. Yes.

Interviewer:	So, OK, so that’s interesting. Yes, going back to the specifics of people living with dementia, what we’re trying to do, again, is to come up with some very practical things that people have found helpful when involving people living with dementia. So you have experience, obviously, holding these arts initiatives and events, just an opening question, maybe let’s start looking first at initial involvement, how do you get the person to get out of their home and come to the place and engage? Because, I suppose there’s going to be barriers. Have you found anything in particular helpful, or some specific barriers that it’s important to address?

Respondent:	Yes, so I think the main barrier that we face here is because our work is all in rural areas in [anonymised]. I should’ve said that at the beginning. As opposed to the sort of [anonymised], that core city. And so, because we’re working predominantly in rural areas, transport is our biggest problem. That’s the thing we hit against, and I’m sure that’s probably reflected in wider social prescribe – as a barrier. I think in terms of – yes, it’s first of all ensuring that we are putting the messaging about our sessions perhaps in, yes, in those places where somebody who is even starting out on their journey with dementia, or their loved one, that they come across the information. So whether that is maybe – I always try and ensure that we’re listed on local directories of resources, or whether that’s come through from a social prescriber, ensuring they’ve come across that information. And then I think it’s really important that we support them in bridging, and bringing into that session with that really personable – that connection with us. We make sure, though we’re a small team, we ensure that we’ve had a conversation ahead of time with them, to let them know what’s to be expected, what’s the venue going to be like, what does the session have planned, the artist coming in, what’s that going to be like. And so, to make them feel as comfortable and rest assured as possible, and give them the information.

Us, as a team, we are all – we all have had dementia training, so we know very clearly, I think that’s super important, but as important, the artist we work with, we ensure that they either have had that dementia training, or they have experience working with people living with dementia, because it’s a – and I say that as one of those artists, but also with my role, that it’s a very specific, nuanced skill to be able to hold space for lots of different people living with dementia in very different ways, and to create warm, safe, welcoming environments that are flexible and adaptable to who walks in the room, and how they might be feeling that day.

So, yes, it’s the getting them there, and like I say, transport can be a big part of that, but it’s also the trust-building, and knowing that a friendly, warm face is going to be there, that they might recognise already, or that they’ve spoken to on the phone, and that we’ve helped that, bridging that where we can. And then, coming in the session, we’re facilitating – I wish I could bring you to a session to show you, but it’s a really special place because it’s a balance between that very relaxed, creative activity, and as much as it is the social time, that time, and sometimes for, I think, one participant, she’s living with dementia, and her husband leaves her, and that’s his only respite he gets each week, is those two hours that she’s within us, essentially, within our care.

And so, also within the session structure, there’s an engagement time within an activity, but there’s also quite a big chunk of time to have tea, cake, refreshments, and the social aspect of it. Yes, so that’s – I’m just trying to think –

Interviewer:	Yes. No, I suppose one great point that you brought up is that you’ve got in place strategies to make sure that the person understands that it’s accessible, it’s relevant to them, it’s safe, and I suppose, and correct me if I’m wrong, but in order for people to attend, it’s also kind of important to convey this message to the caregiver. So, I wonder – because obviously, they facilitate the whole process. So, I wonder whether – you touched on respite, sometimes it’s an opportunity for caregivers to have some time for themselves, or some quality time with the person, if they’re together in the session. I suppose, do you have specific ways in which you can sell the message to the caregiver that this is also helpful for them? Do you know what I mean? Not just for the person, but we’re kind of doing this for you as well.

Respondent:	Yes. Yes, it’s a really interesting question, because it’s so specific on the person, because we also have a lot of – we can also use that very clear language around stating very clearly that it’s been designed specifically for people with degenerative diseases, but not everybody associates with that completely at all, or positively, and sometimes we don’t always shout about the dementia-inclusivity of our sessions. I think looking at it completely, we also, it is technically for over 50s. I think the next layer to the criteria is, we have that people are either lonely or socially isolated, and then if you come down a bit further in that criteria list, it’s dementia-inclusive, but also inclusive for other degenerative diseases.

And so, what I would say is – and much like our work, it’s person-centred. So, I would probably do my – I would be doing my reading on the person, in that conversation we were having initially, to get a sense of that. And I would be trying to meet them where they are. I think that’s what I would do. I would tune myself, probably, to that person, and get a sense of, do we need to be having that conversation about, yes, dementia inclusion and appropriateness, and what do you need, and what does the person you’re with need?

Or, they don’t have to tell us if they have a degenerative disease, they don’t have to declare that to us. This is just an open, safe space, but we just happen to have another almost dimension to what we’re doing which is, we have that inclusivity, that net underneath to catchment [unintelligible 00:24:01] for everybody. But not everybody wants to have that, have that conversation, which of course, people might be coming to terms very, perhaps, recently, that they’re beginning their dementia journey, and I’m always very sensitive to that.

Interviewer:	Yes, I take this point. I’ve heard about lots of people who are actually living with dementia, who actually refuse to attend any session or initiative that is dementia exclusive, as in only people living with dementia are going to be there, because this is the future for me, and I don’t want to face it right now. I don’t want to see where I’m heading.

Respondent:	Yes, or I’m more than just the disease, I’m more than just my diagnosis. Yes.

Interviewer:	Yes, exactly, and that’s the truth. It’s about the person. So it’s a person-centred kind of discussion to be held, I suppose actually, between the social prescriber and the patient, at the moment where they develop that rapport and trust, so that they can actually find in the community an activity that is maybe not just dementia specific but dementia inclusive, and so on, and so forth, or maybe that it is dementia specific. Yes, I take that point. That’s quite important. Do you find, [anonymised], that within your sessions, you got fairly balance diversity in terms of people that attend, talking about age, diagnosis, ethnicity?

Respondent:	No. Well, yes, I mean, yes ... Well, I said no to diversity initially because I was going to say that what is on reflection of rural [anonymised], which is, it’s a predominantly –

Interviewer:	Geographical – yes.

Respondent:	Yes, geographical, predominantly white, aging population, however that is not to say that I think we could do more work all the time to try and connect to ethnic communities who are facing even more barriers to this, like you mentioned earlier, in particular, yes, wanting to enter into those healthcare systems, or yes, it’s how intimidating this could be if you don’t have that connection into it, or – because, we do our sessions in different community spaces, you might not even enter that church hall or community hall or library anyway, so there’s so many barriers in place. So yes, so I would day that there’s not, in that sense, not great diversity.

Interviewer:	Yes. It’s reflective of your locality, and I understand that, you can’t have this diversity you have in London or another more metropolitan area.

Respondent:	Yes, yes, yes, yes.

Interviewer:	I wonder whether, in terms of what you can do, because you said, “Potentially we could do something,” I was interested in hearing what you think could be done to address this potentially, looking ahead.

Respondent:	Yes, for sure. Yes, I mean, so something that we’re doing is ensuring that our programming is reflecting diversity. So, the events and the artists that we are programming, we’re having a range of different artists and performers coming from different backgrounds, telling different stories, and I’m finding that that is – that feels an important thing, because what we’re doing is, we are offering different stories and different perspectives to these rural communities. And it’s beginning slowly to, I think, draw in different communities who might not be accessing what we were doing before, but just as importantly, it’s storytelling to the participants we have already, showing them different windows to the world.

So that’s kind of our approach at the moment, but then it’s also – but then I think it goes down to, yes, trust building and connections, and doing that work in those different communities to signpost, and say, “We’re doing this.” And that takes time. But, what we’re doing at the moment is trying to ensure that our programme has that representation, so that’s what we’re bringing to communities, and we hope, in turn, and with more supported work, that can be reflected in our audience coming to those events as well.

Interviewer:	Yes, because –

Respondent:	But yes, there’s loads of work to be done.

Interviewer:	And then there’s diversity in so many different terms, so there’s diversity in tastes and preferences as well, so it makes sense that you’re delivering a variety of activities that responds to, again, that person-centredness that dementia care is all about.

Respondent:	Yes.

Interviewer:	And I was wondering whether, in terms of – because you mentioned doing some of these events in community halls or community centres and churches, maybe there’s potential for you guys to go down the route of potentially temples, or those congregation centres for specific communities, which I think lend themselves well to arts as well.

Respondent:	Yes, yes.

Interviewer:	I’m sure, as you say, it’s a work in progress.

Respondent:	Yes. But, to come back to – I appreciate we tangent there, but to come back to your original question, in terms of diversity of age, yes, so that can be – and also, I guess, dementia as well. So, yes, I would say the vast majority of our participants, certainly I don’t think they do, and they don’t present as if they have dementia, so I would say that we have pockets of people living with dementia in our groups. We do have quite, I would say, quite a range of ages, and we do have that data. I was just thinking, actually, after we set this up, is that we have got a researcher called [anonymised], who works at [anonymised] at [anonymised], and I wondered about mentioning this to you perhaps for you two to link up, or to connect with our work.

So, what she’s doing, she’s currently evaluating all of the data from this, our [anonymised], as well as our – so, within [anonymised], we also do rural touring of live events as well, and she’s crunching that as well, so just to – you were saying about linking up, and I think that maybe there’s something. And she’s written fantastic reports for us before, and at the end –

Interviewer:	That might be helpful. That might be helpful for me to also have a flavour of what the current status around anything regarding social prescribing in dementia is, because part of this research that we’re doing is also to actually identify what the current status is out there, and maybe [anonymised]’s work can give me a background of what you guys have been doing, and supporting that with some data. So, it would be great, yes, if you can make the link up, we can –

Respondent:	I’ll pull that together for you. And I know I sent you our general website with what we’re doing, but I’ll also send you a report, our big report from this work. So previously, we were funded by, I think it was Spirit 2012, so that was between 2017 and 2019, and as part of that, a big report was written, and it was – in fact, I made some notes. So, it was evaluated by [anonymised], and then they used the [anonymised], because I hear that’s dementia friendly, and then it assessed, basically, people’s well-being before and after, over three years. So, within that report, we have all of that hard data about the improvements. And, there was considerable improvements and shifts, but I just wonder about – yes, what I’ll do is, I’ll do some signposting so that you’ve got some of those kind of dates and figures for those reports.

Interviewer:	Yes, yes, and actually, what you have in that report is very important, because the current status of evidence on social prescribing out there is a bit poor, but not because it’s missing, because you have an example of a report that shows that there’s improvement, but because it’s not necessarily labelled as social prescribing, so it’s difficult to find that evidence. But it's important to actually share, share it, because it will create that evidence base and support that shows that it’s important, and it’s valuable and effective. So, yes, do –

Respondent:	Yes. I’ll pull something for you after this, this afternoon, and certainly send you something through.

Interviewer:	That’s very helpful. That’s really helpful. So, going back to the initial point that we were making around –

Respondent:	Sorry, I feel like, you and me, we could talk about this for hours, there’s so much to be said, isn’t there?

Interviewer:	Yes, I know. It ends up not being an interview, but a conversation, which is much more interesting, isn’t it?

Respondent:	Yes.

Interviewer:	I always end up doing that. But, I’m particularly interested, based on your own perspective, because it’s different, as we said at the beginning of it, it’s looking from that specific side. I was wondering how to translate this into a resource. I wanted to create, within this resource, a section that facilitate relationship between you guys, people in the community who are actually providing these activities –

Respondent:	Service providers, I guess.

Interviewer:	Yes, and the social prescribers, so I’m wondering, in terms of translating it into almost a to-do list, what you can do as a social prescriber to actually liaise with groups like yours in the community, I need to come up with some sort of hints, have you tried this, have you tried this, and that’s what I would like to almost discuss. I think it’s very helpful if we discuss it with you today, because I’m not sure we’ll have another chance to have a one-on-one, because the next stage of the research is going to be group workshops, and in that context, I’m afraid there’s not going to be that room to actually delve deep into this.

So, it might be really helpful, actually, for me to come up with some tips, so one thing we discussed is that social prescribers are sometimes unconvinced about the effectiveness of the arts in dementia, and I suppose one way of actually translating that into an action point for them is maybe go into the community, to actually sessions, as you said, go and experience it for yourself. That could be one. The other one is, also look for the evidence, because when it comes to arts and dementia, there is lots of evidence out there that’s been published. I don’t know if you know [anonymised], but that’s just an example of a person that’s published a lot on arts and dementia, so maybe look for the evidence, because that will give you backup data. Can we think of any other points that might be important for them to actually understand, its effective?

Respondent:	Yes, it’s such an interesting book, because I’m in parallel to this, I’m in the middle of trying to – so, something that I saw done really well is, the [anonymised] do a lot of work within this field, and they have the [anonymised] is based there. Yes, so I’ve been to some events held by the [anonymised], who are doing great work in bridging the gap between arts organisation and social prescribers. And, while I was there, I was picking up some resources from the [anonymised], and I’ll see if I can maybe send you a link when I do this email for you.

They’ve got this really lovely resource for social prescribers that shows the – that explains what their sessions are but in an easy, digestible way, but also shows the evidence and the importance of that work in this really great, easy, few-page document. And, that’s what I’m trying to design at the moment, to do that as well, to house that information, like you’re saying, for a social prescriber to see basically, quickly, right, this is legitimate. The evidence is there, they’ve got the experience, and that’s right. So, just to say, I’m in a parallel position to you about trying to think about what a resource to enable that looks like.

Interviewer:	Yes, and I can say you’re on the right track, because what I’ve heard from 20 or more social prescribers I’ve interviewed so far is, we need something that’s really practical and concise, because as you observed, they haven’t got time. They don’t necessarily receive mandatory training, so it’s out of their working hours, to actually look at this stuff, so absolutely. That’s why I said, “Give me some tips.” I know it sounds very dry, or short, but that’s what they seem to want, something quick and accessible.

Respondent:	Yes. Yes.

Interviewer:	So, yes, absolutely with you on this point. You mentioned the [anonymised].

Respondent:	Yes. Yes, so like I say, yes, they’re great resources, so yes, we have links with them. So, they have been running a programme called [anonymised], which has been really important for us, but I’ve got a feeling the funding has just changed, or it’s coming to an end. And, they have been a really valuable connective tool for us, us as an arts organisation, and then with their links to social prescribers.

So, for example, [anonymised] has been doing a monthly email. I don’t know what’s going to happen now that the funding’s changed, but say to date, they’ve been doing a monthly newsletter, and I email all the stuff that we’re doing to them, and then they send it out to all of their links across healthcare community and the arts. So, maybe that’s a practical tip, get yourself signed up for newsletters across all of these different fields.

But yes, I think maybe it’s probably even a bullet point in itself to even consider the arts, because maybe that’s not something that they’ve even – if that doesn’t come into your background or your experience or your day to day prescribing as such, maybe you don’t think about the arts. So just, yes, making sure there’s a clear point to say what else is happening on a wider level, but can you –

Interviewer:	That’s a good point, look outside the box, don’t get fixated in what you’ve done previously in your experience and background. There’s stuff out there that you may not be aware of. And actually, the other point is a good one, get signed up to newsletters from these large national social prescribers academies or associations, because it seems, from what you said, that they kind of collect the various opportunities, and they send them out.

Respondent:	Yes, yes, yes. I feel like I was going to say something else. Yes, I think, yes, getting signed up to newsletters across different, yes, different types of community offerings. So, we have, something else is, we’ve got a lot of voluntary directory resources, sort of in [anonymised], like I mentioned that I ensure that we’re up to date on. So, for example, there’s a website that I think is called [anonymised], and they have a directory listings, and I always ensure that we’re up to date with our events. So, I think just being tapped in across the board on these directories and newsletters. I’m just trying to think

Interviewer:	Yes. The local ones. The local ones –

Respondent:	Yes, local ones, and the national, obviously, as well. Yes, I’m just trying to think. It’s like zoomed-out thinking, I’m trying to think beyond sort of just us at the coalface, trying to think on a wider level. Yes, I think it’s the encouraging of making those links, yes, across potentially different types of offerings that you wouldn’t – yes, feeling brave enough to go out and try things, but yes, I think, I mean, I can’t speak to what their capacity is, but if there is capacity to go out and experience things as an individual first, that feels really important. I’ve heard social prescribers say to me, they love receiving things, they love getting mailings, they love being able – invited to things. But, a lot of it, from my end, can feel like you’re shouting into the void, and you never know who’s – you never – yes.

Interviewer:	You cast some nets and you hope that you’ll catch fishes, but then it not always is the case.

Respondent:	Yes. Yes.

Interviewer:	One point that you touched upon, I think it’s also translating into practical advice could be, you know if a client comes to you and they’re from a specific community, some activities may not necessarily be available locally, and I’m thinking rural areas. It’s good, as you say, to actually, for a social prescriber to be aware of what’s potentially happening beyond their locality.

Respondent:	Yes.

Interviewer:	Say for example, they can signpost some people to remote sessions of something that’s culture relevant, that’s in another area.

Respondent:	Yes, or something –

Interviewer:	So that’s part of that, yes.

Respondent:	Yes, or something like the [anonymised], they do the [anonymised], so that’s sending out stuff via post. Yes, exactly. I think it is, it’s that looking more holistically at what’s on offer across this landscape, and I think, encouraging to make connections with organisations who are doing this work, and saying – getting in touch with people like me to build those links, because there’s – I think sometimes about the problem that we have with people being in rural areas, and they can’t always access our sessions, sometimes we have funding that we’re able to support in individual cases, say transport to a session for an individual for whatever reason that isn’t able to access it. But on a general point, that’s not something we can offer to just – I’m losing my voice again. Sorry, excuse me.

Interviewer:	Sorry, I’m making you speak so much.

Respondent:	No, I feel really passionately about this so don’t worry, the coughing is worth it. But, sorry, that’s a very convoluted way of saying that, have that personal connections with the different organisations, don’t just take it on what those flyers or their website say, because while the website stated that the service could not offer transport, they could find ways and make budget lines work so that they could support an individual in exceptional circumstances. I only learned that by having an in-depth conversation with the people in the organisation. So, I think relationship, really encouraging relationship building.

Interviewer:	Yes. It echoes what a social prescriber told me, which is great. They were telling me sometimes the information that’s on the web of a specific organisation might not necessarily reflect what’s actually being provided at the specific moment in time. So, maybe there’s new stuff, or stuff that’s been cancelled, and the website might not have been necessarily updated. So, it’s always good to pick up the phone, have the conversation or email, but actually have a direct liaison with somebody from that organisation to check this stuff. Criteria might change for specific activities, and all that.

Respondent:	Yes. Yes, yes, yes.

Interviewer:	So, that’s a great one, as well.

Respondent:	Yes, because there’s probably more roles like mine are probably happening in arts organisations, though I sense that this is quite progressive work. But, there’s probably going to be – the way that arts as a whole is shifting in funding, reducing, I think we have to find our place in different – almost like a Venn diagram to find ourselves in different, wider fields of working.

But yes, it relies upon having that openness and curiosity and outward looking, to try and build those. The National Academy of Social Prescribers and the National Association of Link Workers have been doing monthly newsletters with information on initiatives and activities. So, it is good to sign up. Regional conferences are also a good way to actually get all in the same room and be able to network and make those connections. Because, like I say, it’s so down to that personal relationship. So, just encouraging them to try and attend as much of that as possible.

Interviewer:	Yes, that’s an important point that’s been duly noted. I think we came up with some good practical suggestions, so what I’ll do is, I’m in the stage of analysing all the interviews that I’ve done so far, and based on the specific person’s area, to create, translate those interviews into these practical points. And, I feel that, with you, we’ve explored quite in-depth what they can do to actually learn and accept, and embrace what you guys are offering.

Respondent:	Yes.

Interviewer:	So, that’s really helpful, [anonymised], thank you. One thing that I wanted to say before we finish – I don’t want to take too much of your time – is first, what’s next? Because, you said you might be interested in later stages. So, what’s next, essentially, is once I have this idea around – or, once I’ve created this checklist, I’m going to be organising the first workshop, which will include people that I’ve interviewed already, just like yourself, but also, quite interestingly, other stakeholders, so people living with dementia, and caregivers, so actually discuss, is this checklist OK? What’s missing? Is it helpful, and all that, and refine it. So, that’s the idea that we have for the workshop. If you’re interested in taking part, what I can do is, when the time is a bit closer, I can email you with some tentative dates in which we’re organising the groups, and invite you so that you can contribute.

Respondent:	Wonderful.

Interviewer:	But for now, I just thank you. I’ve taken lots of your time so –

Respondent:	No, no, no, my pleasure. No, don’t worry. Yes, it’s all good, and yes, hopefully, I’ve said something that’s sort of made sense. I’m really trying to get my head round all of this, too, and it’s so complex.

Interviewer:	But bouncing off ideas is always very helpful, and I feel like we both benefitted in some respects from this conversation, so that's great.

Respondent:	Yes, absolutely. Most definitely. And, I’ll pull together an email that’s got a bit more of our reports, so you can have something a little bit more – yes, the solid data, and I will –

Interviewer:	That will be very helpful.

Respondent:	Yes, and I’ll do that link with [anonymised], so that you’ve got that connection, because I was just thinking, even internally, perhaps, you might have a way of, yes, having that connection across [anonymised] because it just makes sense if we’re both talking to you both, it makes sense to maybe make that link.

Interviewer:	Yes. Yes, no, that’s very helpful. I’ll wait for that email, and I’ll make contact once we’re closer to the workshops, yes?

Respondent:	Yes. Wonderful.

Interviewer:	Awesome. Thank you so much for your time.

Respondent:	Ah, thanks for your time. I’ve enjoyed speaking.

Interviewer:	You too. Take care.

Respondent:	Take care. Bye.

Interviewer:	Bye.

[End of recorded material 00:50:18]

**SP23**

0:0:0.0 --> 0:0:10.200
Interviewer
Introductory want to know a bit more about you, so maybe what you do and in what capacity you support people living with dementia just to have a bit of a background info.

0:0:10.840 --> 0:0:17.760
Respondent
OK, so I've been working as a social prescriber now for about 15, 16 months.

0:0:18.80 --> 0:0:18.280
Interviewer
Mm hmm.

0:0:19.560 --> 0:0:34.280
Respondent
No training really around dementia. Lots of lots of online training initially, but it was really more around data protection, stuff like that, and then then suddenly I'm in post, I'm meeting with patients, talking to patients.

0:0:36.400 --> 0:0:36.840
Respondent
And.

0:0:38.360 --> 0:0:41.400
Respondent
Initially I think I didn't really meet anybody.

0:0:45.290 --> 0:0:45.810
Interviewer
Right.

0:0:41.850 --> 0:0:46.450
Respondent
Which was referred to me for any sort of support around dementia.

0:0:46.490 --> 0:0:53.130
Respondent
Then we had I work within a team in, so I'm I'm in. Right, right down in the bottom end of [anonymised].

0:0:57.910 --> 0:0:58.230
Interviewer
Yeah.

0:1:7.440 --> 0:1:7.720
Interviewer
Yeah.

0:0:55.130 --> 0:1:9.290
Respondent
In a place called [anonymised], that's our team and very lucky I've got about five or six very supportive colleagues who work within the [anonymised] team and we have a monthly meeting where we can talk about tricky cases.

0:1:10.530 --> 0:1:13.10
Respondent
Or whatever it is that we want to discuss. And usually we have.

0:1:14.530 --> 0:1:38.770
Respondent
A monthly speaker that comes in and just shares information. So around winter time last year, we invited somebody from the [anonymised] Alzheimer's Society to come and talk to us about how we would refer patients into their service for support. So that was about the first time I really had any opportunity to.

0:1:38.810 --> 0:1:46.890
Respondent
To ask questions around dementia and Alzheimer's, try get a bit of clarification about how we refer into their service.

0:1:47.410 --> 0:1:54.330
Respondent
And then then I started to meet with patients. I had one patient who had.

0:1:59.300 --> 0:1:59.700
Interviewer
Yeah.

0:1:55.770 --> 0:2:6.370
Respondent
He had been diagnosed early stages of Alzheimer's, but it wasn't really affecting him too much in his day-to-day life, his memory was a little poor.

0:2:21.20 --> 0:2:23.780
Interviewer
Oh yeah, yeah.

0:2:7.730 --> 0:2:27.130
Respondent
But he was able to come and meet with me independently in the surgery. The reason I'm saying about the memory being poor is I got a couple of phone calls saying your patient has arrived at the wrong surgery. He's come for his appointment, but he's come to the wrong place. So that's when it sort of dawned on me, actually, that.

0:2:41.490 --> 0:2:41.970
Interviewer
OK.

0:2:27.530 --> 0:2:49.50
Respondent
There's there's things like that that he was struggling with, but he was able to live independently at that time and still is as far as I know, because we only work with patients for about up to about 3 months, sometimes could be more than that, sometimes less, depending on what their needs are, usually averaging around 3:00 or 4 appointments.

0:2:50.410 --> 0:2:57.50
Respondent
Working on the question, what matters to them and for this chap, he was feeling very lonely. His wife had passed away.

0:2:58.410 --> 0:3:14.810
Respondent
And although he had family members, they didn't live in [anonymised], so it was about trying to find him a bit more of a social connection. And also he was interested in losing weight. He'd gained weight over the last couple of years and and that was really it wasn't really about supporting him with his dementia.

0:3:15.300 --> 0:3:16.140
Interviewer
Yeah. OK.

0:3:16.810 --> 0:3:28.930
Respondent
But it was the first time I'd met with a patient who I could see struggled a little bit with remembering appointments and things like that. So talked him through how to use apps on his phone.

0:3:29.530 --> 0:3:36.570
Respondent
Reminders text alerts, that sort of thing that might just help when it comes to remembering appointments.

0:3:44.260 --> 0:3:44.300
Interviewer
M.

0:3:38.130 --> 0:3:55.170
Respondent
And what else did I sign posting to? I think I sign posted into a podcast dementia podcast that was available through one of the [anonymised] organisations and I showed him how he could load that up on his phone, on Spotify and and he seemed quite happy with that.

0:3:56.770 --> 0:3:59.210
Respondent
But that was probably my first kind of experience.

0:3:59.850 --> 0:4:15.890
Respondent
Since then, it's about actually supporting the people who care for the people that have got dementia rather than supporting the dementia patient themselves. So that's been my primary experiences, really.

0:4:18.170 --> 0:4:31.290
Respondent
I went and did just it was. It wasn't even a course. It was a meeting. It was a meeting with other social prescribers in [anonymised], hosted by a lady who runs or works for the charity Volunteer [anonymised].

0:4:31.980 --> 0:4:46.740
Respondent
And shared us some Admiral nurses to come along and talk to us about the service that they can offer for patients as well. So then that was added like another bit of information for me another way, another little pathway of referring for specialist support.

0:4:48.140 --> 0:4:59.460
Respondent
Obviously the patient has to have a formal diagnosis of dementia in order to get the support from the Admiral Nurse team, and I've now got referral forms that they've sent to me.

0:5:1.300 --> 0:5:4.420
Respondent
So I think I've probably referred about 3 or 4.

0:5:5.220 --> 0:5:18.260
Respondent
Carers plus the patient that that the person, their loved one that they're caring for because you on the form it's about first of all you got to fill out the information about the person with the dementia diagnosis.

0:5:25.270 --> 0:5:25.470
Interviewer
Mm hmm.

0:5:29.280 --> 0:5:29.560
Interviewer
Yeah.

0:5:19.620 --> 0:5:35.540
Respondent
Then the part of the form is what kind of support are you looking for? What does the carer need? So it's a little bit of information about both parties, but I'll be honest with you, that would have meant only most most patients that I meet.

0:5:35.910 --> 0:5:56.590
Respondent
Errors have got third party consent and permission to make decisions for their loved one, so I've never really had to approach a patient directly to ask for consent. It's all been done via the carer, so that's been about my experience really of supporting patients with dementia.

0:5:57.60 --> 0:5:57.820
Interviewer
Yes.

0:5:58.510 --> 0:6:9.910
Respondent
Helping carers to know what what's available around, so we've got a memory cafe locally in the town where I work, but there's also other memory cafes around.

0:6:10.330 --> 0:6:11.210
Respondent
The area.

0:6:19.90 --> 0:6:19.490
Interviewer
Yeah.

0:6:13.250 --> 0:6:21.210
Respondent
But that's about it. In terms of my experiences, my training. Oh, the only thing I did recently actually was.

0:6:23.490 --> 0:6:26.490
Respondent
I signed up. It was via Eventbrite and it was.

0:6:28.410 --> 0:6:42.50
Respondent
An organisation called it was something like this adventure with dementia or dementia adventures, something like that, and it was an online course. I think it was only for about two or three hours and it was basically for family members.

0:6:42.450 --> 0:6:49.50
Respondent
Support them and how they can support their loved one in this journey that they're on together.

0:6:50.610 --> 0:7:7.250
Respondent
So although I was coming at it from a professional point of view, I learnt quite a bit from just going along and sort of working, working out what what it is actually what's what life is like day-to-day for the person with dementia and also the person that's doing the caring.

0:7:9.180 --> 0:7:10.260
Interviewer
That's that then.

0:7:9.90 --> 0:7:17.450
Respondent
We don't have. Yeah, we don't have a lot of dementia support places in [anonymised]. There's there's a couple of specialist day care centres.

0:7:17.770 --> 0:7:26.410
Respondent
They're nowhere near where I live, so to to refer patients into those services. I just, I don't think it would work. It's too far to travel.

0:7:27.970 --> 0:7:35.530
Respondent
But there has been talk of opening up a specialist dementia unit at our local cottage hospital.

0:7:37.370 --> 0:7:45.610
Respondent
But it's just getting the funding in place for that. I mean, that would be amazing for our area if we could have some kind of specialist unit.

0:7:47.960 --> 0:7:49.520
Interviewer
So that's the the.

0:7:48.480 --> 0:7:50.720
Respondent
Yeah. So that's about it, really.

0:7:50.980 --> 0:8:8.180
Interviewer
That's lots of very helpful information, [anonymised]. Thank you for that. I have some follow up questions because what you said is really interesting. So the first is just a point to clarify. Are you employed within [anonymised] so?

0:8:15.180 --> 0:8:15.460
Interviewer
Yeah.

0:8:21.280 --> 0:8:21.840
Interviewer
I say.

0:8:9.500 --> 0:8:40.540
Respondent
So yes, so I'm based at [anonymised], but my employer is a voluntary organisation. It's an [anonymised] so I that and that's actually how it tends to work in [anonymised]. I think pretty much all of the social prescribers, we're on temporary contracts were employed by charities or organisations, but we're based within medical practises. I think there's only one other SP.

0:8:40.580 --> 0:8:42.460
Respondent
That I've come across, who's employed, who's employed directly.

0:8:43.190 --> 0:8:45.110
Respondent
I the pcn, yeah.

0:8:50.310 --> 0:8:50.390
Respondent
Mm.

0:8:51.560 --> 0:8:52.760
Respondent
Yeah, I think so.

0:8:59.140 --> 0:8:59.540
Respondent
Yeah.

0:9:3.390 --> 0:9:3.510
Respondent
Hmm.

0:9:12.600 --> 0:9:13.120
Respondent
OK.

0:9:15.260 --> 0:9:15.460
Respondent
Mm hmm.

0:8:44.100 --> 0:9:18.780
Interviewer
Right. Yeah. OK. OK, that that kind of reflects the the landscape that I've seen nationally, 'cause, we're we're running the study nationally and and lots of social prescribers are employed from charities. Sometimes they're employed by pcns. So they work in different surgeries and it's the network that employs them and sometimes also have seen people employed by the local authority. So it's there's diversity out there, but you certainly fit within what I've learnt from other places as well.

0:9:19.660 --> 0:9:24.980
Interviewer
The other thing that I wanted to ask is that's quite interesting is.

0:9:26.420 --> 0:9:29.620
Interviewer
In your opinion, why do you think that you get?

0:9:31.340 --> 0:9:37.940
Interviewer
So very few people who actually are living with dementia as opposed to caregivers.

0:9:39.740 --> 0:9:44.740
Interviewer
In in referrals, do you know what I mean? Because you said it's mostly caregivers, yeah.

0:9:42.650 --> 0:9:48.50
Respondent
Yes, I do know what you mean. Yeah, I think, well, very possibly.

0:9:50.50 --> 0:9:50.850
Respondent
Because.

0:10:1.910 --> 0:10:2.190
Interviewer
Yeah.

0:10:2.270 --> 0:10:9.710
Respondent
So we're looking at telephone call appointments as being the main way that GPS are communicating with parents, parents, patients.

0:10:12.310 --> 0:10:20.910
Respondent
And and for some that telephone call might not be given, but it might be a waste of two months. This is what I'm hearing in worse. Sort of worst case scenarios.

0:10:21.420 --> 0:10:21.620
Interviewer
Mm hmm.

0:10:22.950 --> 0:10:24.990
Respondent
So I think that probably.

0:10:26.430 --> 0:10:33.390
Respondent
They are, I would say the GPS are probably not speaking or seeing their dementia patients face to face anymore.

0:10:34.280 --> 0:10:34.800
Interviewer
Right.

0:10:34.30 --> 0:10:35.190
Respondent
Or very rarely.

0:10:47.170 --> 0:10:48.170
Interviewer
Yeah, yeah.

0:10:55.940 --> 0:10:56.140
Interviewer
Mm hmm.

0:10:36.510 --> 0:11:4.270
Respondent
And I would imagine that the if there's concerns that it's the carer who is making the phone call and then they've got permission to speak to the GP on their patients behalf. So that's probably for me where my referrals are coming in is that the GP will have a consultation with the carer and have concerns over care or burnout, for example. How can I, as a social prescriber, support the care?

0:11:4.870 --> 0:11:27.990
Respondent
With what they're doing to give them a bit more strength, a bit more resilience, a bit more, whatever it is understanding about about the disease. But to be honest with you, the majority of carers that I've met who have been referred to me, they're far more experienced actually than I am with dementia, because they're living with it. They're living with their loved one who's got it.

0:11:29.950 --> 0:11:35.430
Respondent
It took me a little while to understand the process of how you get a diagnosis in [anonymised] as well.

0:11:36.530 --> 0:11:48.330
Respondent
So for example, I met with a patient just recently. She's early 80s. She's married to a chap who she believes has dementia. The GP believes, has dementia.

0:11:50.50 --> 0:11:52.90
Respondent
But the memory clinic.

0:11:52.930 --> 0:11:54.90
Respondent
Won't do.

0:11:55.370 --> 0:12:7.890
Respondent
An assessment with him because he is currently what has been using alcohol and is a well, he's what do they call it a funk. He's a functioning alcoholic.

0:12:8.540 --> 0:12:8.940
Interviewer
Yeah.

0:12:22.720 --> 0:12:23.0
Interviewer
Yeah.

0:12:9.80 --> 0:12:28.760
Respondent
And because of that and his alcohol use, they they won't assess him. So because of that, because he doesn't have a dementia diagnosis, I'm then stuck because I can't refer to specialist services like the Admiral Nurse team because he doesn't have a formal diagnosis. So this this lovely wife who? Well.

0:12:30.320 --> 0:12:31.600
Respondent
As you said, this is confidential.

0:12:32.240 --> 0:12:32.760
Interviewer
Yeah, yeah.

0:12:33.120 --> 0:12:40.40
Respondent
You know, she admitted that ideally in her world she she would have liked to have divorced her husband about 20 years ago.

0:12:40.540 --> 0:12:40.820
Interviewer
Yeah.

0:12:40.520 --> 0:12:52.920
Respondent
But didn't. And now she finds that she's she's just left in this really, really terribly awkward position where she has to care for him, but she doesn't really love him as as she did.

0:12:54.440 --> 0:13:6.840
Respondent
And she's angry, and she's angry at the lack of help and the lack of assessment for him because she says he only drinks at night time. Why can't they assess him during the day when he's sober?

0:13:7.230 --> 0:13:7.590
Interviewer
Yeah.

0:13:8.480 --> 0:13:11.240
Respondent
But there's that's the decision that's been made by the.

0:13:11.680 --> 0:13:13.520
Respondent
The clinic and not myself.

0:13:14.380 --> 0:13:16.380
Interviewer
So she's stuck, basically. She's still.

0:13:18.750 --> 0:13:19.590
Interviewer
Yeah, yeah.

0:13:15.240 --> 0:13:34.40
Respondent
So then I'm. Yes. Yeah. And then I'm a little bit stuck because I can't refer on. All I can do is signpost. I can signpost to the memory cafe, for instance, that online course that I did. Anyone can sign up to it. It's a free course on Eventbrite, and I suggested that she did that and found her. The link for it.

0:13:36.0 --> 0:13:45.960
Respondent
But it doesn't really. I mean like said. So she's in her early 80s, he's in his mid 80s and she's tired and she's got her own health problems as well.

0:13:46.500 --> 0:13:46.820
Interviewer
Yeah.

0:13:46.610 --> 0:14:2.530
Respondent
And I think he's just found it ever so frustrating. And what I also find interesting is the amount of people that I will sign post to the memory cafes for that specialist support. But they don't want to go.

0:14:2.770 --> 0:14:3.250
Interviewer
Hmm yeah.

0:14:3.970 --> 0:14:11.450
Respondent
The patient doesn't want to go, the carer doesn't want to have the battle with the patient to get them to go and yet.

0:14:12.890 --> 0:14:16.650
Respondent
If they could overcome that hurdle and they actually both went together.

0:14:31.500 --> 0:14:31.620
Interviewer
Hmm.

0:14:17.390 --> 0:14:34.630
Respondent
I think they'll find that they would really enjoy it because they'll see that there's people there that can support the patient in whatever it might they do, all sorts of wonderful activities, lots of singing and lots of music day trips, things like that.

0:14:35.950 --> 0:14:46.270
Respondent
And while that carer is there, they're not necessarily have to be sat with their loved one. They can be off in a different space, having a cup of tea and a chat.

0:14:46.750 --> 0:14:57.670
Respondent
With other people that are going through similar experiences. So it's that mutually supportive environment. But the battle, the hurdle is to actually get patients to engage and to go.

0:14:58.320 --> 0:14:59.640
Interviewer
Yeah, yeah, I I.

0:14:59.390 --> 0:15:6.550
Respondent
And that's where I feel stuck sometimes because I I can't magic any other resources out there, you know.

0:15:14.60 --> 0:15:14.420
Respondent
Yeah.

0:15:32.230 --> 0:15:32.470
Respondent
And.

0:15:7.40 --> 0:15:37.40
Interviewer
No, no. And and it's it's interesting because he said that most of the times, obviously you speak to the caregiver. So it's about caring for the caregiver, which means you're looking somehow at their needs in terms of care and and. And therefore, when it comes to addressing those needs by, for example, going in, going to a dementia cafe, you get stuck because from the perspective of the patient, you're not.

0:15:38.40 --> 0:15:42.440
Interviewer
Addressing what matters to them, if you know what I mean, right? It's tricky.

0:15:44.940 --> 0:15:46.940
Interviewer
With with the with the person, right?

0:15:47.910 --> 0:15:48.190
Interviewer
Yeah.

0:15:40.320 --> 0:15:54.800
Respondent
Exactly. Yes, yes, yes. You're not not having that conversation? Yes, you're right with with the actual patient. Yeah, you're only. I'm only in the position to advise or signpost to the caregiver. Yeah.

0:15:53.950 --> 0:15:55.310
Interviewer
Yeah, exactly.

0:15:56.360 --> 0:15:59.0
Respondent
Who will go back and feedback, perhaps to their loved one?

0:16:0.560 --> 0:16:6.880
Respondent
[anonymised] has suggested that we do this, and [anonymised] suggests we do that. But if if they don't want to do it.

0:16:8.240 --> 0:16:11.0
Respondent
Or if they feel it's not what they need at that time.

0:16:12.890 --> 0:16:13.890
Respondent
Yes.

0:16:12.720 --> 0:16:18.200
Interviewer
And I suppose with the mantra is also, it also changes day by day. That's also quite tricky, isn't it?

0:16:17.960 --> 0:16:23.320
Respondent
Absolutely. Yeah. I mean, I spoke to a lady this morning. She's in her 80s, her husband.

0:16:25.80 --> 0:16:28.680
Respondent
Has dementia. He has good days and bad days.

0:16:29.960 --> 0:16:52.720
Respondent
And she said it's wonderful and a good day. Everything's fine, you know? And and and life is as it used to be and we we can talk and do things together. But on a bad day she said it's just he he's abusive. He swears at me. He doesn't remember who I am. What what's going on and and I think for a lady in her 80s to be.

0:16:54.240 --> 0:16:59.240
Respondent
To be the only real caregiver. I mean, what they're looking into at the moment is a package of care.

0:16:59.640 --> 0:17:9.560
Respondent
It's an external care is coming in to support him, but that's not really around the dementia research. It's more around his physical needs.

0:17:10.160 --> 0:17:10.360
Interviewer
Mm hmm.

0:17:11.680 --> 0:17:36.520
Respondent
But the hope is that if somebody else externally comes in, it wants maybe two times a day, that just gives the wife just a little bit of respite and a little bit of a break. But that's best case scenario. I mean it might not even they might not be eligible for a package of care, you know, adult adult social care are looking into that now and they'll they'll be the ones to make that decision.

0:17:52.270 --> 0:17:52.390
Respondent
Hmm.

0:17:57.600 --> 0:17:58.320
Respondent
Yes.

0:17:37.50 --> 0:17:59.810
Interviewer
Yeah, I what I what I found really interesting about what you said before is that somehow the caregiver most of the times is acting as the social prescriber for the person living with dementia. And you are acting as a social prescriber for the caregiver. It's an interesting dynamic, isn't it? And maybe unique to dementia.

0:18:1.250 --> 0:18:1.570
Interviewer
Somehow.

0:18:1.510 --> 0:18:22.30
Respondent
Very possibly because there's also that you know where we are with consent. So let's say [anonymised] has a consultation with the caregiver about the person that's got the dementia and then the GP says, can I have your consent to contact the social prescriber, the referral?

0:18:23.610 --> 0:18:27.690
Respondent
Let's have a think, does it come the referral usually comes through.

0:18:31.20 --> 0:18:31.460
Interviewer
Yeah, yeah.

0:18:29.160 --> 0:18:35.720
Respondent
With the patient's name, but the details on the task from the GP are.

0:18:35.760 --> 0:18:42.640
Respondent
Along the lines of please can you contact the wife of or the husband of and then their contact details.

0:18:59.660 --> 0:18:59.940
Interviewer
Yeah.

0:18:44.10 --> 0:19:6.130
Respondent
Because I suppose if I were to ring the dementia patient themselves and and cut out the carer, then they might not have any recollection of any consent given or have any idea who I am. And and then we're just at that. So it has to, it almost sort of has to come via the caregiver.

0:19:6.540 --> 0:19:6.740
Interviewer
Mm hmm.

0:19:13.490 --> 0:19:13.890
Interviewer
Yeah.

0:19:7.410 --> 0:19:20.490
Respondent
But then rarely gets moved on to the person. I don't. I don't think I've ever been passed over on the phone. Oh, [anonymised]'s on the phone. Can you have a chat with? No, it doesn't really work like that, interestingly.

0:19:32.440 --> 0:19:32.520
Respondent
Mm.

0:19:40.40 --> 0:19:41.160
Respondent
Yes.

0:19:42.440 --> 0:19:42.920
Respondent
Yeah.

0:19:44.840 --> 0:19:45.280
Respondent
Yeah.

0:19:19.380 --> 0:19:50.740
Interviewer
Yeah. Is it fair to say that actually in situation where in situations where the patient leaves independently or they don't have anybody in terms of caregiver, that is actually the case where you somehow liaise with the actual patient you mentioned at the beginning, the old old guy that the old man that you're supporting has got the mild dementia. It's interesting because ironically it's there that you actually link with the person when there's no.

0:19:58.180 --> 0:19:58.300
Interviewer
Hmm.

0:19:48.700 --> 0:20:0.500
Respondent
Mm hmm. Yeah, I think that's the only link I've actually straight forward link I've had where I haven't gone via any other caregiver because he doesn't have a caregiver.

0:20:3.270 --> 0:20:8.710
Respondent
I'm just now thinking, are there any I suppose? Do you know what probably?

0:20:9.800 --> 0:20:12.600
Respondent
Let's think if there's somebody with severe dementia.

0:20:14.120 --> 0:20:14.640
Respondent
Living alone.

0:20:15.660 --> 0:20:15.780
Interviewer
Hmm.

0:20:16.0 --> 0:20:26.760
Respondent
They probably wouldn't actually be living alone. They would probably be put into a care home situation that would that that's I guess where I I think the understanding is for me.

0:20:31.380 --> 0:20:31.580
Interviewer
Mm hmm.

0:20:28.480 --> 0:20:39.80
Respondent
Is if somebody is is severe enough that they probably wouldn't be, wouldn't be able to live alone, and a decision would have been made via adult social care to.

0:20:40.300 --> 0:20:41.620
Respondent
Put them in a care home.

0:20:40.330 --> 0:20:42.530
Interviewer
So so actually.

0:20:44.10 --> 0:21:1.170
Interviewer
These brings us to actually conclude that social prescribing is applicable in cases where you either have a caregiver or family member or your early stage dementia. Is it fair to say you think?

0:21:3.600 --> 0:21:3.800
Interviewer
Mm hmm.

0:20:59.880 --> 0:21:15.720
Respondent
Yes, yes, I think so, yes. Yeah, I went to the other thing potentially actually with other social prescribers is how many home visits they they do. So I am, I'm allowed to do home visits.

0:21:20.0 --> 0:21:23.840
Respondent
But because it takes up such a lot of time.

0:21:27.400 --> 0:21:31.280
Respondent
Oh, I think I'm just. I'm just trying to think of an example where I visited.

0:21:32.640 --> 0:21:36.600
Respondent
A couple who where the husband and the wife? The wife had dementia.

0:21:38.400 --> 0:21:43.920
Respondent
The referral had come via, I think it came via the GP.

0:21:44.140 --> 0:21:44.260
Respondent
And.

0:21:45.390 --> 0:22:11.750
Respondent
I can't remember why, but I did a home visit rather than it be over the telephone and I'm, you know, when I when I met with the actual patient with dementia, she was absolutely fine. She was able to talk to me about her diagnosis. She did refer to her husband quite a bit in the conversation, just to double check. She'd say something like, isn't that right, Jeff, you know? Yeah. I remember going to the doctor. Isn't that right? [anonymised] would go. Yes, that's right.

0:22:11.720 --> 0:22:12.160
Interviewer
Yeah.

0:22:13.710 --> 0:22:16.110
Respondent
But they were actually.

0:22:17.410 --> 0:22:20.210
Respondent
They were ticking along quite nicely.

0:22:21.690 --> 0:22:50.90
Respondent
And I remember sort of almost questioning the referral, like what is it that, what is it that they wanted out of social prescribing, what did the referrer think that the couple were going to get from this? Because as this happens a lot with social prescribing just in general, though not not necessarily, dementia related is that maybe during a consultation a patient says, Oh yes, I'm finding life really difficult at the moment. And the GP thinks great social prescriber on the case.

0:22:51.250 --> 0:23:22.410
Respondent
I then get in touch and they say, Oh no, dear, no, I'm absolutely fine, dear. Oh, I was just all I remember. I was just having a a particularly bad day and the GP was very kind. And but I'm I'm actually doing OK and I don't really want your help. And then I sort of try and get in and another little angle. Is there any any other support here there you know, and get them to tell me a little bit about themselves. But sometimes it just does come back with. No, I'm doing fine and then I leave them my number and if they need to get back in touch with me they can.

0:23:23.90 --> 0:23:25.250
Respondent
And I think that's what happened with the couple.

0:23:26.120 --> 0:23:27.680
Respondent
They already knew, yeah.

0:23:26.240 --> 0:23:30.280
Interviewer
Can I just can I just chip in [anonymised] about the couple?

0:23:31.760 --> 0:23:39.600
Interviewer
Does does this make you think that somehow when there's a face to face contact with patients and families in general?

0:23:41.400 --> 0:23:48.360
Interviewer
There's more engagement and it these kind of produces a bit more effective results than over the phone.

0:23:53.840 --> 0:23:55.160
Interviewer
It didn't, OK.

0:23:45.540 --> 0:23:57.100
Respondent
Yes. Well, I'd like to thinks so, yes, actually I. But in this case, it didn't particularly come to anything just in this particular case.

0:23:58.540 --> 0:24:10.460
Respondent
But then I don't think that I think the lady with dementia, she was actually doing OK. She'd had a diagnosis, but I don't think things had deteriorated. She felt she had very good family support.

0:24:11.940 --> 0:24:14.900
Respondent
She had good friends who came and sat and spoke with her.

0:24:22.120 --> 0:24:22.520
Interviewer
Yeah.

0:24:16.100 --> 0:24:35.620
Respondent
Her husband was able to go off and do some things independently while family members came to sit with her. So in terms of that they were they were OK. They knew about the memory cafe. They didn't want a referral to the Admiral nurses because they didn't feel that things had deteriorated to that extent.

0:24:36.200 --> 0:24:36.400
Interviewer
Mm hmm.

0:24:36.860 --> 0:24:41.260
Respondent
But then I'm just thinking of a conversation I had this morning talking to a wife.

0:24:42.940 --> 0:24:44.620
Respondent
And I've made this.

0:24:45.100 --> 0:24:48.20
Respondent
So the first appointment was via telephone call.

0:24:49.780 --> 0:24:55.940
Respondent
Just a sort of triage and work out what was going on for her and and for her husband.

0:24:57.380 --> 0:25:12.460
Respondent
My call today was a follow up like a little check in and chat call to see because I knew a referral had gone. I didn't do the referral, but a referral had gone into adult social care requesting a package of care and for occupational therapy to come and do a visit.

0:25:14.420 --> 0:25:17.820
Respondent
So I just wanted to kind of kind of check in check in with her today to see.

0:25:18.250 --> 0:25:39.10
Respondent
If adult social care had been in touch, which they had, I wanted to see if she'd been able to sort of convince her husband to go to the memory cafe. And that was a definite no. But I had signposted her to a monthly support group called [anonymised] and.

0:25:40.220 --> 0:25:54.740
Respondent
I don't think it's necessarily dementia related. I think it can be anyone who is a carer and anyone who is being cared for can go along to this group, but it is only once a month.

0:25:54.780 --> 0:26:11.380
Respondent
And my last conversation with her since that conversation in today's phone call, the group had actually met yet. So she's going to try and go in November because it's on like, the third Wednesday of the month. It's really hard to remember when these things are.

0:26:12.340 --> 0:26:17.300
Respondent
Issues put it in her diary and she said that she would really try and take her husband along to that.

0:26:19.100 --> 0:26:32.900
Respondent
So then I've now put it in my diary to give her another telephone call in about four weeks time as another little check in and chat to see how she's doing. But again, it's because I suppose the check in and chat is more for her.

0:26:34.260 --> 0:26:37.380
Respondent
Rather than, it is for the the man with the dementia.

0:26:40.650 --> 0:26:42.10
Respondent
But that's yeah.

0:26:40.570 --> 0:26:46.850
Interviewer
In which case I suppose phone contact is fine because I suppose yeah with with a caregiver.

0:26:44.710 --> 0:27:9.590
Respondent
Yeah, but if I can't, if I done a home visit, for example, and this is what's making me think now instead of it being a telephone call as a follow up, let's say I arranged a home visit next time and he met me and I met him and we were able to sort of have a chat together and find out what matters to him, have that social prescribing conversation if possible.

0:27:10.710 --> 0:27:15.110
Respondent
He might then perhaps be a little bit more open to doing something like the memory cafe.

0:27:15.540 --> 0:27:16.220
Respondent
And.

0:27:16.890 --> 0:27:17.10
Interviewer
Hmm.

0:27:23.0 --> 0:27:23.280
Interviewer
Yeah.

0:27:18.230 --> 0:27:24.510
Respondent
But this is the chap that has his good days and then he has his very bad days. So it's.

0:27:26.290 --> 0:27:34.610
Respondent
It's really it's again, it's a good, good question and maybe it may be a home visit should be something when you get a referral through.

0:27:36.60 --> 0:27:36.900
Interviewer
I mean, I yeah.

0:27:36.60 --> 0:27:41.100
Respondent
Around a patient with dementia? Yeah. To maybe make prioritise it as a home visit.

0:27:40.580 --> 0:27:47.780
Interviewer
If it's, if it's an option, because what I've heard umm [anonymised] from other services is that unfortunately not always.

0:27:49.940 --> 0:27:50.540
Respondent
Yeah, yeah.

0:27:51.590 --> 0:27:52.30
Respondent
Yeah.

0:27:49.280 --> 0:27:56.80
Interviewer
This is a possibility for for social prescribers. Some are really discouraged, to be honest, to to, to go out.

0:28:4.620 --> 0:28:4.740
Interviewer
Hmm.

0:28:12.680 --> 0:28:13.800
Interviewer
Yeah, yeah, yeah.

0:28:16.560 --> 0:28:17.440
Interviewer
Yeah, it.

0:27:54.690 --> 0:28:20.810
Respondent
Yes, and I it's funny enough within our team. So I work for [anonymised] and my other team members work for a charity mental health charity called [anonymised] are well, we're not. It's not necessarily encouraged, but we're not discouraged from doing a home visit, whereas my pen trees colleagues are definitely discouraged and it's very much telephone call or face to face in the surgery.

0:28:22.330 --> 0:28:22.490
Respondent
Yeah.

0:28:22.160 --> 0:28:24.160
Interviewer
It varies. It varies a lot, so.

0:28:24.590 --> 0:28:25.510
Respondent
I can imagine.

0:28:25.880 --> 0:28:39.120
Interviewer
But, I can see that in some cases, particularly with people living with dementia, if you really want to sort of gauge what matters to them as opposed to the caregiver, it it could be ideal in some respects.

0:28:40.880 --> 0:28:42.560
Interviewer
OK. That's that's really interesting.

0:28:44.80 --> 0:28:47.640
Interviewer
Another question that I wanted to ask you is.

0:28:55.430 --> 0:28:55.550
Respondent
Hmm.

0:29:0.400 --> 0:29:0.800
Respondent
Yeah.

0:29:2.10 --> 0:29:3.10
Respondent
Yeah, yeah.

0:28:48.890 --> 0:29:7.330
Interviewer
What? What would you wish you had known? Because you talked about training and it seems from what you said that it's always been down to you to your personal kind of interest of signing up to stuff as opposed to sort of mandatory. So I was wondering, what would you wish you had known?

0:29:8.850 --> 0:29:14.210
Interviewer
When you first started off in the job in relation to dementia, that would have been really helpful.

0:29:21.690 --> 0:29:22.210
Interviewer
Hmm mm hmm.

0:29:13.980 --> 0:29:42.380
Respondent
Think what would have been very helpful right from the start would be to know how someone gets diagnosed with dementia from the first point of that conversation with the GP of, oh, I'm a bit worried that I'm forgetting things and I don't. Yeah, I'm not sure what's going on to to then what the GP then does to, then how that gets referred to the memory clinic and then who makes those decisions at the memory clinic, who makes that diagnosis?

0:29:42.610 --> 0:29:45.970
Respondent
So that would have been really useful to have known that from the start.

0:29:46.20 --> 0:29:49.260
Interviewer
And can I ask you why you think it would have been helpful?

0:29:48.690 --> 0:30:0.210
Respondent
I think probably just so when I then meet with patients, I know what their experience has been and where they might be on their path or their little journey with it, you know, within dementia.

0:30:0.580 --> 0:30:1.340
Interviewer
Yeah, yeah.

0:30:2.10 --> 0:30:15.730
Respondent
So I might come across. I don't think I've come across anyone who's saying I think. I think I may have dementia, but I don't know what to do about it. I don't think I've had that consultation yet. But what I have had is.

0:30:16.840 --> 0:30:25.320
Respondent
I my memory is declining. I went to see my GP. They told me they asked me a set of us. It's like 6 or 7 questions I think.

0:30:26.880 --> 0:30:49.760
Respondent
And then based on my responses, they either said yes, I'm concerned I'll refer you on to the memory clinic or no. You've answered the question satisfactorily, so you're OK for now, but we'll monitor you. I never really get because of a lot of social prescribers. The whole point of us really is we're not medically trained. I don't think they want people necessarily coming from a medical background.

0:30:50.160 --> 0:30:50.480
Interviewer
Yeah.

0:31:3.700 --> 0:31:3.780
Interviewer
Mm.

0:30:51.290 --> 0:31:13.130
Respondent
But just having that not really having that essential bit of information about how the referral process and the dementia diagnosis happens, that's that's been quite key for me to learn that. And then what I really wish I'd known right from the start as well is things like about the Admiral nurses and at the Alzheimer's Society.

0:31:14.410 --> 0:31:18.410
Respondent
And the fact that a social prescribers, we can refer to them for support.

0:31:20.530 --> 0:31:21.690
Respondent
The trouble is.

0:31:23.260 --> 0:31:27.740
Respondent
With training, I suppose is that with, because with social prescribing.

0:31:31.380 --> 0:31:32.220
Interviewer
Yeah, yeah.

0:31:32.960 --> 0:31:34.240
Interviewer
Literally, yeah.

0:31:29.60 --> 0:31:53.60
Respondent
You never know what the referral is going to be about. It could be anything, and so anything. And so if you're told right at the start, I mean I we do have lists of resources we have, you know, lots of things we can look at in terms of online stuff. But I think with this it's like your own journey into it. You have to sort of learn as you go.

0:32:5.380 --> 0:32:5.540
Interviewer
It.

0:32:12.170 --> 0:32:12.410
Interviewer
Yeah.

0:31:54.940 --> 0:32:20.780
Respondent
But yes, it was a revelation. After about, let's see, I was probably about 8 months into the job and I remember being this in this meeting and a couple of dimension Admiral nurses stood up and told us about what they'd and had never heard of them. I didn't even know they existed. And I just thought, how could I have done this job all this time and not know that you're even out there? But then that was the whole point of the meeting was to tell people about it.

0:32:20.30 --> 0:32:22.990
Interviewer
Yeah. Is it fair to say that?

0:32:24.310 --> 0:32:28.670
Interviewer
Your job by nature is more reactive than proactive.

0:32:30.590 --> 0:32:31.30
Interviewer
Yeah.

0:32:29.280 --> 0:32:31.600
Respondent
I suppose so, yes. Yeah, I think.

0:32:49.710 --> 0:32:49.990
Interviewer
Yeah.

0:32:34.640 --> 0:33:0.880
Respondent
It's funny because trying to support colleagues who are who are now new to social prescribing, so we've had a couple of new members to the team and it's it's like how much information do I give them at the start? I don't want to overwhelm them. So and and again, it's almost like they need to find their own path. But we are the team have got a WhatsApp group. So if anyone is ever struggling at home thinking or working in the office.

0:33:5.810 --> 0:33:6.10
Interviewer
Yeah.

0:33:1.400 --> 0:33:11.240
Respondent
And they come across something like I have no idea who to refer this patient to. What can I do for support here? We message each other and between us we've got this collective knowledge.

0:33:11.900 --> 0:33:11.940
Interviewer
M.

0:33:12.480 --> 0:33:14.800
Respondent
And we're constantly building on that.

0:33:20.500 --> 0:33:20.860
Interviewer
Yeah.

0:33:22.860 --> 0:33:25.420
Interviewer
Yeah, yeah, sure, sure. Take your time. Don't worry.

0:33:29.280 --> 0:33:29.600
Interviewer
Ha ha.

0:33:16.320 --> 0:33:30.520
Respondent
But it does. It does take time. I think, just to build up that knowledge will excuse me, a man. I'm just going to go and get a glass of water. Sorry, I'll just have been. It's a day of doing a lot of talking. I've been on the phone to patients. Bear with me a SEC.

0:33:30.670 --> 0:33:31.70
Interviewer
Yeah.

0:33:51.570 --> 0:34:9.370
Respondent
And that's better. Thank you. Yeah. So if let's say I started in July 2022, if somebody had told me all then about Alzheimer's Society, about memory cafes, and about the Admiral nurses and about the memory clinic.

0:34:10.930 --> 0:34:21.10
Respondent
Yes, I could have taken that information and I probably would have written it down somewhere, would have stored it somewhere, put it on my computer and then I might not have met a patient for six months.

0:34:20.720 --> 0:34:21.800
Interviewer
Exactly, yeah.

0:34:21.810 --> 0:34:24.130
Respondent
And this is yeah, so.

0:34:33.80 --> 0:34:33.520
Respondent
Yeah.

0:34:35.750 --> 0:34:37.270
Respondent
No, no.

0:34:47.80 --> 0:34:48.280
Respondent
Yes. Yeah.

0:34:23.480 --> 0:34:51.840
Interviewer
And and and by the time you may meet a patient, the information might not be up to date anymore. That's why I'm saying by nature. You you tend to be reactive. You can't know anything. Everything about anything beforehand. That's just the nature of it. Yeah, but but. But also great point you made about having a supportive team where expertise may lie in another person and sharing with them on WhatsApp when when needed that that seems to be what's. Yeah.

0:35:6.80 --> 0:35:6.480
Interviewer
Yeah.

0:34:50.0 --> 0:35:21.200
Respondent
Absolutely. I found that that's been incredibly useful and I'm very lucky because I don't think I've. I've met other other social prescribers who feel very isolated in their jobs. And I think that's why actually a lot of people have left the profession is because they didn't. They didn't feel that they had any peer support and they didn't necessarily feel they had support in terms of clinical supervision from the GPS and their practise. And we're just sort of out on a limb doing the best that they could but.

0:35:21.560 --> 0:35:32.560
Respondent
Yeah, if you don't. If I if I didn't have the team around me, I think I really would have struggled and might have might have sort of second guessed or questioned whether or not this job was right for me.

0:35:34.200 --> 0:35:43.240
Respondent
But I have been very fortunate and and I do, I really do enjoy the job and I feel like I mean, every day is a school day. I'm learning constantly.

0:35:45.950 --> 0:35:46.950
Respondent
Yes. Yeah.

0:35:44.240 --> 0:35:51.520
Interviewer
But that's also what's exciting about it cause 'cause it's never the the the same the same old thing, every single that, yeah, yeah.

0:35:49.570 --> 0:35:54.450
Respondent
Absolutely. Yeah. Yeah. Oh, my gosh. Yes. It's never the same.

0:35:54.820 --> 0:35:57.260
Interviewer
Yeah, sometimes a bit too much maybe.

0:36:22.450 --> 0:36:22.690
Respondent
And.

0:36:24.370 --> 0:36:24.450
Respondent
Mm.

0:35:58.540 --> 0:36:28.620
Interviewer
OK. So that that's really, really helpful, [anonymised]. Thanks a lot. It gives me good kind of snapshots of what how you guys operate and and and what the challenges have been so far for you and it's also very interesting because we've interviewed lots of people from all over the country, but actually you're the first person I'm interviewing from the southwest which is, you know particular especially when it comes to a reality and all that that comes with it so.

0:36:29.190 --> 0:36:29.350
Interviewer
Yeah.

0:36:34.340 --> 0:36:34.740
Interviewer
Yeah.

0:36:27.310 --> 0:36:36.750
Respondent
Yes, yes. And [anonymised] has got, oh, huge, huge problems with transport because of its rarality.

0:36:38.190 --> 0:36:58.150
Respondent
Yeah, just thinking about where I am, where I'm based there. There may well be lots of things that we could link patients to within [anonymised], but if it's North [anonymised], you're talking a 2 hour drive from where we live and it's just where as I'm just thinking of other counties where I've lived in where it's perhaps much easier to access.

0:36:58.880 --> 0:36:59.120
Interviewer
Uh huh.

0:37:8.420 --> 0:37:8.820
Interviewer
Yeah.

0:37:11.860 --> 0:37:12.340
Interviewer
Wow. Wow. Wow.

0:36:59.110 --> 0:37:13.70
Respondent
Face to face groups and things like that, because I don't know. Maybe things are a little bit more sensual, but if you think [anonymised] is just this long stretch of it goes down, down and down and we're literally right on the tip of it, yeah.

0:37:14.710 --> 0:37:14.910
Respondent
I mean.

0:37:14.70 --> 0:37:30.110
Interviewer
I suppose maybe in in some respects that has kind of affected the possibility for you to to travel on on the part of your employer somehow, although you said that the other social prescribing link worker is not encouraged actually to to visit.

0:37:37.200 --> 0:37:37.280
Interviewer
Mm.

0:37:28.700 --> 0:37:41.780
Respondent
Yes, I mean, I probably the furthest I've had to travel for a home visit, it's not. It's probably not been too far, probably 5 miles tops. So it's not. It's not too onerous, it's I think.

0:37:51.110 --> 0:37:51.190
Interviewer
Mm.

0:37:43.500 --> 0:38:6.420
Respondent
Well, OK, I'll be, I'll be honest. So I work in the surgery 2 1/2 days out of the week and the rest of the time is I try and protect it as admin time. For instance, I've had my colleague that I share the office with is she's on annual leave at the moment, so all of the referrals are coming through to me.

0:38:6.870 --> 0:38:7.670
Interviewer
Yeah, yeah.

0:38:34.620 --> 0:38:34.740
Interviewer
Hmm.

0:38:7.580 --> 0:38:38.140
Respondent
And I need to have that time to ring those patients. Now, if I'd set aside today, Monday as, oh, I'll go and do my home visits today, then I'm really then squeezing my time to and then it becomes quite stressful and a little bit unmanageable. I need to protect my time where I've got a space in the surgery. I'm very, very lucky. They give us a dedicated room. But equally within the same town in another surgery.

0:38:49.30 --> 0:38:49.790
Interviewer
Oh gosh.

0:38:54.220 --> 0:38:55.580
Interviewer
In the community, yeah.

0:39:1.720 --> 0:39:2.760
Interviewer
Yeah, yeah.

0:39:6.210 --> 0:39:6.970
Interviewer
Know each other.

0:39:8.580 --> 0:39:9.60
Interviewer
Yeah.

0:38:38.870 --> 0:39:10.230
Respondent
I've got there's two social prescribers that work there and there's no space to see patients in the surgery. There's no space. That all has to be telephone calls or or they meet in a an agreed location like a coffee shop. Yeah. And that's so it's OK. But if you're talking about very confidential things, it's not great and it's a small community. So people might think, oh, why is that person doing that over there in the corner of Costa Coffee, you know?

0:39:36.720 --> 0:39:36.960
Interviewer
Yeah.

0:39:10.690 --> 0:39:42.290
Respondent
The other thing that's takes that's been taking up a bit of our time is that we, we do get involved with community groups as well. So setting up community groups, going along and making sure that things are going well at community groups. So I'm involved in one that's trying to combat social isolation. So there's there is pressure on my time to attend that each week and make sure that the group are ticking along quite nicely, meeting new patients who are very anxious about coming into a new space for the first time.

0:39:43.620 --> 0:39:47.220
Respondent
Oh, I'm just sorry. My computer's locked you out. Are you still there?

0:39:47.280 --> 0:39:49.720
Interviewer
Yeah. Yeah, I'm here. Yeah, that's all.

0:39:48.580 --> 0:39:50.180
Respondent
You've just disappeared off my screen.

0:39:55.190 --> 0:39:56.390
Interviewer
Yeah. So.

0:39:52.630 --> 0:40:9.510
Respondent
Oh, and I've put in the wrong password. Oh bear with me a SEC. And the other thing that social prescribers have done recently, and I've been very involved in this, is setting up something called a pain cafe. So that's helping to support patients in a non medical way. Patients who live with persistent and chronic pain.

0:40:11.70 --> 0:40:17.70
Respondent
So yeah, so when it comes to do I do I do home visits? Yes, I do.

0:40:18.370 --> 0:40:26.850
Respondent
But it's usually really only if the patient physically cannot get to the surgery, and they might be hard of hearing. So a telephone call isn't going to work.

0:40:28.490 --> 0:40:37.50
Respondent
Yeah, I'm just thinking I'm visiting a lady this week because she's had a couple of strokes. She's she can't speak on the telephone because of the strokes.

0:40:38.570 --> 0:40:46.570
Respondent
So I go and going to go and visit her at her home and meet her son there with her. She's in her 80s. She's got poor mobility.

0:40:47.500 --> 0:41:2.700
Respondent
To try and get her into a taxicab to get her to come and visit me in the surgery, it's just it's not going to work. So that's when I'll do things like a home visit. But having had this conversation with you today, it has opened my eyes a little bit to actually.

0:41:3.700 --> 0:41:11.820
Respondent
It might be really useful to go into a home visit to the dementia patient and the caregiver at the same time to see.

0:41:10.360 --> 0:41:41.40
Interviewer
Yeah. Yeah. What I've heard from other social prescribers who do believe it's important is also around kind of assessing about the environment with your own eye. You know, sometimes you visit a person living with dementia and you use specific strategies that allow you to see where they are in terms of their journey. So one social prescriber told me that she kind of she goes into people's homes and she asked for a cup of tea and the moment.

0:41:41.80 --> 0:41:44.600
Interviewer
The person opens the opens the fridge, she's able to see whether there's enough food in the fridge.

0:41:44.80 --> 0:41:45.480
Respondent
Yeah, yeah.

0:41:45.320 --> 0:41:53.560
Interviewer
Or whether you know the milk comes out of the bottle in lumps, that sort of stuff that you're only able to see if you're in in the person's environment.

0:42:0.200 --> 0:42:0.560
Interviewer
Yeah.

0:41:53.160 --> 0:42:7.480
Respondent
Absolutely. I mean, it is a real it, yes, it's a real eye opener when you go and do a home visit. Yes, you do. You get so much more from it than you would a telephone consultation or even a consultation in the office.

0:42:8.840 --> 0:42:10.240
Respondent
In the in the medical practise, yeah.

0:42:28.700 --> 0:42:31.180
Respondent
Yes, brilliant. OK.

0:42:12.110 --> 0:42:32.710
Interviewer
And I just wanted to say, [anonymised], what's the information that we've collected so far, including what I've just said to you about home visits? We're all including in in this resource. So hopefully it will be helpful for other people to to see what others have done in terms of that. Yeah, it will be all in there.

0:42:34.510 --> 0:42:41.350
Interviewer
Let me see. Was there anything final that you wanted to add or are you happy with our conversation so far?

0:42:48.860 --> 0:42:49.700
Interviewer
Yeah, yeah.

0:42:40.710 --> 0:42:51.710
Respondent
Yeah. No, that's been fine. No, that's it's that's been good to talk to you and it be. Yeah, it'd be interesting to see what your study sort of comes up with in terms of resources and.

0:42:59.680 --> 0:43:0.0
Respondent
Yeah.

0:42:51.640 --> 0:43:20.880
Interviewer
What's what? We'll do, [anonymised]. I I explained this to every every participant is that I will keep in touch with updates. And maybe when the time comes for for the group workshops where other support. So social prescribers will be together to discuss further development of the resource. I will make contact with you and see whether you're interested. We're going to be doing this remotely. So there's no, you know, travel implications. But I think it's going to be interesting in terms of.

0:43:21.760 --> 0:43:23.360
Interviewer
Networking with other social.

0:43:24.210 --> 0:43:24.970
Interviewer
Prescribers.

0:43:34.610 --> 0:43:35.170
Interviewer
Yeah.

0:43:23.210 --> 0:43:40.370
Respondent
Absolutely. Yeah. No, I I think it's quite it. It's nice to be involved in a study like this and and to sort of almost find out what other social prescribers do and don't do and so on, especially at in terms of not necessarily being in [anonymised], but outside of the southwest to see, yeah.

0:43:45.320 --> 0:43:45.400
Respondent
Mm.

0:43:40.560 --> 0:43:50.80
Interviewer
Yeah, it's, it's, it's good opportunities for you know, sharing and learning. That's what I always find and and it was today with you. So thank you so much for that.

0:43:49.690 --> 0:43:52.290
Respondent
I have no problem at all. I'm happy to be of help. Oh, it's nice to meet you. Thanks very much. OK, you take care. OK. Bye. Bye bye.

0:44:43.70 --> 0:44:47.350
Interviewer
It was lovely to meet you too. Thank you. Take care. Bye. Bye bye.

**SP24**

0:0:0.0 --> 0:0:0.120
Interviewer
You.

0:0:1.470 --> 0:0:20.590
Interviewer
To the issue of potentially social prescribing at the moment being just for, you know, people who are at early stages who are really well supported by somebody and by default somewhat excluding lots of people who are living independently or maybe at later stages in the process.

0:0:21.220 --> 0:0:41.180
Respondent
I think the whole thing like so recently I updated my criteria for social prescribing and this is this is a whole broader thing and basically it's got to be anybody that's that's willing to make a change and that wants to make a change and they have to have obviously capacity.

0:0:42.460 --> 0:0:53.820
Respondent
We're not support workers and I think like we have been given so many when we first started, we were, we were just given clients that nobody else knew what to do with because they weren't services.

0:0:54.430 --> 0:1:6.70
Respondent
So when I first started, we were getting mental health crisis. We were getting, you know, further down the line with dementia where, you know, I had a chap that was on his own.

0:1:9.70 --> 0:1:9.190
Respondent
And.

0:1:22.130 --> 0:1:22.370
Interviewer
Yeah.

0:1:9.270 --> 0:1:24.510
Respondent
He you know, I suggested all these things to him and he didn't remember them. He didn't. He didn't. You know, I rang back on my my follow-ups and he hadn't remembered even speaking to me. So it's that it's that capacity, you know, people that we support have got to be.

0:1:25.290 --> 0:1:28.50
Respondent
At that point, when they can make the decisions and they can.

0:1:29.510 --> 0:1:41.350
Respondent
You know, and I mean, if I think with that service, I think there needs to be more of a specialised service to help people with dementia that are on their own. And I actually think that it's a, it's.

0:1:43.110 --> 0:1:44.790
Respondent
It's a gap in provision.

0:1:45.280 --> 0:1:46.360
Interviewer
Mm hmm M.

0:1:46.80 --> 0:2:16.400
Respondent
And I think as social prescribers, we're we're sometimes expected too much to to solve everything and and and you know in the five years of working as a social prescriber like I've got a team of people now in our PCN that I can refer to. So we have a mental health practitioner and then the nurses, you know, the dementia nurses and things they they tend to deal more with that side of things. So I think it's not, it's not a factor being inclusive I.

0:2:16.440 --> 0:2:20.200
Respondent
So we are inclusive, but I think it's a factor of being very clear of our.

0:2:21.390 --> 0:2:24.590
Respondent
Criteria our boundaries as social prescribers.

0:2:26.310 --> 0:2:28.790
Respondent
And I think if anybody.

0:2:28.870 --> 0:2:31.990
Respondent
Requires that additional support where it's at 1 to 1 support.

0:2:36.230 --> 0:2:36.550
Interviewer
Yeah.

0:2:32.810 --> 0:2:43.450
Respondent
Like a support worker, we we can't do that role. Our role is linking somebody to a service, to information, to to advice guidance.

0:2:44.60 --> 0:2:44.260
Interviewer
Mm hmm.

0:2:45.450 --> 0:2:54.970
Respondent
And I and I think you know, unless unless you're a specialised social prescriber, I know there's some roles now, aren't there where there are these new roles being developed.

0:2:56.950 --> 0:2:59.550
Respondent
Then yeah, I mean that's my, that's my feeling on it.

0:2:59.600 --> 0:3:0.0
Interviewer
Yeah.

0:3:1.390 --> 0:3:5.670
Respondent
I mean it's the same with, you know, patients with learning disabilities.

0:3:20.280 --> 0:3:20.560
Interviewer
Yeah.

0:3:7.190 --> 0:3:29.150
Respondent
If they're learning disability, prevents them from being able to access something independently, then I would do a referral to adult social services for a needs assessment to look at things we can get them a support worker and I think it should be the same with dementia as well, that if they're at that point where they wouldn't be able to access something independently for for whatever reason.

0:3:31.110 --> 0:3:48.910
Respondent
It's that looking into. Is there somebody that you know is there a service that could support them? Is is there a family member that can support them and and looking into that kind of side of what they're what their support network is. But again, that wouldn't be something we would really do. We we would do a referral to a service to look into that.

0:3:51.290 --> 0:3:54.490
Respondent
And I work really closely with adult social services and.

0:3:57.170 --> 0:3:57.650
Respondent
Again.

0:3:57.690 --> 0:4:16.970
Respondent
I had another referral for a lady in a village that had dementia, and I kept ringing her to invite her to the information hub because it was literally around the corner from her and I thought, well, if she came, adult social services are there, you know, we've got home groups, she can come and have a cup of tea. She never, ever turned up. And then.

0:4:18.650 --> 0:4:21.130
Respondent
Adult social services said that, you know, she.

0:4:22.290 --> 0:4:34.690
Respondent
Was going wandering around the village. She was very confused. She she was feeling that somebody was sort of like after her and like, and I just thought at that point it was like well.

0:4:36.850 --> 0:4:51.810
Respondent
Adult social services were then involved and you know that that was the right service to be involved in at the time and in the end a neighbour, I said, well, you know, if if is there anyone that could sort of bring, you know, remind her and bring her to the hub and in the end the neighbour brought her.

0:4:52.850 --> 0:5:2.10
Respondent
But but again, it, you know, wasn't she came and it wasn't really sort of what she was looking for or what, you know, just nothing sort of came about it but.

0:5:10.560 --> 0:5:10.600
Interviewer
M.

0:5:22.380 --> 0:5:22.700
Interviewer
Yeah.

0:5:29.780 --> 0:5:29.900
Interviewer
Hmm.

0:5:4.530 --> 0:5:33.10
Respondent
Yeah, I I just feel we need a service. I almost feel like we need a dementia befriending service or that that sort of that's something in between because, you know, we can still signpost people to projects and things like that. But I think that it's just having a service that could go with them. And I know a lot of the dementia service in [anonymised], they do go with them to the memory cafes if they haven't got family members. But I think even that they're very.

0:5:33.50 --> 0:5:37.770
Respondent
You know, they're their full capacity and I just think we need more money put into.

0:5:41.440 --> 0:5:41.720
Interviewer
Yeah.

0:5:39.210 --> 0:5:47.490
Respondent
The same with mental health as well. You know, we need more money put into these additional services that can help people to access them.

0:5:48.290 --> 0:5:57.970
Interviewer
Do you do you feel at the moment you have 'cause you talked a bit before about a bit of a pressure on social prescribers to almost, almost feel like?

0:6:1.430 --> 0:6:1.870
Respondent
Yeah.

0:6:11.420 --> 0:6:11.900
Respondent
Yeah.

0:5:59.450 --> 0:6:17.970
Interviewer
Their role does not stop at signposting, but actually there's an expectation that somehow you take on that kind of gap that you mentioned before in linking the person to the actual service or resource in the community. Do you feel that expectation and how do you actually sort of?

0:6:20.130 --> 0:6:25.490
Interviewer
Address it because I'm sure you haven't got, you know, capacity or resources to do that.

0:6:24.890 --> 0:6:58.690
Respondent
Yeah. Yeah. And sometimes when there isn't anything to signpost people to, you're almost holding them. You're almost holding them in this place for quite a long time until you find something that you can refer. And I I have been in that position. I mean, I've had to get really stricter on my criteria, and I've had to bounce stuff back to the referrer's to say, you know, I'm really sorry, but this isn't suitable, but at least I'm in a position now where I do have we do have more staff members within our PCN. And. And I'm finding that that's that's helped. We did have a care coordinator.

0:6:59.790 --> 0:7:18.870
Respondent
Which which she her role was to be working with our more vulnerable patients and dementia patients as well. And she did do the training. She did that, the free level trainings and then she left. So I am starting to get those referrals back again where where they are more that that care coordinator referrals you know.

0:7:20.510 --> 0:7:33.710
Respondent
So it is a tricky 1 because I try and get stricter on on my criteria, but sometimes you you do feel for people and it might be that you know, I had a lady again, you know, with dementia. She she did have a son living with her.

0:7:34.520 --> 0:8:10.360
Respondent
But it was just a little thing like she she had her eyes tested and her hearing done for so long, and her son was quite young or grandson, I think it was. And he didn't really know how to sort of, you know, link up to services. And I had a social worker saying offer, you know, I'm really desperate for you to just would you be able to sort of signpost her some bits and and again, I sort of thought, well, that's kind of your role, isn't it? You've been working with them. But I said, yeah, that's fine, I'll and. And I phoned her up and she she didn't understand who I was or what what I was trying to do. And then the grandson I was handed over to the grandson and I.

0:8:21.770 --> 0:8:22.130
Interviewer
Yeah.

0:8:37.940 --> 0:8:39.100
Interviewer
Yeah, yeah.

0:8:10.800 --> 0:8:41.40
Respondent
Explain the service that would do home and eye tests, but again, I was expected to book that with the lady and I was like, I wouldn't know her days or times when she's available and I don't do that kind of thing again. That's going. That's going back to support work. And I used to be a support worker. That's my background. Like I've I've worked in all sorts of, you know. And so I think that's why I can, like, tell the difference between when you're going over your boundary of a social prescriber to like, you're going into more care kind of side, you know.

0:8:42.990 --> 0:8:43.590
Respondent
But yeah.

0:8:45.590 --> 0:8:57.310
Respondent
I I yeah, I do think we need more things and I think the memory caps are great and I think you're right. You know there is a sense that we are missing out on people that can't access it because they haven't got anyone to come with them.

0:8:59.690 --> 0:9:29.850
Respondent
But we're we are working on that and I have invited loads of organisations that are LinkedIn with dementia like so things like the sensory trust. There's a lady that comes from there. We've got two dimension nurses that come. We've got adult social services that come that the lady that's got dementia background and it was so nice because I keep getting this lady coming in frequently into the GP practise because she's really struggling caring for her husband with dementia.

0:9:39.470 --> 0:9:39.830
Interviewer
Yeah.

0:9:53.820 --> 0:9:54.100
Interviewer
Yeah.

0:9:30.970 --> 0:9:57.770
Respondent
And I said just you come to the hub, you know, because she said she asked her husband to come and and and he was like no. And that's the other thing, actually. I'll, I'll go back to that in a minute. So she turned up to the hub and it was so lovely because she spoke the whole time to the lady from adult social services and the PC, the PCSO, who also comes the one that you know, wanted to set this up for ages. And I thought, how nice is that, like for her to get the support?

0:9:58.750 --> 0:9:59.950
Respondent
But going back to.

0:10:0.30 --> 0:10:1.710
Interviewer
It's only one place as well.

0:10:0.420 --> 0:10:6.980
Respondent
Yeah, yeah. And and I think like, it works really well. It's not just a memory calf. It's also.

0:10:20.700 --> 0:10:21.140
Interviewer
Yeah.

0:10:8.500 --> 0:10:29.300
Respondent
Again, it's that multi agency support. So a lot I get a lot of feedback from carers that say, oh, it's so hard to get hold of adult social services and then they come to the hubs and they actually meet a human person from adult social services, not not a phone, you know, not a phone call. So but going back to the also.

0:10:30.940 --> 0:10:35.340
Respondent
Patients refusing to want to go to anything. That's the other thing as well. So like.

0:10:36.980 --> 0:10:38.700
Respondent
You know, often with dementia.

0:10:40.740 --> 0:10:46.100
Respondent
People can sort of refuse any kind of like suggestions and and.

0:10:47.540 --> 0:10:54.780
Respondent
Yeah, I mean, my my grandmother had dementia. And so that's my kind of personal experience of it.

0:10:55.700 --> 0:10:55.900
Interviewer
Mm hmm.

0:10:55.780 --> 0:11:1.820
Respondent
But yeah, it's it's difficult because again, you know it, it's.

0:11:3.20 --> 0:11:4.740
Respondent
If they don't want to do it.

0:11:3.10 --> 0:11:13.130
Interviewer
Let me ask you how how do you sort of address issues around rejection of care when you're putting somebody living with dementia?

0:11:23.840 --> 0:11:23.960
Respondent
Hmm.

0:11:14.730 --> 0:11:31.810
Interviewer
Keeping in mind obviously that you know how dementia is, maybe one day is a no two days later could be a yes. It could be, then a no and a yes, it fluctuates a lot. So I wonder as a social prescriber, how do you?

0:11:32.440 --> 0:11:40.360
Interviewer
Sort of work around the nose or or around, you know, keeping a referral open.

0:11:41.760 --> 0:11:48.560
Interviewer
'Cause maybe. Then at some point the person might say yes, how do you actually work around all of these issues that are typical of dementia?

0:12:1.620 --> 0:12:1.980
Interviewer
No.

0:11:47.680 --> 0:12:12.520
Respondent
So that's that's a really that is a really difficult one because it's the capacity again. And if people say no like well if people say no in a in general as a social prescriber, we don't have to work with them. So we're not a statutory service where we, you know, but if if it was the point where you know to self neglect or they're at risk to themselves, then you have to raise it as a safeguarding.

0:12:14.80 --> 0:12:17.800
Respondent
And then that's the time when you can override capacity. Sorry.

0:12:18.210 --> 0:12:25.810
Respondent
To provide consent if it's if there are risk to themselves. So I have had to do that, have had to raise safeguarding.

0:12:27.570 --> 0:12:33.210
Respondent
There was something else that just came to mind actually, which was a really important fact. I wanted to talk. That's gone from my head now.

0:12:36.860 --> 0:12:40.540
Interviewer
I know it happens all the time to me as well, yeah.

0:12:46.250 --> 0:12:46.530
Interviewer
No.

0:12:35.530 --> 0:12:46.690
Respondent
Oh, that's annoying. When that happens, it might come, it might come back in a minute. Yeah. So, yeah, that's always a tricky one. Oh, yeah, that's it. Sorry if I'm talking too much as well. I do have to go on.

0:12:48.120 --> 0:12:49.40
Respondent
I had a case.

0:12:50.820 --> 0:12:56.380
Respondent
Oh, I had a crazy case like, so I had a safeguarding concern from.

0:12:58.200 --> 0:12:58.240
Respondent
A.

0:12:59.390 --> 0:13:6.30
Respondent
It was a local food bank or they were like a food larder. And they said, oh, we're really worried about this woman.

0:13:7.310 --> 0:13:9.510
Respondent
She she's living in a in a year, in a field.

0:13:11.150 --> 0:13:20.590
Respondent
And apparently it's like damaged and all of this. And I was like, oh, OK, I said, can I have the address? And so they gave me this address and it was a Harry Potter address.

0:13:22.200 --> 0:13:37.560
Respondent
And it was like [anonymised], I think. And I couldn't find this person. And. And they said, oh, no, she's registered at your practise. And I could not find this person. And I started getting really worried. You know, when you've had this concern and you can't track this person down.

0:13:37.620 --> 0:13:37.940
Interviewer
Yeah.

0:13:38.940 --> 0:13:49.220
Respondent
So I said to the food ladder. I said look, when she comes again, can you ask her to give me a ring? Because I can't find her on the system at all. So about 2 weeks later, this woman gives me a ring.

0:13:50.420 --> 0:14:6.660
Respondent
And you know when you just got alarm bells ringing and you think something's going on here, doesn't feel right. And I said, I said, well, what is your address? And it was this whole address. But she basically took a took a post code from the neighbour's house and was living in this this year that that had been damaged by the storm.

0:14:8.660 --> 0:14:18.260
Respondent
And and then the reason she hadn't been to the GP GP practise for 10 years and was still in her maiden name. So that's why I couldn't find her on the system.

0:14:19.740 --> 0:14:20.300
Respondent
Anyway.

0:14:21.290 --> 0:14:44.650
Respondent
I I ended up getting quite a few organisations involved and one of them was Saint Patrick's, which is like a homeless agency and they went out to visit her and they raised this concern that she was living in this damaged year hoarding. There was rats and they said it wasn't even habitable, like it wasn't even fit for habitation. So I I did a safeguarding.

0:14:45.790 --> 0:15:14.350
Respondent
But again, because she hadn't been to the GP practise for 10 years, there was nothing on her record. There was no medical, there was no learning disabilities, nothing so adult social services wouldn't open up this case. And I was like, there's really something going on with this woman. Like I I I really, really kept fighting this this woman's cause and saying that this woman really needs help with something going on. And she had a car accident and she couldn't remember how she'd had a car accident. Her car was in this ditch.

0:15:29.940 --> 0:15:30.740
Interviewer
Well, yeah.

0:15:15.430 --> 0:15:46.190
Respondent
So I ended up getting on to [anonymised] Housing again. [anonymised] Housing wouldn't support this woman because they said she she we we can't see it as a like priority need. She's not got, you know. And I was thinking well, there is something going on. So in the end in the end I was trying to get all these services and I found the service called [anonymised] which was another homeless service. But they do assessments and the daughter was getting really worried. The daughter said look, we're really worried about my mum. Like she she doesn't know.

0:15:46.390 --> 0:16:16.550
Respondent
You know what's going on her? Her memory's not great. She's had this car accident. Nobody knows what's going on. She's living in this terrible condition. Nobody's helping her. And or none of these agencies would help because there was no diagnosis or nothing on the record. So I got the service that did diagnosis and they couldn't. They couldn't get hold of her for a long time and it took about 3 attempts to to get this. This kind of diagnosis. It was through the mental health team to start off with. Eventually they did it.

0:16:17.500 --> 0:16:26.740
Respondent
And then it came up that there was a memory concern, and then she actually ended up having a dementia assessment and it and it. And it transpired that she had dementia.

0:16:28.940 --> 0:16:44.140
Respondent
And once she had that diagnosis, all those services then got on board and and started to help her. But it got to that, you know, it took ages to get to that stage to help this person.

0:16:45.60 --> 0:16:45.180
Interviewer
Hmm.

0:16:46.100 --> 0:16:46.700
Respondent
And then.

0:16:47.20 --> 0:16:51.540
Respondent
Yeah. And then it was just, you know, then then she was classed as.

0:16:53.500 --> 0:16:55.500
Respondent
Priority need with housing.

0:16:55.820 --> 0:17:10.740
Respondent
She she got, like a needs assessment and all of that process could happen. But again, I just think as these people slipping through the net, there's there's so many people slipping through the net and there's so many people that aren't even identified by services.

0:17:11.100 --> 0:17:11.820
Interviewer
Yeah, yeah.

0:17:12.650 --> 0:17:29.130
Interviewer
What I've heard a lot in some other services is that there are particular communities who may also be resistant to accessing primary care through their GPS because of, you know, historical.

0:17:33.900 --> 0:17:34.20
Respondent
Hmm.

0:17:39.700 --> 0:17:40.20
Respondent
Yeah.

0:17:30.730 --> 0:17:46.930
Interviewer
Discrimination etcetera culture as well. And it is those people that actually in most cases sleep through the prax as you're saying. And again, those people who told me about this, they emphasised the importance.

0:17:54.680 --> 0:17:54.800
Respondent
Hmm.

0:18:1.640 --> 0:18:2.800
Respondent
Yeah, yeah.

0:17:47.310 --> 0:18:10.270
Interviewer
Of their presence in community hubs or community events where they can really sort of promote social prescribing in a venue that's different from asking people to access your GP, and that enables people to be included in that support that they would otherwise probably miss out on.

0:18:11.10 --> 0:18:16.650
Respondent
Yeah, definitely. And that's that's why the information Hub started and and going back.

0:18:18.170 --> 0:18:36.330
Respondent
To that point I I work in an area of [anonymised] that's extremely deprived and and and there's a there's a there's a streak called [anonymised]. And on [anonymised], there's static homes, and there must be over.

0:18:36.410 --> 0:18:38.50
Respondent
2000 residents and.

0:18:39.610 --> 0:18:40.770
Respondent
We've got migrant workers.

0:18:47.300 --> 0:18:47.460
Interviewer
Well.

0:18:41.640 --> 0:18:49.160
Respondent
We've got unfortunately still modern slavery going on, and some of these factory and work houses and the PCSOs and the police are aware of this.

0:18:50.480 --> 0:19:7.520
Respondent
And and all they can do is hand out leaflets of organisations that support people we've had trafficking going on. We've had it's it's horrendous, like it's horrendous. But most of those people on that lane are not registered with the GP practise because they can't even speak English.

0:19:9.0 --> 0:19:10.840
Respondent
So we did a, we did an outreach.

0:19:12.40 --> 0:19:19.520
Respondent
It was actually an outreach vaccination clinic and my colleague organised it and we had three translators.

0:19:20.880 --> 0:19:29.200
Respondent
We had all the organisations so I worked really closely with Citizens Advice and the lovely lady or lovely 2 ladies that did all the pre settlement.

0:19:32.0 --> 0:19:40.760
Respondent
Like status to make sure all the workers had there because a lot of people didn't even know that they had to apply. So Citizens Advice actually work on foot.

0:19:41.400 --> 0:20:3.920
Respondent
In this community and go round and work with all them, they're still doing it now as well actually, even though it's past the thing, that's they still go round and doing this work and it's fantastic work. And yeah, we did this. Drop it, we did this drop in on on the lane. So we just had a van and and I was shocked.

0:20:4.770 --> 0:20:11.650
Respondent
I mean, I couldn't believe that this was going on in the in, in the UK, the amount of poverty.

0:20:21.290 --> 0:20:21.410
Interviewer
Hmm.

0:20:13.130 --> 0:20:29.330
Respondent
There was a man had broken his arm and he didn't even know how to contact a doctor. He did, wasn't even registered with the doctor. So we we like one of my colleagues whose medical she basically arranged for him to go to A&E.

0:20:29.370 --> 0:20:31.290
Respondent
And we got him registered with the practise.

0:20:33.50 --> 0:20:39.410
Respondent
And and he was so grateful because he came back. He'd gone to it, and he came back when we're still there. And he'd had a cast put on his arm.

0:20:52.680 --> 0:20:53.0
Interviewer
Yeah.

0:20:39.930 --> 0:21:9.290
Respondent
There was a man that needed emergency dental, and again he couldn't speak English and couldn't contact any services, and so that that made me recognise that there was a need for outreach work. So then I I am I got given this van by this local community hall and the lady came with me. That was the manager and we took some cakes and tea and we again brought all the agencies. And again with Citizens Advice.

0:21:10.90 --> 0:21:18.650
Respondent
And we went down to the lane with this fan and it was really good because people were coming up to us and we could link them up to all this information, advice and support.

0:21:20.490 --> 0:21:25.210
Respondent
And you know, again with dementia, there must be residents on that lane.

0:21:26.650 --> 0:21:35.850
Respondent
That could be on their own, could be have a multitude of like health factors going on and a lot of services won't work on that lane.

0:21:36.390 --> 0:21:36.630
Interviewer
Yeah.

0:21:36.250 --> 0:21:38.890
Respondent
It won't go down on their own.

0:21:38.930 --> 0:21:44.250
Respondent
Because there's, there's, there's quite a lot of crime on that lane as well.

0:21:45.950 --> 0:21:55.870
Respondent
It's owned by a traveller community, the site, but the residents are, you know, there's a there's a whole range of residents and.

0:22:3.20 --> 0:22:3.300
Interviewer
Yeah.

0:21:58.130 --> 0:22:7.810
Respondent
You know, there's people that have ended up there because there's no housing options in [anonymised] as well. So yeah, that that there's huge health inequalities there.

0:22:9.190 --> 0:22:9.310
Respondent
And.

0:22:12.940 --> 0:22:13.300
Respondent
Yeah.

0:22:37.330 --> 0:22:37.770
Respondent
Yeah.

0:22:8.460 --> 0:22:40.380
Interviewer
It shows it kind of shows the importance of outreach work within social prescribing because lots of people do not even have a clue of, you know, how to access it, let alone what it is. Some people just don't even know what social prescribing is. So within these communities in particular, I can only imagine the distance from actually, you know, accessing the service that they might experience. And I suppose this also shows the importance of.

0:22:40.580 --> 0:23:7.220
Interviewer
And pathways to referral into social prescribing, because we might tend to assume that, you know, GP is universal, everybody has GP, everybody access their GP. It's not the case you you're saying to me it's not the case at all. So we need to find ways in which we can reach out to people but also ensure that referrals can happen in different ways through different sources.

0:23:6.460 --> 0:23:11.540
Respondent
Yeah, yeah, there was a. There was a company that set up an AI.

0:23:13.300 --> 0:23:22.580
Respondent
On WhatsApp so that you could ask this AI and they they found out all the agencies and organisations.

0:23:24.220 --> 0:23:27.180
Respondent
But the only thing with that is is digital.

0:23:28.780 --> 0:23:35.780
Respondent
Like a lot of people aren't even online, a lot of people don't even have the Internet. Some people don't even have a phone that I support.

0:23:36.540 --> 0:23:55.260
Respondent
So I did do the digital champion training and I have got laptops and tablets I can loan to people now which is great and my fives as well little Internet things. So that's been amazing. And you know if that could help people and well if that could help people to mention their carers as well.

0:23:56.820 --> 0:24:7.60
Respondent
That's the other thing you know to to get online training because there's a there's an organisation called Pro Promus in [anonymised] and they do training for carers.

0:24:7.700 --> 0:24:8.820
Interviewer
Right. OK.

0:24:9.640 --> 0:24:12.600
Respondent
So that they're they're coming next month actually to do a little talk.

0:24:21.300 --> 0:24:21.380
Interviewer
Mm.

0:24:30.560 --> 0:24:30.760
Interviewer
Mm hmm.

0:24:14.280 --> 0:24:33.520
Respondent
But you know some of those courses are online so that that digital inclusion is really important as well because like I said, you know the area where I work of [anonymised] is also a mining area. So when the mines closed, a lot of people lost their jobs and a lot of people can't even read and write.

0:24:35.480 --> 0:24:39.600
Respondent
You know that there's there's real deprivation and there's real.

0:24:39.900 --> 0:24:41.380
Respondent
Lack of employment and.

0:24:43.220 --> 0:24:57.380
Respondent
Yeah. So much going on in these little villages that a lot of people, you know, a lot of feedback from my services that they have no idea if something like that existed. And it's been it's been so great.

0:24:58.820 --> 0:25:12.780
Respondent
Having social prescribing and I absolutely love my job, I know it's been hard at times, but I the thing I love most about my job is the fact that it's that linking people to things that they didn't realise we're out there.

0:25:13.400 --> 0:25:13.680
Interviewer
Yeah.

0:25:14.330 --> 0:25:20.570
Respondent
And you know, seeing how just by providing them that information.

0:25:21.950 --> 0:25:25.230
Respondent
How much it can make such a huge impact on someone's life and?

0:25:25.340 --> 0:25:25.580
Interviewer
Yeah.

0:25:27.880 --> 0:25:31.760
Respondent
You know, I think maybe that is why services have.

0:25:32.760 --> 0:25:38.640
Respondent
But they can kind of think that social prescribers can fix everything. But but we can't, you know, but.

0:25:58.180 --> 0:25:58.500
Interviewer
OK.

0:25:40.240 --> 0:26:4.360
Respondent
The people we can help, it's been a been a massive difference and I think like I said, you know, if there were more services to help those that can't access those, those things independently then then that would be fantastic. I was, I was involved with the involved years ago. I was a project worker for V involved and that that was.

0:26:5.430 --> 0:26:34.630
Respondent
Set up to support people with learning disabilities or or mental health that couldn't go volunteering on their own. So my role was to recruit people that needed that help to go to a volunteer project and then we would set up a volunteer project and arrange for all those people to come. And it's almost something like that for people with dementia, where it was an organisation that identified people that were living on their own with no support that could either.

0:26:39.800 --> 0:26:40.120
Interviewer
Yeah.

0:26:36.520 --> 0:26:54.880
Respondent
You know, maybe pick them up in a minibus and take them to to to a place where they have that support, that that, you know, it was like at the Dementia cafe of the day. The man said that one of the carers said that when his wife is at home, she she's completely different to when she comes to the memory calf.

0:26:56.190 --> 0:26:56.390
Interviewer
Mm hmm.

0:26:56.560 --> 0:27:6.840
Respondent
And when she comes to the memory calf she was drawing and singing and he said she doesn't do that at home. And I think it's taking people out of that like almost like that.

0:27:9.760 --> 0:27:11.480
Interviewer
Yeah, that isolation as well.

0:27:23.500 --> 0:27:26.20
Interviewer
Yeah, I suppose particularly.

0:27:7.410 --> 0:27:26.970
Respondent
Mundane feeling of being stuck at home. Isn't it? Like, you know? Yeah, yeah. And and taking, yeah, people to a place where they can integrate with others and have that support. And you know, we we need we need more of that really. And and it's always that transport is the biggest thing.

0:27:34.340 --> 0:27:34.420
Respondent
Mm.

0:27:27.270 --> 0:27:55.950
Interviewer
Yeah, and I suppose it's particularly true in especially remote areas or rural areas where this taking somebody to somewhere is quite important. Where, you know, there's less availability of public transport or maybe they do not have anybody who literally takes them, they they might not have family or so yeah, it is crucial to to have that, that infrastructure there. I think it's very important.

0:28:4.980 --> 0:28:5.180
Interviewer
Mm hmm.

0:27:55.420 --> 0:28:26.20
Respondent
Yeah. And there used to be a lovely project with [aonnymised] where it was for anybody over 65 that that were that lived on their own and they got funding for a minibus and they used to pick people up from home and they would pay something like £5 or something and they would take them to [aonnymised] and then they would have a little walk around the [anonymised]. And then they would have lunch together and then they would take them home again. And that was one of the loveliest projects.

0:28:26.710 --> 0:28:34.390
Respondent
Because those people were, it was for people that, you know, couldn't drive and were literally stuck out. I mean, I got a lady that went to it that lives on the Moor.

0:28:34.920 --> 0:28:35.120
Interviewer
Yeah.

0:28:35.770 --> 0:28:51.890
Respondent
And you know, she's housebound and she can't get out otherwise. And that was her, like Lifeline, you know, and then unfortunately, it came to an end. But that was such a fantastic project. And I mean, the only other service that does that is age uk, but.

0:28:52.890 --> 0:28:59.690
Respondent
That picks people up and takes people to a day centre, but that isn't for like everyone, you know, it's.

0:28:58.960 --> 0:29:0.520
Interviewer
Yeah, yeah.

0:29:2.310 --> 0:29:33.790
Interviewer
It's it's important to to keep in mind that there's a huge diversity of needs and preferences and people out there and and one thing that needs, you know, seems to be quite important looking forward-looking ahead for social prescribing is that kind of specialisation that you talked about at the beginning of the interview so that you know different needs in different people can be addressed by, you know, some some sort of.

0:29:33.830 --> 0:29:34.910
Interviewer
Workers who have a specialisation.

0:29:35.580 --> 0:29:45.180
Interviewer
In in a specific in a specific area. So that's something that came up in my interviews as well. But some of the topics that you touched upon.

0:29:46.780 --> 0:29:56.340
Interviewer
They really echo what I've heard from others, so this gives me sort of substantial backup evidence as to what the current issues are.

0:29:58.140 --> 0:30:9.140
Interviewer
Particularly around, as you said, supporting somebody who's not necessarily early stage but a bit later on all the issues around, you know, rejection, rejection of care.

0:30:9.450 --> 0:30:19.930
Interviewer
And how to ensure that the person really is engaged in the process of, you know, of Co developing a care package essentially.

0:30:21.370 --> 0:30:27.690
Interviewer
So that's also kind of recurring. All the issues around you know.

0:30:36.310 --> 0:30:36.710
Respondent
Yeah.

0:30:27.770 --> 0:30:40.810
Interviewer
Inequality of access for different communities that also frequently came up as well as the important outreach work. And I I hear that lots and lots of practises are actively attending.

0:30:50.740 --> 0:30:50.980
Respondent
Yeah.

0:30:41.740 --> 0:31:11.860
Interviewer
Days events in you know communities to promote social prescribing and make sure that people know that they are there if they want to and and self referral is also something that's kind of developing looking ahead so that people do not do not have to go through the you know primary care referral pathway and they can directly self refer so all these things Fiona they're coming up from from other interviews as well so.

0:31:12.660 --> 0:31:17.700
Interviewer
It was good today because it kind of confirmed, even from the viewpoint of somebody working.

0:31:18.780 --> 0:31:45.420
Interviewer
A bit more rurally in [anonymised] cause I've heard from lots of people from, you know, London, Manchester, Newcastle. So it's important that we've had this conversation today together because I want to represent the views of those who are also working, you know, other localities. So yeah, so it it was great. I was wondering, do you have any kind of final points that you wanted to mention before I wrap it up?

0:31:46.440 --> 0:32:3.920
Respondent
So just actually on the point of the lady I said about, you know, that got the diagnosis through that organisation. I forgot to say, actually she was very apprehensive, not apprehensive. She she actually refused to get any support initially. She said well, there's nothing wrong with me. My memory's fine.

0:32:5.360 --> 0:32:15.400
Respondent
And so sorry, I forgot to mention that was quite a big factor that it took quite a long time to encourage her to get a diagnosis. And I had to get this daughter involved and.

0:32:16.180 --> 0:32:27.540
Respondent
We had to have like a multi agency meeting with the lady to to discuss our concerns and only then she, you know, consented. But I think it is that capacity consent.

0:32:29.820 --> 0:32:38.940
Respondent
Yeah, it is. Is one of the major, major issues and and and for my area, definitely transport and and resources and.

0:32:40.700 --> 0:32:49.420
Respondent
Yeah. And one other thing I wanted to just mention as well. Do you know about the Brazilian health model because that's being adopted that's being used in [anonymised]?

0:32:50.30 --> 0:32:51.590
Interviewer
OK, tell me about it.

0:32:52.260 --> 0:32:52.380
Respondent
And.

0:32:52.580 --> 0:32:55.140
Respondent
So I don't really know much about it myself, but.

0:32:57.740 --> 0:33:3.340
Respondent
Certain pcns have got funding for they're called like health and well-being workers.

0:33:4.50 --> 0:33:8.970
Respondent
And these health and well-being workers actually go out to the communities and work with families.

0:33:9.210 --> 0:33:9.450
Interviewer
Uh huh.

0:33:10.330 --> 0:33:18.810
Respondent
But our practise hasn't gotten any and when I when I asked them, it said it's something to do with people that deprived post codes.

0:33:21.60 --> 0:33:41.580
Respondent
And but I I I my understanding was it that it was deprived postcodes of people that are registered, and I highlighted that this meeting I said but my area of [anonymised] is one of the highest deprived areas of [anonymised]. Why have we not got one health and well-being worker? And they said it's probably because they're not registered at practises. So the data's not come through.

0:33:42.860 --> 0:33:45.700
Interviewer
Right, OK. So are you saying that?

0:33:52.330 --> 0:33:52.730
Interviewer
Yeah.

0:33:43.520 --> 0:33:55.240
Respondent
And so we haven't been but, but I don't, I don't know much about it, but it might be worth if you get a chance to look it up. I'll see if I can find it on here, what it's called.

0:33:57.520 --> 0:34:1.360
Respondent
But it's something to do with central weather like central.

0:34:10.990 --> 0:34:11.590
Interviewer
OK.

0:34:3.40 --> 0:34:22.120
Respondent
Data or something of registrations, but because a lot of mine aren't registered, it's not. Yeah, I don't know. I it's. I need to find out. We've got a new PCN manager that I'm having a meeting about this because I've not really been explained very well on on. You know what it's all about.

0:34:24.480 --> 0:34:28.0
Interviewer
So is this specific to health and well-being coaches roles?

0:34:28.650 --> 0:34:30.90
Respondent
Yeah, yeah.

0:34:30.520 --> 0:34:31.200
Interviewer
OK.

0:34:31.930 --> 0:34:34.930
Respondent
So, oh, here it is. Is it this one?

0:34:47.470 --> 0:34:47.670
Interviewer
Mm hmm.

0:34:36.950 --> 0:34:47.950
Respondent
Yeah. So they they're in [anonymised]'s investing in loads of them and I'm just thinking, I mean that might be a positive thing for people that are on their own.

0:34:47.990 --> 0:34:51.670
Respondent
But again, I don't know what the whole kind of criteria of it.

0:34:54.320 --> 0:34:57.360
Respondent
Oh yeah, here it. Here it is. I'll send you.

0:34:57.730 --> 0:34:59.610
Interviewer
Yeah, that would be great. So I can.

0:34:58.890 --> 0:35:1.90
Respondent
I'll send you the link to this.

0:35:2.430 --> 0:35:6.510
Respondent
Yeah, it's the Brazilian model of community health and well-being.

0:35:6.940 --> 0:35:7.620
Interviewer
OK.

0:35:7.820 --> 0:35:9.100
Respondent
In primary care.

0:35:10.560 --> 0:35:18.760
Respondent
But I think it's that case that there is a primary care, so the deprived post codes have been identified through the registration process, but.

0:35:18.780 --> 0:35:21.20
Interviewer
Actually, I suspect that's the case.

0:35:28.490 --> 0:35:29.450
Interviewer
Yeah, yeah.

0:35:21.410 --> 0:35:31.970
Respondent
And I think that's why I've been left out. But again, that research should maybe be more outreach and getting clearer data kind of thing. I'll pop this in the chat.

0:35:32.560 --> 0:35:38.120
Interviewer
Thank you. Yeah, I'll look it up. 'cause. I've I. It's the first time to be honest. I've heard about this.

0:35:39.480 --> 0:35:40.0
Interviewer
Brazilian.

0:35:42.170 --> 0:35:43.850
Interviewer
Model of of care.

0:35:48.270 --> 0:35:48.830
Interviewer
Yeah.

0:35:44.420 --> 0:35:50.220
Respondent
I'll just see if I can find it relating to [anonymised] as well, because they're doing a huge on it in [anonymised].

0:35:58.750 --> 0:36:1.790
Respondent
If I find out some more stuff in oh.

0:36:5.580 --> 0:36:13.380
Respondent
Yeah, if I find out some more stuff relating to how it's, I think it's through volunteer [anonymised], actually.

0:36:14.900 --> 0:36:16.940
Respondent
I think they're coordinating it all.

0:36:19.860 --> 0:36:20.540
Interviewer
That's really.

0:36:23.930 --> 0:36:24.130
Interviewer
Mm hmm.

0:36:19.860 --> 0:36:24.780
Respondent
But that could be a that could be a really good resource if.

0:36:24.820 --> 0:36:27.580
Respondent
They can support people with dementia as well.

0:36:31.540 --> 0:36:35.140
Interviewer
I'm just looking at the document you sent.

0:36:35.930 --> 0:36:36.810
Respondent
Oh, here it is.

0:36:35.800 --> 0:36:37.760
Interviewer
And it looks really.

0:36:39.920 --> 0:36:44.480
Interviewer
Interesting. And you? Apparently they're piloting this in [anonymised].

0:36:46.190 --> 0:36:49.190
Interviewer
Or at least this this document says so.

0:37:5.760 --> 0:37:8.880
Respondent
Yeah, if I'll see if I can find it more relating to.

0:37:10.150 --> 0:37:10.670
Interviewer
Yeah.

0:37:11.190 --> 0:37:13.910
Respondent
I think it is a a national thing anyway.

0:37:15.430 --> 0:37:17.990
Respondent
They're rolling out all free the UK.

0:37:20.480 --> 0:37:23.600
Respondent
Because it was a real success in Brazil.

0:37:24.460 --> 0:37:24.780
Interviewer
Yeah.

0:37:26.350 --> 0:37:34.910
Interviewer
Yeah, if if you find anything more about about this, do you do you let me know. I'll do the same. 'cause. I'm gonna look into this.

0:37:35.440 --> 0:37:35.960
Respondent
Yeah.

0:37:36.570 --> 0:37:38.410
Interviewer
'Cause that might be really helpful.

0:37:42.290 --> 0:37:42.970
Interviewer
Oh.

0:37:37.150 --> 0:37:49.750
Respondent
Yeah, I'll send. I'll send that over. Yeah, because I I we're having a meeting about it. So I can. I can pop over some information. 'cause. I'm hoping we're gonna get them because especially if they do outreach, we desperately need that.

0:37:50.180 --> 0:37:50.460
Interviewer
Yeah.

0:37:51.270 --> 0:37:56.710
Respondent
Especially for that area, I was talking to you about. Like, there's. Yeah, huge health deprivation there.

0:37:58.920 --> 0:38:8.680
Interviewer
Yeah, I I would say let's let's keep in touch with updates around this if if we're able to find something more around it.

0:38:10.80 --> 0:38:11.360
Interviewer
OK. Thank you.

0:38:7.80 --> 0:38:12.0
Respondent
Yeah. Yeah, of course. Yeah. Brilliant. And I'll do the forms for you.

0:38:14.180 --> 0:38:14.340
Respondent
Yeah.

0:38:21.640 --> 0:38:22.440
Respondent
Oh, OK.

0:38:12.210 --> 0:38:23.490
Interviewer
Oh, that's very helpful. Thank you so much. You if you, if you have any difficulties filling them in with the computer, you can print them out, fill them in by hand, even take a photo and send it back is fine.

0:38:23.910 --> 0:38:31.510
Respondent
OK, brilliant. Yeah, some of the areas that I work in the we still don't have very good Internet. It's like being back in the dark ages.

0:38:29.890 --> 0:38:32.810
Interviewer
Yeah, I know.

0:38:33.50 --> 0:38:33.650
Respondent
But that's great.

0:38:32.850 --> 0:38:34.290
Interviewer
That's why I always keep the photo option.

0:38:36.190 --> 0:38:39.190
Interviewer
Yeah, alright, you too, Fiona.

0:38:35.70 --> 0:38:40.790
Respondent
Yeah, yeah. Oh, nice to meet you anyway. And yeah, you take care.

0:38:42.230 --> 0:38:43.870
Respondent
Thanks then. Bye bye bye.

0:38:40.800 --> 0:38:44.0
Interviewer
You too. Thanks so much. Bye. Take care. Bye bye.
